# An Energy-Reduced Mediterranean Diet, Physical Activity, and Body Composition: An Interim Subgroup Analysis of the PREDIMED-Plus Randomized Clinical Trial

**DOI:** 10.1001/jamanetworkopen.2023.37994

**Published:** 2023-10-18

**Authors:** Jadwiga Konieczna, Miguel Ruiz-Canela, Aina M. Galmes-Panades, Itziar Abete, Nancy Babio, Miquel Fiol, Vicente Martín-Sánchez, Ramón Estruch, Josep Vidal, Pilar Buil-Cosiales, Jesús F. García-Gavilán, Manuel Moñino, Alba Marcos-Delgado, Rosa Casas, Romina Olbeyra, Montserrat Fitó, Frank B. Hu, Miguel Ángel Martínez-Gonzalez, J. Alfredo Martínez, Dora Romaguera, Jordi Salas-Salvadó

**Affiliations:** 1 Research Group on Nutritional Epidemiology & Cardiovascular Physiopathology, Health Research Institute of the Balearic Islands, University Hospital Son Espases, Palma de Mallorca, Spain; 2 Centro de Investigación Biomédica en Red Fisiopatología de la Obesidad y la Nutrición, Institute of Health Carlos III, Madrid, Spain; 3 Department of Preventive Medicine and Public Health, IDISNA, University of Navarra, Pamplona, Spain; 4 Global Health Research Group, Health Research Institute of the Balearic Islands, University of the Balearic Islands, Palma de Mallorca, Spain; 5 Department of Nutrition, Food Sciences, and Physiology, University of Navarra, Pamplona, Spain; 6 Universitat Rovira i Virgili, Departament de Bioquímica i Biotecnologia, Alimentaciò, Nutrició Desenvolupament i Salut Mental ANUT-DSM, Reus, Spain; 7 Institut d’Investigació Pere Virgili, Reus, Spain; 8 CIBER de Epidemiología y Salud Pública, Instituto de Salud Carlos III, Madrid, Spain; 9 Institute of Biomedicine, University of León, León, Spain; 10 Department of Internal Medicine, IDIBAPS, Hospital Clinic, University of Barcelona, Barcelona, Spain; 11 Institut de Recerca en Nutrició i Seguretat Alimentaria, University of Barcelona, Barcelona, Spain; 12 CIBER Diabetes y Enfermedades Metabólicas, Instituto de Salud Carlos III, Madrid, Spain; 13 Department of Endocrinology, Institut d’Investigacions Biomédiques August Pi Sunyer, Hospital Clinic, University of Barcelona, Barcelona, Spain; 14 Primary Care Services, Navarra Regional Health Service, Pamplona, Spain; 15 Cardiovascular Risk and Nutrition Research Group, Hospital del Mar Medical Research Institute, Barcelona, Spain; 16 Channing Division of Network Medicine, Department of Medicine, Brigham & Women’s Hospital and Harvard Medical School, Boston, Massachusetts; 17 Department of Nutrition, Harvard T. H. Chan School of Public Health, Boston, Massachusetts; 18 Precision Nutrition and Cardiometabolic Health Program, IMDEA Food, CEI UAM + CSIC, Madrid, Spain

## Abstract

**Importance:**

Strategies targeting body composition may help prevent chronic diseases in persons with excess weight, but randomized clinical trials evaluating lifestyle interventions have rarely reported effects on directly quantified body composition.

**Objective:**

To evaluate the effects of a lifestyle weight-loss intervention on changes in overall and regional body composition.

**Design, Setting, and Participants:**

The ongoing Prevención con Dieta Mediterránea-Plus (PREDIMED-Plus) randomized clinical trial is designed to test the effect of the intervention on cardiovascular disease prevention after 8 years of follow-up. The trial is being conducted in 23 Spanish research centers and includes men and women (age 55-75 years) with body mass index between 27 and 40 and metabolic syndrome. The trial reported herein is an interim subgroup analysis of the intermediate outcome body composition after 3-year follow-up, and data analysis was conducted from February 1 to November 30, 2022. Of 6874 total PREDIMED-Plus participants, a subsample of 1521 individuals, coming from centers with access to a dual energy x-ray absorptiometry device, underwent body composition measurements at 3 time points.

**Intervention:**

Participants were randomly allocated to a multifactorial intervention based on an energy-reduced Mediterranean diet (MedDiet) and increased physical activity (PA) or to a control group based on usual care, with advice to follow an ad libitum MedDiet, but no physical activity promotion.

**Main Outcomes and Measures:**

The outcomes (continuous) were 3-year changes in total fat and lean mass (expressed as percentages of body mass) and visceral fat (in grams), tested using multivariable linear mixed-effects models. Clinical relevance of changes in body components (dichotomous) was assessed based on 5% or more improvements in baseline values, using logistic regression. Main analyses were performed in the evaluable population (completers only) and in sensitivity analyses, multiple imputation was performed to include data of participants lost to follow-up (intention-to-treat analyses).

**Results:**

A total of 1521 individuals were included (mean [SD] age, 65.3 [5.0] years; 52.1% men). In comparison with the control group (n=761), participants in the intervention arm (n=760) showed greater reductions in the percentage of total fat (between group differences after 1-year, −0.94% [95% CI, −1.19 to −0.69]; 3 years, −0.38% [95% CI, −0.64 to −0.12] and visceral fat storage after 1 year, -126 g [95% CI, −179 to −73.3 g]; 3 years, −70.4 g [95% CI, −126 to −15.2 g] and greater increases in the percentage of total lean mass at 1 year, 0.88% [95% CI, 0.63%-1.12%]; 3-years 0.34% [95% CI, 0.09%-0.60%]). The intervention group was more likely to show improvements of 5% or more in baseline body components (absolute risk reduction after 1 year, 13% for total fat mass, 11% for total lean mass, and 14% for visceral fat mass; after 3-years: 6% for total fat mass, 6% for total lean mass, and 8% for visceral fat mass). The number of participants needed to treat was between 12 and 17 to attain at least 1 individual with possibly clinically meaningful improvements in body composition.

**Conclusions and Relevance:**

The findings of this trial suggest a weight-loss lifestyle intervention based on an energy-reduced MedDiet and physical activity significantly reduced total and visceral fat and attenuated age-related losses of lean mass in older adults with overweight or obesity and metabolic syndrome. Continued follow-up is warranted to confirm the long-term consequences of these changes on cardiovascular clinical end points.

**Trial Registration:**

isrctn.org Identifier: ISRCTN89898870

## Key Points

**Question:**

Does a 3-year weight-loss lifestyle intervention affect age-dependent changes in body composition?

**Findings:**

In this interim analysis of a randomized clinical trial including 1521 individuals, a 3-year intervention based on an energy-reduced Mediterranean diet and physical activity promotion, compared with advice to follow an ad libitum Mediterranean diet, reduced the total and visceral fat and attenuated the loss of lean mass in older adults with overweight or obesity and metabolic syndrome. These changes were likely of public health and even clinical relevance.

**Meaning:**

The findings of this study suggest that a combination of an energy-reduced Mediterranean diet and physical activity mitigates the potential negative effects of age-dependent changes in body composition; continued follow-up is warranted to confirm health consequences in the long term.

## Teaser

This randomized clinical trial examines changes in body composition components with use of a Mediterranean diet plus physical activity compared with ad libitum use of the Mediterranean diet in adults with overweight and obesity after 3 years.

## Introduction

Specific body composition components seem to be the key in the development of obesity-associated chronic diseases. In particular, excess visceral fat and loss of muscle mass, which occurs with increasing age, have been associated with a higher risk for a broad spectrum of health outcomes including cardiovascular disease and type 2 diabetes (T2D).^1,2^ Thus, effective strategies targeting specific body composition components beyond weight management are warranted to improve health in the long term.

The Mediterranean diet (MedDiet), and particularly an energy-reduced MedDiet, is emerging as an effective strategy for weight loss and its long-term maintenance.^3^ Results of the previous Prevención con Dieta Mediterránea (PREDIMED) trial that did not introduce any caloric reduction in the MedDiet after 5 years^4^ are also supportive that energy-reduced MedDiet might be an optimal approach for weight loss and cardiovascular disease prevention. In addition, a review of 18 intervention trials revealed that a MedDiet intervention, which either does or does not involve an energy-reduced focus, can significantly reduce measures of central obesity, as determined by waist circumference, waist-hip ratio, or visceral fat mass.^5^ On one hand, trials in this field are hampered by indirect measurement methods, small sample size, or short duration. On the other hand, there is an established positive effect of physical exercise on visceral fat in adults with overweight,^6^ and on muscle mass and function in healthy older adults.^7^ However, to our knowledge, the effects of a lifestyle intervention combining an energy-restricted MedDiet and physical activity (PA) on body composition in older individuals have not been examined.

The PREDIMED-Plus randomized clinical trial (RCT) provides a unique opportunity to assess the effects on body composition of multifactorial intervention with an energy-reduced MedDiet, PA promotion, and behavioral support for weight-loss goals in middle-aged and older adults with metabolic syndrome initially with overweight or obesity. In this study we aimed to determine the long-term effects of the lifestyle intervention on changes in age-related overall and regional body composition after the first 3 years of the PREDIMED-Plus trial.

## Methods

### Study Design

This study is a predefined interim analysis after 3 years of follow-up in a subset of participants in the ongoing PREDIMED-Plus RCT. The details on methods and procedures of the trial have been published elsewhere,^8,9,10^and the study protocol is available in Supplement 1. Briefly, PREDIMED-Plus is a multicenter, parallel-group, single-blinded for the staff and investigators trial aimed to assess the effects of weight-loss intervention with an energy-reduced MedDiet and PA vs usual care for primary cardiovascular disease prevention. The intervention is designed to be conducted during 6 years, with a further 2-year observational follow-up to collect clinical events. There are 2 primary end points: (1) incidence of cardiovascular disease (a composite of nonfatal myocardial infarction, nonfatal stroke, and cardiovascular death), and (2) weight loss and its long-term maintenance. Several secondary (ie, waist circumference, T2D, and mortality) and intermediate (ie, biochemical parameters and body composition) outcomes have been established, as indicated in the study protocol and the statistical analysis plan previously published.^9^ Participants were randomly allocated in a 1:1 ratio to 1 of the 2 study arms, using a computer-based system with stratification (center, sex, and age) in blocks of 6 participants. Couples sharing the same household were randomized together using the couple as a unit of randomization (in total, n = 404; in this subset, n = 61). The PREDIMED-Plus study protocol was reviewed and approved by the institutional review boards from all 23 participating centers. All participants provided written informed consent. No financial compensation is provided. The study reported herein was conducted from February 1 to November 30, 2022. This work followed the Consolidated Standards of Reporting Trials (CONSORT) reporting guideline.^11^

### Population and Analytical Sample

Participants were recruited between October 23, 2013, and December 23, 2016, in 23 Spanish centers. Eligible participants were middle-aged and older men (age 55-75 years) and women (age 60-75 years) without previous cardiovascular events. The inclusion criteria included the presence of overweight or obesity (body mass index for overweight: 27-29; for obesity: 30-39 [calculated as weight in kilograms divided by height in meters squared]) and metabolic syndrome.^12^

Of the total cohort (N = 6874), the subsample of participants (n = 1556) who underwent dual energy x-ray absorptiometry (DXA) measurements in 7 of the 23 recruiting centers having access to DXA scanners (DXA substudy) was used for the present study. In each of these 7 centers, either all or a subsample of participants were invited for DXA scans (details provided in eTable 1 in Supplement 2). To control for selection bias, characteristics of participants with DXA data were compared with those of the rest of participants enrolled in the PREDIMED-Plus trial and showed no meaningful differences (eTable 2 in Supplement 2).

We excluded individuals with missing values in variables of interest at baseline, resulting in 1521 participants included in the final analyses (intervention group, n = 760; control group, n = 761). The percentages of participants with the baseline DXA scans completing the follow-up visits were 79.9% for 1 year and 74.5% for 3 years (flowchart in eFigure 1 in Supplement 2).

### Intervention

Participants in the intervention group received a tailored face-to-face nutritional and behavioral program (details in eMethods in Supplement 2 and in previous publications^8,9,13^). In addition to 30% energy reduction, they were encouraged to limit consumption of some foods (processed meats, butter, margarine, cream, sweetened beverages, added sugar, biscuits, and bread and other refined cereals; whole grains were promoted) to better capture the caloric restriction applied to a MedDiet pattern^14^ for the purpose of weight loss. They were also encouraged to progressively increase aerobic PA, with the final goal of walking 45 minutes per day or more or the equivalent during 6 days per week, and doing exercises to improve strength, flexibility, and balance. Participants from the intervention group were contacted by trained dietitians 3 times a month (a group session, an individual session, and a telephone call) during the first year. They also received behavioral and motivational support strategies including self-monitoring, goal setting, and problem solving. Participants from the control group were given general advice to follow ad libitum the traditional MedDiet,^15,16^ without PA promotions, during group sessions twice a year.

### Body Composition

Body composition components (fat, lean, and bone mass) were measured with a third-generation DXA scanner (DXA Lunar Prodigy Primo and Lunar iDXA; GE Healthcare) connected with enCore software by trained operators at baseline, 1-year, and 3-year follow-up. Operational definition of regions of interest for body composition measured with DXA is available in the eMethods in Supplement 2.

For visceral fat measurements in an android region, DXA scans were reanalyzed using CoreScan software application (GE Healthcare) validated using computed tomographic scans as a reference tool.^17^ The primary end points were lean and fat mass expressed as the percentage of DXA-derived total body mass (sum of total bone, fat, and lean mass), to better capture changes in body composition proportions during weight loss, and visceral fat mass expressed in grams, given that it is the most usual measure for this body fat compartment. To deepen our understanding of body composition changes, other secondary outcomes were also examined: overall lean and fat mass expressed in absolute amounts (grams), and visceral fat expressed as the percentage of total fat mass. Furthermore, the ratio of android to gynoid fat was calculated to account for age-related adiposity redistribution from central to peripheral regions, as well as the ratio of lean mass to fat mass as a marker of sarcopenic obesity.

### Other Variables

A general questionnaire was administered to participants at baseline to collect data on sociodemographic characteristics and medical conditions. Smoking status was categorized as current, never, or former smoker, and educational level as higher education or technician, secondary education, and primary education or less. The prevalence of T2D was used as a dichotomous variable (Table 1 footnote).

**Table 1.** Baseline Characteristics of Study Participants With DXA Measurements by Study Group^a^

Baseline characteristic	Participants, No. (%)		
	All (N = 1521)	Intervention (n = 760)	Control (n = 761)
Sex			
Men	792 (52.1)	402 (52.9)	390 (51.3)
Women	729 (47.9)	358 (47.1)	371 (48.7)
Age, mean (SD), y	65.3 (5.0)	65.1 (5.1)	65.4 (4.9)
BMI, mean (SD)	32.5 (3.3)	32.6 (3.3)	32.4 (3.4)
Height, mean (SD), cm	163 (9.4)	163 (9.3)	163 (9.4)
Weight, mean (SD), kg	86.3 (12.7)	86.7 (12.7)	85.8 (12.8)
Waist circumference, mean (SD), cm	107 (9.3)	108 (9.1)	107 (9.4)
Type 2 diabetes	400 (26.3)	210 (27.6)	190 (25.0)
Smoking habits			
Current	195 (12.8)	111 (14.6)	84 (11.0)
Former	684 (45.0)	328 (43.2)	356 (46.8)
Never	642 (42.2)	321 (42.2)	321 (42.2)
Educational level			
University or technician	325 (21.4)	159 (20.9)	166 (21.8)
Secondary school	456 (30.0)	233 (30.7)	223 (29.3)
Primary school or less	740 (48.6)	368 (48.4)	372 (48.9)
Physical activity, mean (SD), METs min/wk	2685 (2313)	2566 (2211)	2803 (2406)
Chair-stand test, mean (SD), No. of repeats	14.7 (4.8)	14.7 (4.7)	14.8 (4.9)
Sedentary behavior, mean (SD), h/d	5.86 (1.86)	5.84 (1.82)	5.89 (1.89)
Total energy intake, mean (SD), kcal/d	2427 (582)	2412 (566)	2443 (599)
Alcohol intake, mean (SD), g/d	11.6 (15.3)	11.4 (15.7)	11.8 (14.9)
Adherence to energy-reduced MedDiet, mean (SD), points	8.35 (2.61)	8.25 (2.58)	8.45 (2.63)
Total fat mass, mean (SD), g	34 307 (7229)	34 423 (7256)	34 191 (7204)
Total fat mass, mean (SD), %	40.5 (6.9)	40.5 (6.9)	40.4 (6.9)
Visceral fat mass, mean (SD), g	2290 (892)	2281 (902)	2299 (883)
Visceral fat mass, mean (SD), %	6.79 (2.5)	6.72 (2.4)	6.86 (2.5)
Total lean mass, mean (SD), g	48 190 (9649)	48 308 (9642)	48 073 (9661)
Total lean mass, mean (SD), %	56.5 (6.6)	56.4 (6.6)	56.6 (6.5)

Abbreviations: BMI, body mass index (calculated as weight in kilograms divided by height in meters squared); DXA, dual energy x-ray absorptiometry; MedDiet, energy-restricted Mediterranean diet; METs, metabolic equivalents.

^a^ Percentages of fat mass and lean mass were calculated in relation to DXA-derived total body mass (sum of total bone, fat, and muscle mass), whereas the percentage of visceral fat was calculated in relation to total fat mass. The prevalence of type 2 diabetes was used as a dichotomous variable, wherein its presence was defined as previous self-reported diagnosis of diabetes, hemoglobin A_1c_ 6.5% or greater (to convert to proportion of total hemoglobin, multiply by 0.01), use of antidiabetic medication, or having fasting glucose levels greater than 126 mg/dL (to convert to millimoles per liter, multiply by 0.0555) in both the screening and baseline visits.

Height (centimeters) was measured without shoes in duplicate using a wall-mounted stadiometer. Population questionnaires validated in Spanish were used to collect data on total leisure-time PA (metabolic equivalent of tasks per minute per week), sedentary behavior (hours per day), total energy intake (kilocalories per day), and alcohol intake (grams per day).^18^

### Statistical Analyses

These analyses were prespecified and performed according to the previously published statistical analysis plan (Supplement 3).^9^ Required sample size was calculated only for the primary outcomes of the trial (for the combined cardiovascular end point the required sample size was 3000 in each group and for weight loss 337 in each group)^8,9^ but not for intermediate outcomes. However, under analogous assumptions than those a priori adopted for weight change, the statistical power will be sufficiently high for assessing changes in body composition with a total sample size of 1521. Main analyses were performed in the evaluable population (completers only) and in sensitivity analyses, multiple imputation was performed to include data of participants lost to follow-up (intention-to-treat analyses).

Linear mixed-effects models were used to assess between-group differences in changes in body components at baseline, 1 year, and 3 years of follow-up. Three-level linear mixed models were fitted with random intercepts at the recruiting center, cluster family, and individual participants, including an interaction term of study arm with time. Multivariable models were adjusted for sex, age, and baseline levels of smoking status, educational level, T2D, height, total leisure-time PA, sedentary behavior, total energy intake, and alcohol intake. To enable a direct comparison between body composition parameters, models were rerun with all outcome variables converted into *z* scores (mean [SD], 0 [1]).

In sensitivity analyses, the missing follow-up data on primary and secondary outcomes were handled using multivariate imputation with chained equations (Stata mi command), creating 100 imputations for each missing measurement. The imputation models included as factors age, sex, study arm, weight (waist when imputing visceral fat and its indicators), height, smoking habit, educational level, leisure-time PA, sedentary behavior, total energy intake, alcohol intake, T2D, recruiting center, and the baseline value of the imputed variable as factors.

We also evaluated the potential effect of the PREDIMED-Plus intervention on dichotomous variables indicating at least 5% improvement in baseline body composition as an indicator of clinically relevant changes over follow-up. Given that the targets for clinically relevant changes in body composition are still unclear,^19^ the threshold of 5% was chosen as it is a cutoff point customarily used for weight-loss strategies targeting beneficial cardiometabolic changes.^20,21^ We used logistic regression analysis with a robust variance estimator after adjusting for the same set of covariates as in the main analyses. Logistic regression models for marginal effects estimating absolute risk differences^22,23^ and the numbers needed to treat^24,25^ were applied to estimate the intervention effect magnitude.

Post hoc stratified analyses were conducted for primary outcomes by baseline categories of sex (men and women), age (<65 and ≥65 years), T2D prevalence (yes and no), and smoking habits (current or former smoker, or never smoker) using linear mixed-effects multivariable models. An interaction term between time, randomization arm of the trial, and each potential effect modifier was included in the models. We set the *P* value at .002 (0.05/24) after applying a Bonferroni correction to account for multiple testing in this subgroup analysis.

Statistical analyses were performed using Stata, version 17.0 (StataCorp LLC). All statistical tests were 2-sided, and the threshold of significance was set at .05, unless stated otherwise.

## Results

### Characteristics of Study Participants

Participants with DXA scans (mean [SD] age, 65.3 [5.0] years; 52.1% men, 47.9% women) assigned to the intervention and control groups showed similar characteristics at baseline (Table 1). The subsample of participants with DXA scans at baseline (n = 1556) was similar to the rest of participants enrolled in the PREDIMED-Plus trial (n = 5318) in terms of sociodemographic, health-related, and lifestyle factors. Relative differences between included and nonincluded individuals were less than 10%, except for self-reported PA, with a relative difference of only 12%. The size of the between-groups difference for chair-stand test, an objective measure of physical fitness, was even lower, at less than 10% (eTable 2 in Supplement 2).

### Body Composition Changes by Study Group Over Follow-Up

Overall, significant between-group differences were observed across follow-up for all primary outcomes (Table 2). Participants in the intervention group showed significant reductions in the percentage of total fat mass expressed as within-group mean changes and 95% CIs (model 2: 1-year vs baseline, −1.14%; 95% CI, −1.32% to −0.96%; 3-year vs baseline, −0.52%; 95% CI, −0.71% to −0.33%), and grams of visceral fat (1-year vs baseline, −154 g; 95% CI, −191 to −116 g; 3-year vs baseline, −75.1 g, 95% CI, −115 to −35.3 g), as well as significant within-group increases in percentage of total lean mass (1-year vs baseline, 1.07%; 95% CI, 0.90%-1.25%; 3-year vs baseline, 0.47%; 95% CI, 0.29%-0.65%) over follow-up time, although the magnitude of change was larger at year 1. Changes within the control group were almost negligible, resulting in statistically significant between-group differences in changes over follow-up for all outcomes. Adjusted between-group differences of means (model 1) of body composition parameters (primary outcomes) in the overall sample can be seen in the Figure and those by sex can be seen in eFigure 2 in Supplement 2. In general, changes in body composition parameters showed similar patterns in both sexes, although these were more pronounced in men than in women.

**Table 2.** Effect of the PREDIMED-Plus Intervention on Body Composition Changes Over Follow-Up Time in Participants Who Completed Follow-Up^a^

Variable	Model 1 (adjusted)				Model 2 (unadjusted)			
	Mean change (95% CI)		Between-group-difference		Mean change (95% CI)		Between-group difference	
	Changes in intervention (n = 760)	Changes in control (n = 761)	Mean difference in changes (95% CI)	*P* value^b^	Changes in intervention (n = 760)	Changes in control (n = 761)	Mean difference in changes (95% CI)	*P* value^b^
**Primary outcomes**								
Total fat mass, %								
Year 1 vs baseline	−1.14 (−1.33 to −0.96)	−0.21 (−0.39 to −0.03)	−0.93 (−1.19 to −0.68)	<.001	−1.14 (−1.32 to −0.96)	−0.20 (−0.38 to −0.02)	−0.94 (−1.19 to −0.69)	<.001
Year 3 vs baseline	−0.52 (−0.71 to −0.33)	−0.14 (−0.33 to 0.04)	−0.38 (−0.64 to −0.12)		−0.52 (−0.71 to −0.33)	−0.14 (−0.32 to 0.04)	−0.38 (−0.64 to −0.12)	
Total lean mass, %								
Year 1 vs baseline	1.08 (0.90 to 1.25)	0.20 (0.03 to 0.38)	0.87 (0.63 to 1.12)	<.001	1.07 (0.90 to 1.25)	0.20 (0.03 to 0.37)	0.88 (0.63 to 1.12)	<.001
Year 3 vs baseline	0.47 (0.29 to 0.66)	0.13 (−0.04 to 0.31)	0.34 (0.09 to 0.59)		0.47 (0.29 to 0.65)	0.13 (−0.05 to 0.30)	0.34 (0.09 to 0.60)	
Visceral fat mass, g								
Year 1 vs baseline	−150 (−188 to −112)	−26.9 (−64.1 to 10.4)	−123 (−177 to −70.2)	<.001	−154 (−191 to −116)	−27.2 (−64.4 to 9.95)	−126 (−179 to −73.3)	<.001
Year 3 vs baseline	−72.2 (−112 to −32.3)	−5.50 (−43.9 to 32.9)	−66.7 (−122 to −11.3)		−75.1 (−115 to −35.3)	−4.68 (−43.0 to 33.6)	−70.4 (−126 to −15.2)	
**Secondary outcomes**								
Total fat mass, g								
Year 1 vs baseline	−1683 (−1936 to −1430)	−296 (−544 to −48.0)	−1387 (−1742 to −1033)	<.001	−1677 (−1930 to −1424)	−284 (−532 to −36.3)	−1393 (−1747 to −1039)	<.001
Year 3 vs baseline	−1022 (−1284 to −759)	−277 (−530 to −24.5)	−744 (−1109 to −380)		−1018 (−1280 to −756)	−260 (−512 to −7.25)	−758 (−1122 to −393.9)	
Total lean mass, g								
Year 1 vs baseline	−294 (−433 to −155)	−52.4 (−188 to 83.6)	−241 (−436 to −46.7)	.001	−300 (−439 to −162)	−48.6 (−184 to 87.0)	−252 (−446 to −57.7)	.001
Year 3 vs baseline	−625 (−769 to −481)	−269 (−408 to −131)	−356 (−556 to −156)		−626 (−770 to −483)	−260 (−398 to −122)	−366 (−565 to −167)	
Visceral fat mass, %								
Year 1 vs baseline	−0.14 (−0.23 to −0.04)	−0.05 (−0.14 to 0.04)	−0.09 (−0.22 to 0.05)	.44	−0.15 (−0.24 to −0.05)	−0.05 (−0.15 to 0.04)	−0.10 (−0.23 to 0.04)	.37
Year 3 vs baseline	−0.03 (−0.13 to 0.07)	0.01 (−0.09 to 0.10)	−0.03 (−0.17 to 0.10)		−0.04 (−0.14 to 0.06)	0.01 (−0.09 to 0.10)	−0.04 (−0.18 to 0.09)	
Android to gynoid fat mass ratio								
Year 1 vs baseline	−0.02 (−0.02 to −0.01)	−0.01 (−0.01 to 0.00)	−0.01 (−0.02 to 0.00)	.12	−0.02 (−0.03 to −0.01)	−0.01 (−0.02 to 0.00)	−0.01 (−0.02 to 0.00)	.10
Year 3 vs baseline	−0.02 (−0.02 to −0.01)	0.00 (−0.01 to 0.00)	−0.01 (−0.02 to 0.00)		−0.02 (−0.03 to −0.01)	−0.00 (−0.01 to 0.00)	−0.01 (−0.02 to 0.00)	
Total lean mass to total fat mass ratio								
Year 1 vs baseline	0.09 (0.07 to 0.10)	0.01 (0.00 to 0.03)	0.07 (0.05 to 0.09)	<.001	0.09 (0.07 to 0.10)	0.01 (−0.00 to 0.03)	0.07 (0.05 to 0.09)	<.001
Year 3 vs baseline	0.04 (0.02 to 0.05)	0.01 (0.00 to 0.03)	0.03 (0.01 to 0.05)		0.04 (0.02 to 0.05)	0.01 (−0.00 to 0.03)	0.03 (0.01 to 0.05)	

Abbreviation: PREDIMED-Plus, Prevención con Dieta Mediterránea-Plus.

^a^ Percentages of fat mass and lean mass were calculated in relation to dual energy x-ray absorptiometry–derived total body mass (sum of total bone, fat, and muscle mass), whereas percentage of visceral fat was calculated in relation to total fat mass. Three-level linear mixed models with random intercepts at recruiting center, cluster family, and individual participant were used to assess intervention group effects on changes in body composition parameters measured repeatedly over time (at each follow-up visit and for the overall follow-up period). Potential interactions of study arm with time were tested in these models: model 1: without additional adjustments; model 2: model 1 plus sex (dichotomous), age (continuous), and baseline levels of smoking status (3 categories), educational level (3 categories), type 2 diabetes prevalence (dichotomous), height, total leisure-time physical activity, sedentary behavior, total energy intake and alcohol intake (continuous). The number of participants at baseline was 1521 (n=760 intervention group, n=761 control group); at 1 year n=1215 (n=595 for intervention group, n=620) for all body components, except for visceral fat n=1208 (n=592 for intervention group, n=616); at 3 years n=1133 (n=543 for intervention group, n=590) for all body components, except for visceral fat n=1091 (n=522 for intervention group, n=569).

^b^ *P* value represents the intervention group effects assessed for the overall follow-up period.

**Figure.**
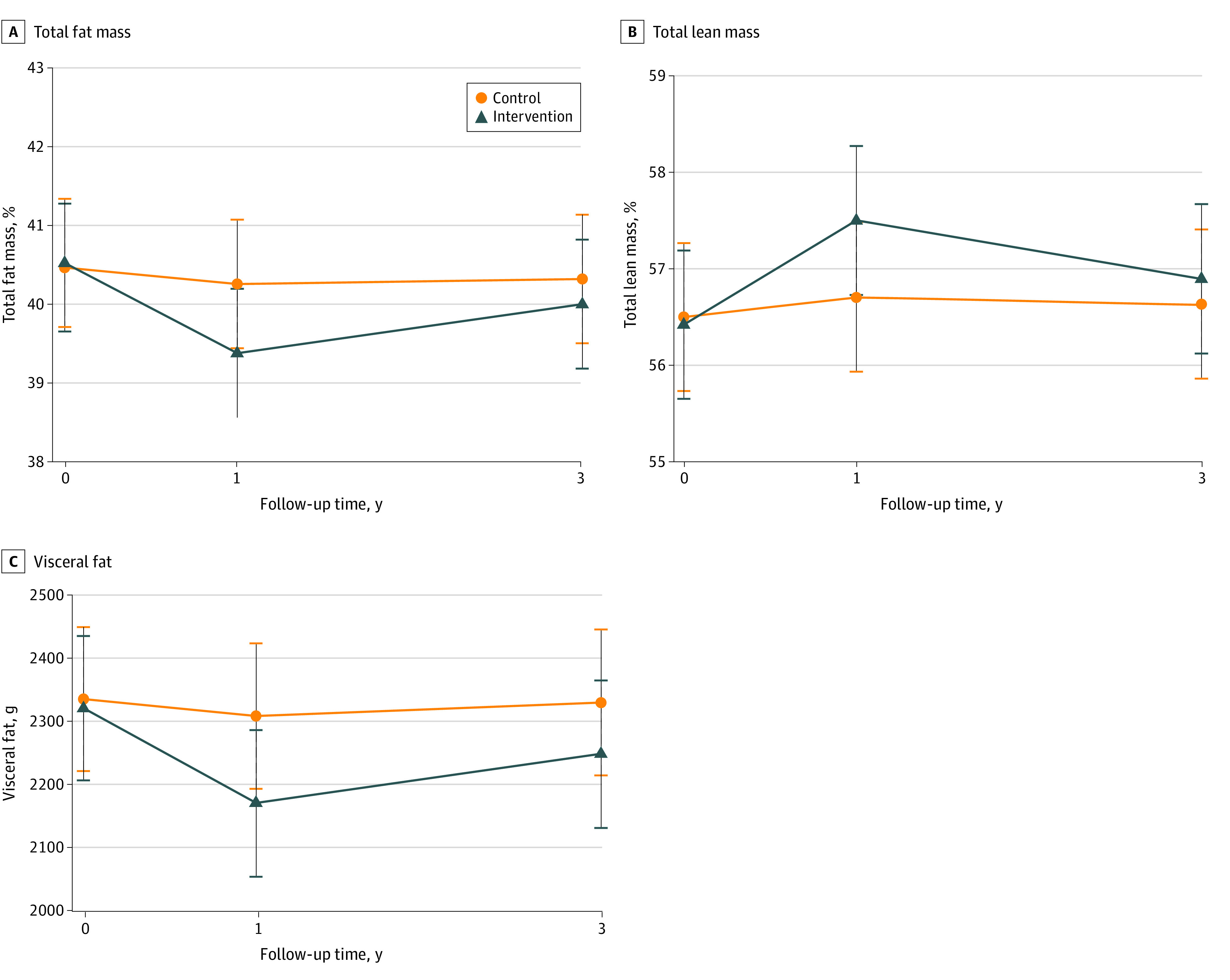
Adjusted Means of Body Composition Parameters Through Follow-Up by Study Arm The primary trial outcomes of fat mass (A), lean mass (B), and visceral mass (C) were calculated in participants who completed the study. Percentages of fat mass and lean mass were calculated in relation to dual energy x-ray absorptiometry–derived total body mass (sum of total bone, fat, and muscle mass). Means were minimally adjusted for center, cluster family, and individualized participant. Error bars indicate 95% CIs.

When looking at body mass components in absolute amounts (total fat and total lean mass in grams, included as secondary outcomes in Table 2), participants in the intervention group lost mostly total fat (model 2: mean change at 1 year vs baseline, −1677 g; 95% CI, −1930 to −1424 g) and, to a lesser extent, lean mass (−300 g; 95% CI, −439 to −162 g) during the first year of the trial, and then partially regained fat mass (mean change at 3 years vs baseline, −1018 g; 95% CI, −1280 to −756 g) and lost lean mass (−626 g; 95% CI, −770 to −483 g) thereafter, albeit at a slower rate. Changes observed in total fat mass and lean mass among participants in the control group were of lower magnitude; hence, between-group changes were significant for both fat and lean mass. Despite the loss of lean mass in the intervention group as a result of the weight-loss process, the ratio of lean mass to fat mass increased over follow-up in the intervention group and was unchanged in the control group (significant between-group changes). When looking at other indicators of fat distribution, such as the percentage of visceral fat in relation to total fat or the android to gynoid fat ratio, no between-group differences in changes were observed over follow-up, despite small favorable changes in the intervention group (Table 2). Similar trajectories in these secondary outcomes were observed in men and women (eFigure 3 in Supplement 2).

The same analyses conducted with body composition parameters converted into *z* scores (eTable 3 in Supplement 2) showed that between-group differences were of the same magnitude but in opposite directions for percentages of total fat and lean mass, and the magnitude of change was much greater for total fat than for total lean mass, when expressed in grams. The size of the effect of the intervention vs control group on grams of total and visceral fat masses was comparable. Analyses using multiple imputation showed similar results (eTable 4 in Supplement 2).

### Clinically Meaningful Improvements in Body Composition

Compared with the controls, participants in the intervention group were more likely to show improvements of 5% or more from baseline values in all body components at 1 and 3 years of follow-up, although these improvements were more evident during the first year (absolute risk reduction after 1 year, 13% for total fat mass, 11% for total lean mass, and 14% for visceral fat mass; after 3 years, 6% for total fat mass, 6% for total lean mass and 8% for visceral fat mass) (Table 3). For every 12 persons receiving the intensive lifestyle intervention, as compared with the control arm, 1 additional individual will clinically improve their visceral fat mass at year 3. For percentage fat and percentage lean mass, the number needed to treat was 17.

**Table 3.** Effect of the PREDIMED-Plus Intervention on at Least 5% Improvements of Baseline Values in Body Composition Components Over Follow-Up Time in Participants Who Completed Follow-Up: Adjusted Risk Parameters From Logistic Regression Models for Marginal Effects^a^

Variable	Improvements in intervention group		Improvements in control group		Intervention vs control (95% CI)		
	No./total No. (%)	Proportion with improvement (95% CI)	No./total No. (%)	Proportion with improvement (95% CI)	Improvement ratio	Absolute difference	No. needed to treat^b^
**Total fat mass, %**							
Year 1 vs baseline	168/595 (28.2)	0.28 (0.24-0.31)	89/620 (14.4)	0.15 (0.12-0.17)	1.91 (1.52-2.40)	0.13 (0.09-0.18)	7.7 (5.6-11.1)
Year 3 vs baseline	126/543 (23.2)	0.23 (0.20-0.27)	100/590 (17.0)	0.17 (0.14-0.20)	1.37 (1.09-1.73)	0.06 (0.02-0.11)	16.7 (9.1-50.0)
**Total lean mass, %**							
Year 1 vs baseline	113/595 (19.0)	0.19 (0.16-0.22)	80/620 (8.01)	0.08 (0.06-0.10)	2.30 (1.69-3.14)	0.11 (0.07-0.14)	9.1 (7.1-14.3)
Year 3 vs baseline	87/543 (16.0)	0.16 (0.13-0.19)	58/590 (9.8)	0.10 (0.07-0.12)	1.63 (1.21-2.21)	0.06 (0.02-0.10)	16.7 (10.0-50.0)
**Visceral fat mass, g**							
Year 1 vs baseline	312/592 (52.7)	0.53 (0.49-0.57)	237/616 (38.5)	0.39 (0.35-0.42)	1.37 (1.21-1.55)	0.14 (0.09-0.20)	7.1 (5.0-11.1)
Year 3 vs baseline	236/522 (45.2)	0.45 (0.41- 0.49)	214/569 (37.6)	0.38 (0.34-0.42)	1.20 (1.05-1.39)	0.08 (0.02-0.13)	12.5 (7.7-50.0)

Abbreviation: PREDIMED-Plus, Prevención con Dieta Mediterránea-Plus.

^a^ Between-group differences in change from baseline to 1 and 3 years were assessed using logistic regression models for marginal effects with robust variance estimator after adjusting for sex (dichotomous), age (continuous), and baseline levels of smoking status (3 categories), educational level (3 categories), type 2 diabetes prevalence (dichotomous), height, total leisure-time physical activity, sedentary behavior, total energy intake and alcohol intake (continuous). Postestimation tests were applied (Stata adjrr command) to estimate adjusted risk parameters. Percentages of fat mass and lean mass were calculated in relation to dual energy x-ray absorptiometry–derived total body mass (sum of total bone, fat, and muscle mass). Calculation of 5% improvements in body composition: for each outcome at 1 and 3 years, dummy variables were created, where 0 indicates lack of improvements in body composition parameters from baseline (ie, <5% reduction from baseline in percentage of total fat mass and grams of visceral fat mass, and <5% increase in percentage of total lean mass) and 1 indicates that parameters are equal or improved from baseline (ie, ≥5% reduction from baseline in percentage of total fat mass and grams of visceral fat mass, and ≥5% increase in percentage of total lean mass).

^b^ Number needed to treat: 1/risk difference. 95% CI: (maximum number needed to treat; minimum number needed to treat) = (1/risk difference minimum 95% CI, 1/risk difference maximum 95% CI).

### Effect Modification Analyses

In the post hoc analyses, no interactions with sex and smoking habits were found for any of the outcomes studied over follow-up time, but significant interactions with age were observed (eFigure 4 in Supplement 2). Younger participants (age <65 years) showed larger beneficial changes in body composition parameters at year 1, but those changes were not sustained at year 3. The magnitude of change was lower in older participants (age ≥65 years) but more stable over time. Moreover, for all these body components, between-group differences were greater and statistically significant only in participants without T2D.

## Discussion

Results from this interim analysis of an ongoing primary prevention RCT indicate that a 3-year weight-loss intervention based on an energy-reduced MedDiet and PA, compared with advice to follow the MedDiet without weight-loss goals, resulted in significant improvements in body composition in middle-aged and older adults with chronic health conditions. In particular, we found that this multifactorial lifestyle intervention was effective in reducing total body fat and visceral fat. In addition, the lifestyle intervention led to an attenuation of age-related decreases in lean mass.

In the present study, participants from both groups lost total fat mass in absolute amount, and in parallel, total fat mass relative to total body mass. The decrease in fat mass was small and relatively stable over the follow-up period in the control group. In contrast, in the intervention group, fat mass decreased appreciably, particularly during the first year of the trial, and it was partially regained in the period up to the third year of the trial. Participants in the intervention group showed greater reductions in fat mass (both in grams and percentage of body mass) than those in the control group over the entire follow-up time.

We found that only participants from the intervention group decreased grams of visceral fat mass, while in the control group the mass of this fat depot was unchanged over time. The reduction observed in the intervention group did not seem to be independent from changes in total fat mass, as there was no effect of the PREDIMED-Plus intervention on percentage of visceral fat in relation to total fat mass nor on the ratio android to gynoid fat mass. This could indicate that greater loss of total fat mass over time is necessary to mobilize visceral fat mass storage in older adults with overweight or obesity and metabolic syndrome. Even though visceral fat constitutes a small portion of total body fat, the size of the effects of the PREDIMED-Plus intervention on total and visceral fat mass was comparable, as observed in analyses using normalized into *z* scores body components (eTable 3 in Supplement 2).

Besides fat mass, intentional weight loss is usually associated with loss of lean mass, similar to the process of aging. In this regard, we observed that both the control and intervention groups showed significant loss of lean mass in the absolute amount between baseline and 3-year follow-up time, with the effect being greater for the participants in the intervention group. In comparison with the controls, participants in the intervention group showed increases in the percentage of total lean mass in relation to the total body mass (lean + fat + bone mass) and the total lean mass to total fat mass ratio. This finding is noteworthy as it represents a more favorable body composition profile sustained during a 3-year period, suggesting that participants in the intervention group achieved weight loss preferentially at the expense of total fat rather than lean mass. Lifestyle interventions for weight loss should include strategies preserving lean body mass to delay age-dependent loss of muscle mass or sarcopenia (loss of muscle mass and strength),^26^ but also to minimize the reduction of energy expenditure associated with the loss of lean mass and therefore a potentially increased risk of weight regain at midterm.^27^

Despite the fact that the magnitude of body composition changes was rather mild, although statistically significant, we found that these changes were likely of clinical relevance (based on attainment of at least 5% improvements in baseline values). In particular, we found that for every 12 or 17 persons receiving an intervention with energy-reduced MedDiet and PA, as opposed to advice to follow ad libitum MedDiet, 1 additional individual would improve visceral fat mass (n = 12) or percentage of fat and lean mass (n = 17). In the same direction, previous findings from the pilot study of the PREDIMED-Plus trial^10^ and other RCTs^28,29^ have evidence that total or visceral fat reductions (independent of the type of intervention) were paralleled by beneficial changes in most classic cardiometabolic risk factors. Undoubtedly, continued follow-up is warranted to confirm whether these moderate improvements may be effective in preventing cardiovascular events or mortality.

### Strengths and Limitations

In addition to the multicenter clinical trial design, the relatively large sample size and follow-up period, the strengths of our study include use of repeatedly measured data and determination of body composition with precise imaging techniques with standardized measurements. The trial also has limitations. First, our findings are based on interim analysis of the intermediate outcomes of an ongoing RCT. Second, the generalizability of these results to other populations (younger and/or healthier) may be limited. Furthermore, measures of body composition were not available in the total cohort, due to limited access to DXA scanners in most of the recruiting centers. Lastly, there were losses to follow-up in DXA participation leading to missing data in body composition variables; however, the results were similar when multiple imputation was conducted.

## Conclusions

In this interim subgroup analysis of body composition in the PREDIMED-Plus RCT, we found evidence that a 3-year weight-loss intervention with energy-reduced MedDiet and PA may mitigate age-dependent changes in body composition by reducing total and visceral fat and delaying loss of lean mass in middle-aged and older adults with overweight or obesity and metabolic syndrome. These changes are likely of public health and clinical relevance. Given the metabolic relevance of specific body components, especially visceral fat and lean mass, the benefits of this lifestyle intervention could be very promising. However, continued follow-up is warranted to confirm the long-term consequences of these changes on health.

## Notes

Supplement 1.
Trial Protocol

Supplement 2. **eTable 1.** Number of Study Participants With and Without Data on Body Composition From Centers Having Access to DXA Device **eTable 2.** Comparison of Principal Baseline Characteristics of Study Participants With Data on Body Composition Measured With DXA and Participants Without DXA Data From the Total Cohort **eFigure 1.** Flow Chart for the Selection of Participants for Analysis **eMethods.** Details on PREDIMED-Plus Intervention and Body Composition Measurement With DXA
eReferences
**eFigure 2.** Adjusted Means (95% CIs) of Body Composition Parameters (Primary Outcomes) Through Follow-Up by Study Arm Separately in Men and Women (Completers-Only) **eFigure 3.** Adjusted Means (95% CIs) of Body Composition Parameters (Secondary Outcomes) Through Follow-Up by Study Arm in Overall Sample and Separately in Men and Women **eTable 3.** Effect of the PREDIMED-Plus Intervention on Body Composition Changes (z-Scores of Primary and Secondary Outcomes) Over Follow-Up Time (Completers-Only) **eTable 4.** Effect of the PREDIMED-Plus Intervention on Body Composition Changes (Primary and Secondary Outcomes) Over Follow-Up Time (Multiple Imputations, n = 100) **eFigure 4.** Effect of the PREDIMED-Plus Intervention on Body Composition Changes Over Follow-Up Time by Subgroups (Completers-Only)

Supplement 3.
Statistical Analysis Plan

Supplement 4.
Data Sharing Statement

## References

[1] Britton KA, Massaro JM, Murabito JM, Kreger BE, Hoffmann U, Fox CS. Body fat distribution, incident cardiovascular disease, cancer, and all-cause mortality. J Am Coll Cardiol. 2013;62(10):921-925. doi:10.1016/j.jacc.2013.06.027 23850922 PMC4142485

[2] Knowles R, Carter J, Jebb SA, Bennett D, Lewington S, Piernas C. Associations of skeletal muscle mass and fat mass with incident cardiovascular disease and all-cause mortality: a prospective cohort study of UK Biobank participants. J Am Heart Assoc. 2021;10(9):e019337. doi:10.1161/JAHA.120.019337 33870707 PMC8200765

[3] Mancini JG, Filion KB, Atallah R, Eisenberg MJ. Systematic review of the Mediterranean diet for long-term weight loss. Am J Med. 2016;129(4):407-415.e4. doi:10.1016/j.amjmed.2015.11.028 26721635

[4] Estruch R, Martínez-González MA, Corella D, ; PREDIMED Study Investigators. Effect of a high-fat Mediterranean diet on bodyweight and waist circumference: a prespecified secondary outcomes analysis of the PREDIMED randomised controlled trial. Lancet Diabetes Endocrinol. 2019;7(5):e6-e17. doi:10.1016/S2213-8587(19)30074-9 31003626

[5] Bendall CL, Mayr HL, Opie RS, Bes-Rastrollo M, Itsiopoulos C, Thomas CJ. Central obesity and the Mediterranean diet: a systematic review of intervention trials. Crit Rev Food Sci Nutr. 2018;58(18):3070-3084. doi:10.1080/10408398.2017.1351917 29039967

[6] Vissers D, Hens W, Taeymans J, Baeyens J-P, Poortmans J, Van Gaal L. The effect of exercise on visceral adipose tissue in overweight adults: a systematic review and meta-analysis. PLoS One. 2013;8(2):e56415. doi:10.1371/journal.pone.0056415 23409182 PMC3568069

[7] Beaudart C, Dawson A, Shaw SC, ; IOF-ESCEO Sarcopenia Working Group. Nutrition and physical activity in the prevention and treatment of sarcopenia: systematic review. Osteoporos Int. 2017;28(6):1817-1833. doi:10.1007/s00198-017-3980-9 28251287 PMC5457808

[8] Martínez-González MA, Buil-Cosiales P, Corella D, ; PREDIMED-Plus Study Investigators. Cohort profile: design and methods of the PREDIMED-Plus randomized trial. Int J Epidemiol. 2019;48(2):387-388o. doi:10.1093/ije/dyy225 30476123

[9] Sayón-Orea C, Razquin C, Bulló M, . Effect of a nutritional and behavioral intervention on energy-reduced Mediterranean diet adherence among patients with metabolic syndrome: interim analysis of the PREDIMED-Plus randomized clinical trial. JAMA. 2019;322(15):1486-1499. doi:10.1001/jama.2019.14630 31613346 PMC6802271

[10] Salas-Salvadó J, Díaz-López A, Ruiz-Canela M, ; PREDIMED-Plus investigators. Effect of a lifestyle intervention program with energy-restricted Mediterranean diet and exercise on weight loss and cardiovascular risk factors: one-year results of the PREDIMED-Plus trial. Diabetes Care. 2019;42(5):777-788. doi:10.2337/dc18-0836 30389673

[11] Schulz KF, Altman DG, Moher D; CONSORT Group. CONSORT 2010 statement: updated guidelines for reporting parallel group randomised trials. BMJ. 2010;340:c332. doi:10.1136/bmj.c332 20332509 PMC2844940

[12] Alberti KG, Eckel RH, Grundy SM, ; International Diabetes Federation Task Force on Epidemiology and Prevention; National Heart, Lung, and Blood Institute; American Heart Association; World Heart Federation; International Atherosclerosis Society; International Association for the Study of Obesity. Harmonizing the metabolic syndrome: a joint interim statement of the International Diabetes Federation Task Force on Epidemiology and Prevention; National Heart, Lung, and Blood Institute; American Heart Association; World Heart Federation; International Atherosclerosis Society; and International Association for the Study of Obesity. Circulation. 2009;120(16):1640-1645. doi:10.1161/CIRCULATIONAHA.109.192644 19805654

[13] Schröder H, Cárdenas-Fuentes G, Martínez-González MA, ; PREDIMED-Plus investigators. Effectiveness of the physical activity intervention program in the PREDIMED-Plus study: a randomized controlled trial. Int J Behav Nutr Phys Act. 2018;15(1):110. doi:10.1186/s12966-018-0741-x 30424822 PMC6234632

[14] Schröder H, Zomeño MD, Martínez-González MA, ; PREDIMED-Plus investigators. Validity of the energy-restricted Mediterranean diet adherence screener. Clin Nutr. 2021;40(8):4971-4979. doi:10.1016/j.clnu.2021.06.030 34364236

[15] Schröder H, Fitó M, Estruch R, . A short screener is valid for assessing Mediterranean diet adherence among older Spanish men and women. J Nutr. 2011;141(6):1140-1145. doi:10.3945/jn.110.135566 21508208

[16] Martínez-González MA, García-Arellano A, Toledo E, ; PREDIMED Study Investigators. A 14-item Mediterranean diet assessment tool and obesity indexes among high-risk subjects: the PREDIMED trial. PLoS One. 2012;7(8):e43134. doi:10.1371/journal.pone.0043134 22905215 PMC3419206

[17] Kaul S, Rothney MP, Peters DM, . Dual-energy x-ray absorptiometry for quantification of visceral fat. Obesity (Silver Spring). 2012;20(6):1313-1318. doi:10.1038/oby.2011.393 22282048 PMC3361068

[18] Fernández-Ballart JD, Piñol JL, Zazpe I, . Relative validity of a semi-quantitative food-frequency questionnaire in an elderly Mediterranean population of Spain. Br J Nutr. 2010;103(12):1808-1816. doi:10.1017/S0007114509993837 20102675

[19] Swainson MG, Batterham AM, Hind K. Age- and sex-specific reference intervals for visceral fat mass in adults. Int J Obes (Lond). 2020;44(2):289-296. doi:10.1038/s41366-019-0393-1 31201361

[20] Pi-Sunyer X, Blackburn G, Brancati FL, ; Look AHEAD Research Group. Reduction in weight and cardiovascular disease risk factors in individuals with type 2 diabetes: one-year results of the look AHEAD trial. Diabetes Care. 2007;30(6):1374-1383. doi:10.2337/dc07-0048 17363746 PMC2665929

[21] Zomer E, Gurusamy K, Leach R, . Interventions that cause weight loss and the impact on cardiovascular risk factors: a systematic review and meta-analysis. Obes Rev. 2016;17(10):1001-1011. doi:10.1111/obr.12433 27324830

[22] Norton EC, Miller MM, Kleinman LC. Computing adjusted risk ratios and risk differences in Stata. Stata J. 2013;13(3):492-509. doi:10.1177/1536867X1301300304

[23] Norton EC, Dowd BE, Maciejewski ML. Marginal effects—quantifying the effect of changes in risk factors in logistic regression models. JAMA. 2019;321(13):1304-1305. doi:10.1001/jama.2019.1954 30848814

[24] Saver JL, Lewis RJ. Number needed to treat: conveying the likelihood of a therapeutic effect. JAMA. 2019;321(8):798-799. doi:10.1001/jama.2018.21971 30730545

[25] Altman DG. Confidence intervals for the number needed to treat. BMJ. 1998;317(7168):1309-1312. doi:10.1136/bmj.317.7168.1309 9804726 PMC1114210

[26] Cruz-Jentoft AJ, Bahat G, Bauer J, ; Writing Group for the European Working Group on Sarcopenia in Older People 2 (EWGSOP2), and the Extended Group for EWGSOP2. Sarcopenia: revised European consensus on definition and diagnosis. Age Ageing. 2019;48(1):16-31. doi:10.1093/ageing/afy169 30312372 PMC6322506

[27] Martins C, Gower BA, Hunter GR. Association between fat-free mass loss after diet and exercise interventions and weight regain in women with overweight. Med Sci Sports Exerc. 2022;54(12):2031-2036. doi:10.1249/MSS.0000000000002992 35797356 PMC9669159

[28] Gepner Y, Shelef I, Schwarzfuchs D, . Effect of distinct lifestyle interventions on mobilization of fat storage pools CENTRAL magnetic resonance imaging randomized controlled trial. Circulation. 2018;137(11):1143-1157. doi:10.1161/CIRCULATIONAHA.117.030501 29142011

[29] Zelicha H, Kloting N, Kaplan A, . The effect of high-polyphenol Mediterranean diet on visceral adiposity: the DIRECT PLUS randomized controlled trial. BMC Med. 2022;20(1):327. doi:10.1186/s12916-022-02525-8 36175997 PMC9523931

