# Supplementary material for: An Energy-Reduced Mediterranean Diet, Physical Activity, and Body Composition: An Interim Subgroup Analysis of the PREDIMED-Plus Randomized Clinical Trial
Source: JAMA Netw Open. 2023 Oct 18;6(10):e2337994. doi: 10.1001/jamanetworkopen.2023.37994 (PMC10585413; doi:10.1001/jamanetworkopen.2023.37994)
Supplement: Supplement 1. — Trial Protocol [file jamanetwopen-e2337994-s001.pdf]

EFFECT OF AN ENERGY-RESTRICTED MEDITERRANEAN DIET, PHYSICAL ACTIVITY AND BEHAVIORAL TREATMENT ON THE PRIMARY PREVENTION OF CARDIOVASCULAR DISEASE

THE PREDIMED-PLUS TRIAL

RESEARCH PLAN

January 2014

Table of contents

	Page
**Amendments to the protocol after January 2014**	**3**
**2014 PROTOCOL**	
**Abstract**	**4**
**Background**	**6**
**Hypothesis**	**11**
**Objectives**	**11**
**Methodology**	**13**
**Statistical Analysis Plan**	**50**
**Strengths and limitations**	**53**
**Committees and governance**	**54**
**Sources of funding and administrative issues**	**55**
**References**	**57**
**Annex I (substudies)**	**71**
**Annex II (participating centers)**	**72**

AMENDMENTS TO THE PROTOCOL AFTER JANUARY 2014

1. In January 2014 the Steering Committee following the advice of the Data Safety and Monitoring Board decided to amend the Protocol and omit the 1.5 kg weight loss criterion to be achieved during the run-in period. Such change only affected the first 70 participants who were eligible and randomized in 2 vanguard centers.
2. September-July 2014. Initially, we decided to provide 500 g per month at no cost of nuts to each participant during the intervention in order to reinforce adherence to the Mediterranean diet in both arms of the trial. However, due to lack of economic resources, only participants included in the pilot study recruited in 2 vanguard centers up to July 2014 received 250 g of free nuts (125g of pistachios and 125g of almonds per month) along with the recommendation to consume a total monthly amount of 500g. Subsequently, all trial participants received during the follow-up 125g of almonds per month out of the total recommended amount of 500g.
3. Four coordinated grants to fund the trial were received from the Instituto de Salud Carlos III (Madrid) for the periods 2014-2016 and 2017-2019 (Coordinator J. Salas-Salvado) and for the periods 2015-2017 and 2018-2020 (Coordinator J. Vidal).
4. November 2014. In order to increase the possibility of meeting target numbers of recruitees by December 2017, in November 2014 the Steering Committee accepted the inclusion of three additional recruiting centers (see below).

**21**	Jesús Vioque López	vioque@umh.es	Universidad Miguel Hernández, Sant Joan d'Alacant, Alicante
**22**	Miguel Delgado Rodríguez	mdelgado@ujaen.es	Universidad de Jaén, Jaén
**23**	Vicente Martin	vicente.martin@unileon.es	Universidad de León, León

5. December 2016. Dr. Francisco Tinahones (Hospital de Málaga) was incorporated as the seventh member of the Steering Committee.
6. February 2018. Dr. Julia Wärnberg (jwarnberg@uma.es, School of Health Sciences) replaced the Principal Investigator of the recruitment center at Malaga University (Preventive Medicine, Medical School), Prof. Enrique Gómez-Gracia, who continued in the trial as Principal Investigator of a new Support Group (A8).

**A8**	Enrique Gómez-Gracia	egomezgracia@gmail.com	Preventive Medicine, Medical School, University of Malaga

ABSTRACT

In the 21st century we are witnessing an **alarming increase in overweight and obesity**. In **Spain**, **over 60%** of adults are **overweight or obese** and the prevalence of adult **abdominal obesity exceeds 35%** (Gutiérrez-Fisac et al., 2012). The increase in morbid obesity is especially worrying (Basterra-Gortari et al., 2011) because the medium-to-long-term consequences for the **risk of cardiovascular** disease (CVD) and other causes of death or illness can be **catastrophic**.

Observational studies have shown that all-cause mortality increases progressively in parallel with adiposity and that the risk of **cardiovascular mortality** is especially high (Berrington de González et al., 2010). However, a meta-analysis published in early 2013 (Flegal, 2013) raised considerable controversy by much downgrading the consequences of overweight and moderate obesity. The results of this meta-analysis could be explained by the existence of biases, such as unusual definitions for categories of overweight, tobacco as a confounding factor, inverse causality, and the exclusion of relevant studies (Tobias and Hu, 2013). Nevertheless, the controversy persists and will probably do so well into the next few decades. Doubts such as these will only be solved by randomized intervention trials (Hernán and Taubman, 2008). Thus, clinical studies are needed to determine whether **intentional weight loss** reduces cardiovascular mortality and CVD incidence.

In the PREDIMED-PLUS trial we will evaluate the safety and effectiveness of a multifaceted intervention program for alleviating excessive cardiovascular morbidity and mortality in overweight and obese individuals. The study's aim is to determine the effect on CVD morbidity and mortality of an intensive weight loss intervention program based on an energy-restricted traditional Mediterranean diet, increased physical activity and behavioral therapy in comparison with an intervention based on standard dietary advice (energy-unrestricted Mediterranean diet) and traditional health care for CVD prevention. We hypothesize that an intensive lifestyle intervention program aimed at weight loss and based on the traditional Mediterranean diet is a sustainable long-term approach for achieving weight loss in overweight and obese adults and that the lifestyle changes achieved will have a beneficial effect on cardiovascular morbidity and mortality (Estruch et al., 2013; Shai et al., 2008). This study may provide a useful tool for tackling excess morbidity and mortality associated with overweight and obesity.

Objectives

The PREDIMED-PLUS trial will evaluate the effect on primary CVD prevention of an intensive intervention program comprising a 17-item energy-restricted Mediterranean

diet with specific weight-loss goals, physical activity promotion and behavioral support (intervention group) in comparison to a control group using Mediterranen diet recommendations (comprising a 14-item energy-unrestricted Mediterranean diet) but without advice to increase physical activity or reduce energy intake (Schröder et al., 2011) (control group). The diet that will be assigned to the control group has previously been shown to be effective for CVD prevention in the PREDIMED trial (Estruch et al., 2013). The primary end-point will be a composite of hard clinical cardiovascular events (myocardial infarction, stroke or cardiovascular death) as defined in the PREDIMED trial (Martinez-Gonzalez et al., 2012).

The main objective of PREDIMED-PLUS is to evaluate, in comparison with a **control group** given non-intensive, energy-unrestricted dietary advice (also Mediterranean-type), the effect of an **intensive lifestyle intervention** comprising an **energy-restricted Mediterranean diet**, increased physical activity and behavioral support on:

1. The incidence of **cardiovascular events** (non-fatal myocardial infarction, non-fatal stroke, or cardiovascular death).

2. **Weight loss** and long-term maintenance of weight-loss.

The secondary objectives are to determine whether an intensive weight-loss-oriented lifestyle intervention program has a beneficial effect to reduce waist circumference and the following overweight- and obesity-related conditions: acute coronary syndromes with or without coronary revascularization, heart failure, atrial fibrillation, peripheral artery disease, venous thrombosis, type-2 diabetes mellitus and its complications, overall incidence of cancer, specific cancers in main cancer sites (breast, colorectal, prostate, lung and stomach), osteoporotic fractures, gallstone disease, symptomatic gout, neurodegenerative disorders (dementia and Parkinson’s disease), unipolar depression and eating behavior disorders. We will also determine the effect of the intervention on the following intermediate markers: nutrient intake and overall dietary pattern, systolic and diastolic blood pressure, blood lipids, fasting glucose level, kidney function, uric acid level, the percentage of individuals requiring anti-hypertensive, anti-diabetic or lipid-lowering medication, C-reactive protein levels, hemoglobin A1C levels, liver function, ECG traits, cognitive function, quality of life, and psychopathological symptoms.

Methodology

We are conducting a randomized, multicenter **field trial** aimed at the primary prevention of CVD in overweight or obese adults with metabolic syndrome using an intensive intervention program based on an energy-restricted Mediterranean diet, increased physical activity, and behavioral support and a control group given advice on an *ad libitum* Mediterranean diet for the prevention of cardiovascular morbidity and mortality in accordance with the PREDIMED trial. We stress to participants the importance of attending medical visits and provide them with general written recommendations on lifestyle for the management of the metabolic syndrome. This new trial, the planning and design of which is outlined in this paper, is called **PREDIMED-PLUS**.

Involved in this new multicenter trial are 20 recruiting centers that aim to recruit a total of **6,000 participants**, of whom **3,000** will be assigned to the intensive intervention group and **3,000** to the control group. Recruitment takes place between 2013 and 2017. The intervention will last at least 6 years and the median follow-up time for the clinical endpoints is expected to be 8 years. The results of the trial, including anthropometric changes and impact on major obesity-related disorders, are expected to be highly applicable to public healthcare since they will provide a better prognosis for overweight and obese adults. The results are also expected to be highly efficient since they should provide a non-pharmacological approach to the prevention of the main cause of mortality and one of the leading causes of loss of disability-adjusted life years (Lozano et al., 2012).

BACKGROUND

The **global overweight and obesity epidemic** is increasing at an alarming rate. Now a global public health crisis, it affects over 60% of the adult population. Between 1980 and 2008, worldwide obesity prevalence almost **doubled** (Finucane et al., 2011; Malik et al., 2013). In Spain, the **prevalence of adult abdominal obesity is over 35%** and **over 60%** of the adult population is overweight or obese (Gutiérrez-Fisac et al., 2012). Moreover, there is a particularly worrying increase in morbid obesity (Basterra-Gortari et al., 2011). The medium-to-long-term consequences of obesity on the risk of CVD and death are **devastating** and have the capacity to both **render the health system unsustainable** and curtail economic growth. Urgent priority must be given to finding solutions based on the best scientific evidence available.

The link between obesity and mortality appears to diminish with age. However, if this were true, it would be difficult to recommend weight loss for older populations. This notion has been challenged by observational epidemiological studies that, after generational and cohort confusion are adequately controlled, suggest the opposite, i.e., that with advancing age the link between obesity and mortality becomes even stronger (Masters, 2013).

A meta-analysis of observational studies published in 2013 (Flegal, 2013) raised controversy by considerably downgrading the consequences of overweight and moderate obesity. However, as Tobias and Hu (2013) have argued, the conclusions of Flegal's meta-analysis can probably be explained by the existence of biases, such as unusual definitions for categories of overweight, tobacco as a confounding factor, inverse causality, and the exclusion of relevant studies. This issue will only be solved by randomized clinical trials (Hernán and Taubman, 2008). However, the controversy is likely to continue well into the next few decades until the results of clinical intervention studies are available that overcome the limitations and inherent bias of the observational designs that have evaluated the link between overweight or obesity and the incidence of serious clinical events or mortality in initially healthy individuals. Observational studies with a better control of bias have found that all-cause mortality increases progressively as adiposity outside the normal weight range—measured by body mass index (BMI, defined as weight in kilograms divided by the square of height in meters)—increases, and that this risk is especially high for **cardiovascular mortality** (Berrington de González et al., 2010). An increase in body weight is associated not only with higher mortality but also with greater morbidity due to CVD (Ni Mhurchu et al., 2004; Song et al., 2004; Flint et al., 2010); greater risk of developing some types of cancer, diabetes and depression (Luppino et al., 2010); and poorer cognitive function (Gunstad et al.,

2010). Large-scale randomized studies with robust designs are needed to obtain best-quality evidence.

Expert panels set up by the National Institutes of Health and the World Health Organization recommend that overweight and obese adults with comorbid conditions should lose 10% of their initial weight and that a lifestyle intervention program should be the primary treatment (National Institute of Health, 1998; World Health Organization, 1998). Moreover, according to the American Dietetic Association, a weight-loss-oriented intensive lifestyle intervention program should include an energy-restricted diet, physical activity and behavioral therapy. The only randomized trial that has addressed the long-term effect of an intensive weight-loss lifestyle program in obese adults on CVD and mortality is the Look AHEAD study (Ryan, 2003). This trial, which comprised 5,145 participants (Rejeski et al., 2012), ended prematurely in October 2012 due to lack of efficiency (Look AHEAD Research Group, 2013). The trial included only diabetic subjects and used a low-fat diet (<30% of total energy intake with <10% from saturated fat). To some extent this is opposite to the Mediterranean diet used in the PREDIMED trial (Zazpe et al., 2008; Martínez-González et al, 2012; Estruch et al., 2013), which is rich in vegetable fats such as healthful virgin olive oil and nuts. In recent years, scientific associations have recommended low-fat diets that contribute less than 30% of energy in the form of fat as the most suitable way to promote general health and weight loss. The high energy density and high palatability of high-fat foods are feared to produce potentially adverse effects on body weight and cardiovascular health (National Institute of Health, 2000). However, the discontinuation of the Look AHEAD trial due to futility, the inefficiency of the Women's Health Initiative Dietary Modification Trial (Look Ahead Research Group, 2013; Howard, 2006), and the favorable results of the PREDIMED (Estruch et al., 2013) and DIRECT studies (Shai et al., 2008) provide powerful arguments against approaches based on low-fat diets.

Although diets that recommend complex carbohydrates, a reduction in fat intake and energy restriction to produce weight loss are generally accepted, there is no clear evidence that dietary fat is associated with a greater increase in weight (Willett, 2001; Nordmann et al., 2006; Larsen et al., 2010; Hu et al., 2012; Bueno et al., 2013). One dietary paradigm that is different from the low-fat diet and that can be more useful for developing and implementing programs aimed at achieving prolonged weight loss and improving the metabolic alterations associated with overweight and obesity is the **traditional Mediterranean diet**. This dietary pattern is rich in fat from vegetable sources (virgin olive oil and nuts) and includes an abundance of minimally processed plant foods (vegetables, fruits, whole grains and legumes), low consumption of meat (especially red

and processed meats), moderate consumption of fish and wine (which is usually consumed with meals) and frugal meals. The high fat contents of the traditional Mediterranean diet make the diet more palatable and therefore more acceptable and easily sustainable in the long term.

In its 2010 edition, the Dietary Guidelines for Americans recognized the traditional Mediterranean diet, together with the DASH diet, as a healthy diet for the prevention of CVD, although when the recommendation was made, randomized clinical trials with regard to the primary prevention of major clinical events as the main outcome had not yet been conducted. This was confirmed by the results of the PREDIMED trial on the primary prevention of cardiovascular disease conducted in Spain between 2003 and 2010 (Estruch et al., 2013).

With regard to the weight-loss properties of the traditional Mediterranean diet, in a meta-analysis of randomized trials, allocation to a Mediterranean diet in comparison with control diets showed a small but significant effect on body weight reduction (mean differences: -1.75 kg, CI 95%: -2.86 a -0.64 kg). This effect was doubled when the Mediterranean diet was energy-restricted (Esposito et al., 2011). Another meta-analysis of observational studies (Sofi et al., 2010) found that greater adherence to the Mediterranean diet was associated with significant reductions in total mortality, cardiovascular mortality, mortality due to cancer, the incidence of non-fatal cardiovascular events, and the risk of neurodegenerative illnesses. A subsequent update of the meta-analysis of the Mediterranean diet and CVD reported a 13% relative reduction in risk for every two-point increase in adherence to the Mediterranean diet (scale 0–9) after identifying and treating sources of heterogeneity (Martínez-Gonzalez and Bes-Rastrollo, 2014).

The **PREDIMED** (**PREvención con DIeta MEDiterránea**) trial, which included 7,447 participants over an average of five years, was the largest nutritional intervention **trial** ever conducted **in** Europe. PREDIMED showed that, in comparison with advice on a low-fat diet, a high-fat **Mediterranean diet** supplemented with extra-virgin olive oil or mixed nuts implemented in a setting of primary cardiovascular prevention resulted in a **30% reduction in CVD events** after intervention for a median of 4.8 years (Estruch et al., 2013). **PREDIMED** is recognized worldwide as a landmark study that marks a turning point in the **prevention of chronic diseases**. The effective reduction in cardiovascular events when the **Mediterranean diet** was used in a randomized trial provides the best-possible scientific evidence for preventing **CVD**, **the main cause of death in the world**. We should also point out that the **PREDIMED** diets were *ad libitum*, increased physical activity was not promoted, and no counsel to lose weight was given.

It has been postulated that the link between adherence to the traditional Mediterranean diet and the risk of CVD can be mediated by several mechanisms, including a reduction in low degree inflammation (Chrysohoou et al., 2004; Esposito et al., 2004; Mena et al., 2009; Camargo et al., 2011; Urpi-Sarda et al., 2012; Meneses et al., 2012), higher levels of adiponectin (Detopoulou et al., 2010; Razquin et al., 2010), lower coagulability (Chrysohoou et al., 2004; Pérez-Jiménez et al., 2006; Pérez-Jiménez et al., 2002), improved endothelial function (Esposito et al., 2004; Ruano et al., 2005; Fuentes et al., 2008), lower oxidative stress (Dai et al., 2008; Chrysohoou et al., 2011; Razquin et al., 2009), a lower concentration of atherogenic lipoproteins (Jones et al., 2012), lower levels of oxidized LDL particles (Fito et al., 2007), and a lower uptake of oxidized LDL by macrophages (Moreno et al., 2008). Moreover, the two foods supplemented in PREDIMED (extra-virgin olive oil and nuts) also have beneficial biological properties. Extra-virgin olive oil has a healthy fatty acid profile and contains numerous bioactive phenolic compounds (Pérez-Jiménez et al., 2006; Covas et al., 2009; López-Miranda et al., 2010). The phenolic compounds of olive oil have anti-inflammatory properties (Fito et al., 2008), beneficially impact he lipid profile (Benkhalti et al., 2002; Covas et al., 2006), improve oxidative stress markers (Covas et al., 2006), have a platelet antiagregant effect (de Roos et al., 2011; Fito et al., 2008), and stimulate mitochondrial biogenesis (Zhu et al., 2010). Nuts also have a healthy fatty acid profile, based on mono- and polyunsaturated fatty acids, and contain minerals, vitamins and other antioxidant bioactives, essential amino acids, fiber, and phytosterols (Ros, 2009). The consumption of nuts has been associated with lower levels of total cholesterol, LDL and non-HDL cholesterol, and apolipoprotein B-100 (Li et al., 2009; Sabaté et al., 2010), and lower inflammation (Jiang et al., 2006). Nuts also have an antioxidant effect, benefit heart rate and improve platelet aggregation and endothelial function (Ros, 2009; Defilippis et al., 2010). All of these mechanisms explain the antiatherogenic effect of a Mediterranean diet that is rich in nuts and extra-virgin olive oil. In fact, in the PREDIMED trial a strong protective effect against peripheral artery disease was observed (Ruiz-Canela et al., 2014). In a sub-study of the PREDIMED trial we also observed that both a Mediterranean diet enriched with nuts and a Mediterranean diet enriched with olive oil reduced the incidence of type-2 diabetes by 48% (Salas-Salvadó et al., 2011). When we analyzed this association among all study's participants, we also found that the Mediterranean diet had a significant protective effect against diabetes (Salas- Salvadó et al., 2014).

Though PREDIMED study, was not a weight loss trial, the provision of abundant fat-rich foods from natural vegetable origin (extra-virgin olive oil and nuts) did not conduct

to weight gain. There is still insufficient **experimental evidence** to support the hypothesis that intentional weight loss via a healthy diet and favorable lifestyle changes reduces mortality or the incidence of CVD in the long term. Specifically, the impact of weight loss on the risk of CVD within the framework of a **Mediterranean dietary pattern** has not yet been tested in a sufficiently large randomized clinical trial (Malik and Hu, 2007). In light of the obesity epidemic, we propose to conduct a new trial, **PREDIMED-PLUS**, which will go beyond the achievements of the **PREDIMED** trial in order to tackle more specifically the problems of overweight and obesity. Our proposed strategy has positive effects for weight loss (based on the loss of fat mass) and long-term weight-loss maintenance (Shai et al., 2008; Beunza et al., 2010; Romaguera et al., 2010). Even more interestingly, this research may demonstrate that a **multifaceted lifestyle intervention** program (dietary pattern + weight loss + physical activity + behavioral support) can be an even more effective means of reducing the cardiovascular risk associated with overweight and obesity than a non-energy-restricted traditional Mediterranean diet. We expect our contribution via the **PREDIMED-PLUS** trial to reveal synergies between the effects of an intensive weight-loss intervention program (with **energy restriction**, physical activity and behavioral support) and the beneficial effects of greater adherence to a high-quality diet (the **Mediterranean diet**) on the incidence of CVD.

Blood and urine samples will be collected and stored at the beginning and throughout the trial. Later analyses of molecular/biochemical biomarkers within the framework of genetic, epigenetic, transcriptomic, metabolomic and proteomic studies might help to determine the benefits of the intervention and the underlying mechanisms.

HYPOTHESIS

An **intensive lifestyle intervention program** based on an energy-restricted **traditional Mediterranean diet**, increased physical activity and behavioral support is a **sustainable** approach that leads to long-term weight loss in overweight and obese adults with metabolic syndrome in such a way that the changes in lifestyle achieved will have long-term benefits on the incidence of CVD.

In comparison with a control intervention that provides advice on the Mediterranean diet but does not restrict calorie intake and does not promote physical activity, an intensive lifestyle intervention based on an energy-restricted **traditional Mediterranean diet**, promotion of physical activity, and behavioral support (Intervention group) in overweight or obese individuals with metabolic syndrome will:

1. Reduce the risk of cardiovascular events;
2. Achieve a greater reduction in body weight and lead to better long-term weight-loss maintenance;

OBJECTIVES

Our long-term objective is to provide effective treatment for reducing excessive **cardiovascular morbidity and mortality** in overweight or **obese** adults**,** irrespective of whether the participants are diabetic at the beginning of the study. To achieve this, we will compare the effects on rates of cardiovascular disease of an **intensive** lifestyle and weight loss intervention program based on the **traditional Mediterranean diet** and including increased physical activity, energy restriction and behavioral support (intervention group) with that of a non-intensive intervention program that provides both education on **the traditional Mediterranean diet for the prevention of CVD in accordance with the principles outlined in the PREDIMED trial and usual care** by primary healthcare professionals (control group). The importance of attending visits to healthcare professionals will be stressed and general recommendations on management of the metabolic syndrome will be provided.

Main specific objectives

To evaluate the effect of an **intensive** weight-loss-oriented lifestyle intervention program based on a **traditional Mediterranean diet** with **energy restriction**, **increased physical activity and behavioral** therapy on:

1. The incidence of CVD (non-fatal myocardial infarction, non-fatal stroke and cardiovascular death);
2. **Weight loss and long-term weight-loss maintenance**;

Secondary specific objectives

This intensive intervention program is likely to result in reduction of waist circumference and acute coronary syndromes, coronary revascularization, total mortality, heart failure, peripheral artery disease, venous thrombosis, atrial fibrillation, type-2 diabetes and its complications, total cancer, cancer in main cancer sites (breast, prostate, colorectal, lung and stomach), gallstone diseases, symptomatic gout, neurodegenerative disorders (dementia and Parkinson’s disease), unipolar depression, osteoporotic fractures, and eating behavior disorders.

We will also address the effect of the intervention on the following intermediate outcomes: nutrient intake and overall dietary pattern, systolic and diastolic blood

pressure, serum lipid concentrations, fasting glucose, glycated hemoglobin and uric acid, kidney function, liver function, C-reactive protein, anti-hypertensive, anti-diabetic and lipid-lowering medication needs, ECG traits, cognitive function, quality of life, and psychopathological scales.

We will also store plasma, serum, peripheral cells and urine samples to evaluate other hypotheses in the future, depending on availability of additional funding.

METHODOLOGY

1. Summary

We are conducting a parallel-group, multicenter, randomized, primary prevention trial in adult **men aged 55–75** and adult **women aged 60–75** with a **BMI ≥27 and <40 kg/m^2** who meet at least **three criteria for the metabolic syndrome**. In the Caucasian population, cardiovascular risk is considered to be increased if waist circumference is ≥ 80 cm in women and ≥ 94 cm in men. In the South American population, the value is the same for women but for men risk is considered to be increased if waist circumference is ≥ 90 cm (Alberti et al., 2009). Diabetic participants will comprise no more than 25% of the final sample. This latter component and the use of the **traditional Mediterranean diet** will substantially distinguish the PREDIMED-PLUS trial from the Look AHEAD trial conducted in the United States (Ryan et al., 2003; Gregg et al., 2012; Look AHEAD Research Group, 2013), which was recently discontinued due to lack of efficiency. Participants will be divided proportionally at random into two groups: a control group and an intensive lifestyle intervention group. Intervention will be maintained for **6 years** and average follow-up time **for clinical events will be 8 years**.

The control group receives usual healthcare from primary care medical professionals, all the written material, instructions on following the traditional **Mediterranean diet**—which was used in the PREDIMED study and has been shown to have benefits for the prevention of cardiovascular morbidity and mortality—and general lifestyle recommendations for managing the metabolic syndrome. Every six months, the control group participants are also invited to participate in group sessions led by the team of PREDIMED-PLUS dietitians, wherein they receive a free supply of virgin olive oil (6 liters every 6 months) and nuts (3 kg every 6 months) in order to promote the Mediterranean diet and encourage compliance with the trial.

Participants in the **intensive lifestyle intervention** group are prescribed a **traditional Mediterranean diet** but in this case it is **energy-restricted**. Dietary intervention is associated with increased physical activity and behavioral therapy programs. It has specific weight-loss objectives and includes self-monitoring and

frequent follow-up throughout the study. Participants in this group take part in individual interview sessions and motivational group sessions three times per month during the first year of the intervention and twice per month thereafter. They are provided free extra-virgin olive oil (one liter per month) and nuts (500 g per month)*. The participants' degree of compliance with the intervention is monitored periodically so that the intervention can be adjusted if necessary.

For the intensive intervention group, the specific weight-loss objectives are to achieve **an average reduction in baseline body weight of over 8%** and an average **reduction in waist circumference of over 5%** in the first six months and to maintain these figures over an additional period of seven and a half years. The final objective is to obtain a between-group average absolute difference in weight loss and waist circumference reduction of over 5%.

Primary final outcomes include: a) non-fatal myocardial infarction, b) non-fatal stroke, and c) cardiovascular death. Other primary objective will be weight loss (and weight-loss maintenance). The trial protocol will be registered at ClinicalTrials.gov (National Institutes of Health) and comply with the CONSORT guidelines for the dissemination of results (Moher et al., 2001).

2. Research team

The trial will comprise 6,000 participants, one-half of whom will be assigned to the intensive intervention group and one-half to the control group. Recruitment of participants began in 2013 and will end in 2017. The 2 vanguard centers were Navarra-Epidemiology (starting the recruitment in September 2013 and the randomization in October 2013) and Reus (starting the recruitment in November 2013, and the randomization in January 2014). Training of dietitians for the other recruiting centers took place in December 2013. We assume that we will reach our recruitment objectives with 20 centers each recruiting an average of 300 participants.

To launch the trial, we have set up a team of leading researchers with experience in diet and lifestyle interventions and productive and well-documented collaborative

---

**The decision to administer 500 g of mixed nuts per month was based on the results of epidemiological and clinical studies. For example, the SUN (Seguimiento Universidad de Navarra) study found that individuals who consumed nuts two or three times per week (400 g/month) had a significantly lower risk of weight gain and metabolic syndrome than those who rarely ate them or never did (Fernández-Montero et al., 2012; Bes-Rastrollo et al., 2007). A Mediterranean-type diet with moderate fat intake containing 25 g per day (750 g/month) of peanuts or other types of nuts was found to be associated with better adherence to intervention and greater weight loss than a low-fat diet (McManus et al., 2001). The decision was also based on associations observed between baseline nut consumption and mortality in the PREDIMED trial (Guasch-Ferre et al., 2013) and in American cohorts of nurses and healthcare professionals (Bao et al., 2013).*

research careers in nutrition, evaluation of physical activity, internal medicine, cardiology, endocrinology, primary health care, epidemiology, and basic sciences.

The prior experience gained by the 11 PREDIMED recruiting centers is one of our most valuable assets for the PREDIMED-PLUS trial. Also, by incorporating other centers of scientific excellence with proven experience in nutritional intervention clinical trials (some of which also belong to the background research network of the PREDIMED trial, CIBEROBN), the correct development of the PREDIMED-PLUS trial will be guaranteed and feasibility will be improved.

At the same time, subprojects will be devised so that all participating groups can develop their own specific nutritional research activities. This will also serve to further interest in the project and enhance scientific output.

3. Preliminary studies: the PREDIMED trial

In this section we present a summary of the methodology and key findings of the **PREDIMED** trial (Estruch et al., 2006; Zazpe et al., 2008; Martínez-González et al., 2012; Estruch et al., 2013), which was conducted in the context of CIBEROBN and the PREDIMED network (RD06/0045). PREDIMED was a multicenter, parallel group, trial with three intervention groups (see www.predimed.es). In 2006, the results of a pilot study were published that evaluated the effects at three months of the three interventions on classical and emergent cardiovascular risk factors in the first 772 participants (Estruch et al., 2006). The design and methods of the PREDIMED trial have been described elsewhere (Martínez-González et al., 2012). Participants in the study were men aged 55-80 and women aged 60-80 without CVD at the beginning of the study but with a high risk of CVD due to the presentation of type 2 diabetes or at least three of the following six cardiovascular risk factors: smoking, high blood pressure, high LDL cholesterol level, low HDL cholesterol level, overweight or obesity, and family history of early coronary heart disease.

Candidates for the trial were recruited at primary care health centers. Eighty-nine per cent of those invited to participate agreed to do so and signed the corresponding informed consent form. The final sample size for the trial was 7,447 participants. The protocol was approved by the ethics research committees of all study centers and registered at the Clinical Trials Register in London (ISRCTN35739639).

The participants were randomly assigned in a 1:1:1 ratio to one of the following three dietary intervention groups: 1) an energy-unrestricted **Mediterranean diet** supplemented with **extra-virgin olive oil**; 2) an energy-unrestricted **Mediterranean diet**

supplemented with **nuts**; or 3) an energy-unrestricted **control** diet with advice on how to follow a low-fat diet.

At the beginning of the study and quarterly thereafter, dietitians conducted individual and group dietary-training sessions (separately for each group) with a maximum of 20 participants per group (Zazpe et al., 2008). At each session of the Mediterranean diet groups, a 14-item questionnaire (Martínez-González et al., 2004; Schroeder et al., 2011) was used to assess participants' adherence to the Mediterranean diet. At each session of the control group, a 9-item questionnaire was used to assess participants' adherence to the control diet. In this way the diets could be personalized and appropriate dietary changes could be negotiated individually.

Participants in the two **Mediterranean-diet** groups received either **extra-virgin olive oil** (1 liter per week for the participant and his or her family) or 30 g of **mixed nuts** per day (15 g of walnuts, 7.5 g of hazelnuts, and 7.5 g of almonds) at no cost and in accordance with their randomly chosen group, while those in the control group received small non-food gifts throughout the trial. At no point during the intervention was calorie restriction advised or increased physical activity encouraged.

Information on the primary end points (cardiovascular death, non-fatal stroke, or acute non-fatal myocardial infarction) was obtained through continuous contact with the participants, contact with primary healthcare physicians, *ad hoc* annual reviews of medical records conducted by a medical team at each center, and annual consultation of the National Death Index. The data were analyzed on an intention-to-treat basis. Participants were followed for a median of 4.8 years.

Participants in the two Mediterranean diet groups increased their adherence to the diet, as reflected in an average increase of 2 points on the 14-point dietary-screening questionnaire throughout the duration of the study. These participants also had higher average scores than those in the control group on all items except red and processed meats and sugary soda drinks, which were discouraged for all three intervention groups.

After a median follow-up of 4.8 years, 288 participants suffered a major cardiovascular event. In comparison with the control group, the multivariable-adjusted hazard ratios were 0.70 (95% confidence interval [CI], 0.54 to 0.92) for the group assigned to the Mediterranean diet with extra-virgin olive oil and 0.72 (95% CI, 0.54 to 0.96) for the group assigned to the Mediterranean diet with nuts.

FIGURE 1. Cumulative incidence of major cardiovascular events (cardiovascular death, non-fatal myocardial infarction, and non-fatal stroke) by randomly assigned group (n=7,447).

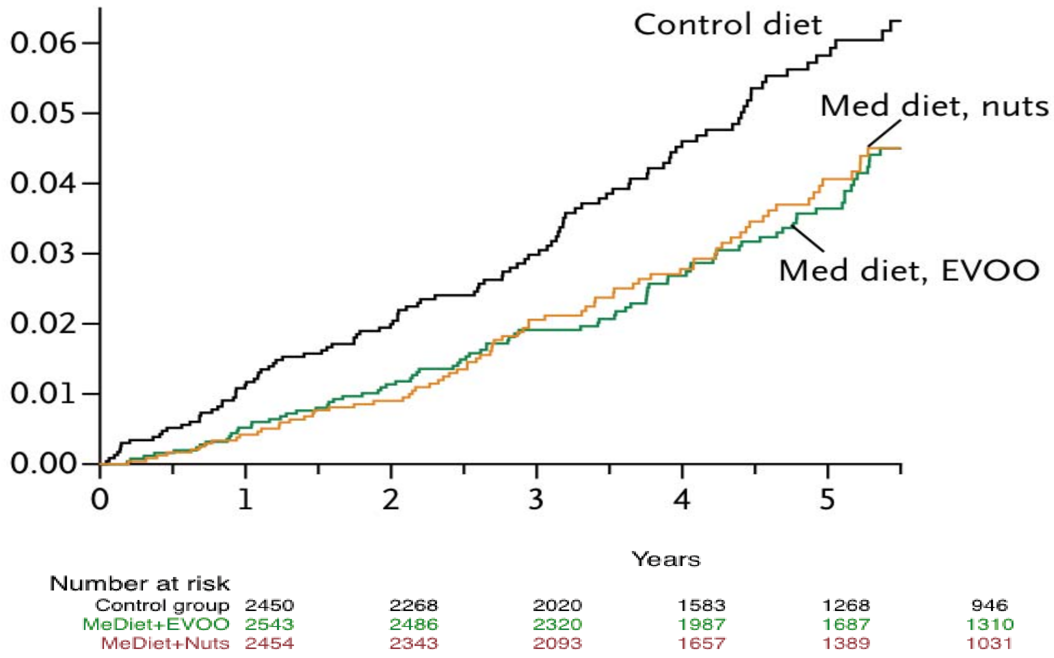

4. Proposed trial (PREDIMED-PLUS) and eligibility criteria

Candidates for the PREDIMED-PLUS trial are adults aged 55-75 for men and 60-75 for women with a body mass index ≥27 and <40 kg/m^2 who meet at least three criteria for the metabolic syndrome (Alberti et al., 2009). These criteria must be taken into consideration in view of recent evidence of the beneficial role of the Mediterranean diet on the metabolic syndrome (Kastorini et al., 2011; Salas-Salvadó et al., 2008), insulin resistance and diabetes (Salas-Salvadó, 2014), especially when accompanied by a program of physical activity for endurance (Fernández et al., 2012). We will try to ensure that 50% of the study population is made of women and that diabetic participants do not exceed 25% of the total cohort. Individuals who participated in the PREDIMED trial will not be eligible to participate in PREDIMED-PLUS.

4.1. Exclusion criteria:

- Illiteracy or inability/unwillingness to give written informed consent or communicate with study staff.
- Institutionalization (the participant is a permanent or long-stay resident in a care home).

- Documented history of previous CVD, including: angina; myocardial infarction; coronary revascularization procedures; stroke (ischemic or hemorrhagic, including transient ischemic attacks); symptomatic peripheral artery disease that required surgery or was diagnosed with vascular imaging techniques; ventricular arrhythmia; uncontrolled atrial fibrillation; congestive heart failure (New York Heart Association Class III or IV); hypertrophic myocardiopathy; and history of aortic aneurism >=5.5 cm in diameter or aortic aneurism surgery.
- Active malignant cancer or history of malignancy within the last 5 years (except non-melanoma skin cancer).
- Inability to follow the recommended diet (for religious reasons, swallowing disorders, etc.) or to carry out physical activity.
- A low predicted likelihood to change dietary habits according to the Prochaska and DiClemente Stages of Change Model (Nigg et al., 1999).
- Inability to follow the scheduled intervention visits (institutionalization, lack of autonomy, inability to walk, lack of stable address, travel plans, etc.).
- Inclusion in another program that provides advice on weight loss (> 5 kg) in the six months before the selection visit.
- History of surgical procedures for weight loss or intention to undergo bariatric surgery in the next 12 months.
- History of small or large bowel resection.
- History of inflammatory bowel disease.
- Obesity of known endocrine origin (except for treated hypothyroidism).
- Food allergy to any component of the Mediterranean diet.
- Immunodeficiency or HIV-positive status.
- Cirrhosis or liver failure.
- Serious psychiatric disorders: schizophrenia, bipolar disorder, eating disorders, or depression with hospitalization within the last 6 months.
- Any severe co-morbidity condition with less than 24 months' life expectancy.
- Alcohol abuse or addiction (or total daily alcohol intake >50 g) or drug abuse within the past 6 months.
- History of major organ transplantation.
- Concurrent therapy with immunosuppressive drugs or cytotoxic agents.
- Current treatment with systemic corticosteroids.
- Current use of weight loss medication.
- Concurrent participation in another randomized clinical trial.

• Patients with an acute infection or inflammation (e.g. pneumonia) will be allowed to participate in the study 3 months after resolution of their condition.
• Any other condition that may interfere with adherence to the study protocol.

5. Recruitment and retention strategies

Medical doctors from primary care centers associated with the recruiting centers recruit the participants. The mission of the primary care physicians is to ensure a high recruitment rate and an almost 100% diligence in the revision of medical records and collection of clinical information on events during follow-up. As the physicians involved in the recruitment process will also be responsible for the participants' medical care, no potential ethical conflict regarding confidentiality exists when identifying suitable candidates or reviewing medical records. Participants' eligibility criteria and demographic data are collected from the medical records at the primary care centers, which are entirely computer-based. This is done at a pre-screening evaluation stage before the potential participant is contacted. Candidates are interviewed briefly by telephone, informed about the study, and invited to attend a screening visit at the recruiting center.

In this first formal visit (first screening visit), the candidates are explained the purpose and characteristics of the study and, if they agree to take part, they are asked to sign a written informed consent. Our experience with the PREDIMED trial showed that over 95% of eligible candidates approached in this way agreed to participate (Martínez-González et al., 2012; Estruch et al., 2013). Also, in the PREDIMED trial, which also included a long-term lifestyle intervention program (median follow-up time of 4.8 years), overall retention rate was above 90% (Estruch et al., 2013).

6. Informed consent/Ethics Committee

The institutional review boards of all the recruiting centers approved the study protocol. As described and detailed below, all participants sign informed written consent forms.

7. Launch of the trial

The proposed calendar was as follows:

- Between August 2013 and January 2014, the dietitians and nursing staff were hired and trained to deliver the trial's protocol.
- Between August 2013 and May 2014, eligible candidates began to be called, interviewed and invited to participate in the trial.

- Next, the first evaluation and intervention visits took place with randomly distributed selected participants.
- The recruitment period will end on December 2016.

7.1. The aim of the initial stage of the trial (telephone calls and interviews) is to evaluate the willingness of each candidate to participate in the study, comply with the proposed intervention, and lose weight. In addition, they are thoroughly screened in order to ensure that the eligibility criteria are met and evaluate the probability that they will:

a) attend the scheduled sessions,

b) complete the protocol's assessment tools, i.e., the self-monitoring and recording of lifestyle and food habits and, most importantly,

c) change their dietary habits in accordance with the Stages-of-Change model (Nigg et al., 1999), as occurred in the PREDIMED trial.

7.2. The run-in period (for evaluation prior to randomization) lasts four weeks. It comprises an initial screening visit, a phone call at 2 weeks, and a final evaluation visit.

7.2.1. The first screening visit (45-60 min) comprises:

a) administration of a questionnaire on inclusion and exclusion criteria. Candidates who are deemed eligible to participate in the trial continue to the next stage.

b) explanation of the study, distribution of the **study information sheet**, and completion of the **informed consent forms** (these are essential for inclusion in the trial). Eligible candidates are asked to sign two informed consent forms: one for participation and analysis of general variables and one for the collection of DNA for genetic analyses. All procedures and anticipated time commitment are explained in detail. Candidates are also told that, if they do not satisfy the eligibility criteria, they will be excluded from the study. The informed consent form includes a statement allowing researchers to review the participants' medical records throughout the trial at both the primary care centers and reference hospitals in order to ascertain the occurrence of any events.

c) performance of ECG and recording of height, weight, waist circumference and blood pressure.

d) distribution of a leaflet containing general recommendations on managing the metabolic syndrome.

e) Distribution of a 3-day food record questionnaire (2 working days and 1 weekend day), a leisure-time physical activity questionnaire, and a self-measurement chart in which participants self-record their weight, waist circumference and hip circumference

(participants are given a tape measure). Dietitians give instructions on how and when to complete the food record and physical activity questionnaires and how to record their weight, waist and hip measurements (once a week during the trial).

f) Distribution of the clinical psychopathological questionnaires (Beck Depression Inventory (BDI-II), multidimensional scale of weight locus of control, eating disorders diagnostic criteria, and quality of life scale (SF-36)) to be completed at home (see below).

Participants are asked to return their completed questionnaires at the third screening visit (see below).

7.2.2. Second screening visit. After 2 weeks, participants receive a telephone call to assess their change in weight and remind them to bring to the next screening visit their completed food record, and physical activity questionnaires, self-measurement chart, psychopathological questionnaires, and quality of life scale.

7.2.3. Third screening visit. This evaluation visit on completion of the four-week run-in period (30 min) includes:

a) Collection by the dietitian of the participants' food record and physical activity questionnaires, self-measurement charts, psychopathological questionnaires, and quality of life scales.

b) Measurement by the dietitian of the participants' weight and hip circumferences.

c) Administration and completion of a 143-item food-frequency questionnaire and 5 cognitive-neuropsychological tests, which, unlike the clinical psychopathological questionnaires, must be completed in the presence of PREDIMED-PLUS personnel. These 5 tests are: the Mini-Mental State examination, the phonological verbal fluency test, the reverse digits test, the trail making test, and the clock test (see below).

d) Explanation to participants that they will be informed by telephone if they have been selected to participate in the trial.

e) Explanation of night-time fasting for *in situ* extraction of blood sample and first morning urine sample, and of basal evaluation immediately after randomization for candidates who are chosen to participate in the trial.

Only candidates who satisfy the following four criteria are selected and randomly assigned to one of the two intervention groups:

1) Full attendance at the two previous sessions, at the scheduled times and having answered the telephone call;

2) Correct completion of the clinical psychopathological questionnaires (Beck Depression Inventory (BDI-II), multidimensional scale of weight locus of control, eating disorders diagnostic criteria, and quality of life scale (SF-36);
3) Correct completion of the food record and physical activity questionnaires;
4) Correct self-recording of at least three weight measurements and three waist-circumference measurements.
5) Loss of >1.5 kg during the run-in period.

The lag time between completion of the run-in phase and the beginning of the intervention ranges from one week to one month.
Exceptionally, depending on the availability of the candidate to attend the Run-in S3 visit, and considering that the 4 mentioned criteria are met, the candidates can be randomized to one of the two intervention groups after completing Run-in S2 visit face-to-face.

8. Initial screening, follow-up visits, and evaluations

Table 1 shows the main data collection measurements and activities by visit.

TABLE 1. The following data are collected per visit in the PREDIMED-PLUS trial.

	RUN-IN PERIOD												
	S1	S2	S3	Baseline	M-6	Y-1	Y-2	Y-3	Y-4	Y-5	Y-6	Y-7	Y-8
1. ELIGIBILITY QUESTIONNAIRE	X												
2. 3-DAY FOOD REGISTER	e		c										
3. ANTHROPOMETRIC MEASUREMENTS*	X		X	X	X	X	X	X	X	X	X	X	X
4. GENERAL QUESTIONNAIRE				X									
5. 143-ITEM FFQ			X		X	X	X	X	X	X	X	X	X
6. MEDITERRANEAN DIET QUESTIONNAIRE (17/14-Items)**				X	X	X	X	X	X	X	X	X	X
7. PHYSICAL ACTIVITY QUESTIONNAIRE‡	e†		c†	X	X	X	X	X	X	X	X	X	X
8. CHAIR TEST (Physical activity evaluation)				X	X	X	X	X	X	X	X	X	X
9. ACCELEROMETERS			e	X	X	X	X	X	X	X	X	X	X
10. FOLLOW-UP QUESTIONNAIRE					X	X	X	X	X	X	X	X	X
11. ELECTROCARDIOGRAM	X				X	X	X	X	X	X	X	X	X
12. BLOOD PRESSURE MEASUREMENT	X		X	X	X	X	X	X	X	X	X	X	X
13. BLOOD SAMPLE COLLECTION				X	X	X		X		X		X	X
14. MORNING SPOT URINE COLLECTION				X	X	X		X		X		X	X
15. NAIL COLLECTION				X		X		X		X		X	X
16. COGNITIVE-NEUROPSYCHOLOGICAL TESTS≠			X				X		X		X		X
17. PSYCHOPATHOLOGICAL QUESTIONNAIRES€	e		X			X	X	X	X	X	X	X	X
18. QUALITY OF LIFE QUESTIONNAIRES≈	e		X			X		X		X		X	

S: Screening visit; FFQ: Food-frequency questionnaire; M: Month; e: Delivery ; c: Collection.
* Anthropometric measurements include: weight, height, waist circumference and hip circumference.
‡ Short version of the Minnesota leisure time physical activity questionnaire; PAR-Q, RAPA (RAPA1 and RAPA2) questionnaires; and the NHS sedentary lifestyle questionnaire
† Long version of the Minnesota leisure time physical activity questionnaire.
**Short questionnaires on adherence to the Mediterranean Diet. The control group uses the same 14-item questionnaire that was used in the PREDIMED trial (Schroeder et al., 2011). The intervention group uses the 17-item energy-restricted Mediterranean diet questionnaire (see below).
≠Mini-Mental Status Examination, clock test, phonological verbal fluency test (animals + P), the reverse series of digits test (WAIS-III), and the trail making test.
€Beck Depression Inventory (BDI-II), multidimensional scale of weight locus of control, eating disorders diagnostic criteria, and SF-36 quality of life scale.

Eligibility: Eligibility for inclusion in the trial is assessed at the beginning of the study. Selected participants must satisfy all the eligibility criteria. Exclusion criteria are also verified.

Anthropometric measurements: Weight and waist circumference will be recorded at each visit, with participants in light clothing and without shoes or accessories, using a high-quality electronic scale that will be calibrated every 3 months with a unit of known mass. Height is measured at study entrance with a stadiometer. Waist circumference is measured midway between the lowest rib and the iliac crest. Hip circumference is measured at the widest part at the baseline visit and on a yearly basis.

General information: Information on medical history, family history and use of medication is collected at the baseline visit by means of the general questionnaire, using the same protocol as in the PREDIMED trial (See supplementary file in Estruch R et al., New Engl J Med 2013).

Evaluation of food habits and dietary intake: The previously validated 143-item food-frequency questionnaire is administered at the third screening visit and at each annual follow-up visit to evaluate the diet of each participant (Fernández-Ballart et al., 2010). In addition, the 17-item energy-restricted Mediterranean diet questionnaire (see below) is completed at each visit. This questionnaire, which includes several changes with respect to a previously validated tool (Schroder et al., 2011), is used both to assess participants' compliance with the intervention and to guide the individual motivational interviews during follow-up. The control group, on the other hand, is administered the same 14-item questionnaire that was used for the PREDIMED trial (Schröder et al., 2011).

Physical activity: Except for the first screening visit (when participants complete the long Minnesota leisure time physical activity questionnaire), at the beginning of the study, after 6 months, and during the follow-up visits, participants will complete a short version of a previously validated physical activity questionnaire (Elosua et al., 1994; Elosua et al., 2000). Also at these latter visits (baseline, after 6 months, and during the follow-up visits), participants will perform the chair test (30 seconds) in order to evaluate their physical fitness and complete the following questionnaires: the PAR-Q (Physical Activity Readiness Questionnaire), the RAPA (RAPA1 and RAPA2) (Rapid Assessment of Physical Activity), and the NHS (Nurses' Health Study) sedentary lifestyle questionnaire. All these questionnaires are described in the PREDIMED-PLUS website: http://www.PREDIMEDPLUS.COM. Each participant randomly assigned to the intervention group is also provided with a pedometer (Yamax SW200 Digi-Walker) to self-monitor the number of steps walked per day. GENEActiv accelerometers are provided as well to a subset of participants (50% of participants in the intensive

intervention group and 20% of those in the control group) in order to quantify physical activity at baseline, 6 months, 1 year, and each year thereafter. In accordance with an evaluation based on physical status, recommendations on aerobic physical activities and strength training are progressively made and activities to improve balance and flexibility encouraged on completion of physical activity.

Self-reported information during follow-up. At the follow-up visits, participants are asked about clinical events that may have occurred between visits and information about medication prescribed is updated.

Evaluation of adverse effects: At 6 months and yearly thereafter, participants complete a specific questionnaire to report any adverse effects felt to be derived from the intervention or weight loss.

Electrocardiogram: ECGs are performed at the primary care centers at the first screening visit, 6 months, and at annual follow-up visits thereafter. The ECGs will be scanned, stored and registered in the specific database designed for that purpose. The nursing staff at each recruiting center will be responsible for receiving and scanning the ECGs, digitizing their contents, and maintaining the registry and database.

Fasting blood collection: Fasting blood samples are collected at the baseline visits, 6 months, 12 months, 3 years, 5 years, 7 years, and at the final follow-up visit. Conventional analyses [lipid profile (total cholesterol, HDL-cholesterol, LDL-cholesterol, and triglycerides), fasting plasma glucose, blood cell count, serum sodium, potassium, calcium, uric acid, urea, creatinine, albumin, C-reactive protein, erythrocyte sedimentation rate, hemoglobin A1C, liver function tests (serum bilirubin, alkaline phosphatase, alanine transaminase, aspartate aminotransferase and gamma-glutamyltranspeptidase) and optional coagulation tests (prothrombin time, activated partial thromboplastin time and fibrinogen)] are performed at the baseline and follow-up visits according to the trial protocol. The nursing staff at the recruiting centers is responsible for collecting, processing, delivering, storing and preserving the samples, digitizing the information, and maintaining the registry and database for all samples and analyses.

Morning spot urine collection: A sample of morning spot urine is collected *in situ* at the baseline visit, 6 months, 12 months, 3 years, 5 years and 7 years, and at the final follow-up visit. Conventional analyses (albumin and creatinine in urine) are performed at the baseline and follow-up visits according to the trial protocol. The nursing staff is responsible for collecting, processing, delivering, preserving, recording, and maintaining the samples.

Nail sample collection: Nail samples are collected *in situ* at the baseline visit, 12 months, 3 years, 5 years and 7 years and at the final follow-up visit. Patients are asked to attend the visits without having cut their toenails. Using toenail clippers, the patients' nails will be cut and placed in labelled zip-lock bags.

Neuropsychological and Quality of Life evaluation:

This evaluation includes three parts:

A) Cognitive Function

B) Quality of life

C) Psychopathology

At baseline and every two years thereafter (2-, 4-, 6- and 8-year follow-up visits), participants complete a battery of 6 tests of *Cognitive Function* (see below, section A). The first (Mini-Mental State Examination (MMSE) is a general screening cognitive test, while the other 5 tests explore different cognitive domains and are aimed at assessin changes in cognitive performance. The *Cognitive Function* tests will be alternated with the *Quality of life* tests (see section B), so that in even years the 6 tests of *Cognitive Function* will be collected and in odd years only the quality of life test will be administered (see below, section B). The 2-year lapse between sequential cognitive tests will reduce bias due to a "learning" effect.

The *Quality of Life* scales (Short -Form 36 or SF -36, see section B) are collected from all participants at the beginning of the study and in odd years thereafter (after 1-, 3-, 5-, and 7- year follow-up visits), while the psychopathology questionnaires (see below, section C) are collected at the beginning of the study and annually.

All instruments included in the *Cognitive* battery (A), *Quality of Life* (B), and psychopathology (C) have been standardized for the Spanish population in the age range of the study. The complete battery of cognitive, quality of life, and psychopathology examinations includes the following tests:

A) *Six cognitive neuropsychological tests (lasting 16 minutes, to be completed in face-to-face interviews)*:

1) MMSE (Folstein et al, 1975);

2) Semantic verbal fluency test: "animals in 1 minute" (Ramier and Hécaen, 1970, 1977; Benton et al., 1994);

3) Phonemic verbal fluency test: "words in 1 minute starting with the letter 'p' (Benton et al., 1994);

4) Verbal and visual working memory: reverse digits test (WAIS-III), Wechsler, 1997):

5) Trail Making Test (Reitan, 1973);
6) Clock test (Clock drawing test or CDT).

Normative data for these tests in the Spanish population have been published by Peña-Casanova et al. (2009a, 2009b). These six tests are collected in the run-in period and each even follow-up year thereafter (years 2, 4, 6 and 8). These six tests, which take roughly 16 minutes to complete, are administered by PREDIMED-PLUS personnel at the third screening visit and each even follow-up year thereafter (years 2, 4, 6 and 8).

B) *One test of Quality of Life (5-10 minutes, to be completed at home)*:
The SF-36 (36-item) quality of life questionnaire (Alonso et al., 1995, 1998; Ware and Gandek 1998) is administered during the run-in period and every odd year of follow-up thereafter (years 1, 3, 5 and 7). In this way, these tests will be alternated with the neuropsychological questionnaires.

C) Three Psychopathological questionnaires (lasting 20-25 minutes, to be completed at home):
1) Beck Depression Inventory (BDI-II) (Beck, Steer and Brown, 1996; Sanz, Navarro and Vázquez, 2003);
2) Multidimensional scale of weight locus control (Wallston, Wallston and DeVellis, 1978);
3) Screening for comorbid eating disorders with diagnostic criteria (DSM-IV-TR; APA, 2000).

The 10 questionnaires above (sections A, B and C) are collected in all participants in the PREDIMED-PLUS study.
The four questionnaires of sections B) and C) are delivered to the participants at the screening visit 1 to be completed at home or at another time outside the study visit. Participants are required to deliver filled-in questionnaires to the recruiting centers within a 15-day period. The same procedure is repeated at follow-up visits when required. The nursing staff at each recruiting center is responsible for collecting, processing, sending, and keeping all the information pertaining to the cognitive tests.
To ensure that graphical data from cognitive tests (drawings of the MMSE and clock and Trail Making tests) are saved for future monitoring. The questionnaires from Group A, B and C tests are collected in paper format, taking advantage of the optical scanning forms for the MMSE, the clock test and the FS-36 designed for this purpose. Once completed,

the MMSE, the clock test and the SF-36 are mailed to the Navarra center (after saving a security photocopy in the recruiting center) to be computerized by optical reading, a time-saving and materially mistake-free procedure.

9. Outcome definition and ascertainment

Clinical events will be ascertained by a Clinical Event Ascertainment Committee led by Dr. Fernando Arós of the Vitoria group. The committee members are M. Aldamiz, A. Alonso, J. Berjón, L. Forga, J. Gállego, M. A. García Layana, A. Larrauri, J. Portu, J. Timiraos, and M. Serrano-Martínez. Clinical event ascertainment will be based on information collected from the participants' medical records, which each year will be reviewed on an *ad hoc* basis by the medical doctors participating in the PREDIMED-PLUS trial. These doctors and the members of the Ascertainment Committee will be blinded to the assignment of participants to the two intervention groups. The reports sent to the Clinical Events Committee will contain no personal information about the participants and will be identified only by a code.

9.1. Primary outcomes

1. Non-fatal acute coronary syndrome (acute myocardial infarction), non-fatal stroke or cardiovascular mortality.

1.a. Acute myocardial infarction (MI) will be defined according to the third universal definition of MI on behalf of the Joint ESC/ACCF/AHA/WHF Task Force (Thygesen et al., 2012) as evidence of myocardial necrosis in a clinical setting consistent with acute myocardial ischemia.

Any one of the following criteria meets the diagnosis for MI:

- Detection of a rise and/or fall of cardiac biomarker values [preferably cardiac troponin (cTn)] with at least one value above the 99th percentile upper reference limit (URL)

  AND

- At least one of the following:
  - (i) Symptoms of ischemia.
  - (ii) New or presumed new significant ST-segment–T wave (ST–T) changes or new left bundle branch block.
  - (iii) Development of pathological Q waves in the ECG.
  - (iv) Imaging evidence of new loss of viable myocardium or new regional wall motion abnormality.

(v) Identification of an intracoronary thrombus by angiography.

Prior MI

Any one of the following criteria meets the diagnosis for prior MI:

• Pathological Q waves with or without symptoms in the absence of non-ischemic causes.

• Imaging evidence of a region of loss of viable myocardium that is thinned and fails to contract, in the absence of a non-ischemic cause.

• Pathological findings of a prior MI

1.b. Stroke will be defined as an acute neurological deficit lasting more than 24 hours caused by an abrupt impairment of brain function due to blockage of blood flow in a particular artery supplying the brain (thrombosis or arterial embolism) or a cerebral haemorrhage.

Ischemic Stroke is defined following the updated definition of stroke for the 21st Century: A Statement for Healthcare Professionals from the American Heart Association/American Stroke Association (Sacco RL, et al. 2013) as an episode of neurological dysfunction caused by focal cerebral, spinal, or retinal infarction. Central nervous system (CNS) infarction is brain, spinal cord, or retinal cell death attributable to ischemia, based on:

1. Pathological, imaging, or other objective evidence of cerebral, spinal cord, or retinal focal ischemic injury in a defined vascular distribution;
2. Clinical evidence of cerebral, spinal cord, or retinal focal ischemic injury based on symptoms persisting ≥24 hours or until death, and exclusion of other potential causes such as hypoglycaemia or seizures.

Silent CNS infarction will not be considered as a primary end-point if defined as imaging or neuropathological evidence of CNS infarction without a history of acute neurological dysfunction attributable to the lesion.

Haemorrhagic Stroke. Stroke caused by intracerebral hemorrhage is defined as rapidly developing clinical signs of neurological dysfunction attributable to an intracerebral hemorrhage, defined as a focal collection of blood within the brain parenchyma or ventricular system that is not caused by trauma. Stroke caused by subarachnoid hemorrhage is defined as a rapidly developing signs of neurological dysfunction and/or headache because of bleeding into the subarachnoid space, which is not caused by trauma.

Silent cerebral hemorrhage will not be considered as primary end-point. It is defined as a focal collection of chronic blood products within the brain parenchyma, subarachnoid space, or ventricular system detected at neuroimaging or neuropathological examination that is not caused by trauma and lacks a history of acute neurological dysfunction attributable to the lesion.

1.c. Cardiovascular mortality: Includes sudden death and non-sudden cardiovascular death (Buxton AE, et al. 2006).

Sudden (cardiac) death is due to cessation of cardiac activity with hemodynamic collapse, typically due to sustained ventricular tachycardia/ventricular fibrillation. It may be:

— Witnessed instantaneously in a previously stable patient. This may occur with or without preceding signs or symptoms, or may occur immediately following sudden dyspnea, light-headedness, or palpitations.

— Unwitnessed. Patient found dead who at the time of last witnessed contact was in his/her usual state of health without medical complaints or obvious difficulty. This applies to patients dying during sleep.

Non-sudden cardiac death: Includes deaths of patients from acute pulmonary edema with severe, progressive heart failure, cardiogenic shock, or after a recent cardiac surgical procedure.

Non-cardiac vascular death: Includes deaths due to thromboembolic events, stroke, dissecting aneurysm and peripheral artery disease.

2. Weight change. The study nurse records weight at each follow-up visit. The measurement are made according to the study manual of operations and with participants dressed in light clothing and no shoes and accessories.

9.2. Secondary outcomes

1. Total mortality. This endpoint comprises all causes of death, including those from CVD (see point 1c of primary end-point), as well as trauma, renal failure, neoplasia, sepsis, suicide and death of undetermined cause. All deaths should be confirmed by reviewing the National Death Index.

2. Changes in waist circumference. The study nurse will measure waist circumference at each follow-up visit according to the manual of operations.

3. Non-ST-segment elevation acute coronary syndrome (unstable angina): The diagnosis of unstable angina will be made following the definition of the ESC Guidelines for the management of acute coronary syndromes in patients presenting without persistent ST-segment elevation (Hamm et al, 2011); It requires the presence of at least one of the following clinical characteristics:

    a. Prolonged (>20 min) anginal pain at rest.

    b. New onset (de novo) angina (Class II or III of the Classification of the Canadian Cardiovascular Society).

    c. Recent destabilization of previously stable angina with at least Canadian Cardiovascular Society Class III angina characteristics (crescendo angina).

4. Coronary revascularization (percutaneous or surgical): The two main indications for percutaneous or surgical revascularization are:

    1) Patients with unstable angina or non-ST-segment elevation acute coronary syndrome.

    2) Patients considered likely to benefit from such surgery on the basis of the location and severity of chest pain, the number of vessels affected, and the presence of left ventricular dysfunction (Hamm et al, 2011).

5. Heart failure. Acute and chronic heart failure (HF) is a syndrome in which patients have typical symptoms and signs resulting from an abnormality of cardiac structure or function (McMurray JJ, et al. 2012; Yancy CW, et al. 2013). The cardinal manifestations of HF are dyspnea and fatigue, which may limit exercise tolerance, and fluid retention, which may lead to pulmonary and/or splanchnic congestion and/or peripheral edema.

    5.a. The diagnosis of HF with Reduced Ejection Fraction requires three conditions to be satisfied: 1. Symptoms typical of HF; 2. Signs typical of HF and 3. Reduced ejection fraction (< 40%)

    5.b. The diagnosis of HF with Preserved Ejection Fraction requires four conditions to be satisfied: 1. Symptoms typical of HF; 2. Signs typical of HF; 3. Normal or only mildly reduced left ventricular ejection fraction and non-dilated left ventricle; and 4. Relevant structural heart disease (left ventricular hypertrophy/left atrium enlargement) and/or diastolic dysfunction

5.c. A Heart Failure event may include hospitalization or an urgent outpatient visit. In this setting the event needs to meet ALL of the following criteria:

- The patient exhibits documented new or worsening symptoms of HF on presentation, including at least ONE of the following: Dyspnea, decreased exercise tolerance, fatigue or other symptoms of worsened end-organ perfusion or volume overload.
- The patient has objective evidence of new or worsening HF, consisting of at least TWO physical examination findings OR one physical examination finding and at least ONE laboratory criterion), including: Physical examination findings considered to be due to heart failure, including new or worsened peripheral edema, increasing abdominal distention or ascites (in the absence of primary hepatic disease), rales/crackles/crepitations at pulmonary auscultation, increased jugular venous pressure and/or hepatojugular reflux, S3 gallop, and clinically significant or rapid weight gain thought to be related to fluid retention
- Laboratory evidence of new or worsening HF, if obtained within 24 hours of presentation, including: Increased B-type natriuretic peptide (BNP)/ N-terminal pro-BNP (NT-proBNP) concentrations OR cardiological evidence of pulmonary congestion OR echocardiographic data of congestion or decreased cardiac output.
- The patient receives initiation or intensification of specific treatment for HF.

6. Peripheral artery disease. Ascertainment will be made according to the Inter-Society Consensus for the Management of Peripheral Arterial Disease (TASC II) (Norgren et al., 2007) and ESC Guidelines for the diagnosis of peripheral artery disease (Tendera M, et al., 2011). For participants with intermittent claudication, aged 60-69 with one cardiovascular risk factor, or aged ≥70 years, a resting ankle-brachial systolic pressure index ≤0.90 or an abnormal echo-Doppler examination, magnetic resonance imaging, or arteriography will be considered as diagnostic (confirmed case).
7. Venous thromboembolism (VTE): all VTE need to satisfy the standard diagnosis criteria for venous thrombosis or (thromb-) Pulmonary Embolism (PE) in the general population (see below 1-3). The diagnosis should be confirmed by objective imaging techniques (including echography, phlebography, pulmonary computed tomography angiography (angioCTA), NMR, etc.) and not only be based on the clinical suspicion.

Standard diagnosis criteria for VTE in clinical studies (Carrier M et al, 2012):

1. Deep venous thrombosis, defined as the loss of venous compressibility or the inability

of filling the deep vein intraluminal segment at the lower/upper limbs, as detected by echography with venous compression or phlebography, respectively.

- The presence of thrombus at the distal lower limb (distal from the popliteal vein) qualifies for primary VTE only if it is asymptomatic.
- All proximal thrombus qualify for final primary end-point if detected by imaging techniques (echography or radiology), regardless of whether it is or not asymptomatic.

2. Pulmonary Embolism (PE) is defined as:

Contrast pulmonary arteriography:

- Defects in intraluminal filling, as contrasted with two projections.
- Sudden stoppage of the contrast in one or several vessels with a diameter greater than 2.5 mm
- Pulmonary scintigraphy based on ventilation/perfusion (V/Q):

o A V/Q-pulmonary scintigraphy with high probability of PE in patients with no low clinical probability of PE.

- Pulmonary angiography using computed tomography:

o Defects in filling sub-segmental or more proximal vessels

3. Fatal PE is defined as:

- Death exclusively caused by PE and/or its confirmation at autopsy or using radiology techniques

Important considerations:

a) Superficial venous thrombophlebitis should not be described as VTE.

b) It is highly recommended to describe VTE according to the anatomic position:

- Lower limbs
- Upper limbs
- Pulmonary embolism
- Others: vessels at the splanchnic level, cerebral veins, etc.

c) The description of the VTE is highly convenient (for instance, distal to popliteal vein vs. proximal VTE; sub-segmental level vs. central PE)

d) VTE associated with a central catheter (for instance, deep venous thrombosis at the upper limbs) should be reported separately.

e) Incidental VTE should be differenced from any other symptomatic events.

8. Atrial fibrillation (AF): AF is defined following the Guidelines of the American College of Cardiology Foundation/American Heart Association Task Force on Practice Guidelines together with the European Society of Cardiology, the European Heart Rhythm Association, and the Heart Rhythm Society (Camm AJ, et al. 2010; Fuster V,

et al. 2011), as a cardiac arrhythmia with the following characteristics:

(1) The surface ECG shows 'absolutely' irregular RR intervals, i.e., RR intervals that do not follow a repetitive pattern.

(2) There are no distinct P waves on the surface ECG. Some apparently regular atrial electrical activity may be seen in some EKG leads, most often in lead V1.

(3) The atrial cycle length (when visible), i.e., the interval between two atrial activations, is usually variable and <200 ms (>300 bpm).

9. Type 2-diabetes. New-Onset Type 2 Diabetes cases are diagnosed following the recommendations of the American Diabetes Association:
   1. HbA1C ≥6.5%. This test should be performed in a laboratory using a method that is National Glycohemoglobin Standardization Program (NGSP) certified and standardized to the DCCT assay. OR
   2. Fasting plasma glucose (FPG) ≥126 mg/dL (7.0 mmol/L). Fasting is defined as no caloric intake for at least 8 hours OR
   3. Two-hour plasma glucose (PG) ≥ 200 mg/dL (11.1 mmol/L) during an oral glucose tolerance test (OGTT). This test should be performed as described by the WHO, using a glucose load containing the equivalent of 75 g anhydrous glucose dissolved in water OR
   4. In a patient with classic symptoms of hyperglycemia or hyperglycemic crisis, a random plasma glucose ≥200 mg/dL (11.1 mmol/L).

   In the absence of unequivocal hyperglycemia, results should be confirmed by repeat testing (American Diabetes Association, 2011).

10. Type-2 diabetes complications. Participants will be assessed yearly for microvascular complications of diabetes:
    1. Diabetic nephropathy: Kidney disease in diabetes is defined based on the alteration of glomerular filtration rate (GFR) and /or the presence of persistent albuminuria at levels of 30 mg/24 h or more (normal albumin excretion is currently defined as < 30 mg/24 h). GFR is estimated through a quantitative formula, the Modification of Diet in Renal Disease (MDRD) equation, that measures the progression of kidney involvement. Persistent albuminuria is determined by the urine albumin to creatinine ratio (normal <30 mg albumin/g creatinine) in a routine morning urine sample. Because of variability in urinary albumin excretion, two of three morning specimens collected within a 3- to 6-month period should be abnormal before considering a patient to have developed increased urinary

albumin excretion or a progression of albuminuria. The presence of one or two of the above criteria indicates renal disease in these patients and the requirement for appropriate follow-up for progression of renal disease (American Diabetes Association, 2011).

The stages of chronic kidney disease by GFR will be reported as follows:

1. Kidney damage with normal or increased GFR ≥90 mL/min/1.73 m^2 body surface area
2. Kidney damage* with mildly decreased GFR 60–89 mL/min/1.73 m^2 body surface area
3. Moderately decreased GFR 30–59 mL/min/1.73 m^2 body surface area

4 Severely decreased GFR 15–29 mL/min/1.73 m^2 body surface area

5. Kidney failure <15 mL/min/1.73 m^2 body surface area or dialysis

2. Diabetic retinopathy: Diagnosed by ophthalmologic examination and/or treatment with laser photocoagulation (American Diabetes Association, 2011).
3. Diabetic polyneuropathy: Diagnosed by clinical symptoms, neurological examination and results of electrophysiological studies of peripheral nerves (American Diabetes Association, 2011).

*Kidney damage defined as abnormalities in urine, blood, or imaging tests.

11. Cancer. All cancers except non-melanoma skin cancer will be considered. Cancer cases will be coded according to the International Classification of the World Health Organization (International Agency for Research in Cancer, WHO, 2014).

12. Dementia/Alzheimer's disease. Cases will be ascertained according to the Recommendations from the National Institute on Aging and the Alzheimer´s Association workgroup (McKhann et al., 2011) or if a diagnosis of dementia is reported by a neurologist.

13. Other dementias: Cases will be ascertained according to McKhann et al. 2011 criteria (see below) or if a diagnosis of dementia is reported by a neurologist.

Dementia is diagnosed when there are cognitive or behavioral (neuropsychiatric) symptoms that: 1. Interfere with the ability to function at work or at usual activities; and 2. Represent a decline from previous levels of cognitive functioning; and 3. Are not explained by delirium or major psychiatric disorder; 4. Cognitive impairment is detected and diagnosed through a combination of (1) history-taking from the patient

and a knowledgeable informant and (2) an objective cognitive assessment; 5. The cognitive or behavioral impairment involves a minimum of two domains).

14. Parkinson's disease. Cases will be ascertained according to the diagnostic criteria described by Hughes et al (Hughes AJ, et al., 1992) or if reported by a neurologist.

15. Unipolar depression. The diagnosis should be made according to the DSM-V criteria (American Psychiatric Association, 2013). In this, definition major depression, persistent depression, and other depressions included in Depressive Disorders (DSM V) are accepted. Diagnosis of depression made by primary care physicians or psychiatrist in participants treated with antidepressant drugs for more than 6 moths will be accepted. If this is the case, ICD 10 (International Statistical Classification of Diseases and Related Health Problems, 10th version) diagnosis of depressive episodes are also accepted. For physicians and psychiatrists not using ICD 10 or DSM V, a positive response to the two questions included in the NICE clinical guidelines is recommended (https://www.nice.org.uk).

16. Osteoporotic fractures. Low-energy fracture is defined as the fracture produced by a same-level fall. Fractures will be identified from X-rays reports obtained from at least two radiological reports. High trauma fractures, potentially pathological fractures (e.g., cancer or Paget's disease), or fractures of the head, fingers and toes will not be considered (Bliuc D, et al. 2009).

17. Gallstone disease or cholecystectomy: Gallstone disease will be diagnosis according to the findings obtained by imaging techniques including abdominal ultrasonography, computed tomography or magnetic resonance imaging. Diagnosis of cholecystectomy will require the corresponding surgical report.

18. Symptomatic gout: Defined following the criteria of the *American College of Rheumatology*. Typically the disease first presents as arthritis that is acute and episodic, but can be recurrent. Gout can also present as chronic arthritis of one or more joints. This clinical picture is built on a foundation of an excess body burden of uric acid, manifested in part by hyperuricemia, which is defined as serum uric acid levels greater than 7.0 mg/dL (Khanna D, et al. 2012).

19. Transient Ischemic Attack: The diagnosis should be made according to the Scientific Statement of the American Heart Association/American Stroke Association Stroke Council (Easton JD et al. 2009): a transient episode of neurological dysfunction caused by focal brain, spinal cord, or retinal ischemia, without acute infarction demonstrated by neuroimaging, preferably magnetic resonance imaging techniques.

20. Cataracts surgery: Defined by a medical report of cataracts surgery.

21. Surgery for obesity: Defined by a medical report of bariatric surgery.

9.3. Intermediate markers

Changes in nutrient intake and dietary patterns will be determined by changes in the 17-item score of adherence to the energy-restricted Mediterranean diet (intensive intervention group) or the 14-item score (control group) and by changes in food and nutrient intake determined by the 143-item food frequency questionnaire administered during follow-up.

Changes in systolic and diastolic blood pressure, serum lipid concentrations, fasting glucose levels, renal function, uric acid, hemoglobin A1C, C-reactive protein, and liver function will be evaluated yearly for the duration of the intervention.

Also evaluated yearly will be the percentage of participants in each group requiring anti-hypertensive, anti-diabetic or lipid-lowering medication, results of ECGs, cognitive function, quality of life, and psychological and neuropsychological questionnaire scores.

10. Randomization procedure (random assignment)

Between one and four weeks after the third screening visit, each recruiting center will randomly assign eligible candidates to one of two groups, intensive intervention group or usual care (control) group, using a centrally-controlled, computer-generated random-number system (available at: www.predimedplus.com). The coordinating center will be responsible for the randomization procedure by which participants will be randomly assigned with stratification by center, sex, and age group (<65, 65-70, >70 years). Married or unmarried couples are randomized together. The recruiting centers enter the participants' identification criteria into the internet-based system. The system then automatically assigns the participants or partners of participants to their groups.

Once this occurs, the group assigned cannot be changed. In the specific cases of couples in which the spouse was recruited at different times, the last spouse entering the study will be assigned (not randomised) to the same study arm than his/her partner in order to ensure high adherence to the intervention and avoid contamination and potential conflicts between partners of the same household.

11. Intervention protocol

All participants will continue to receive usual healthcare from their family doctors and primary care physicians throughout the duration of the trial. At no time will PREDIMED-PLUS personnel deliver medical care.

11.1. Phases of the study for participants assigned to the intensive intervention program with energy-restricted Mediterranean diet (intervention group)

First six months

In addition to the initial visit, participants assigned to the intensive intervention program will take part in six individual sessions (I) and 6 group sessions (G) in the first six months (see below):

**Month 1**		**Month 2**		**Month 3**		**Month 4**		**Month 5**		**Month 6**	
G	I	G	I	G	I	G	I	G	I	G	I

Participants will also receive a third monthly contact by way of a **telephone call** from a dietitian aimed at reinforcing the trial's objectives and answering any queries.

During these first six months, **participants in the intervention group** are encouraged to aim for a reduction in their initial weight of 10% and a reduction in their initial waist circumference of between 5 and 10%. The aim of the trial during these first six months is that the **average** weight loss of the participants in the intervention group is above 8% and the average waist circumference reduction is above 5%. Success in achieving an initial weight loss is known to be a predictor of long-term weight loss. For this reason, all participants will be given a chart on which to record and correctly monitor their own weight and waist circumference.

During this period they will be encouraged to substitute one meal for low-calorie foods and so will be offered a wide range of pleasant alternatives in keeping with the culinary traditions of the Mediterranean diet (see below).

Finally, if by the final visit the participant has still been unable to reach the objectives established for this phase of the trial (month 6), he or she will take part in a **motivational interview session** with the dietitian in order to determine why he/she has not reached his or her weight-loss goal (see below), try to readdress the situation, and provide appropriate rescue measures.

<u>Months 7-12</u>

Participants will attend one individual session (I) and one group session (G) every month in months 7 to 12 of the trial.

YEAR 1	Month 7		Month 8		Month 9		Month 10		Month 11		Month 12	
Months 7-12	G	I	G	I	G	I	G	I	G	I	G	I

They will also receive a third contact every month by way of a **telephone call** from a dietitian aimed at reinforcing the trial's objectives and answering any queries.

The first-year follow-up visit (see below) will coincide with the last individual visit for this phase.

<u>Years 2-6</u>

After the first year and in each of the remaining years of the trial (years 2-6), the participants will attend one quarterly individual session (I) and one monthly group session (G) and will receive two quarterly telephone calls (T), in accordance with the table below:

Month 13-72	Month 13		Month 14		Month 15		Month 16		Month 17		Month 18		Month 19		Month 20		Month 21		Month 22		Month 23		Month 24	
	G	T	G	T	G	I	G	T	G	T	G	I	G	T	G	T	G	I	G	T	G	T	G	I

Months 25-36 will follow the same procedure as months 13-24, and this procedure will be repeated for the successive years.

The annual follow-up visits (see below) will coincide with the last individual session of each year (month 24 above). Throughout the trial, any missed visits will be reprogrammed.

11.2. Program of individual and group sessions for participants assigned to the energy-restricted Mediterranean diet

A) Individual visits

All individual visits comprise:

i) Distribution of a 17-item questionnaire of adherence to an energy-restricted Mediterranean diet.

ii) Weight and waist circumference measurement by a dietitian.

iii) An individual motivational interview with the dietitian in accordance with the changes in weight observed and the participant's scores on the 17-item adherence to Mediterranean diet questionnaire (see below).

iv) Encouragement to self-monitor weight and waist circumference. Participants are provided charts for self-registering and self-monitoring weight and waist circumference in accordance with the Body Weight Simulator of the National Institute of Diabetes and Digestive and Kidney Diseases (Hall et al., 2011; National Institute of Diabetes and Digestive and Kidney Diseases, 2012). This simulator is also provided to participants at the first individual session with use instructions.

v) Personalized recommendations for increasing physical activity.

B) Group sessions

At these group sessions participants are provided shopping lists, menus, recipes, descriptions of typical components of the Mediterranean diet and advice on lifestyle changes. PREDIMED-PLUS dietitians lead these sessions, which are attended by no more than 20 participants. The sessions comprise:

i) An introductory talk to review the **17-item questionnaire** on adherence to the **energy-restricted Mediterranean diet** (see below).

ii) A 15-minute presentation of the main aspects of the **Mediterranean diet** with audiovisual material prepared by the coordinating center.

iii) Answers to any queries on any aspect of the intervention.

iv) Delivery of the following documents:

- Description of 4-5 low-calorie foods typical of a Mediterranean diet and adapted to the season.
- Weekly food shopping list adapted to the season.
- Weekly meal plan (with detailed menus) adapted to the shopping list.
- Recipes for the suggested menus.

v) Delivery of gratis virgin olive oil (one liter per month) and nuts (500 g per month) to each participant.

vi) At the end of the session participants are reminded of the date of the next session.

11.3. Program of individual and group sessions for participants assigned to the **control group**.

Control group participants receive usual medical care from medical staff at their health institutions. The importance of their attending usual medical visits will be stressed to them. Participants receive all the written information related to the **Mediterranean diet** used in the PREDIMIED trial as well as leaflets with general lifestyle recommendations for managing the metabolic syndrome. At the beginning of the study, a group session and an individual session is held at which dietitians deliver documents similar to those used in the PREDIMED trial (shopping lists, recipes, menus, and descriptions of Mediterranean diet components). The dietitians do not provide participants in the control group with instructions on how to lose weight, as this is the responsibility of their family doctors or specialists (usual care). They are also offered a group session every 6 months. At the initial visit and at each 6-month group session, participants are provided free virgin olive oil (6 liters every 6 months) and nuts (3 kg every 6 months). In order to encourage compliance with the trial, supply of olive oil and nuts to the participants is contingent on their attending these sessions. The 6-month group sessions include tips on how to follow the **Mediterranean diet** to prevent CVD but advice on calorie restriction, weight loss or increased physical activity is not given and no such objectives are entertained.

11.4. Dietary and lifestyle intervention

The **Intervention Committee** led by Jordi Salas-Salvadó coordinates the dietary and lifestyle intervention. This committee is made up of four coordinators (Jordi Salas-Salvadó, Montse Fitó, Ramón Estruch and Miguel Ángel Martínez-Gonzalez), three of whom will be responsible for the three intervention sub-committees: Dietary Intervention (chair: Jordi Salas-Salvadó; members: Nancy Babio, Emilio Ros and Ana Sánchez-Tainta); Physical Activity (chair: Montse Fitó; members: Helmut Schröder, Ascensión Marcos, Miguel A. Martínez-González, Dolores Corella, and Julia Warnberg); and Behavior Treatment (chair: Ramon Estruch; members: Fernando Fernández-Aranda, Cristina Botella and Jordi Salas-Salvadó). This Committee is responsible for designing the lifestyle intervention program for the intensive intervention group and ensuring that it is implemented correctly. Miguel Ruiz-Canela, Miguel A. Martínez-González and Jordi Salas-Salvadó are responsible for ethical considerations.

<u>Dietary recommendations</u>

Many aspects of a diet's quality can affect body weight and the risk of obesity-related illnesses to a greater extent than relative macronutrient content (Mozaffarian et al., 2011; Ludwig, 2012). In recommendations given to participants, two food groups (A and B) will be clearly differentiated:

A) Traditional dietary patterns based on whole foods or minimally processed foods, such as the **Mediterranean diet**, which incorporates many cardioprotective foods and few harmful ones. The consumption of virgin olive oil, nuts (especially walnuts), fruits and vegetables, salads, whole grains, fiber-rich foods and low-fat yogurts have been consistently associated with weight loss or lower weight gain (Martinez-Gonzalez, Bes-Rastrollo, 2011; Mozaffarian et al., 2011).

B) On the other hand, sugar-sweetened beverages, fast foods, refined grain products (especially white bread, which is widely consumed in Spain), white rice, pasta (except for whole-grain pasta), French fries, potatoes, trans fats (mainly present in commercial bakery products in Spain), sweets, cakes, pies, sugar, precooked meals, sausages or cold cuts of processed meats, and patés have been consistently associated with weight gain (Schulze et al., 2006; Mozaffarian et al., 2011).

The main focus of the intensive intervention program (intervention group) lies therefore in the diet's overall quality, with the aim of avoiding foods from the B group and replacing them with foods from the A group.

In addition, by taking into account energy requirements estimates according to the Institute of Medicine equation as well as the participants' basal metabolic rate and level of physical activity, a reduction in energy intake of roughly 600 kcal (about 30% of estimated energy requirements) is envisaged. The energy-restricted Mediterranean diet involves reduced consumption of meat and cold cuts, sugars, white bread, processed fruit juices and sugary beverages, and other foods from the B group, as follows:

**ENERGY-RESTRICTED MEDITERRANEAN DIET**	
**NUTRIENT**	**RECOMMENDED INTAKE**
Calories[1]	Reduction of ≈600 kcal/day (about 30%) from usual intake
Total fat[2]	35-40 % of total calories
Saturated Fatty Acids	8-10 % of total calories
Monounsaturated Fatty Acids	> 20 % of total calories
Polyunsaturated Fatty Acids	> 10 % of total calories
Cholesterol[3]	< 300 mg/day
Proteins[4]	Approximately **20** % of total calories
Carbohydrates[5]	**40-45** % or more of total calories (of low glycemic

	index)
Sodium chloride	No more than 100 mmol/day (roughly 2.4 g of sodium or roughly 6 g of sodium chloride)
Dietary fiber	**30-35** g/day

1. A reduction in calories of 500 to 1,000 kcal/ day will help to achieve a weight loss of 0.5 to 1 kg/week.

Alcohol provides unnecessary calories and displaces the intake of more nutrient-dense foods. Not only does the consumption of alcohol increase the number of calories in one's diet but in epidemiological and experimental studies it has also been associated with obesity. For this reason, although the 17-item adherence to the Mediterranean diet questionnaire contains one item for the consumption of wine, the impact of calories from alcohol on the overall calorie intake should be carefully evaluated and monitored and the consumption of alcoholic beverages other than wine should be avoided.

2. The consumption of wine permitted is one or two glasses per day for women and two or three glasses per day for men. The consumption of other sources of alcohol other than wine is discouraged. Red wine is preferred over other types and it is recommended that the wine be consumed at mealtimes.

3. Fat restriction involves fat from animal foods. Olive oil and nuts must be the preferred sources of fat.

4. Proteins should be derived first from plant and second from lean animal sources (like fish or poultry).

5. Carbohydrates should be derived from solid, minimally processed and fiber-rich foods with a low glycemic index, such as vegetables, fruits and whole grains, all of which are good sources of vitamins, minerals, and fiber. A diet that is rich in soluble fiber such as oat bran, legumes, and most fruits and vegetables may be effective in reducing blood cholesterol levels and insulin resistance. A diet that is high in all types of fiber may also help to control weight by promoting satiety and maintaining lower levels of total energy intake.

6. During weight loss, attention should be given to maintaining an adequate intake of vitamins and minerals. Maintaining the recommended calcium intake of 1,000 to 1,500 mg/day is especially important for postmenopausal women who may be at risk of osteoporosis.

Participants in the intensive intervention group receive counseling to help them progressively increase their compliance with the following 17 objectives (the **17-item questionnaire on adherence to the energy-restricted Mediterranean diet**). **One point** will be awarded for **each objective met**:

1. Use only extra-virgin olive oil for cooking, salad dressings, and spreads.
2. Consume ≥3 portions of fruit per day.
3. Consume ≥ 2 portions of vegetables/garden produce per day (at least 1 portion raw or in a salad).
4. Reduce consumption of white bread to ≤1 serving/day (1 serving = 75 g).
5. Consume whole grain cereals and pasta ≥5 times per week.
6. Consume ≤1 serving (1 serving = 100-150 g) of red meat, hamburgers, or meat products (ham, sausage, etc.) per week.
7. Consume less than 1 serving of butter or cream per week (1 serving = 12 g).
8. Consume less than one sugary beverage or sugar-sweetened fruit juice per week.
9. Consume ≥3 servings of legumes per week (1 serving = 150 g).
10. Consume ≥3 servings of fish or shellfish per week (1 serving = 100-150 g fish, or 4-5 units or 200 g shellfish).
11. Consume < 3 sweets or pastries, such as cakes, cookies, sponge cake, or custard, per week.
12. Consume ≥ 3 servings of nuts (including peanuts) per week (1 serving = 30 g).
13. Consume chicken, turkey or rabbit meat instead of beef, pork, hamburgers or sausages.
14. Use *sofrito* (sauce made with tomato and onion, leek or garlic, simmered in olive oil) ≥ 2 times per week.
15. Do not add sugar to beverages (coffee, tea); instead, replace sugar with non-caloric artificial sweeteners.
16. Reduce consumption of pasta or rice <3 servings per week (unless the pasta or rice are whole grain products).
17. Consume 2-3 glasses of wine (200 mL) per day (men) or 1-2 glasses of wine per day (women). This item will be only promoted among participants who were consumers of alcohol at baseline. Abstainers will never be invited to start consuming wine or any other alcoholic beverage.

The intervention tool for the control group, on the other hand, is the PREDIMED 14-item adherence questionnaire to the non-energy-restricted Mediterranean diet

(Schroeder et al., 2011). However, the 17-item questionnaire is also collected in control group participants for comparison purposes.

Physical exercise recommendations

Participants are encouraged to gradually increase their level of physical activity to at least 45 minutes per day (6 days per week) after 6 months of intervention and their progress is monitored. The physical activity program includes aerobic activities, such as gentle walking or any equivalent activity of moderate intensity and resistance training (Fernández et al., 2012). The dietitians adapt their recommendations to personal preferences and encourage participants to switch between activities with the same metabolic equivalence of tasks.

Psycho-behavioral therapy

Participants are instructed on strategies and provided tools for solving problems associated with consuming high calorie foods and performing sedentary activities. They are encouraged to learn how to recognize lack of control on food intake under stressful or anxious situations and how to exercise self-control.

Recommendations on the use of tobacco

The PREDIMED-PLUS dietitians will make no recommendations on the use of tobacco. This is the responsibility of the medical professionals in the primary care centers in accordance with usual medical practice.

Individual motivational interviews

Personal interviews with the dietitian at each individual visit are adapted to the participant's clinical conditions, preferences and beliefs. Dietary changes are introduced in order to achieve the recommended diet for each participant and suitable lifestyle changes are incorporated. Objectives are accorded via a negotiated agreement between the two parties (dietitian and participant) depending on what participants consider to be an attainable goal. The main objective is to change not only the participant's consumption of certain foods but also his or her overall dietary pattern. Attention can vary between changing portion sizes, changing the frequency of dietary components, and changing cooking methods.

Achievements made in the previous months, however minor, are always considered an essential support mechanism for improving self-esteem and self-reward. Special care

is taken to ensure that participants do not receive contradictory dietary advice from health professionals external to the PREDIMED-PLUS trial.

As described, each participant receives oral and written information on the food components and culinary customs of the **energy–restricted Mediterranean diet**, as well as charts for self-registering and self-monitoring changes in weight and waist circumference at each visit.

Participants who during the active weight-loss phase have observed a lower weight loss than expected or who have not maintained the weight loss they had achieved receive special reinforcement and a series of rescue measures to help them achieve weight loss and weight-loss maintenance. In such cases, agreements are negotiated between the dietitians and the participants.

Role of the dietitians

The PREDIMED-PLUS dietitians are directly responsible for the dietary intervention. They have been specifically trained and certified to deliver the PREDIMED-PLUS intervention protocol. All intervention procedures are conducted in accordance with the PREDIMED-PLUS operation's manual. Throughout the study, annual meetings will be held at which the dietitians will discuss any problems they may have identified and find possible solutions. The dietitians and trial coordinators will discuss any problems arising during the trial, thus ensuring a process of continuous feedback.

12. Training and calibration procedures

A general trial operations manual and staff training documents are set forth to ensure standardized procedures across the various recruiting centers. Before implementation of the protocol, study personnel attended a 3-day training course at the coordinating center. This included theoretical and practical group discussions with experts on lifestyle interventions in order to convey the goals of the study, develop all the specific aspects involved in implementing the intervention, and impart training on the informed-consent process, anthropometric and blood pressure measurements, data collection by optical scanning or online systems, and biological sample collection and processing. The abilities of all contracted personnel were evaluated at personal interviews during this training course. The research team stressed the importance of creating a trusting and empathic relationship with the participants and paying attention to their individual needs in order to maximize their motivation and retention into the trial. Study personnel keep a copy of the operations manual detailing all the training points. In addition, all the personnel responsible for the intervention will attend annual meetings and be in constant

contact with the principal investigators in order to ensure standardized implementation of the trial protocol. In accordance with the protocol, all scales and other measurement instruments will be periodically calibrated.

13. Retention and compliance with strategies and supervision procedures

The recruitment of participants and the **compliance rates for the intervention strategies** are crucial to the success of a trial of this nature. For this reason, a run-in (pre-evaluation) period **prior to randomization** was planned: only participants who adhere to **all** the requirements of the protocol during the run-in period are accepted into the trial. The lag time between the end of the run-in period and the start of the intervention ranges from one week to one month.

The researchers involved in this trial have already gained invaluable experience in managing long-term trials through the PREDIMED trial, wherein they developed strategies for ensuring participants' compliance with the protocol and encouraging their **long-term retention**. We understand that this is a particularly sensitive aspect for participants in the control group. Therefore, at each group session of both the intensive intervention group and the usual care group, virgin olive oil and mixed nuts is provided at no cost to all participants. Our experience in the PREDIMED trial showed that such gifts, especially the virgin olive oil, greatly helped to encourage participants' retention. Other retention strategies include providing feedback on findings during follow-up to the participants' usual health-care providers as well as supplying other non-coercive material incentives for both groups. Additionally, in the intensive intervention group, where significant weight-loss is anticipated, contact with participants is ongoing and flexible interventions and rescue measures tailored to the participants' needs are implemented. Self-control, self-reward and self-monitoring techniques will also reinforce participants' compliance with the intervention. Finally, the intervention is adapted to the needs of the participants, which should encourage compliance.

14. Biological samples and laboratory procedures

The nursing staff contracted at each recruiting center is responsible for collecting, processing and storing the biological samples in freezers at a temperature of -80ºC. Blood samples are collected at the recruiting centers in the same way as they are collected at the participants' usual healthcare centers. In addition, 55.5 ml of blood are extracted and collected in the following tubes: two 10 ml K2E EDTA tubes; one 4.5 ml citrate tube; and two 10 ml and 6 ml gel serum separator tubes. The serum, citrate plasma and EDTA plasma samples are distributed in aliquots of 200 µl and 500 µl and

stored at -80°C for future analyses at the recruiting centers. For the intensive intervention group, the biochemical measurements will be performed in a blind fashion and in the same batch for consecutive samples of each participant. Each recruiting center has an ultra-low-temperature freezer with enough capacity to store biological samples until final delivery. All biological samples are processed at each recruiting center no later than one hour after extraction. During transportation from the primary care centers to the laboratories, the biological samples are stored at 4ºC in a portable cooler. Urine tests are conducted at the recruiting centers in the same way they are conducted at the participants' usual healthcare centers in accordance with the specific PREDIMED-PLUS protocol for collecting biological samples.

15. Quality control

The general database for the PREDIMED-PLUS trial will be managed and maintained by the research group of the IMIM Institute (CIBERobn). The food-frequency questionnaires and the food records, as well as quality of life test, clok and Minimental tests are processed and managed at the University of Navarra. Data collected from accelerometers to measure physical activity are processed at the Malaga recruiting center in collaboration with the CSIC/UAM. These data are sent every three months to the IMIM, where they are incorporated into the General Database. Event detection data, collected from information gathered during the intervention and at the follow-up visits, will be introduced into specific forms at the recruiting centers, preferably using online systems, and sent at least once a month to the data manager at the IMIM, who will send monthly reports of missing or inappropriate entries back to the recruiting center coordinators to solve any raised queries. The IMIM also sends monthly reports to the different recruitment centers with the sumsample of each node. The steering committee has been set up to ensure the quality of the project and correct any flaws or divergences. This committee is made up of Jordi Salas-Salvadó (PREDIMED-Plus coordinator), Miguel Angel Martínez-González, Ramón Estruch, Montserrat Fitó, Emilio Ros, and Dolores Corella. At every annual PREDIMED-PLUS meeting, the IMIM will conduct a current data management information session. An annual summary will be sent to the recruiting center coordinator for distribution to all groups.

To reduce data entry expenses and speed up processing, the questionnaires and data forms are processed by optical scanning or by online data transfer forms. The data forms are entered in duplicate and missing data checks are performed. All forms sent to another recruiting center must be photocopied and stored at that center. After data entry, cross-form edit checks are performed and any data inconsistencies are identified. To

detect any still-unsolved problems, audits will be run periodically at each recruiting center. Reports will be drafted to summarize any problems in the database and provide an additional step to ensure the quality and accuracy of the data. To minimize the possibility of error, a detailed operations manual has been prepared.

Annual staff training meetings will be conducted. The data manager and an audit committee will evaluate the performance of each recruiting center. Appropriate new procedures and corrective measures will be implemented whenever deficiencies are noted. Until the end of the trial, all field centers will be masked to the trial outcome data except for the two trial statisticians, one in Navarra (M.A. Martínez-González) and one at the IMIM in Barcelona (Joan Vila), who will always perform the statistical analyses in duplicate with two statistical analysis units. Because of the nature of the trial, however, the dietitians at each field center know which intervention has been assigned to each participant. The medical doctors who will prepare the annual report on the *ad hoc* review of the participants' medical records will be blinded to group assignment, as will the **Clinical Event Ascertainment Committee**. The members of the Steering Committee, who will attend the meetings of the Data and Safety Monitoring Board, will also remain blinded to the results of intermediate analyses throughout the trial. The Steering Committee will be informed of the total number of events observed but not of the groups in which they occurred.

STATISTICAL ANALYSIS PLAN

All analyses will be performed on an intention-to-treat basis. Miguel A. Martínez-González will be the senior statistician responsible for the statistical analysis plan. All major data analyses will be conducted under his supervision. Statistical analyses for the main aims of the study will be also conducted in duplicate by the center at IMIM, Barcelona (responsible statistician at IMIM: Joan Vila).

1. Analysis of the effect of the intervention

Since the data take into account time to the event, Cox's regression models will be used to determine the effect of the intervention on the incidence of cardiovascular events. For changes in weight and waist circumference, mixed models of analysis of variance and generalized estimating equations (GEE) will be used. These models will include the following adjustment covariates:

1) All factors that, according to the scientific literature, are related to the event; and
2) All factors that reach statistical significance in univariate analyses.

In these models we will evaluate: 1) the proportional hazards assumption; 2) the linearity of the continuous variables, using smoothing spline methods; 3) the effect of extreme observations on the estimation of parameters, by calculating delta-beta values. The use of further approaches (i.e. normalizing transformations, stratified analyses, etc.) will depend on the results obtained above. Given that participants will be clustered by recruiting centers, some degree of correlation structure may be expected. Center will therefore be included as a stratification variable, including frailty estimates, in the Cox regression models. The goodness-of-fit of the models will be examined using the modified Hosmer-Lemeshow test for survival studies. Robust estimators of variance that account for the clustering effect of members of the same household (the second member is not randomized for feasibility reasons) will be used to take into account the intra-cluster correlation. Sensitivity analyses will be conducted after excluding the second (non-randomized) members of the same household. In addition to the stratification by center, all Cox models will be also stratified by sex and educational level.

2. Interim analyses and stopping rules

Data from the PREDIMED-PLUS trial will be analyzed after 3 years of median follow-up, after 5 years of median follow-up, and at the end of the trial. For methodological reasons but especially for ethical ones, suitable follow-up for a trial must include at least one interim analysis (Schulz, Grimes, 2005). However, to preserve an overall alpha error of

0.05, interim analyses have to be penalized. We will use the O'Brien and Fleming boundaries (O'Brien and Fleming, 1979). With this method, the boundaries are stricter at the earlier stages of the study than at the later ones. Applying this rule leads to the following p values for stopping the trial:
First interim analysis (median follow-up: 3 years); threshold p value: 0.0005.
Second interim analysis (median follow-up: 5 years); p value: 0.014.
Final analysis (median follow-up: 8 years); p value: 0.045

These p values should not be considered compulsory for stopping the trial but guidelines for guaranteeing the security of the data. In making their decision, the Data and Safety Monitoring Board must take into account, for example, the size of the effect, the follow-up time at each recruiting center, the heterogeneity between the effects at the recruiting centers, as well as evidence from other current trials and observational studies. All the above must be taken into consideration when deciding either to continue or to interrupt the trial after each interim analysis. Reasons for interrupting the trial include: 1) convincing evidence of the beneficial effect of the intervention (the trial will be stopped only if the effect of the intervention is great); 2) convincing evidence of a harmful effect from the intervention; 3) results suggesting it is highly unlikely that the proposed hypothesis will be accepted due to, for example, a very small effect of the intervention that dramatically affects the trial's statistical power.

3. Estimations of sample size

We will determine the effect of the intensive weight-loss lifestyle intervention with an energy-restricted **Mediterranean diet** on the two primary outcomes below, assuming a two-tailed alpha error of 0.05.

1. Effect of the intervention on the **incident CVD** (non-fatal myocardial infarction, non-fatal stroke, and cardiovascular death). The cumulative projected incidence after including as primary events all non-fatal acute myocardial infarctions and all microinfarctions with positive high-sensitivity troponin tests after 6 years will be at least 10% in the control group, if we take into account the results of the PREDIMED trial after 4.8 years (which did not include high-sensitivity troponin tests). The hazard ratio (HR) for the combined primary endpoint is anticipated to be 0.70 (Estruch et al., 2013) and will probably be even lower (greater protective effect) if we consider that in the PREDIMED trial no energy restriction was implemented, physical activity was not encouraged, and weight loss was not a target of the intervention. Under these assumptions, therefore,

even if the dropout rates were to reach 20%, the required sample size would be 2,400 per group (see Figure 3). To be conservative, however, we aim to recruit 6,000 participants and assign 3,000 participants to each group. The participants will be recruited at 20 recruiting centers, each of which will have the goal of recruiting, educating and following approximately 300 participants, 150 of whom will be in the control group and 150 in the intensive intervention group.

FIGURE 3. Estimation of the sample size required per intervention group in the PREDIMED-PLUS trial

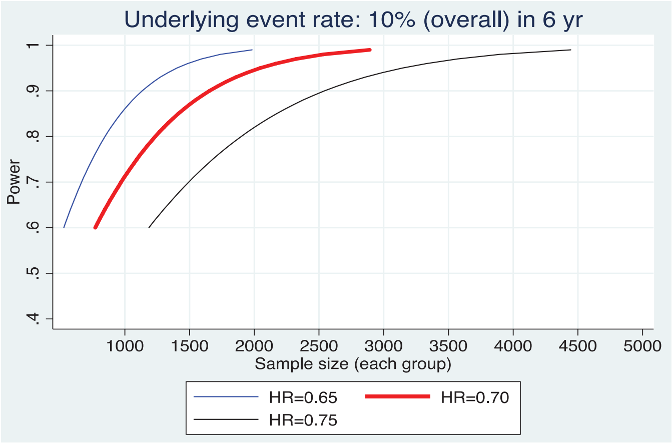

2. Effect of the intervention on **weight change**. Based on previous studies, we can expect a minimum weight change for participants in the control group and a weight loss of 3-4.5 kg for those in the intensive lifestyle intervention group, with a standard deviation of 8 kg (Shai et al., 2008; Sacks et al., 2009; Wing, 2010). If we assume our intervention will have only a small effect on weight change and then calculate sample size according to a weight change of 1 kg in the usual care group, a weight change of 3 kg in the intensive lifestyle intervention group, and a standard deviation of 8 kg, in order to achieve a statistical power of 0.80 we would need a sample size of only 337 in each group. Since the number of participants to be recruited is much higher than this figure, the statistical power needed to reach this objective is largely guaranteed.

STRENGTHS AND LIMITATIONS

1. Strengths

A. This trial provides a multidisciplinary approach to tackling the serious problem presented by the overweight and obesity epidemic. Our target group comprises obese or overweight adults, who represent an increasing proportion of the general population. For these subjects, an intervention based on a profound lifestyle change incorporating improvements in the dietary pattern, weight loss, behavioral therapy, and increased physical activity can be a novel and useful model for reducing the burden of obesity and associated diseases, thus contributing to the sustainability of the healthcare system. The trial clearly addresses priority objectives of the public healthcare system since it tackles both the principal epidemic of our times (overweight and obesity) and the principal cause of death around the world (CVD).

B. This innovative proposal presents a novel paradigm for nutritional recommendations aimed at achieving weight loss, i.e., a traditional dietary pattern characterized by a moderate-to-high fat content. We believe this new approach will help improve compliance with the intervention and overcome the main challenge of any dietary interventions aimed at fighting overweight and obesity: long-term weight loss maintenance (Shai et al., 2008; Beunza et al., 2010; Romaguera et al., 2010).

C. The intervention is well structured and the trial is suitably designed for determining the effect of the intervention on the main clinical outcomes. Moreover, since the trial is conducted in the context of primary healthcare and incorporates epidemiological, clinical and basic aspects, it has a high capacity for both transferability and reproducibility.

D. The research team includes investigators with invaluable experience in lifestyle intervention trials (e.g., PREDIMED). As these investigators come from a wide range of fields, their work will be complementary and the trial's chances of success will be enhanced. All these reasons, together with the success and achievements of the PREDIMED trial, attest to the viability of this trial proposal.

E. From a strategic perspective, this is a timely proposal since it provides continuity for the collaborative project in which most CIBEROBN centers have participated, i.e., PREDIMED.

2. Limitations

A. Our study will enroll participants aged between 55 and 75 years old. This may preclude generalization to younger age groups.

B. In a large-scale clinical trial, one limitation to consider is participants' dropout rates. However, we hope to ensure compliance in both groups by: a) providing free foods (olive

oil and mixed nuts); b) establishing personal relationships with each participant via individual and group sessions; c) administering, at the start of the study, the Prochaska and DiClemente Stages of Change Model, by which a low predicted probability of changing dietary habits will be a criterion for exclusion; and d) establishing, at the start of the study, a one-month run-in period in order to identify and select participants with a greater likelihood of compliance with the protocol and retention into the study (see section 5 and 13).

C. Homogeneity of the interventions is difficult because it is based on three components: diet, physical exercise, and behavior. For this reason we have developed a detailed protocol for implementing the intervention and have established a committee for each intervention component. We also conducted a staff training session at the beginning of the study and will conduct annual follow-up sessions throughout.

COMMITTEES AND GOVERNANCE

The PREDIMED-PLUS **Executive Committee** includes the principal investigators from all the participating centers (see Annex 1). It will provide scientific and strategic orientation for decision-making and will be responsible for designing, implementing and publishing the study's protocol and guaranteeing the quality of its implementation and management. It will determine its own guidelines and approve the criteria and guidelines of the other committees within the study. It will convene at least twice a year to discuss and report on the study's progress.

The **Steering Committee**, made up of Jordi Salas Salvadó (Chair), Miguel Angel Martínez-González (PI of the ERC-Advanced Research Grant), Ramón Estruch, Montserrat Fitó, Emilio Ros, and Dolores Corella, is responsible for ensuring the quality of the project and correcting any flaws or divergences that may be detected.

Data Safety and Monitoring Board

To ensure the smooth running of the trial and the safety of participants, an Independent **Data Safety and Monitoring Board** has been set up. This Board is made up of: Chairman, Meir J. Stampfer (Harvard School of Public Health); members Joan Sabaté (Loma Linda University), Arne Astrup (Copenhagen University), Francisco Fernandez-Avilés (Universidad Complutense of Madrid) and Xavier Pi-Sunyer (Columbia University). The Board will convene at least once a year to review the implementation of the protocol and monitor the trial's progress. It will examine the competence of each recruiting center, evaluate their compliance with the study's objectives, and decide whether they may continue in the trial.

In addition, a report will be mailed periodically by the PREDIMED-PLUS Steering Committee to the Board members with relevant statistical analyses for judging on the continuation of the PREDIMED-PLUS trial. Throughout the study, the Board members can request any statistical analysis on a blinded or unblinded basis. The Board may recommend termination of the trial at any time if an unacceptable incidence of adverse events or significant differences in mortality between study groups are observed. The Executive Committee of the PREDIMED-PLUS trial, however, will make the final decision.

SOURCES OF FUNDING AND ADMINISTRATIVE ISSUES

This study was supported by the official funding agency for biomedical research of the Spanish government, Instituto de Salud Carlos III (ISCIII) through the Fondo de Investigación para la Salud (FIS) (grant to Jordi Salas-Salvadó) and CIBEROBN, which is co-funded by the European Regional Development Fund, and by the European Research Council (Advanced Research Grant 2013-2018; 340918) grant to Miguel Ángel Martínez-Gonzalez.

Trial's website

http://www.predimedplus.com

Contact's name and address

Jordi Salas-Salvadó, MD, PhD. Human Nutrition Unit. Faculty of Medicine and Health Sciences, Universitat Rovira i Virgili, C/ Sant Llorenç, 21, 43201 Reus (Spain). Telephone number: +34 977759312; Fax number: +34 977759322. E-mail: jordi.salas@urv.cat.

Prof. Miguel A. Martínez-González, MD, PhD, MPH, Department of Preventive Medicine and Public Health, Facultad de Medicina–Clínica Universidad de Navarra, Irunlarrea 1, 31008 Pamplona, Spain. Phone: +34 636 355 333, E-mail: mamartinez@unav.es

PREDIMED-PLUS Registration

The PREDIMED--Plus trial was registered at the International Standard Randomized Controlled Trial (ISRCT; http://www.isrctn.com/ISRCTN89898870) with number 89898870 and a registration date of 24 July 2014.

REFERENCES

American Diabetes Association. Standards of medical care in diabetes-2011. Diabetes Care 2011;37 Suppl 1:S11-61.

Alberti KG, Eckel RH, Grundy SM, et al. Harmonizing the metabolic syndrome: a joint interim statement of the International Diabetes Federation Task Force on Epidemiology and Prevention; National Heart, Lung, and Blood Institute; American Heart Association; World Heart Federation; International Atherosclerosis Society; and International Association for the Study of Obesity. Circulation 2009;120:1640-5.

Alonso J, Prieto L, Anto JM. [The Spanish version of the SF-36 Health Survey (the SF-36 health questionnaire): an instrument for measuring clinical results]. Med Clin (Barc) 1995;104:771-6.

Alonso J, Regidor E, Barrio G, Prieto L, Rodriguez C, de la Fuente L. [Population reference values of the Spanish version of the Health Questionnaire SF-36]. Med Clin (Barc) 1998;111: 410-6.

American Dietetic Association. Adult Weight Management Evidence-Based Nutrition Practice Guideline. Ver http://www.adaevidencelibrary.com/ topic.cfm?cat=2798 &auth=1, consulted 12/4/2012.

American Psychiatry Association. Diagnostic and Statistical Manual of Mental Disorders, Fifth Edition (DSM-5) Arlington, VA, American Psychiatric Association, 2013.

Bao Y, Han J, Hu FB, et al. Association of nut consumption with total and cause-specific mortality. N Engl J Med 2013;369:2001-11.

Basterra-Gortari FJ, Beunza JJ, Bes-Rastrollo M, Toledo E, García-López M, Martínez-González MA. [Increasing trend in the prevalence of morbid obesity in Spain: from 1.8 to 6.1 per thousand in 14 years]. Rev Esp Cardiol 2011;64:424-6.

Benton AL, Hamsher, K, Sivan AB. Multilingual Aphasia Examination (3rd ed.). San Antonio, Texas: Psychological Corporation; 1994.

Berrington de Gonzalez A, Hartge P, Cerhan JR, et al. Body-mass index and mortality among 1.46 million white adults. N Engl J Med 2010;363:2211-9.

Bes-Rastrollo M, Sabaté J, Gómez-Gracia E, Alonso A, Martínez JA, Martínez-González MA. Nut consumption and weight gain in a Mediterranean cohort: The SUN study. Obesity (Silver Spring) 2007;15:107-16.

Beunza JJ, Toledo E, Hu FB, et al. Adherence to the Mediterranean diet, long-term weight change, and incident overweight or obesity: the Seguimiento Universidad de Navarra (SUN) cohort. Am J Clin Nutr 2010;92:1484-93.

Benkhalti F, Prost J, Paz E, Perez-Jimenez F, El Modafar C & El Boustani E. Effects of feeding virgin olive oil or their polyphenols on lipid of rat liver. Nutrition Research 2002; 22:1067-1075.

Beck A T, Steer R A, y Brown GK. BDI-II. Beck Depression Inventory-Second Edition Manual. San Antonio, TX: The Psychological Corporation, 1996.

Bueno NB, de Melo IS, de Oliveira SL, da Rocha Ataide T. Very-low-carbohydrate ketogenic diet v. low-fat diet for long-term weight loss: a meta-analysis of randomised controlled trials. Br J Nutr 2013;110:1178-87.

Buxton AE, Calkins H, Callans DJ,et al. ACC/AHA/HRS 2006 key data elements and definitions for electrophysiological studies and procedures: a report of the American College of Cardiology/American Heart Association Task Force on Clinical Data Standards (ACC/AHA/HRS Writing Committee to Develop Data Standards on Electrophysiology). Circulation 2006;114:2534-70.

Bliuc D, Nguyen ND, Milch VE, Nguyen TV, Eisman JA, Center JR. Mortality risk associated with low-trauma osteoporotic fracture and subsequent fracture in men and women. JAMA 2009;301:513-21.

Bray F, Znaor A, Cueva P, et al. Planning and Developing Population-Based Cancer Registration in Low- and Middle-Income Settings. IARC Technical Publication No. 43. International Agency for Research on Cancer. WHO, Geneva 2014.

Camargo A, Delgado-Lista J, Garcia-Rios A, et al. Expression of proinflammatory, proatherogenic genes is reduced by the Mediterranean diet in elderly people. Br J Nutr 2012;108:500-8.

Camm AJ, Kirchhof P, Lip GY, et al. ESC Committee for Practice Guidelines. Guidelines for the management of atrial fibrillation: the Task Force for the Management of Atrial Fibrillation of the European Society of Cardiology (ESC). European Heart Rhythm Association; European Association for Cardio-Thoracic Surgery, Europace. 2010;10:1360-420.

Carrier M, Khorana AA, Zwicker JI, Lyman GH, Le Gal G, Lee AY; subcommittee on Haemostasis and Malignancy for the SSC of the ISTH. Venous thromboembolism in cancer clinical trials: recommendation for standardized reporting and analysis. J Thromb Haemost 2012;10:2599-601.

Chrysohoou C, Panagiotakos DB, Pitsavos C, Das UN, Stefanadis C. Adherence to the Mediterranean diet attenuates inflammation and coagulation process in healthy adults: The ATTICA Study. J Am Coll Cardiol 2004;44:152-8.

Chrysohoou C, Skoumas J, Pitsavos C, et al. Long-term adherence to the Mediterranean diet reduces the prevalence of hyperuricaemia in elderly individuals, without known cardiovascular disease: the Ikaria study. Maturitas 2011;70:58-64.

Covas MI, Konstantinidou V, Fito M. Olive oil and cardiovascular health. J Cardiovasc Pharmacol 2009;54:477-482.

Covas MI, Nyyssonen K, Poulsen HE, et al. The effect of polyphenols in olive oil on heart disease risk factors: a randomized trial. Ann Intern Med 2006;145: 333-41.

Dai J, Jones DP, Goldberg J, et al. Association between adherence to the Mediterranean diet and oxidative stress. Am J Clin Nutr 2008;88:1364-70.

de Roos B, Zhang X, Rodriguez Gutierrez G, et al. Anti-platelet effects of olive oil extract: in vitro functional and proteomic studies. Eur J Nutr 2011;50: 553-62.

Defilippis AP, Blaha MJ, Jacobson TA. Omega-3 Fatty acids for cardiovascular disease prevention. Curr Treat Options Cardiovasc Med 2010;12:365-80.

Detopoulou P, Panagiotakos DB, Chrysohoou C, et al. Dietary antioxidant capacity and concentration of adiponectin in apparently healthy adults: the ATTICA study. Eur J Clin Nutr 2010; 64:161-8.

DSM-IV-TR; APA, 2000. American Psychiatric Association. (2000). Diagnostic and statistical manual of mental disorders (4th ed., text rev.). Washington, DC.

Easton JD, Saver JL, Albers GW, et al. Definition and evaluation of transient ischemic attack: a scientific statement for healthcare professionals from the American Heart Association/American Stroke Association Stroke Council; Council on Cardiovascular Surgery and Anesthesia; Council on Cardiovascular Radiology and Intervention; Council on Cardiovascular Nursing; and the Interdisciplinary Council on Peripheral Vascular Disease. The American Academy of Neurology

affirms the value of this statement as an educational tool for neurologists. Stroke 2009;40:2276-93.

Elosua R, Garcia M, Aguilar A, Molina L, Covas MI, Marrugat J. Validation of the Minnesota Leisure Time Physical Activity Questionnaire In Spanish Women. Investigators of the MARATDON Group. Med Sci Sports Exerc 2000;32:1431-7.

Elosua R, Marrugat J, Molina L, Pons S, Pujol E. Validation of the Minnesota Leisure Time Physical Activity Questionnaire in Spanish men. The MARATHOM Investigators. Am J Epidemiol 1994;139:1197- 209.

Esposito K, Marfella R, Ciotola M, et al. Effect of a Mediterranean-style diet on endothelial dysfunction and markers of vascular inflammation in the metabolic syndrome: a randomized trial. JAMA 2004;292:1440-6.

Esposito K, Kastorini CM, Panagiotakos DB, Giugliano D. Mediterranean diet and weight loss: metaanalysis of randomized controlled trials. Metab Syndr Relat Disord 2011;9:1-12.

Estruch R, Martínez-González MA, Corella D, et al. Effects of a Mediterranean-style diet on cardiovascular risk factors. A randomized trial. Ann Intern Med 2006;145:1-11.

Estruch R, Ros E, Salas-Salvado J, et al. Primary prevention of cardiovascular disease with Mediterranean diets: the PREDIMED trial. 2013;368:1279-90.

European Stroke Organisation (ESO) Executive Committee and ESO Writing Committee. Guidelines for management of ischaemic stroke and transient ischaemic attack 2008. Cerebrovasc Dis 2008;25:457-507.

Fernández JM, Rosado-Álvarez D, Da Silva Grigoletto ME, et al. Moderate-to-high-intensity training and a hypocaloric Mediterranean diet enhance endothelial progenitor cells and fitness in subjects with the metabolic syndrome. Clin Sci (Lond) 2012;123:361-73.

Fernández-Ballart JD, Piñol JL, Zazpe I, et al. Relative validity of a semi-quantitative food-frequency questionnaire in an elderly Mediterranean population of Spain. Br J Nutr 2010;103:1808-16.

Fernández-Montero A, Bes-Rastrollo M, Beunza JJ, et al. Nut consumption and incidence of metabolic syndrome after 6-year follow-up: the SUN (Seguimiento Universidad de Navarra, University of Navarra Follow-up) cohort. Public Health Nutr 2012 Oct 23:1-9.

Finucane MM, Stevens GA, Cowan MJ, et al. National, regional, and global trends in body-mass index since 1980: systematic analysis of health examination surveys and epidemiological studies with 960 country-years and 9.1 million participants. Lancet 2011;377:557-67.

First MB, Spitzer RL, Gibbon M, Williams J, Structured Clinical Interview for DSM-IV-TR Axis I Disorders, Research Version, Non-patient Edition (SCID-I/P). New York: Biometrics Research, New York State Psychiatric Institute. 2002.

Fuster V, et al. "ACC/AHA/ESC 2006 guidelines for the management of patients with atrial fibrillation executive summary: a report of the American College of Cardiology/American Heart Association Task Force on Practice Guidelines and the European Society of Cardiology Committee for Practice Guidelines(Writing Committee to Revise the 2001 Guidelines for the Management of Patients with Atrial Fibrillation)." Eur Heart J 2006; 27:1979-2030.

Fito M, Cladellas M, de la Torre R, et al. Anti-inflammatory effect of virgin olive oil in stable coronary disease patients: a randomized, crossover, controlled trial. Eur J Clin Nutr 2008;62:570-4.

Fito M, Fitó M, Guxens M, et al. Effect of a traditional Mediterranean diet on lipoprotein oxidation: a randomized controlled trial. Arch Intern Med 2007;167:1195-1203.

Flegal KM, Kit BK, Orpana H, Graubard BI. Association of all-cause mortality with overweight and obesity using standard body mass index categories: a systematic review and meta-analysis. JAMA 2013;309:71-82.

Flint AJ, Hu FB, Glynn RJ, et al. Excess weight and the risk of incident coronary heart disease among men and women. Obesity (Silver Spring) 2010;18:377-83.

Folstein MF, Folstein SE, McHugh PR. Mini-mental state: A practical method for grading the cognitive state of patients for the clinician. J Psychiatr Res 1975;12:189-98.

Fuentes F, López-Miranda J, Pérez-Martínez P, et al. Chronic effects of a high-fat diet enriched with virgin olive oil and a low-fat diet enriched with alpha-linolenic acid on postprandial endothelial function in healthy men. Br J Nutr 2008;100:159-65.

Gregg EW, Chen H, Wagenknecht LE, et al. Association of an intensive lifestyle intervention with remission of type 2 diabetes. JAMA 2012;308:2489-96.

Guasch-Ferré M, Bulló M, Martínez-González MÁ, et al. Frequency of nut consumption and mortality risk in the PREDIMED nutrition intervention trial. BMC Med 2013;11:164.

Gunstad J, Lhotsky A, Wendell CR, Ferrucci L, Zonderman AB. Longitudinal examination of obesity and cognitive function: results from the Baltimore longitudinal study of aging. Neuroepidemiology 2010;34:222-9.

Gutiérrez-Fisac JL, Guallar-Castillón P, León-Muñoz LM, et al. Prevalence of general and abdominal obesity in the adult population of Spain, 2008-2010: the ENRICA study. Obes Rev 2012;13:388-92.

Haaf P, Drexler B, Reichlin T, et al. High Sensitivity Cardiac Troponin in the Distinction of Acute Myocardial Infarction from Acute Cardiac Non-Coronary Artery Disease, Circulation 2012;126:31-40.

Hall KD, Sacks G, Chandramohan D, et al. Quantification of the effect of energy imbalance on bodyweight. Lancet 2011;378:826-37.

Henríquez Sánchez P, Ruano C, de Irala J, Ruiz-Canela M, Martínez-González MA, Sánchez-Villegas A. Adherence to the Mediterranean diet and quality of life in the SUN Project. Eur J Clin Nutr 2012;66:360-8.

Hernán MA, Taubman SL. Does obesity shorten life? The importance of well-defined interventions to answer causal questions. Int J Obes (Lond) 2008;32 Suppl 3:S8-14.

Howard BV, Van Horn L, Hsia J, et al. Low-fat dietary pattern and risk of cardiovascular disease: the Women's Health Initiative Randomized Controlled Dietary Modification Trial. JAMA 2006;295:655-66.

Hu T, Mills KT, Yao L, et al. Effects of low-carbohydrate diets versus low-fat diets on metabolic risk factors: a meta-analysis of randomized controlled clinical trials. Am J Epidemiol 2012;176 Suppl 7:S44-54.

Hamm CW, Bassand JP, et al. ESC Committee for Practice Guidelines. ESC Guidelines for the management of acute coronary syndromes in patients presenting without persistent ST-segment elevation The Task Force for the management of acute coronary syndromes (ACS) in patients presenting without persistent ST-segment elevation of the European Society of Cardiology (ESC). Eur Heart J 2011;32:2999–3054.

Hughes AJ, Daniel SE, Kilford L, Lees AJ. Accuracy of clinical diagnosis of idiopathic Parkinson’s

disease: a clinic-pathological study of 100 cases. J Neurol Neurosurg Psych 1992;5:181-184.

Jiang R, Jacobs DR Jr, Mayer-Davis E, et al. Nut and seed consumption and inflammatory markers in the multi-ethnic study of atherosclerosis. Am J Epidemiol 2006;163:222-31.

Jones JL, Fernandez ML, McIntosh MS, et al. A Mediterranean-style low-glycemic-load diet improves variables of metabolic syndrome in women, and addition of a phytochemical-rich medical food enhances benefits on lipoprotein metabolism. J Clin Lipidol 2011;5:188-96.

Kastorini CM, Milionis HJ, Esposito K, Giugliano D, Goudevenos JA, Panagiotakos DB. The effect of Mediterranean diet on metabolic syndrome and its components: a meta-analysis of 50 studies and 534,906 individuals. J Am Coll Cardiol 2011; 57:1299-313.

Khanna D, Fitzgerald JD, et al. 2012 American College of Rheumatology guidelines for management of gout. Part 1: systematic nonpharmacologic and pharmacologic therapeutic approaches to hyperuricemia. Arthritis Care Res (Hoboken) 2012;64:1431-1446.

Larsen TM, Dalskov SM, van Baak M, et al. Diets with high or low protein content and glycemic index for weight-loss maintenance. N Engl J Med 2010;363:2102-13.

Li TY, Brennan AM, Wedick NM, Mantzoros C, Rifai N, Hu FB. Regular consumption of nuts is associated with a lower risk of cardiovascular disease in women with type 2 diabetes. J Nutr 2009;139:1333-8.

Look AHEAD Research Group. Cardiovascular effects of intensive lifestyle intervention in type 2 diabetes. N Engl J Med 2013;369:145-54.

López-Miranda J, Pérez-Jiménez F, Ros E, et al. Olive oil and health: Summary of the II International Conference on Olive Oil and Health consensus report, Jaén and Córdoba (Spain) 2008. Nutr Metab Cardiovasc Dis 2010;20:284-94.

Lozano R, Naghavi M, Foreman K, et al. Global and regional mortality from 235 causes of death for 20 age groups in 1990 and 2010: a systematic analysis for the Global Burden of Disease Study 2010. Lancet 2012;380:2095-128.

Ludwig DS. Weight loss strategies for adolescents: a 14-year-old struggling to lose weight. JAMA 2012;307:498-508.

Luppino FS, de Wit LM, Bouvy PF, et al. Overweight, obesity, and depression: a systematic review and meta-analysis of longitudinal studies. Arch Gen Psychiatry 2010;67:220-9.

Malik VS. Hu FB. Popular weight-loss diets: from evidence to practice. Nat Clin Pract Cardiovasc Med 2007;4:34-41.

Malik VS, Willett WC, Hu FB. Global obesity: trends, risk factors and policy implications. Nat Rev Endocrinol 2013;9:13-27.

Martínez-González MÁ, Bes-Rastrollo M. Nut consumption, weight gain and obesity: Epidemiological evidence. Nutr Metab Cardiovasc Dis 2011;21(Suppl 1): S40-45.

Martinez-Gonzalez MA, Bes-Rastrollo M. Dietary patterns, Mediterranean diet, and cardiovascular disease. Curr Opin Lipidol 2014;25:20-6.

Martínez-González MÁ, Corella D, Salas-Salvadó J, et al. Cohort profile: design and methods of the PREDIMED study. Int J Epidemiol 2012;41:377-85.

Martínez-González MÁ, Fernández-Jarne E, Serrano-Martínez M, Wright M, Gomez-Gracia E. Development of a short dietary intake questionnaire for the quantitative estimation of adherence to a cardioprotective Mediterranean diet. Eur J Clin Nutr 2004;58:1550-2.

Masters RK, Powers DA, Link BG. Obesity and US Mortality Risk Over the Adult Life Course. Am J Epidemiol 2013;177:431-42.

McKhann GM, Knopman DS, Chertkow H, et al. The diagnosis of dementia due to Alzheimer's disease: recommendations from the National Institute on Aging-Alzheimer's Association workgroups on diagnostic guidelines for Alzheimer's disease. Alzheimers Dement 2011;7:263-9.

McMurray JJ, Adamopoulos S, Anker SD, Auricchio A, Böhm M, Dickstein K, et Al; ESC Committee for Practice Guidelines. ESC Guidelines for the diagnosis and treatment of acute and chronic heart failure 2012. The Task Force for the Diagnosis and Treatment of Acute and Chronic Heart Failure 2012 of the European Society of Cardiology. Developed in collaboration with the Heart Failure Association (HFA) of the ESC. Eur Heart J 2012;33:1787–1847.

McManus K, Antinoro L, Sacks F. A randomized controlled trial of a moderate-fat, low-energy diet compared with a low fat, low-energy diet for weight loss in overweight adults. Int J Obes Relat Metab Disord 2001;25:1503-11.

Mena MP, Sacanella E, Vazquez-Agell M, et al. Inhibition of circulating immune cell activation: a molecular antiinflammatory effect of the Mediterranean diet. Am J Clin Nutr 2009;89:248-56.

Meneses ME, Camargo A, Perez-Martinez P, et al. Postprandial inflammatory response in adipose tissue of patients with metabolic syndrome after the intake of different dietary models. Mol Nutr Food Res 2011;55:1759-70.

Moher D, Schulz KF, Altman DG. The CONSORT statement: revised recommendations for improving the quality of reports of parallel-group randomised trials. Lancet 2001;357:1191-4.

Moreno JA, López-Miranda J, Pérez-Martínez P, et al. A monounsaturated fatty acid-rich diet reduces macrophage uptake of plasma oxidised low-density lipoprotein in healthy young men. Br J Nutr 2008;100:569-75.

Mozaffarian D, Hao T, Rimm EB, Willett WC, Hu FB. Changes in diet and lifestyle and long-term weight gain in women and men. N Engl J Med 2011;364:2392-404.

National Institute of Diabetes and Digestive and Kidney Diseases. Body weight simulator. See: http://bwsimulator.niddk.nih.gov/, consulted 05/01/2012.

National Institute of Health. ClinicalTrials.gov. A service of the U.S. National Institutes of Health. See: http://clinicaltrials.gov/ct2/home, consulted 05/01/2012.

National Institute of Health. Clinical Guidelines on the Identification, Evaluation, and Treatment of Overweight and Obesity in Adults--The Evidence Report. National Institutes of Health. Obes Res 1998;2(6Suppl):51S-209S.

National Institute of Health, National Heart Lung and Blood Institute and North American Association for the Study of Obesity. The practical guide: Identification, evaluation, and treatment of overweight and obesity in adults. 2000.

Ni Mhurchu C, Rodgers A, Pan WH, Gu DF, Woodward M. Body mass index and cardiovascular disease in the Asia-Pacific Region: an overview of 33 cohorts involving 310 000 participants. Int J Epidemiol 2004;33:751-8.

Nigg CR, Burbank PM, Padula C, et al. Stages of change across ten health risk behaviors for older adults. Gerontologist 1999;39: 473-82.

Nordmann AJ, Nordmann A, Briel M, Keller U, Yancy WS Jr, Brehm BJ, Bucher HC. Effects of low-carbohydrate vs low-fat diets on weight loss and cardiovascular risk factors: a meta-analysis of randomized controlled trials. Arch Intern Med 2006;166:285-93.

Norgren L, Hiatt WR, Dormandy JA, Nehler MR, Harris KA, Fowkes FG. Inter-Society Consensus for the Management of Peripheral Arterial Disease (TASC II). J Vasc Surg 2007;45 Suppl S:S5-67.

O'Brien PC, Fleming TR. A multiple testing procedure for clinical trials. Biometrics 1979;35:549-56.

Pedrero-Perez EJ, Ruiz-Sanchez de Leon JM, Lozoya-Delgado P, Llanero-Luque M, Rojo-Mota G, Puerta-García C. Evaluación de los síntomas prefrontales: propiedades psicométricas y datos normativos del cuestionario disejecutivo (DEX) en una muestra de población española. Rev Neurol 2011; 52: 394-404.

Peña-Casanova J, Quiñones-Ubeda S, Gramunt-Fombuena N, et al. Spanish Multicenter Normative Studies (NEURONORMA Project): norms for verbal fluency tests. Arch Clin Neuropsychol 2009a;24:395-411.

Peña-Casanova J, Quiñones-Ubeda S, Quintana-Aparicio M, et al. Spanish Multicenter Normative Studies (NEURONORMA Project): norms for verbal span, visuospatial span, letter and number sequencing, trail making test, and symbol digit modalities test. Arch Clin Neuropsychol 2009b;24:321-41.

Pérez-Jiménez F, Lista JD, Pérez-Martínez P, et al. Olive oil and haemostasis: a review on its healthy effects. Public Health Nutr 2006;9:1083-8.

Ramier AM, Hécaen H. Role Respectif des atteintes frontales et de la latéralisation lésionelle dans les deficits de la fluence verbal. Rev Neurol (Paris) 1970:123:17-22.

Ramier AM, Hécaen H. Les déficits au test de “fluence verbale” chez les subjets guachers avec lesions hemispheriques unilaterales. Rev Neurol (Paris) 1977:133: 571–4.

Razquin C, Martinez JA, Martinez-Gonzalez MA, Mitjavila MT, Estruch R, Marti A. A 3 years follow-up of a Mediterranean diet rich in virgin olive oil is associated with high plasma antioxidant capacity and reduced body weight gain. Eur J Clin Nutr 2009;63:1387-93.

Razquin C, Martínez JA, Martínez-González MA, Salas-Salvadó J, Estruch R, Marti A. A 3-year Mediterranean-style dietary intervention may modulate the association between adiponectin gene variants and body weight change. Eur J Nutr 2010;49:311-9.

Reitan RM. Trail Making Test: Manual for administration and scoring. Tucson: Reitan Neuropsychology Laboratory; 1992.

Rejeski WJ, Ip EH, Bertoni AG, et al. Lifestyle Change and Mobility in Obese Adults with Type 2 Diabetes. N Engl J Med 2012;366:1209-17.

Romaguera D, Norat T, Vergnaud AC, et al. Mediterranean dietary patterns and prospective weight change in participants of the EPIC-PANACEA project. Am J Clin Nutr 2010;92:912-21.

Ros E. Nuts and novel biomarkers of cardiovascular disease. Am J Clin Nutr 2009; 89:1649S-56S.

Ruano J, Lopez-Miranda J, Fuentes F, et al. Phenolic content of virgin olive oil improves ischemic reactive hyperemia in hypercholesterolemic patients. J Am Coll Cardiol 2005; 46:1864-8.

Ruiz-Canela M, Estruch R, Corella D, Salas-Salvadó J, Martínez-González MA. Association of Mediterranean diet with peripheral artery disease: the PREDIMED randomized trial. JAMA 2014;311:415-7.

Ryan DH, Espeland MA, Foster GD, et al. Look AHEAD (Action for Health in Diabetes): design and methods for a clinical trial of weight loss for the prevention of cardiovascular disease in type 2 diabetes. Control Clin Trials 2003;24:610-28.

Sabaté J, Oda K, Ros E. Nut consumption and blood lipid levels: a pooled analysis of 25 intervention trials. Arch Intern Med 2010;170:821-7.

Sacco RL, Kasner SE, Broderick JP, et al. An Updated Definition of Stroke for the 21st Century: A Statement for Healthcare Professionals from the American Heart Association/American Stroke Association. Stroke 2013;44:2064-2089.

Sacks FM, Bray GA, Carey VJ, et al. Comparison of weight-loss diets with different compositions of fat, protein, and carbohydrates. N Engl J Med 2009;360:859-73

Salas-Salvadó J, Fernández-Ballart J, Ros E, et al. Effect of a Mediterranean diet supplemented with nuts on metabolic syndrome status: one-year results of the PREDIMED randomized trial. Arch Intern Med 2008;168:2449-58.

Salas-Salvadó J, Bulló M, Babio N, et al. Reduction in the incidence of type 2 diabetes with the Mediterranean diet: results of the PREDIMED-Reus nutrition intervention randomized trial. Diabetes Care 2011;34:14-9.

Salas-Salvadó J, Bulló M, Estruch R, et al. Prevention of diabetes with Mediterranean diets: a subgroup analysis of a randomized trial. Ann Intern Med 2014;160:1-10.

Sanchez-Villegas A, Ara I, Dierssen T, de la Fuente C, Ruano C, Martínez-González MA. Physical activity during leisure time and quality of life in a Spanish cohort: SUN (Seguimiento Universidad de Navarra) project. Br J Sports Med 2012;46:443-8.

Sanz, Navarro y Vázquez, Adaptación española para el Inventario de Depresión de Beck-II (BDI-II). 1. Propiedades psicométricas en estudiantes universitarios. Análisis y modificación de conducta 2003;29: 239-288.

Schröder H, Fitó M, Estruch R, et al. A short screener is valid for assessing Mediterranean diet adherence among older Spanish men and women. J Nutr 2011;141:1140-5.

Schulz KF, Grimes DA. Multiplicity in randomised trials II: subgroup and interim analyses. Lancet 2005;365:1657-61.

Schulze MB, Fung TT, Manson JE, Willett WC, Hu FB. Dietary patterns and changes in body weight in women. Obesity (Silver Spring) 2006;14:1444-53.

Shai I, Schwarzfuchs D, Henkin Y, et al. Weight loss with a low-carbohydrate, Mediterranean, or low-fat diet. N Engl J Med 2008;359:229-41.

Smith, A. Symbol Digit Modalities Test. Manual. Los Angeles: Western Psychological Services; 1973.

Sofi F, Abbate R, Gensini GF, Casini A. Accruing evidence on benefits of adherence to the Mediterranean diet on health: an updated systematic review and meta-analysis. Am J Clin Nutr 2010;92:1189-96.

Song YM, Sung J, Davey Smith G, Ebrahim S. Body mass index and ischemic and hemorrhagic stroke: a prospective study in Korean men. Stroke 2004;35:831-6.

Tendera M, Aboyans V, Bartelink ML, et al. ESC Guidelines on the diagnosis and treatment of peripheral artery diseases. Eur Heart J 2011;32: 2851–2906.

Tobias DK, Hu FB. Does being overweight really reduce mortality? Obesity (Silver Spring) 2013;21:1746-9.

Thygesen K, Alpert JS, Jaffe L, et al. Third universal definition of myocardial infarction. Eur Heart J 2012;33:2551–2567.

Urpi-Sarda M, Casas R, Chiva-Blanch G, et al. Virgin olive oil and nuts as key foods of the Mediterranean diet effects on inflammatory biomakers related to atherosclerosis. Pharmacol Res 2012; 65:577-83.

Wallston KA, Stein MJ, Smith CA. Form C of the MHLC scales: a condition-specific measure of locus of control. J Pers Assess 1994;63:534-53.

Wallston, Wallston, & DeVellis, 1978, Health Education Monographs, 6, 160-170.

Ware JE, Gandek B, Gandek BJr. Overview of the SF-36 Health Survey and the International Quality of Life Assessment (IQOLA) Project. J Clin Epidemiol 1998;51:903-12.

Wechsler, D. Wechsler Adult Intelligence Scale-III. San Antonio, Texas: Psychological Corporation; 1997a.

Wechsler, D. Wechsler Memory Scale. San Antonio, Texas: Psychological Corporation; 1997b.
Wechsler, D. WAIS III. Escala de inteligencia de Wechsler para adultos-III. Manual técnico. Madrid: TEA Ediciones; 1999.

Wechlsler D. Escala de memoria Wechsler.-III. Manual técnico: TEA Ediciones. S. A; 2004.

Willett WC. Dietary fat plays a major role in obesity: no. Obes 2002;Rev 3:59-68.

Willett WC. Eat, drink, and be healthy: the Harvard Medical School guide to healthy eating. New York: Free Press, 2001

Wilson BA, Alderman N, Burgess PW, Emslie H, Evans JJ. Behavioural assessment of the dysexecutive syndrome. Bury St. Edmunds, UK: Thames Valley Test; 1996.

Wing RR. Long-term effects of a lifestyle intervention on weight and cardiovascular risk factors in individuals with type 2 diabetes mellitus: four-year results of the Look AHEAD trial. Arch Intern Med 2010;170:1566-75.

World Health Organization. Obesity: Preventing and managing the global epidemic. Geneva: World Health Organization, 1998.World Health Organization. International Classification of Diseases for Oncology, 3rd Edition (ICD-O-3).
http://www.who.int/classifications/icd/adaptations/oncology/en/index.html, consulted 24/04/2012.

Yesavage JA. Geriatric Depression Scale. Psychopharmacol Bull 1988;24:709-11.

Yancy CW, Jessup M, Bozkurt B, et al. 2013 ACCF/AHA guideline for the management of heart failure: a report of the American College of Cardiology Foundation/American Heart Association Task Force on Practice Guidelines. J Am Coll Cardiol 2013; 62: e147- e239.

Zazpe I, Sanchez-Tainta A, Estruch R, et al. A large randomized individual and group intervention conducted by registered dietitians increased adherence to Mediterranean-type diets: the PREDIMED study. J Am Diet Assoc 2008;108:1134-44

Zhu L, Liu Z, Feng Z, et al. Hydroxytyrosol protects against oxidative damage by simultaneous activation of mitochondrial biogenesis and phase II detoxifying enzyme systems in retinal pigment epithelial cells. J Nutr Biochem 2010;21:1089-98.

ANNEX 1. SUB-STUDIES

1. Body composition

DEXA or computed tomography will be used to measure body composition at recruiting centers wherein the necessary equipment and technology are available. Body composition will be analyzed by General Electric Lunar DEXA scanner at the Rovira i Virgili University, Universitat de les Illes Balears, Hospital Clinico de Barcelona, and the Departments of Preventive Medicine and Nutrition in Navarra.

2. Other sub-studies

Depending on available funds, sub-studies will be conducted to evaluate gene environment interactions, epigenetic factors such as DNA methylation, histone modification and microRNA alterations, the composition and function of intestinal microbiota by pyrosequencing, and the effect of the intervention on metabolomics, transcriptomics and proteomics.

ANNEX 2. PARTICIPATING CENTERS

Recruitment centers

ID	PI	Email address	Center
01	Enrique Gómez-Gracia	egomezgracia@gmail.com	Universidad de Málaga, Málaga
02	José Lapetra	jlapetra@ono.com	Distrito Sanitario de Atención Primaria de Sevilla, Sevilla
03	J. Alfredo Martínez	jalfmtz@unav.es	Universidad de Navarra - Nutrition, Pamplona
04	María Adoración Romaguera; Miquel Fiol	mariaadoracion.romaguera@ ssib.es; miquelfiol@yahoo.es	Hospital Son Espases, Palma
05	Ramon Estruch	restruch@clinic.ub.es	Hospital Clínic-Medicina Interna, Barcelona
06	Montserrat Fitó	mfito@imim.es	Institut Hospital del Mar d'Investigacions Mèdiques, Barcelona
07	Jordi Salas-Salvadó	jordi.salas@urv.cat	Universitat Rovira i Virgili, Reus
08	Aurora Bueno	abueno@ugr.es	Universidad de Granada, Granada
09	Clotilde Vázquez	cvazquezma@gmail.com	Hospital Ramón y Cajal, Madrid
10	Miguel Ángel Martínez-González	mamartinez@unav.es	Universidad de Navarra - Epidemiology, Pamplona
11	Fernando Aròs	aborau@htxa.osakidetza.net	Hospital Universitario Araba, Vitoria
12	Dolores Corella	dolores.corella@uv.es	Universidad de Valencia, Valencia
13	Lluis Serra-Majem	lserra@dcc.ulpgc.es	Universidad de las Palmas de Gran Canaria, Las Palmas
14	Xavier Pintó	xpinto@csub.scs.es	Hospital de Bellvitge, L'Hospitalet de Llobregat, Barcelona
15	José López Miranda	jlopezmir@gmail.com	Universidad de Córdoba, Córdoba
16	José Maria Ordovás	Jose.Ordovas@tufts.edu	IMDEA, Madrid
17	Pilar Matía Martín	pilar.matia@gmail.com	Hospital Clínico, Madrid
18	Francisco Tinahones	fjtinahones@hotmail.com	Hospital de Málaga, Málaga
19	Josep Tur	pep.tur@uib.es	Universitat de les Illes Balears, Palma
20	Josep Vidal	JOVIDAL@clinic.ub.es	Hospital Clínic – Endocrinología, Barcelona

Support centers

ID	PI	Email address	Center
**A1**	Emilio Ros	eros@clinic.ub.es	Hospital Clínic, Barcelona
**A2**	Rosa M. Lamuela-Raventós	lamuela@ub.edu	Universitat de Barcelona
**A3**	Guillermo Sáez	guillermo.Saez@uv.es	Universitat de Valencia
**A4**	María del Puy Portillo	mariapuy.portillo@ehu.es	Universidad del País Vasco
**A5**	Ascensión Marcos	amarcos@ictan.csic.es	CSIC, Madrid
**A6**	Cristina Botella	botella@uji.es	Universitat Jaume I de Castellón
**A7**	Fernando Fernández-Aranda	ffernandez@bellvitgehospital.cat	Hospital Universitari de Bellvitge

EFFECT OF AN ENERGY-RESTRICTED MEDITERRANEAN DIET, PHYSICAL ACTIVITY AND BEHAVIORAL TREATMENT ON THE PRIMARY PREVENTION OF CARDIOVASCULAR DISEASE

THE PREDIMED-PLUS TRIAL

RESEARCH PLAN

April 2018

IN THIS VERSION OF THE PROTOCOL THE AMMENDMENTS DONE FROM 2014 TO APRIL 2018 ARE INCLUDED

ABSTRACT

In the 21st century we are witnessing an **alarming increase in overweight and obesity**. In **Spain**, **over 60%** of adults are **overweight or obese** and the prevalence of adult **abdominal obesity exceeds 35%** (Gutiérrez-Fisac et al., 2012). The increase in morbid obesity is especially worrying (Basterra-Gortari et al., 2011) because the medium-to-long-term consequences for the **risk of cardiovascular** disease (CVD) and other causes of death or illness can be **catastrophic**.

Observational studies have shown that all-cause mortality increases progressively in parallel with adiposity and that the risk of **cardiovascular mortality** is especially high (Berrington de González et al., 2010; GBD 2015 Obesity Collaborators). However, a meta-analysis published in early 2013 (Flegal, 2013) raised considerable controversy by much downgrading the consequences of overweight and moderate obesity. The results of this meta-analysis could be explained by the existence of biases, such as unusual definitions for categories of overweight, tobacco as a confounding factor, inverse causality, and the exclusion of relevant studies (Tobias and Hu, 2013). Nevertheless, the controversy persists and will probably do so well into the next few decades. Doubts such as these will only be solved by randomized intervention trials (Hernán and Taubman, 2008). Thus, clinical studies are needed to determine whether **intentional weight loss** reduces cardiovascular mortality and CVD incidence.

In the PREDIMED-PLUS trial we will evaluate the safety and effectiveness of a multifaceted intervention program for alleviating excessive cardiovascular morbidity and mortality in overweight and obese individuals. The study's aim is to determine the effect on CVD morbidity and mortality of an intensive weight loss intervention program based on an energy-restricted traditional Mediterranean diet, increased physical activity and behavioral support in comparison with an intervention based on standard dietary advice (energy-unrestricted Mediterranean diet) and traditional health care for CVD prevention. We hypothesize that an intensive lifestyle intervention program aimed at weight loss and based on the traditional Mediterranean diet is a sustainable long-term approach for achieving weight loss in overweight and obese adults and that the lifestyle changes achieved will have a beneficial effect on cardiovascular morbidity and mortality (Estruch et al., 2013; Shai et al., 2008). This study may provide a useful tool for tackling excess morbidity and mortality associated with overweight and obesity.

Objectives

The PREDIMED-PLUS trial evaluates the effect on primary CVD prevention of an intensive intervention program comprising a 17-item energy-restricted Mediterranean

diet with specific weight-loss goals, physical activity promotion and behavioral support (intervention group) in comparison to a control group using Mediterranen diet recommendations (comprising a 14-item energy-unrestricted Mediterranean diet) but without advice to increase physical activity or reduce energy intake (Schröder et al., 2011) (control group). The diet assigned to the control group had previously been shown to be effective for CVD prevention in the PREDIMED trial (Estruch et al., 2013). The primary end-point is a composite of hard clinical cardiovascular events (myocardial infarction, stroke or cardiovascular death) as defined in the PREDIMED trial (Martinez-Gonzalez et al., 2012).

The main objective of PREDIMED-PLUS is to evaluate, in comparison with a **control group** given non-intensive, energy-unrestricted dietary advice (also Mediterranean-type), the effect of an **intensive lifestyle intervention** comprising an **energy-restricted Mediterranean diet**, increased physical activity and behavioral support on:

1. The incidence of **cardiovascular events** (non-fatal myocardial infarction, non-fatal stroke, or cardiovascular death).

2. **Weight loss** and long-term maintenance of weight-loss.

The secondary objectives are to determine whether an intensive weight-loss-oriented lifestyle intervention program has a beneficial effect to reduce waist circumference and the following overweight- and obesity-related conditions: acute coronary syndromes with or without coronary revascularization, heart failure, atrial fibrillation, peripheral artery disease, venous thrombosis, type-2 diabetes mellitus and its complications, overall incidence of cancer, specific cancers in main cancer sites (breast, colorectal, prostate, lung and stomach), osteoporotic fractures, gallstone disease, symptomatic gout, neurodegenerative disorders (dementia and Parkinson's disease), unipolar depression and eating behavior disorders. We will also determine the effect of the intervention on the following intermediate markers: nutrient intake and overall dietary pattern, systolic and diastolic blood pressure, blood lipids, fasting glucose level, kidney function, uric acid level, the percentage of individuals requiring anti-hypertensive, anti-diabetic or lipid-lowering medication, C-reactive protein levels, hemoglobin A1C levels, liver function, ECG traits, cognitive function, quality of life, and psychopathological symptoms.

Methodology

We are conducting a randomized, multicenter **field trial** aimed at the primary prevention of CVD in overweight or obese adults with metabolic syndrome using an intensive intervention program based on an energy-restricted Mediterranean diet, increased physical activity, and behavioral support and a control group given advice on an *ad libitum* Mediterranean diet for the prevention of cardiovascular morbidity and mortality in accordance with the PREDIMED trial. We stress to participants the importance of attending medical visits and provide them with general written recommendations on lifestyle for the management of the metabolic syndrome. This new trial, the planning and design of which is outlined in this paper, is called **PREDIMED-PLUS**.

There are 23 recruiting centers involved in this new multicenter trial including a total of **6,874 participants**, of whom **3,406** were assigned to the intensive intervention group and **3,468** to the control group. Recruitment began in September 2013 and ended in December 2016. The intervention will last at least 6 years and the median follow-up time for the clinical endpoints is expected to be 8 years. The results of the trial, including anthropometric changes and impact on major obesity-related disorders, are expected to be highly applicable to public healthcare since they will provide a better prognosis for overweight and obese adults. The results are also expected to be highly efficient since they should provide a non-pharmacological approach to the prevention of the main cause of mortality and one of the leading causes of loss of disability-adjusted life years (Lozano et al., 2012).

BACKGROUND

The **global overweight and obesity epidemic** is increasing at an alarming rate. Now a global public health crisis, it affects over 60% of the adult population. Between 1980 and 2008, worldwide obesity prevalence almost **doubled** (Finucane et al., 2011; Malik et al., 2013; GBD 2015 Obesity Collaborators, 2017; González-Muniesa et al, 2017). In Spain, the **prevalence of adult abdominal obesity is over 35%** and **over 60%** of the adult population is overweight or obese (Gutiérrez-Fisac et al., 2012). Moreover, there is a particularly worrying increase in morbid obesity (Basterra-Gortari et al., 2011). The medium-to-long-term consequences of obesity on the risk of CVD and death are **devastating** and have the capacity to both **render the health system unsustainable** and curtail economic growth. Urgent priority must be given to finding solutions based on the best scientific evidence available.

The link between obesity and mortality appears to diminish with age. However, if this were true, it would be difficult to recommend weight loss for older populations. This notion has been challenged by observational epidemiological studies that, after generational and cohort confusion are adequately controlled, suggest the opposite, i.e., that with advancing age the link between obesity and mortality becomes even stronger (Masters, 2013).

A meta-analysis of observational studies published in 2013 (Flegal, 2013) raised controversy by considerably downgrading the consequences of overweight and moderate obesity. However, as Tobias and Hu (2013) have argued, the conclusions of Flegal's meta-analysis can probably be explained by the existence of biases, such as unusual definitions for categories of overweight, tobacco as a confounding factor, inverse causality, and the exclusion of relevant studies. This issue will only be solved by randomized clinical trials (Hernán and Taubman, 2008). However, the controversy is likely to continue well into the next few decades until the results of clinical intervention studies are available that overcome the limitations and inherent bias of the observational designs that have evaluated the link between overweight or obesity and the incidence of serious clinical events or mortality in initially healthy individuals. Observational studies with a better control of bias have found that all-cause mortality increases progressively as adiposity outside the normal weight range—measured by body mass index (BMI, defined as weight in kilograms divided by the square of height in meters)—increases, and that this risk is especially high for **cardiovascular mortality** (Berrington de González et al., 2010; GBD 2015 Obesity Collaborators). An increase in body weight is associated not only with higher mortality but also with greater morbidity due to CVD (Ni Mhurchu et al., 2004; Song et al., 2004; Flint et al., 2010); greater risk of developing

some types of cancer (Lauby-Secretan B, et al 2016), diabetes and depression (Luppino et al., 2010); and poorer cognitive function (Gunstad et al., 2010). Large-scale randomized studies with robust designs are needed to obtain best-quality evidence.

Expert panels set up by the National Institutes of Health and the World Health Organization recommend that overweight and obese adults with comorbid conditions should lose 10% of their initial weight and that a lifestyle intervention program should be the primary treatment (National Institute of Health, 1998; World Health Organization, 1998). Moreover, according to the American Dietetic Association, a weight-loss-oriented intensive lifestyle intervention program should include an energy-restricted diet, physical activity and behavioral therapy. The only randomized trial that has addressed the long-term effect of an intensive weight-loss lifestyle program in obese adults on CVD and mortality is the Look AHEAD study (Ryan, 2003). This trial, which comprised 5,145 participants (Rejeski et al., 2012), ended prematurely in October 2012 due to lack of efficiency (Look AHEAD Research Group, 2013). The trial included only diabetic subjects and used a low-fat diet (<30% of total energy intake with <10% from saturated fat). To some extent this is opposite to the Mediterranean diet used in the PREDIMED trial (Zazpe et al., 2008; Martínez-González et al, 2012; Estruch et al., 2013), which is rich in vegetable fats such as healthful virgin olive oil and nuts. In recent years, scientific associations have recommended low-fat diets that contribute less than 30% of energy in the form of fat as the most suitable way to promote general health and weight loss. The high energy density and high palatability of high-fat foods are feared to produce potentially adverse effects on body weight and cardiovascular health (National Institute of Health, 2000). However, the discontinuation of the Look AHEAD trial due to futility, the inefficiency of the Women's Health Initiative Dietary Modification Trial (Look Ahead Research Group, 2013; Howard, 2006), and the favorable results of the PREDIMED (Estruch et al., 2013) and DIRECT studies (Shai et al., 2008) provide powerful arguments against approaches based on low-fat diets.

Although diets that recommend complex carbohydrates, a reduction in fat intake and energy restriction to produce weight loss are generally accepted, there is no clear evidence that dietary fat is associated with a greater increase in weight (Willett, 2001; Nordmann et al., 2006; Larsen et al., 2010; Hu et al., 2012; Bueno et al., 2013). One dietary paradigm that is different from the low-fat diet and that can be more useful for developing and implementing programs aimed at achieving prolonged weight loss and improving the metabolic alterations associated with overweight and obesity is the **traditional Mediterranean diet**. This dietary pattern is rich in fat from vegetable sources (virgin olive oil and nuts) and includes an abundance of minimally processed plant foods

(vegetables, fruits, whole grains and legumes), low consumption of meat (especially red and processed meats), moderate consumption of fish and wine (which is usually consumed with meals) and frugal meals. The high fat contents of the traditional Mediterranean diet make the diet more palatable and therefore more acceptable and easily sustainable in the long term.

In its 2010 edition, the Dietary Guidelines for Americans recognized the traditional Mediterranean diet, together with the DASH diet, as a healthy diet for the prevention of CVD, although when the recommendation was made, randomized clinical trials with regard to the primary prevention of major clinical events as the main outcome had not yet been conducted. This was confirmed by the results of the PREDIMED trial on the primary prevention of cardiovascular disease conducted in Spain between 2003 and 2010 (Estruch et al., 2013).

With regard to the weight-loss properties of the traditional Mediterranean diet, in a meta-analysis of randomized trials, allocation to a Mediterranean diet in comparison with control diets showed a small but significant effect on body weight reduction (mean differences: -1.75 kg, CI 95%: -2.86 a -0.64 kg). This effect was doubled when the Mediterranean diet was energy-restricted (Esposito et al., 2011). Subsequently the PREDIMED trial showed similar advantages for the prevention of weight gain in a considerably longer-term follow-up and with a higher sample size (Estruch et al, 2016).

Several meta-analyses of observational studies (Sofi et al., 2010; Sofi et al, 2014; Martínez-González et al, 2017; Dinu et al, 2018) found that greater adherence to the Mediterranean diet was associated with significant reductions in total mortality, cardiovascular mortality, mortality due to cancer, the incidence of non-fatal cardiovascular events, and the risk of neurodegenerative illnesses. A subsequent update of the meta-analysis of the Mediterranean diet and CVD reported a 13% relative reduction in risk for every two-point increase in adherence to the Mediterranean diet (scale 0–9) after identifying and treating sources of heterogeneity (Martínez-Gonzalez and Bes-Rastrollo, 2014).

The **PREDIMED** (**PREvención con DIeta MEDiterránea**) trial, which included 7,447 participants over an average of five years, was the largest nutritional intervention **trial** ever conducted **in** Europe. PREDIMED showed that, in comparison with advice on a low-fat diet, a high-fat **Mediterranean diet** supplemented with extra-virgin olive oil or mixed nuts implemented in a setting of primary cardiovascular prevention resulted in a **30% reduction in CVD events** after intervention for a median of 4.8 years (Estruch et al., 2013). **PREDIMED** is recognized worldwide as a landmark study that marks a turning point in the **prevention of chronic diseases**. The effective reduction in cardiovascular

events when the **Mediterranean diet** was used in a randomized trial provides the best-possible scientific evidence for preventing **CVD**, **the main cause of death in the world**. We should also point out that the **PREDIMED** diets were *ad libitum*, increased physical activity was not promoted, and no counsel to lose weight was given.

It has been postulated that the link between adherence to the traditional Mediterranean diet and the risk of CVD can be mediated by several mechanisms, including a reduction in low degree inflammation (Chrysohoou et al., 2004; Esposito et al., 2004; Mena et al., 2009; Camargo et al., 2011; Urpi-Sarda et al., 2012; Meneses et al., 2012), higher levels of adiponectin (Detopoulou et al., 2010; Razquin et al., 2010), lower coagulability (Chrysohoou et al., 2004; Pérez-Jiménez et al., 2006; Pérez-Jiménez et al., 2002), improved endothelial function (Esposito et al., 2004; Ruano et al., 2005; Fuentes et al., 2008), lower oxidative stress (Dai et al., 2008; Chrysohoou et al., 2011; Razquin et al., 2009), a lower concentration of atherogenic lipoproteins (Jones et al., 2012), lower levels of oxidized LDL particles (Fito et al., 2007), and a lower uptake of oxidized LDL by macrophages (Moreno et al., 2008). Moreover, the two foods supplemented in PREDIMED (extra-virgin olive oil and nuts) also have beneficial biological properties. Extra-virgin olive oil has a healthy fatty acid profile and contains numerous bioactive phenolic compounds (Pérez-Jiménez et al., 2006; Covas et al., 2009; López-Miranda et al., 2010). The phenolic compounds of olive oil have anti-inflammatory properties (Fito et al., 2008), beneficially impact he lipid profile (Benkhalti et al., 2002; Covas et al., 2006), improve oxidative stress markers (Covas et al., 2006), have a platelet antiagregant effect (de Roos et al., 2011; Fito et al., 2008), and stimulate mitochondrial biogenesis (Zhu et al., 2010). Nuts also have a healthy fatty acid profile, based on mono- and polyunsaturated fatty acids, and contain minerals, vitamins and other antioxidant bioactives, essential amino acids, fiber, and phytosterols (Ros, 2009). The consumption of nuts has been associated with lower levels of total cholesterol, LDL and non-HDL cholesterol, and apolipoprotein B-100 (Li et al., 2009; Sabaté et al., 2010), and lower inflammation (Jiang et al., 2006). Nuts also have an antioxidant effect, benefit heart rate and improve platelet aggregation and endothelial function (Ros, 2009; Defilippis et al., 2010). All of these mechanisms explain the antiatherogenic effect of a Mediterranean diet that is rich in nuts and extra-virgin olive oil. In fact, in the PREDIMED trial a strong protective effect against peripheral artery disease was observed (Ruiz-Canela et al., 2014). In a sub-study of the PREDIMED trial we also observed that both a Mediterranean diet enriched with nuts and a Mediterranean diet enriched with olive oil reduced the incidence of type-2 diabetes by 48% (Salas-Salvadó et al., 2011). When we analyzed this association among all study's participants, we also found that the

Mediterranean diet had a significant protective effect against diabetes (Salas- Salvadó et al., 2014).

Though PREDIMED study, was not a weight loss trial, the provision of abundant fat-rich foods from natural vegetable origin (extra-virgin olive oil and nuts) did not conduct to weight gain. There is still insufficient **experimental evidence** to support the hypothesis that intentional weight loss via a healthy diet and favorable lifestyle changes reduces mortality or the incidence of CVD in the long term. Specifically, the impact of weight loss on the risk of CVD within the framework of a **Mediterranean dietary pattern** has not yet been tested in a sufficiently large randomized clinical trial (Malik and Hu, 2007). In light of the obesity epidemic, we propose to conduct a new trial, **PREDIMED-PLUS**, which will go beyond the achievements of the **PREDIMED** trial in order to tackle more specifically the problems of overweight and obesity. Our proposed strategy has positive effects for weight loss (based on the loss of fat mass) and long-term weight-loss maintenance (Shai et al., 2008; Beunza et al., 2010; Romaguera et al., 2010; Estruch et al 2016). Even more interestingly, this research may demonstrate that a **multifaceted lifestyle intervention** program (dietary pattern + weight loss + physical activity + behavioral support) can be an even more effective means of reducing the cardiovascular risk associated with overweight and obesity than a non-energy-restricted traditional Mediterranean diet. We expect our contribution via the **PREDIMED-PLUS** trial to reveal synergies between the effects of an intensive weight-loss intervention program (with **energy restriction**, physical activity and behavioral support) and the beneficial effects of greater adherence to a high-quality diet (the **Mediterranean diet**) on the incidence of CVD.

Blood and urine samples will be collected and stored at the beginning and throughout the trial. Later analyses of molecular/biochemical biomarkers within the framework of genetic, epigenetic, transcriptomic, metabolomic and proteomic studies might help to determine the benefits of the intervention and the underlying mechanisms.

HYPOTHESIS

An **intensive lifestyle intervention program** based on an energy-restricted **traditional Mediterranean diet**, increased physical activity and behavioral support is a **sustainable** approach that leads to long-term weight loss in overweight and obese adults with metabolic syndrome in such a way that the changes in lifestyle achieved will have long-term benefits on the incidence of CVD.

In comparison with a control intervention that provides advice on the Mediterranean diet but does not restrict calorie intake and does not promote physical activity, an intensive lifestyle intervention based on an energy-restricted **traditional Mediterranean diet**, promotion of physical activity, and behavioral support (Intervention group) in overweight or obese individuals with metabolic syndrome will:

1. Reduce the risk of cardiovascular events;
2. Achieve a greater reduction in body weight and lead to better long-term weight-loss maintenance;

OBJECTIVES

Our long-term objective is to provide effective treatment for reducing excessive **cardiovascular morbidity and mortality** in overweight or **obese** adults**,** irrespective of whether the participants are diabetic at the beginning of the study. To achieve this, we will compare the effects on rates of cardiovascular disease of an **intensive** lifestyle and weight loss intervention program based on the **traditional Mediterranean diet** and including increased physical activity, energy restriction and behavioral support (intervention group) with that of a non-intensive intervention program that provides both education on **the traditional Mediterranean diet for the prevention of CVD in accordance with the principles outlined in the PREDIMED trial and usual care** by primary healthcare professionals (control group). The importance of attending visits to healthcare professionals will be stressed and general recommendations on management of the metabolic syndrome will be provided.

Main specific objectives

To evaluate the effect of an **intensive** weight-loss-oriented lifestyle intervention program based on a **traditional Mediterranean diet** with **energy restriction**, **increased physical activity and behavioral** therapy on:

1. The incidence of CVD (non-fatal myocardial infarction, non-fatal stroke and cardiovascular death);
2. **Weight loss and long-term weight-loss maintenance**;

Secondary specific objectives

This intensive intervention program is likely to result in reduction of waist circumference and acute coronary syndromes, coronary revascularization, total mortality, heart failure, peripheral artery disease, venous thrombosis, atrial fibrillation, type-2 diabetes and its complications, total cancer, cancer in main cancer sites (breast,

prostate, colorectal, lung and stomach), gallstone diseases, symptomatic gout, neurodegenerative disorders (dementia and Parkinson's disease), unipolar depression, osteoporotic fractures, and eating behavior disorders.

We will also address the effect of the intervention on the following intermediate outcomes: nutrient intake and overall dietary pattern, systolic and diastolic blood pressure, serum lipid concentrations, fasting glucose, glycated hemoglobin and uric acid, kidney function, liver function, C-reactive protein, anti-hypertensive, anti-diabetic and lipid-lowering medication needs, ECG traits, cognitive function, quality of life, and psychopathological scales.

We will also store plasma, serum, peripheral cells and urine samples to evaluate other hypotheses in the future, depending on availability of additional funding.

METHODOLOGY

1. Summary

We are conducting a parallel-group, multicenter, randomized, primary prevention trial in adult **men aged 55–75** and adult **women aged 60–75** with a **BMI ≥27 and <40 kg/m²** who meet at least **three criteria for the metabolic syndrome**. In the Caucasian population, cardiovascular risk is considered to be increased if waist circumference is ≥ 80 cm in women and ≥ 94 cm in men. In the South American population, the value is the same for women but for men risk is considered to increased if waist circumference is ≥ 90 cm (Alberti et al., 2009). Diabetic participants comprise 27% of the final sample. This latter component and the use of the **traditional Mediterranean diet** substantially distinguish the PREDIMED-PLUS trial from the Look AHEAD trial conducted in the United States (Ryan et al., 2003; Gregg et al., 2012; Look AHEAD Research Group, 2013), which was recently discontinued due to lack of efficiency. Participants were divided proportionally at random into two groups: a control group and an intensive lifestyle intervention group. Intervention will be maintained for **6 years** and average follow-up time **for clinical events will be 8 years**.

The control group receives usual healthcare from primary care medical professionals, all the written material, instructions on following the traditional **Mediterranean diet**—which was used in the PREDIMED study and has been shown to have benefits for the prevention of cardiovascular morbidity and mortality—and general lifestyle recommendations for managing the metabolic syndrome. Every six months, the control group participants are also invited to participate in group sessions led by the team of PREDIMED-PLUS dietitians, wherein they receive a free supply of virgin olive oil (6

liters every 6 months) and nuts (750 g every 6 months) in order to promote the Mediterranean diet and encourage compliance with the trial.

Participants in the **intensive lifestyle intervention** group are prescribed a **traditional Mediterranean diet** but in this case it is **energy-restricted**. Dietary intervention is associated with increased physical activity and behavioral therapy programs. It has specific weight-loss objectives and includes self-monitoring and frequent follow-up throughout the study. Participants in this group take part in individual interview sessions and motivational group sessions three times per month during the first year of the intervention and twice per month thereafter. They are provided free extra-virgin olive oil (one liter per month) and nuts (125 g per month of the total recommended amount of 500g)[*]. Initially, we decided to provide 500 g per month at no cost of nuts to each participant during the intervention in order to reinforce adherence to the Mediterranean diet in both arms of the trial. However, due to lack of economic resources, only participants included in the pilot study recruited in 2 vanguard centers up to July 2014 received 250 g of free nuts (125g of pistachios and 125g of almonds per month) along with the recommendation to consume a total monthly amount of 500g. Subsequently, all trial participants received during the follow-up 125g of almonds per month out of the total recommended amount of 500g. The participants' degree of compliance with the intervention is monitored periodically so that the intervention can be adjusted if necessary.

For the intensive intervention group, the specific weight-loss objectives are to achieve **an average reduction in baseline body weight of over 8%** and an average **reduction in waist circumference of over 5%** in the first six months and to maintain these figures over an additional period of seven and a half years. The final objective is to obtain a between-group average absolute difference in weight loss and waist circumference reduction of over 5%.

Primary final outcomes include: a) non-fatal myocardial infarction, b) non-fatal stroke, and c) cardiovascular death. Other primary objective will be weight loss (and weight-loss maintenance). The trial protocol will be registered at ClinicalTrials.gov

---

[*]*The decision to recommended 500 g of mixed nuts per month was based on the results of epidemiological and clinical studies. For example, the SUN (Seguimiento Universidad de Navarra) study found that individuals who consumed nuts two or three times per week (400 g/month) had a significantly lower risk of weight gain and metabolic syndrome than those who rarely ate them or never did (Fernández-Montero et al., 2012; Bes-Rastrollo et al., 2007). A Mediterranean-type diet with moderate fat intake containing 25 g per day (750 g/month) of peanuts or other types of nuts was found to be associated with better adherence to intervention and greater weight loss than a low-fat diet (McManus et al., 2001). The decision was also based on associations observed between baseline nut consumption and mortality in the PREDIMED trial (Guasch-Ferre et al., 2013) and in American cohorts of nurses and healthcare professionals (Bao et al., 2013).*

(National Institutes of Health) and comply with the CONSORT guidelines for the dissemination of results (Moher et al., 2001).

2. Research team

The trial comprise 6,874 participants, 3,406 were assigned to the intensive intervention group and 3,468 to the control group. Recruitment of participants began in September 2013 and ended in December 2017. The 2 vanguard centers were Navarra-Epidemiology (starting the recruitment in September 2013 and the randomization in October 2013) and Reus (starting the recruitment in November 2013, and the randomization in January 2014). Training of dietitians for the other recruiting centers took place in December 2013. Initially, our recruitment objective with 20 centers was that they each recruiting an average of 300 participants. But, In order to increase the possibility of meeting target numbers of participants recruited by December 2017, in November 2014, the Steering Committee accepted the inclusion of three additional recruiting centers [Universidad Miguel Hernández, Sant Joan d'Alacant, Alicante (IP: Jesús Vioque); Universidad de Jaén, Jaén (IP: Miguel Delgado Rodríguez) y; Universidad de León, León (IP: Vicente Martín)]. The final sample included 6,874 participants distributed in 23 centers.

To launch the trial, we have set up a team of leading researchers with experience in diet and lifestyle interventions and productive and well-documented collaborative research careers in nutrition, evaluation of physical activity, internal medicine, cardiology, endocrinology, primary health care, epidemiology, and basic sciences.

The prior experience gained by the 11 PREDIMED recruiting centers is one of our most valuable assets for the PREDIMED-PLUS trial. Also, by incorporating other centers of scientific excellence with proven experience in nutritional intervention clinical trials (some of which also belong to the background research network of the PREDIMED trial, CIBEROBN), the correct development of the PREDIMED-PLUS trial will be guaranteed and feasibility will be improved.

At the same time, subprojects will be devised so that all participating groups can develop their own specific nutritional research activities. This will also serve to further interest in the project and enhance scientific output.

3. Preliminary studies: the PREDIMED trial

In this section we present a summary of the methodology and key findings of the **PREDIMED** trial (Estruch et al., 2006; Zazpe et al., 2008; Martínez-González et al., 2012; Estruch et al., 2013), which was conducted in the context of CIBEROBN and the

PREDIMED network (RD06/0045). PREDIMED was a multicenter, parallel group, trial with three intervention groups (see www.predimed.es). In 2006, the results of a pilot study were published that evaluated the effects at three months of the three interventions on classical and emergent cardiovascular risk factors in the first 772 participants (Estruch et al., 2006). The design and methods of the PREDIMED trial have been described elsewhere (Martínez-González et al., 2012). Participants in the study were men aged 55-80 and women aged 60-80 without CVD at the beginning of the study but with a high risk of CVD due to the presentation of type 2 diabetes or at least three of the following six cardiovascular risk factors: smoking, high blood pressure, high LDL cholesterol level, low HDL cholesterol level, overweight or obesity, and family history of early coronary heart disease.

Candidates for the trial were recruited at primary care health centers. Eighty-nine per cent of those invited to participate agreed to do so and signed the corresponding informed consent form. The final sample size for the trial was 7,447 participants. The protocol was approved by the ethics research committees of all study centers and registered at the Clinical Trials Register in London (ISRCTN35739639).

The participants were randomly assigned in a 1:1:1 ratio to one of the following three dietary intervention groups: 1) an energy-unrestricted **Mediterranean diet** supplemented with **extra-virgin olive oil**; 2) an energy-unrestricted **Mediterranean diet** supplemented with **nuts**; or 3) an energy-unrestricted **control** diet with advice on how to follow a low-fat diet.

At the beginning of the study and quarterly thereafter, dietitians conducted individual and group dietary-training sessions (separately for each group) with a maximum of 20 participants per group (Zazpe et al., 2008). At each session of the Mediterranean diet groups, a 14-item questionnaire (Martínez-González et al., 2004; Schroeder et al., 2011) was used to assess participants' adherence to the Mediterranean diet. At each session of the control group, a 9-item questionnaire was used to assess participants' adherence to the control diet. In this way the diets could be personalized and appropriate dietary changes could be negotiated individually.

Participants in the two **Mediterranean-diet** groups received either **extra-virgin olive oil** (1 liter per week for the participant and his or her family) or 30 g of **mixed nuts** per day (15 g of walnuts, 7.5 g of hazelnuts, and 7.5 g of almonds) at no cost and in accordance with their randomly chosen group, while those in the control group received small non-food gifts throughout the trial. At no point during the intervention was calorie restriction advised or increased physical activity encouraged.

Information on the primary end points (cardiovascular death, non-fatal stroke, or acute non-fatal myocardial infarction) was obtained through continuous contact with the participants, contact with primary healthcare physicians, *ad hoc* annual reviews of medical records conducted by a medical team at each center, and annual consultation of the National Death Index. The data were analyzed on an intention-to-treat basis. Participants were followed for a median of 4.8 years.

Participants in the two Mediterranean diet groups increased their adherence to the diet, as reflected in an average increase of 2 points on the 14-point dietary-screening questionnaire throughout the duration of the study. These participants also had higher average scores than those in the control group on all items except red and processed meats and sugary soda drinks, which were discouraged for all three intervention groups.

After a median follow-up of 4.8 years, 288 participants suffered a major cardiovascular event. In comparison with the control group, the multivariable-adjusted hazard ratios were 0.70 (95% confidence interval [CI], 0.54 to 0.92) for the group assigned to the Mediterranean diet with extra-virgin olive oil and 0.72 (95% CI, 0.54 to 0.96) for the group assigned to the Mediterranean diet with nuts.

FIGURE 1. Cumulative incidence of major cardiovascular events (cardiovascular death, non-fatal myocardial infarction, and non-fatal stroke) by randomly assigned group (n=7,447).

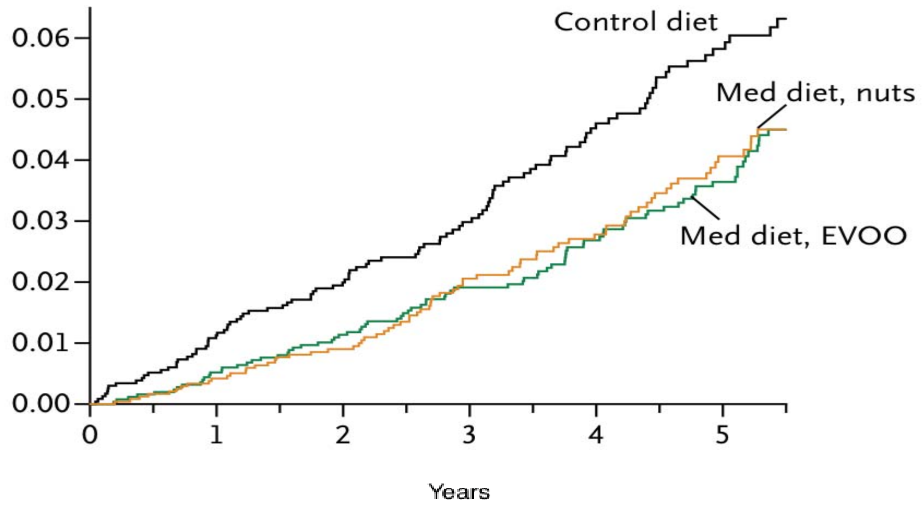

Number at risk						
Control group	2450	2268	2020	1583	1268	946
MeDiet+EVOO	2543	2486	2320	1987	1687	1310
MeDiet+Nuts	2454	2343	2093	1657	1389	1031

4. Proposed trial (PREDIMED-PLUS) and eligibility criteria

Candidates for the PREDIMED-PLUS trial are adults aged 55-75 for men and 60-75 for women with a body mass index ≥27 and <40 kg/m^2 who meet at least three criteria for the metabolic syndrome (Alberti et al., 2009). These criteria must be taken into consideration in view of recent evidence of the beneficial role of the Mediterranean diet on the metabolic syndrome (Kastorini et al., 2011; Salas-Salvadó et al., 2008), insulin resistance and diabetes (Salas-Salvadó, 2014), especially when accompanied by a program of physical activity for endurance (Fernández et al., 2012). Approximately, 50% of the study population is made of women and diabetic participants do not exceed 27% of the total cohort. Individuals who participated in the PREDIMED trial was not eligible to participate in PREDIMED-PLUS.

4.1. Exclusion criteria:

- Illiteracy or inability/unwillingness to give written informed consent or communicate with study staff.
- Institutionalization (the participant is a permanent or long-stay resident in a care home).
- Documented history of previous CVD, including: angina; myocardial infarction; coronary revascularization procedures; stroke (ischemic or hemorrhagic, including transient ischemic attacks); symptomatic peripheral artery disease that required surgery or was diagnosed with vascular imaging techniques; ventricular arrhythmia; uncontrolled atrial fibrillation; congestive heart failure (New York Heart Association Class III or IV); hypertrophic myocardiopathy; and history of aortic aneurism >=5.5 cm in diameter or aortic aneurism surgery.
- Active malignant cancer or history of malignancy within the last 5 years (except non-melanoma skin cancer).
- Inability to follow the recommended diet (for religious reasons, swallowing disorders, etc.) or to carry out physical activity.
- A low predicted likelihood to change dietary habits according to the Prochaska and DiClemente Stages of Change Model (Nigg et al., 1999).
- Inability to follow the scheduled intervention visits (institutionalization, lack of autonomy, inability to walk, lack of stable address, travel plans, etc.).
- Inclusion in another program that provides advice on weight loss (> 5 kg) in the six months before the selection visit.
- History of surgical procedures for weight loss or intention to undergo bariatric surgery in the next 12 months.
- History of small or large bowel resection.

• History of inflammatory bowel disease.
• Obesity of known endocrine origin (except for treated hypothyroidism).
• Food allergy to any component of the Mediterranean diet.
• Immunodeficiency or HIV-positive status.
• Cirrhosis or liver failure.
• Serious psychiatric disorders: schizophrenia, bipolar disorder, eating disorders, or depression with hospitalization within the last 6 months.
• Any severe co-morbidity condition with less than 24 months' life expectancy.
• Alcohol abuse or addiction (or total daily alcohol intake >50 g) or drug abuse within the past 6 months.
• History of major organ transplantation.
• Concurrent therapy with immunosuppressive drugs or cytotoxic agents.
• Current treatment with systemic corticosteroids.
• Current use of weight loss medication.
• Concurrent participation in another randomized clinical trial.
• Patients with an acute infection or inflammation (e.g. pneumonia) will be allowed to participate in the study 3 months after resolution of their condition.
• Any other condition that may interfere with adherence to the study protocol.

5. Recruitment and retention strategies

Medical doctors from primary care centers associated with the recruiting centers recruited the participants. The mission of the primary care physicians was to ensure a high recruitment rate and an almost 100% diligence in the revision of medical records and collection of clinical information on events during follow-up. As the physicians involved in the recruitment process are also responsible for the participants' medical care, no potential ethical conflict regarding confidentiality exists when identifying suitable candidates or reviewing medical records. Participants' eligibility criteria and demographic data were collected from the medical records at the primary care centers, which are entirely computer-based. This was done at a pre-screening evaluation stage before the potential participant is contacted. Candidates were interviewed briefly by telephone, informed about the study, and invited to attend a screening visit at the recruiting center.

In this first formal visit (first screening visit), the candidates were explained the purpose and characteristics of the study and, if they agreed to participate, they were asked to sign a written informed consent. Our experience with the PREDIMED trial showed that over 95% of eligible candidates approached in this way agreed to participate

(Martínez-González et al., 2012; Estruch et al., 2013). Also, in the PREDIMED trial, which also included a long-term lifestyle intervention program (median follow-up time of 4.8 years), overall retention rate was above 90% (Estruch et al., 2013).

6. Informed consent/Ethics Committee

The institutional review boards of all the recruiting centers approved the study protocol. As described and detailed below, all participants signed informed written consent forms.

7. Launch of the trial

The proposed calendar was as follows:

- Between August 2013 and January 2014, the dietitians and nursing staff were hired and trained to deliver the trial's protocol.
- Between August 2013 and May 2014, eligible candidates began to be called, interviewed and invited to participate in the trial.
- Next, the first evaluation and intervention visits took place with randomly distributed selected participants.
- The recruitment period will end on December 2016.

7.1. The aim of the initial stage of the trial (telephone calls and interviews) was to evaluate the willingness of each candidate to participate in the study, comply with the proposed intervention, and lose weight. In addition, they were thoroughly screened in order to ensure that the eligibility criteria were met and evaluated the probability that they:

a) attend the scheduled sessions,

b) complete the protocol's assessment tools, i.e., the self-monitoring and recording of lifestyle and food habits and, most importantly,

c) change their dietary habits in accordance with the Stages-of-Change model (Nigg et al., 1999), as occurred in the PREDIMED trial.

7.2. The run-in period (for evaluation prior to randomization) lasts four weeks. It comprised an initial screening visit, a phone call at 2 weeks, and a final evaluation visit.

7.2.1. The first screening visit (45-60 min) comprised:

a) administration of a questionnaire on inclusion and exclusion criteria. Candidates who were deemed eligible to participate in the trial continue to the next stage.

b) explanation of the study, distribution of the **study information sheet**, and completion of the **informed consent forms** (these are essential for inclusion in the trial). Eligible candidates were asked to sign two informed consent forms: one for participation and analysis of general variables and one for the collection of DNA for genetic analyses. All procedures and anticipated time commitment were explained in detail. Candidates were also told that, if they did not satisfy the eligibility criteria, they would be excluded from the study. The informed consent form includes a statement allowing researchers to review the participants' medical records throughout the trial at both the primary care centers and reference hospitals in order to ascertain the occurrence of any events.

c) performance of ECG and recording of height, weight, waist circumference and blood pressure.

d) distribution of a leaflet containing general recommendations on managing the metabolic syndrome.

e) Distribution of a 3-day food record questionnaire (2 working days and 1 weekend day), a leisure-time physical activity questionnaire, and a self-measurement chart in which participants self-record their weight, waist circumference and hip circumference (participants are given a tape measure). Dietitians gave instructions on how and when to complete the food record and physical activity questionnaires and how to record their weight, waist and hip measurements (once a week during the trial).

f) Distribution of the clinical psychopathological questionnaires (Beck Depression Inventory (BDI-II), multidimensional scale of weight locus of control, eating disorders diagnostic criteria, and quality of life scale (SF-36)) to be completed at home (see below).

Participants were asked to return their completed questionnaires at the third screening visit (see below).

7.2.2. Second screening visit. After 2 weeks, participants received a telephone call to assess their change in weight and remind them to bring to the next screening visit their completed food record, and physical activity questionnaires, self-measurement chart, psychopathological questionnaires, and quality of life scale.

7.2.3. Third screening visit. This evaluation visit on completion of the four-week run-in period (30 min) included:

a) Collection by the dietitian of the participants' food record and physical activity questionnaires, self-measurement charts, psychopathological questionnaires, and quality of life scales.

b) Measurement by the dietitian of the participants' weight and hip circumferences.

c) Administration and completion of a 143-item food-frequency questionnaire and 5 cognitive-neuropsychological tests, which, unlike the clinical psychopathological questionnaires, were completed in the presence of PREDIMED-PLUS personnel. These 5 tests are: the Mini-Mental State examination, the phonological verbal fluency test, the reverse digits test, the trail making test, and the clock test (see below).

d) Explanation to participants that they would be informed by telephone if they had been selected to participate in the trial.

e) Explanation of night-time fasting for *in situ* extraction of blood sample and first morning urine sample, and of basal evaluation immediately after randomization for candidates who were chosen to participate in the trial.

Only candidates who satisfied the following four criteria were selected and randomly assigned to one of the two intervention groups:

1) Full attendance at the two previous sessions, at the scheduled times and having answered the telephone call;

2) Correct completion of the clinical psychopathological questionnaires (Beck Depression Inventory (BDI-II), multidimensional scale of weight locus of control, eating disorders diagnostic criteria, and quality of life scale (SF-36);

3) Correct completion of the food record and physical activity questionnaires;

4) Correct self-recording of at least three weight measurements and three waist-circumference measurements.

Note, that initially the lost of >1.5 kg during the run-in period was one selection criteria. However, In January 2014, the Steering Committee following the advice of the *Data Safety and Monitoring Board* decided to amend the Protocol and omit the 1.5 kg weight loss criterion to be achieved during the run-in period. Such change only affected the first 70 participants who were eligible and randomized in 2 vanguard centers.

The lag time between completion of the run-in phase and the beginning of the intervention ranges from one week to one month.

Exceptionally, depending on the availability of the candidate to attend the Run-in S3 visit, and considering that the 4 mentioned criteria are met, the candidates can be randomized to one of the two intervention groups after completing Run-in S2 visit face-to-face.

8. Initial screening, follow-up visits, and evaluations

Table 1 shows the main data collection measurements and activities by visit.

TABLE 1. The following data are collected per visit in the PREDIMED-PLUS trial.

	RUN-IN PERIOD												
	S1	S2	S3	Baseline	M-6	Y-1	Y-2	Y-3	Y-4	Y-5	Y-6	Y-7	Y-8
**1. ELIGIBILITY QUESTIONNAIRE**	X												
**2. 3-DAY FOOD REGISTER**	e		c										
**3. ANTHROPOMETRIC MEASUREMENTS***	X		X	X	X	X	X	X	X	X	X	X	X
**4. GENERAL QUESTIONNAIRE**				X									
**5. 143-ITEM FFQ**			X		X	X	X	X	X	X	X	X	X
**6. MEDITERRANEAN DIET QUESTIONNAIRE (17/14-Items)****				X	X	X	X	X	X	X	X	X	X
**7. PHYSICAL ACTIVITY QUESTIONNAIRE‡**	e†		c†	X	X	X	X	X	X	X	X	X	X
**8. CHAIR TEST (Physical activity evaluation)**				X	X	X	X	X	X	X	X	X	X
**9. ACCELEROMETERS**			e	X	X	X	X	X	X	X	X	X	X
**10. FOLLOW-UP QUESTIONNAIRE**					X	X	X	X	X	X	X	X	X
**11. ELECTROCARDIOGRAM**	X				X	X	X	X	X	X	X	X	X
**12. BLOOD PRESSURE MEASUREMENT**	X		X	X	X	X	X	X	X	X	X	X	X
**13. BLOOD SAMPLE COLLECTION**				X	X	X		X		X		X	X
**14. MORNING SPOT URINE COLLECTION**				X	X	X		X		X		X	X
**15. NAIL COLLECTION**				X		X		X		X		X	X
**16. COGNITIVE-NEUROPSYCHOLOGICAL TESTS≠**			X				X		X		X		X
**17. PSYCHOPATHOLOGICAL QUESTIONNAIRES€**	e		X			X	X	X	X	X	X	X	X
**18. QUALITY OF LIFE QUESTIONNAIRES≈**	e		X			X		X		X		X	

S: Screening visit; FFQ: Food-frequency questionnaire; M: Month; e: Delivery; c: Collection.

* Anthropometric measurements included: weight, height, waist circumference and hip circumference.

‡ Short version of the Minnesota leisure time physical activity questionnaire; PAR-Q, RAPA (RAPA1 and RAPA2) questionnaires; and the NHS sedentary lifestyle questionnaire

† Long version of the Minnesota leisure time physical activity questionnaire.

**Short questionnaires on adherence to the Mediterranean Diet. The control group uses the same 14-item questionnaire that was used in the PREDIMED trial (Schroeder et al., 2011). The intervention group uses the 17-item energy-restricted Mediterranean diet questionnaire (see below).

≠Mini-Mental Status Examination, clock test, phonological verbal fluency test (animals + P), the reverse series of digits test (WAIS-III), and the trail making test.

€Beck Depression Inventory (BDI-II), multidimensional scale of weight locus of control, eating disorders diagnostic criteria, and SF-36 quality of life scale.

Eligibility: Eligibility for inclusion in the trial was assessed at the beginning of the study. Selected participants must satisfied all the eligibility criteria. Exclusion criteria were also verified.

Anthropometric measurements: Weight and waist circumference are recorded at each visit, with participants in light clothing and without shoes or accessories, using a high-quality electronic scale that is calibrated every 3 months with a unit of known mass. Height is measured at study entrance with a stadiometer. Waist circumference is measured midway between the lowest rib and the iliac crest. Hip circumference is measured at the widest part at the baseline visit and on a yearly basis.

General information: Information on medical history, family history and use of medication is collected at the baseline visit by means of the general questionnaire, using the same protocol as in the PREDIMED trial (See supplementary file in Estruch R et al., New Engl J Med 2013).

Evaluation of food habits and dietary intake: The previously validated 143-item food-frequency questionnaire is administered at the third screening visit and at each annual follow-up visit to evaluate the diet of each participant (Fernández-Ballart et al., 2010). In addition, the 17-item energy-restricted Mediterranean diet questionnaire (see below) is completed at each visit. This questionnaire, which includes several changes with respect to a previously validated tool (Schroder et al., 2011), is used both to assess participants' compliance with the intervention and to guide the individual motivational interviews during follow-up. The control group, on the other hand, is administered the same 14-item questionnaire that was used for the PREDIMED trial (Schröder et al., 2011).

Physical activity: Except for the first screening visit (when participants complete the long Minnesota leisure time physical activity questionnaire), at the beginning of the study, after 6 months, and during the follow-up visits, participants will complete a short version of a previously validated physical activity questionnaire (Elosua et al., 1994; Elosua et al., 2000). Also at these latter visits (baseline, after 6 months, and during the follow-up visits), participants will perform the chair test (30 seconds) in order to evaluate their physical fitness and complete the following questionnaires: the PAR-Q (Physical Activity Readiness Questionnaire), the RAPA (RAPA1 and RAPA2) (Rapid Assessment of Physical Activity), and the NHS (Nurses' Health Study) sedentary lifestyle questionnaire. All these questionnaires are described in the PREDIMED-PLUS website: http://www.PREDIMEDPLUS.COM. Each participant randomly assigned to the intervention group is also provided with a pedometer (Yamax SW200 Digi-Walker) to self-monitor the number of steps walked per day. GENEActiv accelerometers are provided as well to a subset of participants (50% of participants in the intensive

intervention group and 20% of those in the control group) in order to quantify physical activity at baseline, 6 months, 1 year, and each year thereafter. In accordance with an evaluation based on physical status, recommendations on aerobic physical activities and strength training are progressively made and activities to improve balance and flexibility encouraged on completion of physical activity (see protocol of physical activity).

Self-reported information during follow-up. At the follow-up visits, participants are asked about clinical events that may have occurred between visits and information about medication prescribed is updated.

Evaluation of adverse effects: At 6 months and yearly thereafter, participants complete a specific questionnaire to report any adverse effects felt to be derived from the intervention or weight loss.

Electrocardiogram: ECGs are performed at the primary care centers at the first screening visit, 6 months, and at annual follow-up visits thereafter. The ECGs are scanned, stored and registered in the specific database designed for that purpose. The nursing staff at each recruiting center will be responsible for receiving and scanning the ECGs, digitizing their contents, and maintaining the registry and database.

Fasting blood collection: Fasting blood samples are collected at the baseline visits, 6 months, 12 months, 3 years, 5 years, 7 years, and at the final follow-up visit. Conventional analyses [lipid profile (total cholesterol, HDL-cholesterol, LDL-cholesterol, and triglycerides), fasting plasma glucose, blood cell count, serum sodium, potassium, calcium, uric acid, urea, creatinine, albumin, C-reactive protein, erythrocyte sedimentation rate, hemoglobin A1C, liver function tests (serum bilirubin, alkaline phosphatase, alanine transaminase, aspartate aminotransferase and gamma-glutamyltranspeptidase) and optional coagulation tests (prothrombin time, activated partial thromboplastin time and fibrinogen)] are performed at the baseline and follow-up visits according to the trial protocol. The nursing staff at the recruiting centers is responsible for collecting, processing, delivering, storing and preserving the samples, digitizing the information, and maintaining the registry and database for all samples and analyses (see protocol of biological sample collection).

Morning spot urine collection: A sample of morning spot urine is collected *in situ* at the baseline visit, 6 months, 12 months, 3 years, 5 years and 7 years, and at the final follow-up visit. Conventional analyses (albumin and creatinine in urine) are performed at the baseline and follow-up visits according to the trial protocol. The nursing staff is responsible for collecting, processing, delivering, preserving, recording, and maintaining the samples.

Nail sample collection: Nail samples are collected *in situ* at the baseline visit, 12 months, 3 years, 5 years and 7 years and at the final follow-up visit. Patients are asked to attend the visits without having cut their toenails. Using toenail clippers, the patients' nails are cutted and placed in labelled zip-lock bags.

Neuropsychological and Quality of Life evaluation:

This evaluation includes three parts:

A) Cognitive Function

B) Quality of life

C) Psychopathology

At baseline and every two years thereafter (2-, 4-, 6- and 8-year follow-up visits), participants complete a battery of 6 tests of *Cognitive Function* (see below, section A). The first (Mini-Mental State Examination (MMSE) is a general screening cognitive test, while the other 5 tests explore different cognitive domains and are aimed at assessing changes in cognitive performance. The *Cognitive Function* tests will be alternated with the *Quality of life* tests (see section B), so that in even years the 6 tests of *Cognitive Function* will be collected and in odd years only the quality of life test will be administered (see below, section B). The 2-year lapse between sequential cognitive tests will reduce bias due to a "learning" effect.

The *Quality of Life* scales (Short -Form 36 or SF -36, see section B) are collected from all participants at the beginning of the study and in odd years thereafter (after 1-, 3-, 5-, and 7- year follow-up visits), while the psychopathology questionnaires (see below, section C) are collected at the beginning of the study and annually.

All instruments included in the *Cognitive* battery (A), *Quality of Life* (B), and psychopathology (C) have been standardized for the Spanish population in the age range of the study. The complete battery of cognitive, quality of life, and psychopathology examinations includes the following tests:

A) *Six cognitive neuropsychological tests (lasting 16 minutes, to be completed in face-to-face interviews)*:

1) MMSE (Folstein et al, 1975);

2) Semantic verbal fluency test: “animals in 1 minute” (Ramier and Hécaen, 1970, 1977; Benton et al., 1994);

3) Phonemic verbal fluency test: “words in 1 minute starting with the letter ‘p’ (Benton et al., 1994);

4) Verbal and visual working memory: reverse digits test (WAIS-III), Wechsler, 1997):

5) Trail Making Test (Reitan, 1973);
6) Clock test (Clock drawing test or CDT).

Normative data for these tests in the Spanish population have been published by Peña-Casanova et al. (2009a, 2009b). These six tests are collected in the run-in period and each even follow-up year thereafter (years 2, 4, 6 and 8). These six tests, which take roughly 16 minutes to complete, are administered by PREDIMED-PLUS personnel at the third screening visit and each even follow-up year thereafter (years 2, 4, 6 and 8).

B) *One test of Quality of Life (5-10 minutes, to be completed at home)*:
The SF-36 (36-item) quality of life questionnaire (Alonso et al., 1995, 1998; Ware and Gandek 1998) is administered during the run-in period and every odd year of follow-up thereafter (years 1, 3, 5 and 7). In this way, these tests will be alternated with the neuropsychological questionnaires.

C) Three Psychopathological questionnaires (lasting 20-25 minutes, to be completed at home):
1) Beck Depression Inventory (BDI-II) (Beck, Steer and Brown, 1996; Sanz, Navarro and Vázquez, 2003);
2) Multidimensional scale of weight locus control (Wallston, Wallston and DeVellis, 1978);
3) Screening for comorbid eating disorders with diagnostic criteria (DSM-IV-TR; APA, 2000).

The 10 questionnaires above (sections A, B and C) are collected in all participants in the PREDIMED-PLUS study.
The four questionnaires of sections B) and C) are delivered to the participants at the screening visit 1 to be completed at home or at another time outside the study visit. Participants are required to deliver filled-in questionnaires to the recruiting centers within a 15-day period. The same procedure is repeated at follow-up visits when required. The nursing staff at each recruiting center is responsible for collecting, processing, sending, and keeping all the information pertaining to the cognitive tests.
To ensure that graphical data from cognitive tests (drawings of the MMSE and clock and Trail Making tests) are saved for future monitoring. The questionnaires from Group A, B and C tests are collected in paper format, taking advantage of the optical scanning forms for the MMSE, the clock test and the FS-36 designed for this purpose. Once completed,

the MMSE, the clock test and the SF-36 are mailed to the Navarra center (after saving a security photocopy in the recruiting center) to be computerized by optical reading, a time-saving and materially mistake-free procedure.

9. Outcome definition and ascertainment

Clinical events are ascertained by a Clinical Event Ascertainment Committee led by Dr. Fernando Arós of the Vitoria group. The committee members are M. Aldamiz, A. Alonso, J. Berjón, L. Forga, J. Gállego, M. A. García Layana, A. Larrauri, J. Portu, J. Timiraos, and M. Serrano-Martínez. Clinical event ascertainment are based on information collected from the participants' medical records, which each year are reviewed on an *ad hoc* basis by the medical doctors participating in the PREDIMED-PLUS trial. These doctors and the members of the Ascertainment Committee will be blinded to the assignment of participants to the two intervention groups. The reports sent to the Clinical Events Committee contain no personal information about the participants and are identified only by a code.

9.1. <u>Primary outcomes</u>

1. <u>Non-fatal acute coronary syndrome (acute myocardial infarction), non-fatal stroke or cardiovascular mortality</u>.

1.a. Acute myocardial infarction (MI) are defined according to the third universal definition of MI on behalf of the Joint ESC/ACCF/AHA/WHF Task Force (Thygesen et al., 2012) as evidence of myocardial necrosis in a clinical setting consistent with acute myocardial ischemia.

Any one of the following criteria meets the diagnosis for MI:

• Detection of <u>a rise and/or fall of cardiac biomarker values</u> [preferably cardiac troponin (cTn)] with at least one value above the 99th percentile upper reference limit (URL)

AND

• At least <u>one</u> of the following:

(i) Symptoms of ischemia.

(ii) New or presumed new significant ST-segment–T wave (ST–T) changes or new left bundle branch block.

(iii) Development of pathological Q waves in the ECG.

(iv) Imaging evidence of new loss of viable myocardium or new regional wall motion abnormality.

(v) Identification of an intracoronary thrombus by angiography.

Prior MI

Any one of the following criteria meets the diagnosis for prior MI:

• Pathological Q waves with or without symptoms in the absence of non-ischemic causes.

• Imaging evidence of a region of loss of viable myocardium that is thinned and fails to contract, in the absence of a non-ischemic cause.

• Pathological findings of a prior MI

1.b. Stroke is defined as an acute neurological deficit lasting more than 24 hours caused by an abrupt impairment of brain function due to blockage of blood flow in a particular artery supplying the brain (thrombosis or arterial embolism) or a cerebral haemorrhage.

Ischemic Stroke is defined following the updated definition of stroke for the 21st Century: A Statement for Healthcare Professionals from the American Heart Association/American Stroke Association (Sacco RL, et al. 2013) as an episode of neurological dysfunction caused by focal cerebral, spinal, or retinal infarction. Central nervous system (CNS) infarction is brain, spinal cord, or retinal cell death attributable to ischemia, based on:

1. Pathological, imaging, or other objective evidence of cerebral, spinal cord, or retinal focal ischemic injury in a defined vascular distribution;
2. Clinical evidence of cerebral, spinal cord, or retinal focal ischemic injury based on symptoms persisting ≥24 hours or until death, and exclusion of other potential causes such as hypoglycaemia or seizures.

Silent CNS infarction are not considered as a primary end-point if defined as imaging or neuropathological evidence of CNS infarction without a history of acute neurological dysfunction attributable to the lesion.

Haemorrhagic Stroke. Stroke caused by intracerebral hemorrhage is defined as rapidly developing clinical signs of neurological dysfunction attributable to an intracerebral hemorrhage, defined as a focal collection of blood within the brain parenchyma or ventricular system that is not caused by trauma. Stroke caused by subarachnoid hemorrhage is defined as a rapidly developing signs of neurological dysfunction and/or headache because of bleeding into the subarachnoid space, which is not caused by trauma.

Silent cerebral hemorrhage is not considered as primary end-point. It is defined as a focal collection of chronic blood products within the brain parenchyma, subarachnoid space, or ventricular system detected at neuroimaging or neuropathological examination that is not caused by trauma and without a history of acute neurological dysfunction attributable to the lesion.

1.c. Cardiovascular mortality: Includes sudden death and non-sudden cardiovascular death (Buxton AE, et al. 2006).

Sudden (cardiac) death is due to cessation of cardiac activity with hemodynamic collapse, typically due to sustained ventricular tachycardia/ventricular fibrillation. It may be:

— Witnessed instantaneously in a previously stable patient. This may occur with or without preceding signs or symptoms, or may occur immediately following sudden dyspnea, light-headedness, or palpitations.

— Unwitnessed. Patient found dead who at the time of last witnessed contact was in his/her usual state of health without medical complaints or obvious difficulty. This applies to patients dying during sleep.

Non-sudden cardiac death: Includes deaths of patients from acute pulmonary edema with severe, progressive heart failure, cardiogenic shock, or after a recent cardiac surgical procedure.

Non-cardiac vascular death: Includes deaths due to thromboembolic events, stroke, dissecting aneurysm and peripheral artery disease.

2. Weight change. The study nurse records weight at each follow-up visit. The measurement are made according to the study manual of operations and with participants dressed in light clothing and no shoes and accessories.

9.2. Secondary outcomes

1. Total mortality. This endpoint comprises all causes of death, including those from CVD (see point 1c of primary end-point), as well as trauma, renal failure, neoplasia, sepsis, suicide and death of undetermined cause. All deaths should be confirmed by reviewing the National Death Index.

2. Changes in waist circumference. The study nurse measures waist circumference at each follow-up visit according to the manual of operations.

3. Non-ST-segment elevation acute coronary syndrome (unstable angina): The diagnosis of unstable angina is made following the definition of the ESC Guidelines for the management of acute coronary syndromes in patients presenting without persistent ST-segment elevation (Hamm et al, 2011); It requires the presence of at least one of the following clinical characteristics:
   a. Prolonged (>20 min) anginal pain at rest.
   b. New onset (de novo) angina (Class II or III of the Classification of the Canadian Cardiovascular Society).
   c. Recent destabilization of previously stable angina with at least Canadian Cardiovascular Society Class III angina characteristics (crescendo angina).

4. Coronary revascularization (percutaneous or surgical): The two main indications for percutaneous or surgical revascularization are:
   1) Patients with unstable angina or non-ST-segment elevation acute coronary syndrome.
   2) Patients considered likely to benefit from such surgery on the basis of the location and severity of chest pain, the number of vessels affected, and the presence of left ventricular dysfunction (Hamm et al, 2011).

5. Heart failure. Acute and chronic heart failure (HF) is a syndrome in which patients have typical symptoms and signs resulting from an abnormality of cardiac structure or function (McMurray JJ, et al. 2012; Yancy CW, et al. 2013). The cardinal manifestations of HF are dyspnea and fatigue, which may limit exercise tolerance, and fluid retention, which may lead to pulmonary and/or splanchnic congestion and/or peripheral edema.
   5.a. The diagnosis of HF with Reduced Ejection Fraction requires three conditions to be satisfied: 1. Symptoms typical of HF; 2. Signs typical of HF and 3. Reduced ejection fraction (< 40%)
   5.b. The diagnosis of HF with Preserved Ejection Fraction requires four conditions to be satisfied: 1. Symptoms typical of HF; 2. Signs typical of HF; 3. Normal or only mildly reduced left ventricular ejection fraction and non-dilated left ventricle; and 4. Relevant structural heart disease (left ventricular hypertrophy/left atrium

enlargement) and/or diastolic dysfunction

5.c. A Heart Failure event may include hospitalization or an urgent outpatient visit. In this setting the event needs to meet ALL of the following criteria:

- The patient exhibits documented new or worsening symptoms of HF on presentation, including at least ONE of the following: Dyspnea, decreased exercise tolerance, fatigue or other symptoms of worsened end-organ perfusion or volume overload.
- The patient has objective evidence of new or worsening HF, consisting of at least TWO physical examination findings OR one physical examination finding and at least ONE laboratory criterion), including: Physical examination findings considered to be due to heart failure, including new or worsened peripheral edema, increasing abdominal distention or ascites (in the absence of primary hepatic disease), rales/crackles/crepitations at pulmonary auscultation, increased jugular venous pressure and/or hepatojugular reflux, S3 gallop, and clinically significant or rapid weight gain thought to be related to fluid retention
- Laboratory evidence of new or worsening HF, if obtained within 24 hours of presentation, including: Increased B-type natriuretic peptide (BNP)/ N-terminal pro-BNP (NT-proBNP) concentrations OR cardiological evidence of pulmonary congestion OR echocardiographic data of congestion or decreased cardiac output.
- The patient receives initiation or intensification of specific treatment for HF.

6. Peripheral artery disease. Ascertainment is made according to the Inter-Society Consensus for the Management of Peripheral Arterial Disease (TASC II) (Norgren et al., 2007) and ESC Guidelines for the diagnosis of peripheral artery disease (Tendera M, et al., 2011). For participants with intermittent claudication, aged 60-69 with one cardiovascular risk factor, or aged ≥70 years and a resting ankle-brachial systolic pressure index ≤0.90, or an abnormal echo-Doppler examination, magnetic resonance imaging, or arteriography are considered as diagnostic (confirmed case).
7. Venous thromboembolism (VTE): all VTE need to satisfy the standard diagnosis criteria for venous thrombosis or (thromb-) Pulmonary Embolism (PE) in the general population (see below 1-3). The diagnosis should be confirmed by objective imaging techniques (including echography, phlebography, pulmonary computed tomography angiography (angioCTA), NMR, etc.) and not only be based on the clinical suspicion.

Standard diagnosis criteria for VTE in clinical studies (Carrier M et al, 2012):

1. Deep venous thrombosis, defined as the loss of venous compressibility or the inability

of filling the deep vein intraluminal segment at the lower/upper limbs, as detected by echography with venous compression or phlebography, respectively.

- The presence of thrombus at the distal lower limb (distal from the popliteal vein) qualifies for primary VTE only if it is asymptomatic.
- All proximal thrombus qualify for final primary end-point if detected by imaging techniques (echography or radiology), regardless of whether it is or not asymptomatic.

2. Pulmonary Embolism (PE) is defined as:

Contrast pulmonary arteriography:

- Defects in intraluminal filling, as contrasted with two projections.
- Sudden stoppage of the contrast in one or several vessels with a diameter greater than 2.5 mm
- Pulmonary scintigraphy based on ventilation/perfusion (V/Q):

o A V/Q-pulmonary scintigraphy with high probability of PE in patients with no low clinical probability of PE.

- Pulmonary angiography using computed tomography:

o Defects in filling sub-segmental or more proximal vessels

3. Fatal PE is defined as:

- Death exclusively caused by PE and/or its confirmation at autopsy or using radiology techniques

Important considerations:

a) Superficial venous thrombophlebitis should not be described as VTE.
b) It is highly recommended to describe VTE according to the anatomic position:

- Lower limbs
- Upper limbs
- Pulmonary embolism
- Others: vessels at the splanchnic level, cerebral veins, etc.

c) The description of the VTE is highly convenient (for instance, distal to popliteal vein vs. proximal VTE; sub-segmental level vs. central PE)
d) VTE associated with a central catheter (for instance, deep venous thrombosis at the upper limbs) should be reported separately.
e) Incidental VTE should be differenced from any other symptomatic events.

<u>8. Atrial fibrillation (AF)</u>: AF is defined following the Guidelines of the American College of Cardiology Foundation/American Heart Association Task Force on Practice Guidelines together with the European Society of Cardiology, the European Heart Rhythm Association, and the Heart Rhythm Society (Camm AJ, et al. 2010; Fuster V,

et al. 2011), as a cardiac arrhythmia with the following characteristics:

(1) The surface ECG shows 'absolutely' irregular RR intervals, i.e., RR intervals that do not follow a repetitive pattern.

(2) There are no distinct P waves on the surface ECG. Some apparently regular atrial electrical activity may be seen in some EKG leads, most often in lead V1.

(3) The atrial cycle length (when visible), i.e., the interval between two atrial activations, is usually variable and <200 ms (>300 bpm).

9. Type 2-diabetes. New-Onset Type 2 Diabetes cases are diagnosed following the recommendations of the American Diabetes Association:
    1. HbA1C ≥6.5%. This test should be performed in a laboratory using a method that is National Glycohemoglobin Standardization Program (NGSP) certified and standardized to the DCCT assay. OR
    2. Fasting plasma glucose (FPG) ≥126 mg/dL (7.0 mmol/L). Fasting is defined as no caloric intake for at least 8 hours OR
    3. Two-hour plasma glucose (PG) ≥ 200 mg/dL (11.1 mmol/L) during an oral glucose tolerance test (OGTT). This test should be performed as described by the WHO, using a glucose load containing the equivalent of 75 g anhydrous glucose dissolved in water OR
    4. In a patient with classic symptoms of hyperglycemia or hyperglycemic crisis, a random plasma glucose ≥200 mg/dL (11.1 mmol/L).

    In the absence of unequivocal hyperglycemia, results should be confirmed by repeat testing (American Diabetes Association, 2014).

10. Type-2 diabetes complications. Participants are assessed yearly for microvascular complications of diabetes:
    1. Diabetic nephropathy: Kidney disease in diabetes is defined based on the alteration of glomerular filtration rate (GFR) and /or the presence of persistent albuminuria at levels of 30 mg/24 h or more (normal albumin excretion is currently defined as < 30 mg/24 h). GFR is estimated through a quantitative formula, the Modification of Diet in Renal Disease (MDRD) equation, that measures the progression of kidney involvement. Persistent albuminuria is determined by the urine albumin to creatinine ratio (normal <30 mg albumin/g creatinine) in a routine morning urine sample. Because of variability in urinary albumin excretion, two of three morning specimens collected within a 3- to 6-month period should be abnormal before considering a patient to have developed increased urinary

albumin excretion or a progression of albuminuria. The presence of one or two of the above criteria indicates renal disease in these patients and the requirement for appropriate follow-up for progression of renal disease (American Diabetes Association, 2014).

The stages of chronic kidney disease by GFR are reported as follows:

1. Kidney damage with normal or increased GFR ≥90 mL/min/1.73 m^2 body surface area
2. Kidney damage* with mildly decreased GFR 60–89 mL/min/1.73 m^2 body surface area
3. Moderately decreased GFR 30–59 mL/min/1.73 m^2 body surface area
4. Severely decreased GFR 15–29 mL/min/1.73 m^2 body surface area
5. Kidney failure <15 mL/min/1.73 m^2 body surface area or dialysis

2. Diabetic retinopathy: Diagnosed by ophthalmologic examination and/or treatment with laser photocoagulation (American Diabetes Association, 2014).
3. Diabetic polyneuropathy: Diagnosed by clinical symptoms, neurological examination and results of electrophysiological studies of peripheral nerves (American Diabetes Association, 2014).

*Kidney damage defined as abnormalities in urine, blood, or imaging tests.

11. Cancer. All cancers except non-melanoma skin cancer are considered. Cancer cases are coded according to the International Classification of the World Health Organization (International Agency for Research in Cancer, WHO, 2014).

12. Dementia/Alzheimer's disease. Cases are ascertained according to the Recommendations from the National Institute on Aging and the Alzheimer´s Association workgroup (McKhann et al., 2011) or if a diagnosis of dementia is reported by a neurologist.

13. Other dementias: Cases are ascertained according to McKhann et al. 2011 criteria (see below) or if a diagnosis of dementia is reported by a neurologist.

Dementia is diagnosed when there are cognitive or behavioral (neuropsychiatric) symptoms that: 1. Interfere with the ability to function at work or at usual activities; and 2. Represent a decline from previous levels of cognitive functioning; and 3. Are not explained by delirium or major psychiatric disorder; 4. Cognitive impairment is detected and diagnosed through a combination of (1) history-taking from the patient

and a knowledgeable informant and (2) an objective cognitive assessment; 5. The cognitive or behavioral impairment involves a minimum of two domains).

14. Parkinson's disease. Cases are ascertained according to the diagnostic criteria described by Hughes et al (Hughes AJ, et al., 1992) or if reported by a neurologist.

15. Unipolar depression. The diagnosis must be made according to the DSM-V criteria (American Psychiatric Association, 2013). In this, definition major depression, persistent depression, and other depressions included in Depressive Disorders (DSM V) are accepted. Diagnosis of depression made by primary care physicians or psychiatrist in participants treated with antidepressant drugs for more than 6 moths is accepted. If this is the case, ICD 10 (International Statistical Classification of Diseases and Related Health Problems, 10th version) diagnosis of depressive episodes are also accepted. For physicians and psychiatrists not using ICD 10 or DSM V, a positive response to the two questions included in the NICE clinical guidelines is recommended (https://www.nice.org.uk).

16. Osteoporotic fractures. Low-energy fracture is defined as the fracture produced by a same-level fall. Fractures are identified from X-rays reports obtained from at least two radiological reports. High trauma fractures, potentially pathological fractures (e.g., cancer or Paget's disease), or fractures of the head, fingers and toes are not considered (Bliuc D, et al. 2009).

17. Gallstone disease or cholecystectomy: Gallstone disease isdiagnosed according to the findings obtained by imaging techniques including abdominal ultrasonography, computed tomography or magnetic resonance imaging. Diagnosis of cholecystectomy require the corresponding surgical report.

18. Symptomatic gout: Defined following the criteria of the *American College of Rheumatology*. Typically, the disease first presents as arthritis that is acute and episodic, but can be recurrent. Gout can also present as chronic arthritis of one or more joints. This clinical picture is built on a foundation of an excess body burden of uric acid, manifested in part by hyperuricemia, which is defined as serum uric acid levels greater than 7.0 mg/dL (Khanna D, et al. 2012).

19. Transient Ischemic Attack: The diagnosis must be made according to the Scientific Statement of the American Heart Association/American Stroke Association Stroke Council (Easton JD et al. 2009): a transient episode of neurological dysfunction caused by focal brain, spinal cord, or retinal ischemia, without acute infarction demonstrated by neuroimaging, preferably magnetic resonance imaging techniques.

20. Cataracts surgery: Defined by a medical report of cataracts surgery.

21. Surgery for obesity: Defined by a medical report of bariatric surgery.

9.3. Intermediate markers

Changes in nutrient intake and dietary patterns will be determined by changes in the 17-item score of adherence to the energy-restricted Mediterranean diet (intensive intervention group) or the 14-item score (control group) and by changes in food and nutrient intake determined by the 143-item food frequency questionnaire administered during follow-up.

Changes in systolic and diastolic blood pressure, serum lipid concentrations, fasting glucose levels, renal function, uric acid, hemoglobin A1C, C-reactive protein, and liver function are evaluated yearly for the duration of the intervention.

Yearly, are also evaluated the percentage of participants in each group requiring anti-hypertensive, anti-diabetic or lipid-lowering medication, results of ECGs, cognitive function, quality of life, and psychological and neuropsychological questionnaire scores.

10. Randomization procedure (random assignment)

Between one and four weeks after the third screening visit, each recruiting center randomized assign eligible candidates to one of two groups, intensive intervention group or usual care (control) group, using a centrally-controlled, computer-generated random-number system (available at: www.predimedplus.com). The coordinating center was responsible for the randomization procedure by which participants wererandomized assigned with stratification by center, sex, and age group (<65, 65-70, >70 years). Married or unmarried couples were randomized together. The recruiting centers enter the participants' identification criteria into the internet-based system. The system then automatically assigns the participants or partners of participants to their groups. Once this occurs, the group assigned cannot be changed. In the specific cases of couples in

which the spouse was recruited at different times, the last spouse entering the study was assigned (not randomised) to the same study arm than his/her partner in order to ensure high adherence to the intervention and avoid contamination and potential conflicts between partners of the same household.

11. Intervention protocol

All participants will continue to receive usual healthcare from their family doctors and primary care physicians throughout the duration of the trial. At no time will PREDIMED-PLUS personnel deliver medical care.

11.1. Phases of the study for participants assigned to the intensive intervention program with energy-restricted Mediterranean diet (intervention group)

First six months

In addition to the initial visit, participants assigned to the intensive intervention program took part in six individual sessions (I) and 6 group sessions (G) in the first six months (see below):

Month 1		Month 2		Month 3		Month 4		Month 5		Month 6	
G	I	G	I	G	I	G	I	G	I	G	I

Participants also received a third monthly contact by way of a **telephone call** from a dietitian aimed at reinforcing the trial's objectives and answering any queries.

During these first six months, **participants in the intervention group** were encouraged to aim for a reduction in their initial weight of 10% and a reduction in their initial waist circumference of between 5 and 10%. The aim of the trial during these first six months was that the **average** weight loss of the participants in the intervention group was above 8% and the average waist circumference reduction was above 5%. Success in achieving an initial weight loss is known to be a predictor of long-term weight loss. For this reason, all participants were given a chart on which to record and correctly monitor their own weight and waist circumference.

During this period they were encouraged to substitute one meal for low-calorie foods and so were offered a wide range of pleasant alternatives in keeping with the culinary traditions of the Mediterranean diet (see below).

Finally, if by the final visit the participant had still been unable to reach the objectives established for this phase of the trial (month 6), he or she took part in a

motivational interview session with the dietitian in order to determine why he/she had not reached his or her weight-loss goal (see below), try to readdress the situation, and provide appropriate rescue measures.

<u>Months 7-12</u>

Participants attended one individual session (I) and one group session (G) every month in months 7 to 12 of the trial.

YEAR 1	Month 7		Month 8		Month 9		Month 10		Month 11		Month 12	
Months 7-12	G	I	G	I	G	I	G	I	G	I	G	I

They also received a third contact every month by way of a **telephone call** from a dietitian aimed at reinforcing the trial's objectives and answering any queries.

The first-year follow-up visit (see below) coincided with the last individual visit for this phase.

<u>Years 2-6</u>

After the first year and in each of the remaining years of the trial (years 2-6), the participants are attending one quarterly individual session (I) and one monthly group session (G) and are receiving two quarterly telephone calls (T), in accordance with the table below:

**Mont h**	**Month 13**		**Month 14**		**Mont h 15**		**Month 16**		**Month 17**		**Mont h 18**		**Month 19**		**Month 20**		**Mont h 21**		**Month 22**		**Month 23**		**Mont h 24**	
**13-72**	G	T	G	T	G	I	G	T	G	T	G	I	G	T	G	T	G	I	G	T	G	T	G	I

Months 25-36 follow the same procedure as months 13-24, and this procedure will be repeated for the successive years.

The annual follow-up visits (see below) coincide with the last individual session of each year (month 24 above). Throughout the trial, any missed visits are reprogrammed.

<u>11.2. Program of individual and group sessions for participants assigned to the energy-restricted Mediterranean diet</u>

A) I<u>ndividual visits</u>

All individual visits comprise:

i) Distribution of a 17-item questionnaire of adherence to an energy-restricted Mediterranean diet.

ii) Weight and waist circumference measurement by a dietitian.

iii) An individual motivational interview with the dietitian in accordance with the changes in weight observed and the participant's scores on the 17-item adherence to Mediterranean diet questionnaire (see below).

iv) Encouragement to self-monitor weight and waist circumference. Participants are provided charts for self-registering and self-monitoring weight and waist circumference in accordance with the Body Weight Simulator of the National Institute of Diabetes and Digestive and Kidney Diseases (Hall et al., 2011; National Institute of Diabetes and Digestive and Kidney Diseases, 2012). This simulator is also provided to participants at the first individual session with use instructions.

v) Personalized recommendations for increasing physical activity.

B) Group sessions

At these group sessions participants are provided shopping lists, menus, recipes, descriptions of typical components of the Mediterranean diet and advice on lifestyle changes. PREDIMED-PLUS dietitians lead these sessions, which are attended by no more than 20 participants. The sessions comprise:

i) An introductory talk to review the **17-item questionnaire** on adherence to the **energy-restricted Mediterranean diet** (see below).

ii) A 15-minute presentation of the main aspects of the **Mediterranean diet** with audiovisual material prepared by the coordinating center.

iii) Answers to any queries on any aspect of the intervention.

iv) Delivery of the following documents:

- Description of 4-5 low-calorie foods typical of a Mediterranean diet and adapted to the season.
- Weekly food shopping list adapted to the season.
- Weekly meal plan (with detailed menus) adapted to the shopping list.
- Recipes for the suggested menus.

v) Delivery of gratis virgin olive oil (one liter per month) and nuts (125 g per month) to each participant.

vi) At the end of the session participants are reminded of the date of the next session.

11.3. Program of individual and group sessions for participants assigned to the **control group**.

Control group participants receive usual medical care from medical staff at their health institutions. The importance of their attending usual medical visits is stressed to them. Participants receive all the written information related to the **Mediterranean diet** used in the PREDIMIED trial as well as leaflets with general lifestyle recommendations for managing the metabolic syndrome. At the beginning of the study, a group session and an individual session is held at which dietitians deliver documents similar to those used in the PREDIMED trial (shopping lists, recipes, menus, and descriptions of Mediterranean diet components). The dietitians do not provide participants in the control group with instructions on how to lose weight, as this is the responsibility of their family doctors or specialists (usual care). They are also offered a group session every 6 months. At the initial visit and at each 6-month group session, participants are provided free virgin olive oil (6 liters every 6 months) and nuts (750 g every 6 months). In order to encourage compliance with the trial, supply of olive oil and nuts to the participants is contingent on their attending these sessions. The 6-month group sessions include tips on how to follow the **Mediterranean diet** to prevent CVD but advice on calorie restriction, weight loss or increased physical activity is not given and no such objectives are entertained.

11.4. Dietary and lifestyle intervention

The **Intervention Committee** led by Jordi Salas-Salvadó coordinates the dietary and lifestyle intervention. This committee is made up of four coordinators (Jordi Salas-Salvadó, Montse Fitó, Ramón Estruch and Miguel Ángel Martínez-Gonzalez), three of whom are responsible for the three intervention sub-committees: Dietary Intervention (chair: Jordi Salas-Salvadó; members: Nancy Babio, Emilio Ros and Ana Sánchez-Tainta); Physical Activity (chair: Montse Fitó; members: Helmut Schröder, Ascensión Marcos, Miguel A. Martínez-González, Dolores Corella, and Julia Warnberg); and Behavior Treatment (chair: Ramon Estruch; members: Fernando Fernández-Aranda, Cristina Botella and Jordi Salas-Salvadó). This Committee is responsible for designing the lifestyle intervention program for the intensive intervention group and ensuring that it is implemented correctly. Miguel Ruiz-Canela, Miguel A. Martínez-González and Jordi Salas-Salvadó are responsible for ethical considerations.

Dietary recommendations

Many aspects of a diet's quality can affect body weight and the risk of obesity-related illnesses to a greater extent than relative macronutrient content (Mozaffarian et al., 2011;

Ludwig, 2012). In recommendations given to participants, two food groups (A and B) are clearly differentiated:

A) Traditional dietary patterns based on whole foods or minimally processed foods, such as the **Mediterranean diet**, which incorporates many cardioprotective foods and few harmful ones. The consumption of virgin olive oil, nuts (especially walnuts), fruits and vegetables, salads, whole grains, fiber-rich foods and low-fat yogurts have been consistently associated with weight loss or lower weight gain (Martinez-Gonzalez, Bes-Rastrollo, 2011; Mozaffarian et al., 2011).

B) On the other hand, sugar-sweetened beverages, fast foods, refined grain products (especially white bread, which is widely consumed in Spain), white rice, pasta (except for whole-grain pasta), French fries, potatoes, trans fats (mainly present in commercial bakery products in Spain), sweets, cakes, pies, sugar, precooked meals, sausages or cold cuts of processed meats, and patés have been consistently associated with weight gain (Schulze et al., 2006; Mozaffarian et al., 2011).

The main focus of the intensive intervention program (intervention group) lies therefore in the diet's overall quality, with the aim of avoiding foods from the B group and replacing them with foods from the A group.

In addition, by taking into account energy requirements estimates according to the Institute of Medicine equation as well as the participants' basal metabolic rate and level of physical activity, a reduction in energy intake of roughly 600 kcal (about 30% of estimated energy requirements) is envisaged. The energy-restricted Mediterranean diet involves reduced consumption of meat and cold cuts, sugars, white bread, processed fruit juices and sugary beverages, and other foods from the B group, as follows:

**ENERGY-RESTRICTED MEDITERRANEAN DIET**	
**NUTRIENT**	**RECOMMENDED INTAKE**
Calories[1]	Reduction of ≈600 kcal/day (about 30%) from usual intake
Total fat[2]	35-40 % of total calories
Saturated Fatty Acids	8-10 % of total calories
Monounsaturated Fatty Acids	> 20 % of total calories
Polyunsaturated Fatty Acids	> 10 % of total calories
Cholesterol[3]	< 300 mg/day
Proteins[4]	Approximately **20** % of total calories
Carbohydrates[5]	**40-45** % or more of total calories (of low glycemic index)
Sodium chloride	No more than 100 mmol/day (roughly 2.4 g of sodium or roughly 6 g of sodium chloride)
Dietary fiber	**30-35** g/day

1. A reduction in calories of 500 to 1,000 kcal/ day help to achieve a weight loss of 0.5 to 1 kg/week.

Alcohol provides unnecessary calories and displaces the intake of more nutrient-dense foods. Not only does the consumption of alcohol increase the number of calories in one's diet but in epidemiological and experimental studies it has also been associated with obesity. For this reason, although the 17-item adherence to the Mediterranean diet questionnaire contains one item for the consumption of wine, the impact of calories from alcohol on the overall calorie intake ise carefully evaluated and monitored and the consumption of alcoholic beverages other than wine should be avoided.

2. The consumption of wine permitted is one or two glasses per day for women and two or three glasses per day for men. The consumption of other sources of alcohol other than wine is discouraged. Red wine is preferred over other types and it is recommended that the wine be consumed at mealtimes (Gea et al., 2014).

3. Fat restriction involves fat from animal foods. Olive oil and nuts must be the preferred sources of fat.

4. Proteins must be derived first from plant and second from lean animal sources (like fish or poultry).

5. Carbohydrates must be derived from solid, minimally processed and fiber-rich foods with a low glycemic index, such as vegetables, fruits and whole grains, all of which are good sources of vitamins, minerals, and fiber. A diet that is rich in soluble fiber such as oat bran, legumes, and most fruits and vegetables may be effective in reducing blood cholesterol levels and insulin resistance. A diet that is high in all types of fiber may also help to control weight by promoting satiety and maintaining lower levels of total energy intake.

6. During weight loss, attention must be given to maintaining an adequate intake of vitamins and minerals. Maintaining the recommended calcium intake of 1,000 to 1,500 mg/day is especially important for postmenopausal women who may be at risk of osteoporosis.

Participants in the intensive intervention group receive counseling to help them progressively increase their compliance with the following 17 objectives (the **17-item questionnaire on adherence to the energy-restricted Mediterranean diet**). **One point** is awarded for **each objective met**:

1. Use only extra-virgin olive oil for cooking, salad dressings, and spreads.
2. Consume ≥3 portions of fruit per day.
3. Consume ≥ 2 portions of vegetables/garden produce per day (at least 1 portion raw or in a salad).
4. Reduce consumption of white bread to ≤1 serving/day (1 serving = 75 g).
5. Consume whole grain cereals and pasta ≥5 times per week.
6. Consume ≤1 serving (1 serving = 100-150 g) of red meat, hamburgers, or meat products (ham, sausage, etc.) per week.
7. Consume less than 1 serving of butter or cream per week (1 serving = 12 g).
8. Consume less than one sugary beverage or sugar-sweetened fruit juice per week.
9. Consume ≥3 servings of legumes per week (1 serving = 150 g).
10. Consume ≥3 servings of fish or shellfish per week (1 serving = 100-150 g fish, or 4-5 units or 200 g shellfish).
11. Consume < 3 sweets or pastries, such as cakes, cookies, sponge cake, or custard, per week.
12. Consume ≥ 3 servings of nuts (including peanuts) per week (1 serving = 30 g).
13. Consume chicken, turkey or rabbit meat instead of beef, pork, hamburgers or sausages.
14. Use *sofrito* (sauce made with tomato and onion, leek or garlic, simmered in olive oil) ≥ 2 times per week.
15. Do not add sugar to beverages (coffee, tea); instead, replace sugar with non-caloric artificial sweeteners.
16. Reduce consumption of pasta or rice <3 servings per week (unless the pasta or rice are whole grain products).
17. Consume 2-3 glasses of wine (200 mL) per day (men) or 1-2 glasses of wine per day (women). This item will be only promoted among participants who were consumers of alcohol at baseline. Abstainers will never be invited to start consuming wine or any other alcoholic beverage.

The intervention tool for the control group, on the other hand, is the PREDIMED 14-item adherence questionnaire to the non-energy-restricted Mediterranean diet

(Schroeder et al., 2011). However, the 17-item questionnaire is also collected in control group participants for comparison purposes.

Physical exercise recommendations

Participants are encouraged to gradually increase their level of physical activity to at least 45 minutes per day (6 days per week) after 6 months of intervention and their progress is monitored. The physical activity program includes aerobic activities, such as gentle walking or any equivalent activity of moderate intensity and resistance training (Fernández et al., 2012). The dietitians adapt their recommendations to personal preferences and encourage participants to switch between activities with the same metabolic equivalence of tasks.

Psycho-behavioral therapy

Participants are instructed on strategies and provided tools for solving problems associated with consuming high calorie foods and performing sedentary activities. They are encouraged to learn how to recognize lack of control on food intake under stressful or anxious situations and how to exercise self-control.

Recommendations on the use of tobacco

The PREDIMED-PLUS dietitians make no recommendations on the use of tobacco. This is the responsibility of the medical professionals in the primary care centers in accordance with usual medical practice.

Individual motivational interviews

Personal interviews with the dietitian at each individual visit are adapted to the participant's clinical conditions, preferences and beliefs. Dietary changes are introduced in order to achieve the recommended diet for each participant and suitable lifestyle changes are incorporated. Objectives are accorded via a negotiated agreement between the two parties (dietitian and participant) depending on what participants consider to be an attainable goal. The main objective is to change not only the participant's consumption of certain foods but also his or her overall dietary pattern. Attention can vary between changing portion sizes, changing the frequency of dietary components, and changing cooking methods.

Achievements made in the previous months, however minor, are always considered an essential support mechanism for improving self-esteem and self-reward. Special care

is taken to ensure that participants do not receive contradictory dietary advice from health professionals external to the PREDIMED-PLUS trial.

As described, each participant receives oral and written information on the food components and culinary customs of the **energy–restricted Mediterranean diet**, as well as charts for self-registering and self-monitoring changes in weight and waist circumference at each visit.

Participants who during the active weight-loss phase have observed a lower weight loss than expected or who have not maintained the weight loss they had achieved receive special reinforcement and a series of rescue measures to help them achieve weight loss and weight-loss maintenance. In such cases, agreements are negotiated between the dietitians and the participants.

Role of the dietitians

The PREDIMED-PLUS dietitians are directly responsible for the dietary intervention. They have been specifically trained and certified to deliver the PREDIMED-PLUS intervention protocol. All intervention procedures are conducted in accordance with the PREDIMED-PLUS operation's manual. Throughout the study, annual meetings will be held at which the dietitians will discuss any problems they may have identified and find possible solutions. The dietitians and trial coordinators will discuss any problems arising during the trial, thus ensuring a process of continuous feedback.

12. Training and calibration procedures

A general trial operations manual and staff training documents are set forth to ensure standardized procedures across the various recruiting centers. Before implementation of the protocol, study personnel attended a 3-day training course at the coordinating center. This included theoretical and practical group discussions with experts on lifestyle interventions in order to convey the goals of the study, develop all the specific aspects involved in implementing the intervention, and impart training on the informed-consent process, anthropometric and blood pressure measurements, data collection by optical scanning or online systems, and biological sample collection and processing. The abilities of all contracted personnel were evaluated at personal interviews during this training course. The research team stressed the importance of creating a trusting and empathic relationship with the participants and paying attention to their individual needs in order to maximize their motivation and retention into the trial. Study personnel keep a copy of the operations manual detailing all the training points. In addition, all the personnel responsible for the intervention are attending annual meetings and are in

constant contact with the principal investigators in order to ensure standardized implementation of the trial protocol. In accordance with the protocol, all scales and other measurement instruments are periodically calibrated.

13. Retention and compliance with strategies and supervision procedures

The recruitment of participants and the **compliance rates for the intervention strategies** are crucial to the success of a trial of this nature. For this reason, a run-in (pre-evaluation) period **prior to randomization** was planned: only participants who adhere to **all** the requirements of the protocol during the run-in period are accepted into the trial. The lag time between the end of the run-in period and the start of the intervention ranges from one week to one month.

The researchers involved in this trial have already gained invaluable experience in managing long-term trials through the PREDIMED trial, wherein they developed strategies for ensuring participants' compliance with the protocol and encouraging their **long-term retention**. We understand that this is a particularly sensitive aspect for participants in the control group. Therefore, at each group session of both the intensive intervention group and the usual care group, virgin olive oil and mixed nuts is provided at no cost to all participants. Our experience in the PREDIMED trial showed that such gifts, especially the virgin olive oil, greatly helped to encourage participants' retention. Other retention strategies include providing feedback on findings during follow-up to the participants' usual health-care providers as well as supplying other non-coercive material incentives for both groups. Additionally, in the intensive intervention group, where significant weight-loss is anticipated, contact with participants is ongoing and flexible interventions and rescue measures tailored to the participants' needs are implemented. Self-control, self-reward and self-monitoring techniques are also reinforce participants' compliance with the intervention. Finally, the intervention is adapted to the needs of the participants, which should encourage compliance.

14. Biological samples and laboratory procedures

The nursing staff contracted at each recruiting center is responsible for collecting, processing and storing the biological samples in freezers at a temperature of -80ºC. Blood samples are collected at the recruiting centers in the same way as they are collected at the participants' usual healthcare centers. In addition, 55.5 ml of blood are extracted and collected in the following tubes: two 10 ml K2E EDTA tubes; one 4.5 ml citrate tube; and two 10 ml and 6 ml gel serum separator tubes. The serum, citrate plasma and EDTA plasma samples are distributed in aliquots of 200 µl and 500 µl and

stored at -80°C for future analyses at the recruiting centers. For the intensive intervention group, the biochemical measurements are performed in a blind fashion and in the same batch for consecutive samples of each participant. Each recruiting center has an ultra-low-temperature freezer with enough capacity to store biological samples until final delivery. All biological samples are processed at each recruiting center no later than one hour after extraction. During transportation from the primary care centers to the laboratories, the biological samples are stored at 4ºC in a portable cooler. Urine tests are conducted at the recruiting centers in the same way they are conducted at the participants' usual healthcare centers in accordance with the specific PREDIMED-PLUS protocol for collecting biological samples.

15. Quality control

The general database for the PREDIMED-PLUS trial is managed and maintained by the research group of the IMIM Institute (CIBERobn). The food-frequency questionnaires and the food records, as well as quality of life test, clok and Minimental tests are processed and managed at the University of Navarra. Data collected from accelerometers to measure physical activity are processed at the Malaga recruiting center in collaboration with the CSIC/UAM. These data are sent every three months to the IMIM, where they are incorporated into the General Database. Event detection data, collected from information gathered during the intervention and at the follow-up visits, are introduced into specific forms at the recruiting centers, preferably using online systems, and sent at least once a month to the data manager at the IMIM, who sends monthly reports of missing or inappropriate entries back to the recruiting center coordinators to solve any raised queries. The IMIM also sends monthly reports to the different recruitment centers with the sumsample of each node. The steering committee has been set up to ensure the quality of the project and correct any flaws or divergences. This committee was made up of Jordi Salas-Salvadó (PREDIMED-Plus coordinator), Miguel Angel Martínez-González, Ramón Estruch, Montserrat Fitó, Emilio Ros, and Dolores Corella. In december 2016, Dr. Francisco Tinahones (Hospital de Málaga) was incorporated as the seventh member of the Steering Committee.

At every annual PREDIMED-PLUS meeting, the IMIM conduct a current data management information session. An annual summary is sent to the recruiting center coordinator for distribution to all groups.

To reduce data entry expenses and speed up processing, the questionnaires and data forms are processed by optical scanning or by online data transfer forms. The data forms are entered in duplicate and missing data checks are performed. All forms sent to

another recruiting center must be photocopied and stored at that center. After data entry, cross-form edit checks are performed and any data inconsistencies are identified. To detect any still-unsolved problems, audits are run periodically at each recruiting center. Reports are drafted to summarize any problems in the database and provide an additional step to ensure the quality and accuracy of the data. To minimize the possibility of error, a detailed operations manual has been prepared.

Annual staff training meetings are conducted. The data manager and an audit committee evaluate the performance of each recruiting center. Appropriate new procedures and corrective measures are implemented whenever deficiencies are noted. Until the end of the trial, all field centers are masked to the trial outcome data except for the two trial statisticians, one in Navarra (M.A. Martínez-González) and one at the IMIM in Barcelona (Joan Vila), who will always perform the statistical analyses in duplicate with two statistical analysis units. Because of the nature of the trial, however, the dietitians at each field center know which intervention has been assigned to each participant. The medical doctors who prepare the annual report on the *ad hoc* review of the participants' medical records are blinded to group assignment, as the **Clinical Event Ascertainment Committee**. The members of the Steering Committee, who attend the meetings of the Data and Safety Monitoring Board, also remain blinded to the results of intermediate analyses throughout the trial. The Steering Committee is informed of the total number of events observed but not of the groups in which they occurred.

STATISTICAL ANALYSIS PLAN

All analyses will be performed on an intention-to-treat basis. Miguel A. Martínez-González will be the senior statistician responsible for the statistical analysis plan. All major data analyses will be conducted under his supervision. Statistical analyses for the main aims of the study will be also conducted in duplicate by the center at IMIM, Barcelona (responsible statistician at IMIM: Joan Vila).

1. Analysis of the effect of the intervention

Since the data take into account time to the event, Cox's regression models will be used to determine the effect of the intervention on the incidence of cardiovascular events. For changes in weight and waist circumference, mixed models of analysis of variance and generalized estimating equations (GEE) will be used. These models will include the following adjustment covariates:

1) All factors that, according to the scientific literature, are related to the event; and
2) All factors that reach statistical significance in univariate analyses.

In these models we will evaluate: 1) the proportional hazards assumption; 2) the linearity of the continuous variables, using smoothing spline methods; 3) the effect of extreme observations on the estimation of parameters, by calculating delta-beta values. The use of further approaches (i.e. normalizing transformations, stratified analyses, etc.) will depend on the results obtained above. Given that participants will be clustered by recruiting centers, some degree of correlation structure may be expected. Center will therefore be included as a stratification variable, including frailty estimates, in the Cox regression models. The goodness-of-fit of the models will be examined using the modified Hosmer-Lemeshow test for survival studies. Robust estimators of variance that account for the clustering effect of members of the same household (the second member is not randomized for feasibility reasons) will be used to take into account the intra-cluster correlation. Sensitivity analyses will be conducted after excluding the second (non-randomized) members of the same household. In addition to the stratification by center, all Cox models will be also stratified by sex and educational level.

2. Interim analyses and stopping rules

Data from the PREDIMED-PLUS trial will be analyzed after 3 years of median follow-up, after 5 years of median follow-up, and at the end of the trial. For methodological reasons but especially for ethical ones, suitable follow-up for a trial must include at least one interim analysis (Schulz, Grimes, 2005). However, to preserve an overall alpha error of 0.05, interim analyses have to be penalized. We will use the O'Brien and Fleming boundaries (O'Brien and Fleming, 1979). With this method, the boundaries are stricter at the earlier stages of the study than at the later ones. Applying this rule leads to the following p values for stopping the trial:
First interim analysis (median follow-up: 3 years); threshold p value: 0.0005.
Second interim analysis (median follow-up: 5 years); p value: 0.014.
Final analysis (median follow-up: 8 years); p value: 0.045

These p values should not be considered compulsory for stopping the trial but guidelines for guaranteeing the security of the data. In making their decision, the Data and Safety Monitoring Board must take into account, for example, the size of the effect, the follow-up time at each recruiting center, the heterogeneity between the effects at the recruiting centers, as well as evidence from other current trials and observational studies. All the above must be taken into consideration when deciding either to continue or to interrupt the trial after each interim analysis. Reasons for interrupting the trial include: 1)

convincing evidence of the beneficial effect of the intervention (the trial will be stopped only if the effect of the intervention is great); 2) convincing evidence of a harmful effect from the intervention; 3) results suggesting it is highly unlikely that the proposed hypothesis will be accepted due to, for example, a very small effect of the intervention that dramatically affects the trial's statistical power.

3. Estimations of sample size

We will determine the effect of the intensive weight-loss lifestyle intervention with an energy-restricted **Mediterranean diet** on the two primary outcomes below, assuming a two-tailed alpha error of 0.05.

1. Effect of the intervention on the **incident CVD** (non-fatal myocardial infarction, non-fatal stroke, and cardiovascular death). The cumulative projected incidence after including as primary events all non-fatal acute myocardial infarctions and all microinfarctions with positive high-sensitivity troponin tests after 6 years will be at least 10% in the control group, if we take into account the results of the PREDIMED trial after 4.8 years (which did not include high-sensitivity troponin tests). The hazard ratio (HR) for the combined primary endpoint is anticipated to be 0.70 (Estruch et al., 2013) and will probably be even lower (greater protective effect) if we consider that in the PREDIMED trial no energy restriction was implemented, physical activity was not encouraged, and weight loss was not a target of the intervention. Under these assumptions, therefore, even if the dropout rates were to reach 20%, the required sample size would be 2,400 per group (see Figure 3). To be conservative, initially we aim to recruit 6,000 participants and assign 3,000 participants to each group. However, the final sample included 6,874 participants distributed in 23 centers, 3,406 participants assigned to the intensive intervention group and 3,468 to the control group.

FIGURE 3. Estimation of the sample size required per intervention group in the PREDIMED-PLUS trial

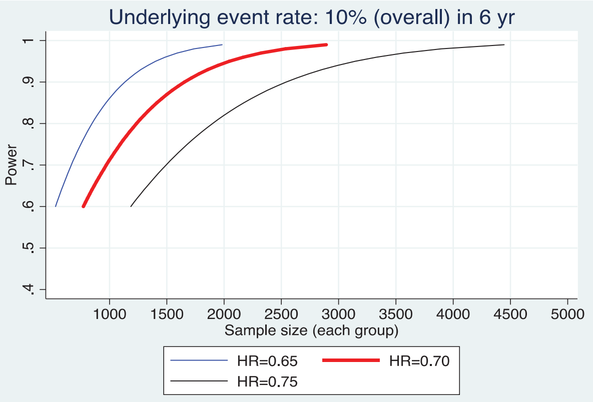

2. Effect of the intervention on **weight change**. Based on previous studies, we can expect a minimum weight change for participants in the control group and a weight loss of 3-4.5 kg for those in the intensive lifestyle intervention group, with a standard deviation of 8 kg (Shai et al., 2008; Sacks et al., 2009; Wing, 2010). If we assume our intervention will have only a small effect on weight change and then calculate sample size according to a weight change of 1 kg in the usual care group, a weight change of 3 kg in the intensive lifestyle intervention group, and a standard deviation of 8 kg, in order to achieve a statistical power of 0.80 we would need a sample size of only 337 in each group. Since the number of participants recruited is much higher than this figure, the statistical power needed to reach this objective is largely guaranteed.

STRENGTHS AND LIMITATIONS

1. Strengths

A. This trial provides a multidisciplinary approach to tackling the serious problem presented by the overweight and obesity epidemic. Our target group comprises obese or overweight adults, who represent an increasing proportion of the general population. For these subjects, an intervention based on a profound lifestyle change incorporating improvements in the dietary pattern, weight loss, behavioral therapy, and increased physical activity can be a novel and useful model for reducing the burden of obesity and associated diseases, thus contributing to the sustainability of the healthcare system. The trial clearly addresses priority objectives of the public healthcare system since it tackles both the principal epidemic of our times (overweight and obesity) and the principal cause of death around the world (CVD).

B. This innovative proposal presents a novel paradigm for nutritional recommendations aimed at achieving weight loss, i.e., a traditional dietary pattern characterized by a moderate-to-high fat content. We believe this new approach will help improve compliance with the intervention and overcome the main challenge of any dietary interventions aimed at fighting overweight and obesity: long-term weight loss maintenance (Shai et al., 2008; Beunza et al., 2010; Romaguera et al., 2010).

C. The intervention is well structured and the trial is suitably designed for determining the effect of the intervention on the main clinical outcomes. Moreover, since the trial is conducted in the context of primary healthcare and incorporates epidemiological, clinical and basic aspects, it has a high capacity for both transferability and reproducibility.

D. The research team includes investigators with invaluable experience in lifestyle intervention trials (e.g., PREDIMED). As these investigators come from a wide range of fields, their work will be complementary and the trial's chances of success will be enhanced. All these reasons, together with the success and achievements of the PREDIMED trial, attest to the viability of this trial proposal.

E. From a strategic perspective, this is a timely proposal since it provides continuity for the collaborative project in which most CIBEROBN centers have participated, i.e., PREDIMED.

2. Limitations

A. Our study enrolled participants aged between 55 and 75 years old. This may preclude generalization to younger age groups.

B. In a large-scale clinical trial, one limitation to consider is participants' dropout rates. However, we hope to ensure compliance in both groups by: a) providing free foods (olive oil and mixed nuts); b) establishing personal relationships with each participant via individual and group sessions; c) administering, at the start of the study, the Prochaska and DiClemente Stages of Change Model, by which a low predicted probability of changing dietary habits will be a criterion for exclusion; and d) establishing, at the start of the study, a one-month run-in period in order to identify and select participants with a greater likelihood of compliance with the protocol and retention into the study (see section 5 and 13).

C. Homogeneity of the interventions is difficult because it is based on three components: diet, physical exercise, and behavior. For this reason we have developed a detailed protocol for implementing the intervention and have established a committee for each intervention component. We also conducted a staff training session at the beginning of the study and will conduct annual follow-up sessions throughout.

COMMITTEES AND GOVERNANCE

The PREDIMED-PLUS **Executive Committee** includes the principal investigators from all the participating centers (see Annex 1). It provides scientific and strategic orientation for decision-making and is responsible for designing, implementing and publishing the study's protocol and guaranteeing the quality of its implementation and management. It determines its own guidelines and approve the criteria and guidelines of the other committees within the study. It convene at least twice a year to discuss and report on the study's progress.

In February 2018, Dr. Julia Wärnberg (School of Health Sciences) replaced the principal investigator of the recruitment center of the University of Malaga, Prof. Enrique Gómez-Gracia, who continues in the trial as a principal investigator of a new support group (A8).

The **Steering Committee**, made up of Jordi Salas Salvadó (Chair), Miguel Angel Martínez-González (PI of the ERC-Advanced Research Grant), Ramón Estruch, Montserrat Fitó, Emilio Ros, Dolores Corella and Francisco Tinahones who was incorporated as the seventh member of the Steering Committee in December 2016, is responsible for ensuring the quality of the project and correcting any flaws or divergences that may be detected.

Data Safety and Monitoring Board

To ensure the smooth running of the trial and the safety of participants, an Independent **Data Safety and Monitoring Board** has been set up. This Board is made up of: Chairman, Meir J. Stampfer (Harvard School of Public Health); members Joan Sabaté (Loma Linda University), Arne Astrup (Copenhagen University), Francisco Fernandez-Avilés (Universidad Complutense of Madrid) and Xavier Pi-Sunyer (Columbia University). The Board has its first meeting in 2015 and is convened at least once a year to review the implementation of the protocol and monitor the trial's progress. It examines the competence of each recruiting center, evaluate their compliance with the study's objectives, and decide whether they may continue in the trial.

In addition, a report is mailed periodically by the PREDIMED-PLUS Steering Committee to the Board members with relevant statistical analyses for judging on the continuation of the PREDIMED-PLUS trial. Throughout the study, the Board members can request any statistical analysis on a blinded or unblinded basis. The Board may recommend termination of the trial at any time if an unacceptable incidence of adverse events or

significant differences in mortality between study groups are observed. The Executive Committee of the PREDIMED-PLUS trial, however, will make the final decision.

SOURCES OF FUNDING AND ADMINISTRATIVE ISSUES

This study was supported by the official funding agency for biomedical research of the Spanish government, Instituto de Salud Carlos III (ISCIII), through the Fondo de Investigación Sanitaria (FIS) that awarded us four coordinated research projects for the 2014-2016 and 2017-2019 annual periods (IP of the coordinated projects Jordi Salas-Salvadó), and for the annual periods of 2015-2017 and 2018- 2020 (IP of the coordinated projects Josep Vidal) ; CIBEROBN, which is co-funded by the European Regional Development Fund, and by the European Research Council (Advanced Research Grant 2013-2018; 340918) grant to Miguel Ángel Martínez-Gonzalez.

Trial's website

http://www.predimedplus.com

Contact's name and address

Prof. Jordi Salas-Salvadó, MD, PhD. Human Nutrition Unit. Faculty of Medicine and Health Sciences, Universitat Rovira i Virgili, C/ Sant Llorenç, 21, 43201 Reus (Spain). Telephone number: +34 977759312; Fax number: +34 977759322. E-mail: jordi.salas@urv.cat.

Prof. Miguel A. Martínez-González, MD, PhD, MPH, Department of Preventive Medicine and Public Health, Facultad de Medicina–Clínica Universidad de Navarra, Irunlarrea 1, 31008 Pamplona, Spain. Phone: +34 636 355 333, E-mail: mamartinez@unav.es

PREDIMED-PLUS Registration

The PREDIMED--Plus trial was registered at the International Standard Randomized Controlled Trial (ISRCT; http://www.isrctn.com/ISRCTN89898870) with number 89898870 and a registration date of 24 July 2014.

REFERENCES

American Diabetes Association. Standards of medical care in diabetes-2014. Diabetes Care 2014;37 Suppl 1:S14-80.

Alberti KG, Eckel RH, Grundy SM, et al. Harmonizing the metabolic syndrome: a joint interim statement of the International Diabetes Federation Task Force on Epidemiology and Prevention; National Heart, Lung, and Blood Institute; American Heart Association; World Heart Federation; International Atherosclerosis Society; and International Association for the Study of Obesity. Circulation 2009;120:1640-5.

Alonso J, Prieto L, Anto JM. [The Spanish version of the SF-36 Health Survey (the SF-36 health questionnaire): an instrument for measuring clinical results]. Med Clin (Barc) 1995;104:771-6.

Alonso J, Regidor E, Barrio G, Prieto L, Rodriguez C, de la Fuente L. [Population reference values of the Spanish version of the Health Questionnaire SF-36]. Med Clin (Barc) 1998;111: 410-6.

American Dietetic Association. Adult Weight Management Evidence-Based Nutrition Practice Guideline. Ver http://www.adaevidencelibrary.com/ topic.cfm?cat=2798 &auth=1, consulted 12/4/2012.

American Psychiatry Association. Diagnostic and Statistical Manual of Mental Disorders, Fifth Edition (DSM-5) Arlington, VA, American Psychiatric Association, 2013.

Bao Y, Han J, Hu FB, et al. Association of nut consumption with total and cause-specific mortality. N Engl J Med 2013;369:2001-11.

Basterra-Gortari FJ, Beunza JJ, Bes-Rastrollo M, Toledo E, García-López M, Martínez-González MA. [Increasing trend in the prevalence of morbid obesity in Spain: from 1.8 to 6.1 per thousand in 14 years]. Rev Esp Cardiol 2011;64:424-6.

Benton AL, Hamsher, K, Sivan AB. Multilingual Aphasia Examination (3rd ed.). San Antonio, Texas: Psychological Corporation; 1994.

Berrington de Gonzalez A, Hartge P, Cerhan JR, et al. Body-mass index and mortality among 1.46 million white adults. N Engl J Med 2010;363:2211-9.

Bes-Rastrollo M, Sabaté J, Gómez-Gracia E, Alonso A, Martínez JA, Martínez-González MA. Nut consumption and weight gain in a Mediterranean cohort: The SUN study. Obesity (Silver Spring) 2007;15:107-16.

Beunza JJ, Toledo E, Hu FB, et al. Adherence to the Mediterranean diet, long-term weight change, and incident overweight or obesity: the Seguimiento Universidad de Navarra (SUN) cohort. Am J Clin Nutr 2010;92:1484-93.

Benkhalti F, Prost J, Paz E, Perez-Jimenez F, El Modafar C & El Boustani E. Effects of feeding virgin olive oil or their polyphenols on lipid of rat liver. Nutrition Research 2002; 22:1067-1075.

Beck A T, Steer R A, y Brown GK. BDI-II. Beck Depression Inventory-Second Edition Manual. San Antonio, TX: The Psychological Corporation, 1996.

Bueno NB, de Melo IS, de Oliveira SL, da Rocha Ataide T. Very-low-carbohydrate ketogenic diet v. low-fat diet for long-term weight loss: a meta-analysis of randomised controlled trials. Br J Nutr 2013;110:1178-87.

Buxton AE, Calkins H, Callans DJ,et al. ACC/AHA/HRS 2006 key data elements and definitions for electrophysiological studies and procedures: a report of the American College of Cardiology/American Heart Association Task Force on Clinical Data Standards (ACC/AHA/HRS Writing Committee to Develop Data Standards on Electrophysiology). Circulation 2006;114:2534-70.

Bliuc D, Nguyen ND, Milch VE, Nguyen TV, Eisman JA, Center JR. Mortality risk associated with low-trauma osteoporotic fracture and subsequent fracture in men and women. JAMA 2009;301:513-21.

Bray F, Znaor A, Cueva P, et al. Planning and Developing Population-Based Cancer Registration in Low- and Middle-Income Settings. IARC Technical Publication No. 43. International Agency for Research on Cancer. WHO, Geneva 2014.

Camargo A, Delgado-Lista J, Garcia-Rios A, et al. Expression of proinflammatory, proatherogenic genes is reduced by the Mediterranean diet in elderly people. Br J Nutr 2012;108:500-8.

Camm AJ, Kirchhof P, Lip GY, et al. ESC Committee for Practice Guidelines. Guidelines for the management of atrial fibrillation: the Task Force for the Management of Atrial Fibrillation of the European Society of Cardiology (ESC). European Heart Rhythm Association; European Association for Cardio-Thoracic Surgery, Europace. 2010;10:1360-420.

Carrier M, Khorana AA, Zwicker JI, Lyman GH, Le Gal G, Lee AY; subcommittee on Haemostasis and Malignancy for the SSC of the ISTH. Venous thromboembolism in cancer clinical trials: recommendation for standardized reporting and analysis. J Thromb Haemost 2012;10:2599-601.

Chrysohoou C, Panagiotakos DB, Pitsavos C, Das UN, Stefanadis C. Adherence to the Mediterranean diet attenuates inflammation and coagulation process in healthy adults: The ATTICA Study. J Am Coll Cardiol 2004;44:152-8.

Chrysohoou C, Skoumas J, Pitsavos C, et al. Long-term adherence to the Mediterranean diet reduces the prevalence of hyperuricaemia in elderly individuals, without known cardiovascular disease: the Ikaria study. Maturitas 2011;70:58-64.

Covas MI, Konstantinidou V, Fito M. Olive oil and cardiovascular health. J Cardiovasc Pharmacol 2009;54:477-482.

Covas MI, Nyyssonen K, Poulsen HE, et al. The effect of polyphenols in olive oil on heart disease risk factors: a randomized trial. Ann Intern Med 2006;145: 333-41.

Dai J, Jones DP, Goldberg J, et al. Association between adherence to the Mediterranean diet and oxidative stress. Am J Clin Nutr 2008;88:1364-70.

de Roos B, Zhang X, Rodriguez Gutierrez G, et al. Anti-platelet effects of olive oil extract: in vitro functional and proteomic studies. Eur J Nutr 2011;50: 553-62.

Defilippis AP, Blaha MJ, Jacobson TA. Omega-3 Fatty acids for cardiovascular disease prevention. Curr Treat Options Cardiovasc Med 2010;12:365-80.

Detopoulou P, Panagiotakos DB, Chrysohoou C, et al. Dietary antioxidant capacity and concentration of adiponectin in apparently healthy adults: the ATTICA study. Eur J Clin Nutr 2010; 64:161-8.

Dinu M, Pagliai G, Casini A, Sofi F. Mediterranean diet and multiple health outcomes: an umbrella review of meta-analyses of observational studies and randomised trials. Eur J Clin Nutr 2018;72:30-43.

DSM-IV-TR; APA, 2000. American Psychiatric Association. (2000). Diagnostic and statistical manual of mental disorders (4th ed., text rev.). Washington, DC.

Easton JD, Saver JL, Albers GW, et al. Definition and evaluation of transient ischemic attack: a scientific statement for healthcare professionals from the American Heart Association/American Stroke Association Stroke Council; Council on Cardiovascular Surgery and Anesthesia; Council on Cardiovascular Radiology and Intervention; Council on Cardiovascular Nursing; and the Interdisciplinary Council on Peripheral Vascular Disease. The American Academy of Neurology affirms the value of this statement as an educational tool for neurologists. Stroke 2009;40:2276-93.

Elosua R, Garcia M, Aguilar A, Molina L, Covas MI, Marrugat J. Validation of the Minnesota Leisure Time Physical Activity Questionnaire In Spanish Women. Investigators of the MARATDON Group. Med Sci Sports Exerc 2000;32:1431-7.

Elosua R, Marrugat J, Molina L, Pons S, Pujol E. Validation of the Minnesota Leisure Time Physical Activity Questionnaire in Spanish men. The MARATHOM Investigators. Am J Epidemiol 1994;139:1197- 209.

Esposito K, Marfella R, Ciotola M, et al. Effect of a Mediterranean-style diet on endothelial dysfunction and markers of vascular inflammation in the metabolic syndrome: a randomized trial. JAMA 2004;292:1440-6.

Esposito K, Kastorini CM, Panagiotakos DB, Giugliano D. Mediterranean diet and weight loss: metaanalysis of randomized controlled trials. Metab Syndr Relat Disord 2011;9:1-12.

Estruch R, Martínez-González MA, Corella D, et al. Effects of a Mediterranean-style diet on cardiovascular risk factors. A randomized trial. Ann Intern Med 2006;145:1-11.

Estruch R, Martínez-González MA, Corella D, et al. Effect of a high-fat Mediterranean diet on bodyweight and waist circumference: a prespecified secondary outcomes analysis of the PREDIMED randomised controlled trial. Lancet Diabetes Endocrinol 2016;4:666-76.

Estruch R, Ros E, Salas-Salvado J, et al. Primary prevention of cardiovascular disease with Mediterranean diets: the PREDIMED trial. 2013;368:1279-90.

European Stroke Organisation (ESO) Executive Committee and ESO Writing Committee. Guidelines for management of ischaemic stroke and transient ischaemic attack 2008. Cerebrovasc Dis 2008;25:457-507.

Fernández JM, Rosado-Álvarez D, Da Silva Grigoletto ME, et al. Moderate-to-high-intensity training and a hypocaloric Mediterranean diet enhance endothelial progenitor cells and fitness in subjects with the metabolic syndrome. Clin Sci (Lond) 2012;123:361-73.

Fernández-Ballart JD, Piñol JL, Zazpe I, et al. Relative validity of a semi-quantitative food-frequency questionnaire in an elderly Mediterranean population of Spain. Br J Nutr 2010;103:1808-16.

Fernández-Montero A, Bes-Rastrollo M, Beunza JJ, et al. Nut consumption and incidence of metabolic syndrome after 6-year follow-up: the SUN (Seguimiento Universidad de Navarra, University of Navarra Follow-up) cohort. Public Health Nutr 2012 Oct 23:1-9.

Finucane MM, Stevens GA, Cowan MJ, et al. National, regional, and global trends in body-mass index since 1980: systematic analysis of health examination surveys and epidemiological studies with 960 country-years and 9.1 million participants. Lancet 2011;377:557-67.

First MB, Spitzer RL, Gibbon M, Williams J, Structured Clinical Interview for DSM-IV-TR Axis I Disorders, Research Version, Non-patient Edition (SCID-I/P). New York: Biometrics Research, New York State Psychiatric Institute. 2002.

Fuster V, et al. "ACC/AHA/ESC 2006 guidelines for the management of patients with atrial fibrillation executive summary: a report of the American College of Cardiology/American Heart Association Task Force on Practice Guidelines and the European Society of Cardiology Committee for Practice Guidelines(Writing Committee to Revise the 2001 Guidelines for the Management of Patients with Atrial Fibrillation)." Eur Heart J 2006; 27:1979-2030.

Fito M, Cladellas M, de la Torre R, et al. Anti-inflammatory effect of virgin olive oil in stable coronary disease patients: a randomized, crossover, controlled trial. Eur J Clin Nutr 2008;62:570-4.

Fito M, Fitó M, Guxens M, et al. Effect of a traditional Mediterranean diet on lipoprotein oxidation: a randomized controlled trial. Arch Intern Med 2007;167:1195-1203.

Flegal KM, Kit BK, Orpana H, Graubard BI. Association of all-cause mortality with overweight and obesity using standard body mass index categories: a systematic review and meta-analysis. JAMA 2013;309:71-82.

Flint AJ, Hu FB, Glynn RJ, et al. Excess weight and the risk of incident coronary heart disease among men and women. Obesity (Silver Spring) 2010;18:377-83.

Folstein MF, Folstein SE, McHugh PR. Mini-mental state: A practical method for grading the cognitive state of patients for the clinician. J Psychiatr Res 1975;12:189-98.

Fuentes F, López-Miranda J, Pérez-Martínez P, et al. Chronic effects of a high-fat diet enriched with virgin olive oil and a low-fat diet enriched with alpha-linolenic acid on postprandial endothelial function in healthy men. Br J Nutr 2008;100:159-65.

GBD 2015 Obesity Collaborators. Health Effects of Overweight and Obesity in 195 Countries over 25 Years. N Engl J Med 2017;377:13-27.

Gea A, Bes-Rastrollo M, Toledo E, Garcia-Lopez M, Beunza JJ, Estruch R, Martinez-Gonzalez MA. Mediterranean alcohol-drinking pattern and mortality in the SUN (Seguimiento Universidad de Navarra) Project: a prospective cohort study. Br J Nutr 2014;111:1871-80.

González-Muniesa P, Martínez-González MA, Hu FB, Després JP, Matsuzawa Y, Loos RJF, Moreno LA, Bray GA, Martinez JA. Obesity. Nat Rev Dis Primers 2017;3:17034

Gregg EW, Chen H, Wagenknecht LE, et al. Association of an intensive lifestyle intervention with remission of type 2 diabetes. JAMA 2012;308:2489-96.

Guasch-Ferré M, Bulló M, Martínez-González MÁ, et al. Frequency of nut consumption and mortality risk in the PREDIMED nutrition intervention trial. BMC Med 2013;11:164.

Gunstad J, Lhotsky A, Wendell CR, Ferrucci L, Zonderman AB. Longitudinal examination of obesity and cognitive function: results from the Baltimore longitudinal study of aging. Neuroepidemiology 2010;34:222-9.

Gutiérrez-Fisac JL, Guallar-Castillón P, León-Muñoz LM, et al. Prevalence of general and abdominal obesity in the adult population of Spain, 2008-2010: the ENRICA study. Obes Rev 2012;13:388-92.

Haaf P, Drexler B, Reichlin T, et al. High Sensitivity Cardiac Troponin in the Distinction of Acute Myocardial Infarction from Acute Cardiac Non-Coronary Artery Disease, Circulation 2012;126:31-40.

Hall KD, Sacks G, Chandramohan D, et al. Quantification of the effect of energy imbalance on bodyweight. Lancet 2011;378:826-37.

Henríquez Sánchez P, Ruano C, de Irala J, Ruiz-Canela M, Martínez-González MA, Sánchez-Villegas A. Adherence to the Mediterranean diet and quality of life in the SUN Project. Eur J Clin Nutr 2012;66:360-8.

Hernán MA, Taubman SL. Does obesity shorten life? The importance of well-defined interventions to answer causal questions. Int J Obes (Lond) 2008;32 Suppl 3:S8-14.

Howard BV, Van Horn L, Hsia J, et al. Low-fat dietary pattern and risk of cardiovascular disease: the Women's Health Initiative Randomized Controlled Dietary Modification Trial. JAMA 2006;295:655-66.

Hu T, Mills KT, Yao L, et al. Effects of low-carbohydrate diets versus low-fat diets on metabolic risk factors: a meta-analysis of randomized controlled clinical trials. Am J Epidemiol 2012;176 Suppl 7:S44-54.

Hamm CW, Bassand JP, et al. ESC Committee for Practice Guidelines. ESC Guidelines for the management of acute coronary syndromes in patients presenting without persistent ST-segment elevation The Task Force for the management of acute coronary syndromes (ACS) in patients presenting without persistent ST-segment elevation of the European Society of Cardiology (ESC). Eur Heart J 2011;32:2999–3054.

Hughes AJ, Daniel SE, Kilford L, Lees AJ. Accuracy of clinical diagnosis of idiopathic Parkinson’s disease: a clinic-pathological study of 100 cases. J Neurol Neurosurg Psych 1992;5:181-184.

Jiang R, Jacobs DR Jr, Mayer-Davis E, et al. Nut and seed consumption and inflammatory markers in the multi-ethnic study of atherosclerosis. Am J Epidemiol 2006;163:222-31.

Jones JL, Fernandez ML, McIntosh MS, et al. A Mediterranean-style low-glycemic-load diet improves variables of metabolic syndrome in women, and addition of a phytochemical-rich medical food enhances benefits on lipoprotein metabolism. J Clin Lipidol 2011;5:188-96.

Kastorini CM, Milionis HJ, Esposito K, Giugliano D, Goudevenos JA, Panagiotakos DB. The effect of Mediterranean diet on metabolic syndrome and its components: a meta-analysis of 50 studies and 534,906 individuals. J Am Coll Cardiol 2011; 57:1299-313.

Khanna D, Fitzgerald JD, et al. 2012 American College of Rheumatology guidelines for management of gout. Part 1: systematic nonpharmacologic and pharmacologic therapeutic approaches to hyperuricemia. Arthritis Care Res (Hoboken) 2012;64:1431-1446.

Larsen TM, Dalskov SM, van Baak M, et al. Diets with high or low protein content and glycemic index for weight-loss maintenance. N Engl J Med 2010;363:2102-13.

Lauby-Secretan B, Scoccianti C, Loomis D, Grosse Y, Bianchini F, Straif K; International Agency for Research on Cancer Handbook Working Group. Body Fatness and Cancer--Viewpoint of the IARC Working Group. N Engl J Med 2016;375:794-8.

Li TY, Brennan AM, Wedick NM, Mantzoros C, Rifai N, Hu FB. Regular consumption of nuts is associated with a lower risk of cardiovascular disease in women with type 2 diabetes. J Nutr 2009;139:1333-8.

Look AHEAD Research Group. Cardiovascular effects of intensive lifestyle intervention in type 2 diabetes. N Engl J Med 2013;369:145-54.

López-Miranda J, Pérez-Jiménez F, Ros E, et al. Olive oil and health: Summary of the II International Conference on Olive Oil and Health consensus report, Jaén and Córdoba (Spain) 2008. Nutr Metab Cardiovasc Dis 2010;20:284-94.

Lozano R, Naghavi M, Foreman K, et al. Global and regional mortality from 235 causes of death for 20 age groups in 1990 and 2010: a systematic analysis for the Global Burden of Disease Study 2010. Lancet 2012;380:2095-128.

Ludwig DS. Weight loss strategies for adolescents: a 14-year-old struggling to lose weight. JAMA 2012;307:498-508.

Luppino FS, de Wit LM, Bouvy PF, et al. Overweight, obesity, and depression: a systematic review and meta-analysis of longitudinal studies. Arch Gen Psychiatry 2010;67:220-9.

Malik VS. Hu FB. Popular weight-loss diets: from evidence to practice. Nat Clin Pract Cardiovasc Med 2007;4:34-41.

Malik VS, Willett WC, Hu FB. Global obesity: trends, risk factors and policy implications. Nat Rev Endocrinol 2013;9:13-27.

Martínez-González MÁ, Bes-Rastrollo M. Nut consumption, weight gain and obesity: Epidemiological evidence. Nutr Metab Cardiovasc Dis 2011;21(Suppl 1): S40-45.

Martinez-Gonzalez MA, Bes-Rastrollo M. Dietary patterns, Mediterranean diet, and cardiovascular disease. Curr Opin Lipidol 2014;25:20-6.

Martínez-González MÁ, Corella D, Salas-Salvadó J, et al. Cohort profile: design and methods of the PREDIMED study. Int J Epidemiol 2012;41:377-85.

Martínez-González MÁ, Fernández-Jarne E, Serrano-Martínez M, Wright M, Gomez-Gracia E. Development of a short dietary intake questionnaire for the quantitative estimation of adherence to a cardioprotective Mediterranean diet. Eur J Clin Nutr 2004;58:1550-2.

Martínez-González MÁ, Hershey MS, Zazpe I, Trichopoulou A. Transferability of the Mediterranean Diet to Non-Mediterranean Countries. What Is and What Is Not the Mediterranean Diet. Nutrients 2017;9:1226.

Masters RK, Powers DA, Link BG. Obesity and US Mortality Risk Over the Adult Life Course. Am J Epidemiol 2013;177:431-42.

McKhann GM, Knopman DS, Chertkow H, et al. The diagnosis of dementia due to Alzheimer's disease: recommendations from the National Institute on Aging-Alzheimer's Association workgroups on diagnostic guidelines for Alzheimer's disease. Alzheimers Dement 2011;7:263-9.

McMurray JJ, Adamopoulos S, Anker SD, Auricchio A, Böhm M, Dickstein K, et Al; ESC Committee for Practice Guidelines. ESC Guidelines for the diagnosis and treatment of acute and chronic heart failure 2012. The Task Force for the Diagnosis and Treatment of Acute and Chronic Heart Failure 2012 of the European Society of Cardiology. Developed in collaboration with the Heart Failure Association (HFA) of the ESC. Eur Heart J 2012;33:1787–1847.

McManus K, Antinoro L, Sacks F. A randomized controlled trial of a moderate-fat, low-energy diet compared with a low fat, low-energy diet for weight loss in overweight adults. Int J Obes Relat Metab Disord 2001;25:1503-11.

Mena MP, Sacanella E, Vazquez-Agell M, et al. Inhibition of circulating immune cell activation: a molecular antiinflammatory effect of the Mediterranean diet. Am J Clin Nutr 2009;89:248-56.

Meneses ME, Camargo A, Perez-Martinez P, et al. Postprandial inflammatory response in adipose tissue of patients with metabolic syndrome after the intake of different dietary models. Mol Nutr Food Res 2011;55:1759-70.

Moher D, Schulz KF, Altman DG. The CONSORT statement: revised recommendations for improving the quality of reports of parallel-group randomised trials. Lancet 2001;357:1191-4.

Moreno JA, López-Miranda J, Pérez-Martínez P, et al. A monounsaturated fatty acid-rich diet reduces macrophage uptake of plasma oxidised low-density lipoprotein in healthy young men. Br J Nutr 2008;100:569-75.

Mozaffarian D, Hao T, Rimm EB, Willett WC, Hu FB. Changes in diet and lifestyle and long-term weight gain in women and men. N Engl J Med 2011;364:2392-404.

National Institute of Diabetes and Digestive and Kidney Diseases. Body weight simulator. See: http://bwsimulator.niddk.nih.gov/, consulted 05/01/2012.

National Institute of Health. ClinicalTrials.gov. A service of the U.S. National Institutes of Health. See: http://clinicaltrials.gov/ct2/home, consulted 05/01/2012.

National Institute of Health. Clinical Guidelines on the Identification, Evaluation, and Treatment of Overweight and Obesity in Adults--The Evidence Report. National Institutes of Health. Obes Res 1998;2(6Suppl):51S-209S.

National Institute of Health, National Heart Lung and Blood Institute and North American Association for the Study of Obesity. The practical guide: Identification, evaluation, and treatment of overweight and obesity in adults. 2000.

Ni Mhurchu C, Rodgers A, Pan WH, Gu DF, Woodward M. Body mass index and cardiovascular disease in the Asia-Pacific Region: an overview of 33 cohorts involving 310 000 participants. Int J Epidemiol 2004;33:751-8.

Nigg CR, Burbank PM, Padula C, et al. Stages of change across ten health risk behaviors for older adults. Gerontologist 1999;39: 473-82.

Nordmann AJ, Nordmann A, Briel M, Keller U, Yancy WS Jr, Brehm BJ, Bucher HC. Effects of low-carbohydrate vs low-fat diets on weight loss and cardiovascular risk factors: a meta-analysis of randomized controlled trials. Arch Intern Med 2006;166:285-93.

Norgren L, Hiatt WR, Dormandy JA, Nehler MR, Harris KA, Fowkes FG. Inter-Society Consensus for the Management of Peripheral Arterial Disease (TASC II). J Vasc Surg 2007;45 Suppl S:S5-67.

O'Brien PC, Fleming TR. A multiple testing procedure for clinical trials. Biometrics 1979;35:549-56.

Pedrero-Perez EJ, Ruiz-Sanchez de Leon JM, Lozoya-Delgado P, Llanero-Luque M, Rojo-Mota G, Puerta-García C. Evaluación de los síntomas prefrontales: propiedades psicométricas y datos normativos del cuestionario disejecutivo (DEX) en una muestra de población española. Rev Neurol 2011; 52: 394-404.

Peña-Casanova J, Quiñones-Ubeda S, Gramunt-Fombuena N, et al. Spanish Multicenter Normative Studies (NEURONORMA Project): norms for verbal fluency tests. Arch Clin Neuropsychol 2009a;24:395-411.

Peña-Casanova J, Quiñones-Ubeda S, Quintana-Aparicio M, et al. Spanish Multicenter Normative Studies (NEURONORMA Project): norms for verbal span, visuospatial span, letter and number sequencing, trail making test, and symbol digit modalities test. Arch Clin Neuropsychol 2009b;24:321-41.

Pérez-Jiménez F, Lista JD, Pérez-Martínez P, et al. Olive oil and haemostasis: a review on its healthy effects. Public Health Nutr 2006;9:1083-8.

Ramier AM, Hécaen H. Role Respectif des atteintes frontales et de la latéralisation lésionelle dans les deficits de la fluence verbal. Rev Neurol (Paris) 1970:123:17-22.

Ramier AM, Hécaen H. Les déficits au test de "fluence verbale" chez les subjets guachers avec lesions hemispheriques unilaterales. Rev Neurol (Paris) 1977:133: 571–4.

Razquin C, Martinez JA, Martinez-Gonzalez MA, Mitjavila MT, Estruch R, Marti A. A 3 years follow-up of a Mediterranean diet rich in virgin olive oil is associated with high plasma antioxidant capacity and reduced body weight gain. Eur J Clin Nutr 2009;63:1387-93.

Razquin C, Martínez JA, Martínez-González MA, Salas-Salvadó J, Estruch R, Marti A. A 3-year Mediterranean-style dietary intervention may modulate the association between adiponectin gene variants and body weight change. Eur J Nutr 2010;49:311-9.

Reitan RM. Trail Making Test: Manual for administration and scoring. Tucson: Reitan Neuropsychology Laboratory; 1992.

Rejeski WJ, Ip EH, Bertoni AG, et al. Lifestyle Change and Mobility in Obese Adults with Type 2 Diabetes. N Engl J Med 2012;366:1209-17.

Romaguera D, Norat T, Vergnaud AC, et al. Mediterranean dietary patterns and prospective weight change in participants of the EPIC-PANACEA project. Am J Clin Nutr 2010;92:912-21.

Ros E. Nuts and novel biomarkers of cardiovascular disease. Am J Clin Nutr 2009; 89:1649S-56S.

Ruano J, Lopez-Miranda J, Fuentes F, et al. Phenolic content of virgin olive oil improves ischemic reactive hyperemia in hypercholesterolemic patients. J Am Coll Cardiol 2005; 46:1864-8.

Ruiz-Canela M, Estruch R, Corella D, Salas-Salvadó J, Martínez-González MA. Association of Mediterranean diet with peripheral artery disease: the PREDIMED randomized trial. JAMA 2014;311:415-7.

Ryan DH, Espeland MA, Foster GD, et al. Look AHEAD (Action for Health in Diabetes): design and methods for a clinical trial of weight loss for the prevention of cardiovascular disease in type 2 diabetes. Control Clin Trials 2003;24:610-28.

Sabaté J, Oda K, Ros E. Nut consumption and blood lipid levels: a pooled analysis of 25 intervention trials. Arch Intern Med 2010;170:821-7.

Sacco RL, Kasner SE, Broderick JP, et al. An Updated Definition of Stroke for the 21st Century: A Statement for Healthcare Professionals from the American Heart Association/American Stroke Association. Stroke 2013;44:2064-2089.

Sacks FM, Bray GA, Carey VJ, et al. Comparison of weight-loss diets with different compositions of fat, protein, and carbohydrates. N Engl J Med 2009;360:859-73

Salas-Salvadó J, Fernández-Ballart J, Ros E, et al. Effect of a Mediterranean diet supplemented with nuts on metabolic syndrome status: one-year results of the PREDIMED randomized trial. Arch Intern Med 2008;168:2449-58.

Salas-Salvadó J, Bulló M, Babio N, et al. Reduction in the incidence of type 2 diabetes with the Mediterranean diet: results of the PREDIMED-Reus nutrition intervention randomized trial. Diabetes Care 2011;34:14-9.

Salas-Salvadó J, Bulló M, Estruch R, et al. Prevention of diabetes with Mediterranean diets: a subgroup analysis of a randomized trial. Ann Intern Med 2014;160:1-10.

Sanchez-Villegas A, Ara I, Dierssen T, de la Fuente C, Ruano C, Martínez-González MA. Physical activity during leisure time and quality of life in a Spanish cohort: SUN (Seguimiento Universidad de Navarra) project. Br J Sports Med 2012;46:443-8.

Sanz, Navarro y Vázquez, Adaptación española para el Inventario de Depresión de Beck-II (BDI-II). 1. Propiedades psicométricas en estudiantes universitarios. Análisis y modificación de conducta 2003;29: 239-288.

Schröder H, Fitó M, Estruch R, et al. A short screener is valid for assessing Mediterranean diet adherence among older Spanish men and women. J Nutr 2011;141:1140-5.

Schulz KF, Grimes DA. Multiplicity in randomised trials II: subgroup and interim analyses. Lancet 2005;365:1657-61.

Schulze MB, Fung TT, Manson JE, Willett WC, Hu FB. Dietary patterns and changes in body weight in women. Obesity (Silver Spring) 2006;14:1444-53.

Shai I, Schwarzfuchs D, Henkin Y, et al. Weight loss with a low-carbohydrate, Mediterranean, or low-fat diet. N Engl J Med 2008;359:229-41.

Smith, A. Symbol Digit Modalities Test. Manual. Los Angeles: Western Psychological Services; 1973.

Sofi F, Abbate R, Gensini GF, Casini A. Accruing evidence on benefits of adherence to the Mediterranean diet on health: an updated systematic review and meta-analysis. Am J Clin Nutr 2010;92:1189-96.

Sofi F, Macchi C, Abbate R, Gensini GF, Casini A. Mediterranean diet and health status: an updated meta-analysis and a proposal for a literature-based adherence score. Public Health Nutr 2014;17:2769-82.

Song YM, Sung J, Davey Smith G, Ebrahim S. Body mass index and ischemic and hemorrhagic stroke: a prospective study in Korean men. Stroke 2004;35:831-6.

Tendera M, Aboyans V, Bartelink ML, et al. ESC Guidelines on the diagnosis and treatment of peripheral artery diseases. Eur Heart J 2011;32: 2851–2906.

Tobias DK, Hu FB. Does being overweight really reduce mortality? Obesity (Silver Spring) 2013;21:1746-9.

Thygesen K, Alpert JS, Jaffe L, et al. Third universal definition of myocardial infarction. Eur Heart J 2012;33:2551–2567.

Urpi-Sarda M, Casas R, Chiva-Blanch G, et al. Virgin olive oil and nuts as key foods of the Mediterranean diet effects on inflammatory biomakers related to atherosclerosis. Pharmacol Res 2012; 65:577-83.

Wallston KA, Stein MJ, Smith CA. Form C of the MHLC scales: a condition-specific measure of locus of control. J Pers Assess 1994;63:534-53.

Wallston, Wallston, & DeVellis, 1978, Health Education Monographs, 6, 160-170.

Ware JE, Gandek B, Gandek BJr. Overview of the SF-36 Health Survey and the International Quality of Life Assessment (IQOLA) Project. J Clin Epidemiol 1998;51:903-12.

Wechsler, D. Wechsler Adult Intelligence Scale-III. San Antonio, Texas: Psychological Corporation; 1997a.

Wechsler, D. Wechsler Memory Scale. San Antonio, Texas: Psychological Corporation; 1997b.
Wechsler, D. WAIS III. Escala de inteligencia de Wechsler para adultos-III. Manual técnico. Madrid: TEA Ediciones; 1999.

Wechlsler D. Escala de memoria Wechsler.-III. Manual técnico: TEA Ediciones. S. A; 2004.

Willett WC. Dietary fat plays a major role in obesity: no. Obes 2002;Rev 3:59-68.

Willett WC. Eat, drink, and be healthy: the Harvard Medical School guide to healthy eating. New York: Free Press, 2001

Wilson BA, Alderman N, Burgess PW, Emslie H, Evans JJ. Behavioural assessment of the dysexecutive syndrome. Bury St. Edmunds, UK: Thames Valley Test; 1996.

Wing RR. Long-term effects of a lifestyle intervention on weight and cardiovascular risk factors in individuals with type 2 diabetes mellitus: four-year results of the Look AHEAD trial. Arch Intern Med 2010;170:1566-75.

World Health Organization. Obesity: Preventing and managing the global epidemic. Geneva: World Health Organization, 1998.World Health Organization. International Classification of Diseases for Oncology, 3rd Edition (ICD-O-3).
http://www.who.int/classifications/icd/adaptations/oncology/en/index.html, consulted 24/04/2012.

Yesavage JA. Geriatric Depression Scale. Psychopharmacol Bull 1988;24:709-11.

Yancy CW, Jessup M, Bozkurt B, et al. 2013 ACCF/AHA guideline for the management of heart failure: a report of the American College of Cardiology Foundation/American Heart Association Task Force on Practice Guidelines. J Am Coll Cardiol 2013; 62: e147- e239.

Zazpe I, Sanchez-Tainta A, Estruch R, et al. A large randomized individual and group intervention conducted by registered dietitians increased adherence to Mediterranean-type diets: the PREDIMED study. J Am Diet Assoc 2008;108:1134-44

Zhu L, Liu Z, Feng Z, et al. Hydroxytyrosol protects against oxidative damage by simultaneous activation of mitochondrial biogenesis and phase II detoxifying enzyme systems in retinal pigment epithelial cells. J Nutr Biochem 2010;21:1089-98.

ANNEX 1. SUB-STUDIES

1. Body composition

DEXA or computed tomography is used to measure body composition at recruiting centers wherein the necessary equipment and technology are available. Body composition is analyzed by General Electric Lunar DEXA scanner at the Rovira i Virgili University, Universitat de les Illes Balears, Hospital Clinico de Barcelona, and the Departments of Preventive Medicine and Nutrition in Navarra.

2. Other sub-studies

Depending on available funds, sub-studies will be conducted to evaluate gene environment interactions, epigenetic factors such as DNA methylation, histone modification and microRNA alterations, the composition and function of intestinal microbiota by pyrosequencing, and the effect of the intervention on metabolomics, transcriptomics and proteomics.

ANNEX 2. PARTICIPATING CENTERS

Recruitment centers

ID	PI	Email address	Center
01	Julia Wärnberg	jwarnberg@uma.es	Escuela de Ciencias de la Salud, Universidad de Málaga-IBIMA, Málaga
02	José Lapetra	jlapetra@ono.com	Distrito Sanitario de Atención Primaria de Sevilla, Sevilla
03	J. Alfredo Martínez	jalfmtz@unav.es	Universidad de Navarra - Nutrition, Pamplona
04	María Adoración Romaguera; Miquel Fiol	mariaadoracion.romaguera@ ssib.es; miquelfiol@yahoo.es	Hospital Son Espases, Palma
05	Ramon Estruch	restruch@clinic.ub.es	Hospital Clínic-Medicina Interna, Barcelona
06	Montserrat Fitó	mfito@imim.es	Institut Hospital del Mar d'Investigacions Mèdiques, Barcelona
07	Jordi Salas-Salvadó	jordi.salas@urv.cat	Universitat Rovira i Virgili, Reus
08	Aurora Bueno	abueno@ugr.es	Universidad de Granada, Granada
09	Clotilde Vázquez	cvazquezma@gmail.com	Hospital Ramón y Cajal, Madrid
10	Miguel Ángel Martínez-González	mamartinez@unav.es	Universidad de Navarra - Epidemiology, Pamplona
11	Fernando Aròs	aborau@htxa.osakidetza.net	Hospital Universitario Araba, Vitoria
12	Dolores Corella	dolores.corella@uv.es	Universidad de Valencia, Valencia
13	Lluis Serra-Majem	lserra@dcc.ulpgc.es	Universidad de las Palmas de Gran Canaria, Las Palmas
14	Xavier Pintó	xpinto@csub.scs.es	Hospital de Bellvitge, L'Hospitalet de Llobregat, Barcelona
15	José López Miranda	jlopezmir@gmail.com	Universidad de Córdoba, Córdoba
16	José Maria Ordovás	Jose.Ordovas@tufts.edu	IMDEA, Madrid
17	Pilar Matía Martín	pilar.matia@gmail.com	Hospital Clínico, Madrid
18	Francisco Tinahones	fjtinahones@hotmail.com	Hospital de Málaga, Málaga
19	Josep Tur	pep.tur@uib.es	Universitat de les Illes Balears, Palma
20	Josep Vidal	JOVIDAL@clinic.ub.es	Hospital Clínic – Endocrinología, Barcelona
21	Jesús Vioque López	vioque@umh.es	Universidad Miguel Hernández, Sant Joan d'Alacant, Alicante
22	Miguel Delgado Rodríguez	mdelgado@ujaen.es	Universidad de Jaén, Jaén
23	Vicente Martin	vicente.martin@unileon.es	Universidad de León, León

Support centers

ID	PI	Email address	Center
**A1**	Emilio Ros	eros@clinic.ub.es	Hospital Clínic, Barcelona
**A2**	Rosa M. Lamuela-Raventós	lamuela@ub.edu	Universitat de Barcelona
**A3**	Guillermo Sáez	guillermo.Saez@uv.es	Universitat de Valencia
**A4**	María del Puy Portillo	mariapuy.portillo@ehu.es	Universidad del País Vasco
**A5**	Ascensión Marcos	amarcos@ictan.csic.es	CSIC, Madrid
**A6**	Cristina Botella	botella@uji.es	Universitat Jaume I de Castellón
**A7**	Fernando Fernández-Aranda	ffernandez@bellvitgehospital.cat	Hospital Universitari de Bellvitge
**A8**	Enrique Gómez-Gracia	egomezgracia@gmail.com	Medicina Preventiva, Facultad de Medicina, Universidad de Málaga, Málaga
