# Supplementary material for: An Energy-Reduced Mediterranean Diet, Physical Activity, and Body Composition: An Interim Subgroup Analysis of the PREDIMED-Plus Randomized Clinical Trial
Source: JAMA Netw Open. 2023 Oct 18;6(10):e2337994. doi: 10.1001/jamanetworkopen.2023.37994 (PMC10585413; doi:10.1001/jamanetworkopen.2023.37994)
Supplement: Supplement 2. — eTable 1. Number of Study Participants With and Without Data on Body Composition From Centers Having Access to DXA Device eTable 2. Comparison of Principal Baseline Characteristics of Study Participants With Data on Body Composition Measured With DXA and Participants Without DXA Data From the Total Cohort eFigure 1. Flow Chart for the Selection of Participants for Analysis eMethods. Details on PREDIMED-Plus Intervention and Body Composition Measurement With DXA eReferences eFigure 2. Adjusted Means (95% CIs) of Body Composition Parameters (Primary Outcomes) Through Follow-Up by Study Arm Separately in Men and Women (Completers-Only) eFigure 3. Adjusted Means (95% CIs) of Body Composition Parameters (Secondary Outcomes) Through Follow-Up by Study Arm in Overall Sample and Separately in Men and Women eTable 3. Effect of the PREDIMED-Plus Intervention on Body Composition Changes (z-Scores of Primary and Secondary Outcomes) Over Follow-Up Time (Completers-Only) eTable 4. Effect of the PREDIMED-Plus Intervention on Body Composition Changes (Primary and Secondary Outcomes) Over Follow-Up Time (Multiple Imputations, n = 100) eFigure 4. Effect of the PREDIMED-Plus Intervention on Body Composition Changes Over Follow-Up Time by Subgroups (Completers-Only) [file jamanetwopen-e2337994-s002.pdf]

Supplementary Online Content

Konieczna J, Ruiz-Canela M, Galmes-Panades AM, et al. An energy-reduced Mediterranean diet, physical activity, and body composition: an interim subgroup analysis of the PREDIMED-Plus randomized clinical trial. *JAMA Netw Open*. 2023;6(10):e2337994. doi:10.1001/jamanetworkopen.2023.37994

eTable 1. Number of Study Participants With and Without Data on Body Composition From Centers Having Access to DXA Device

eTable 2. Comparison of Principal Baseline Characteristics of Study Participants With Data on Body Composition Measured With DXA and Participants Without DXA Data From the Total Cohort

eFigure 1. Flow Chart for the Selection of Participants for Analysis

eMethods. Details on PREDIMED-Plus Intervention and Body Composition Measurement With DXA

eReferences

eFigure 2. Adjusted Means (95% CIs) of Body Composition Parameters (Primary Outcomes) Through Follow-Up by Study Arm Separately in Men and Women (Completers-Only)

eFigure 3. Adjusted means (95% CIs) of Body Composition Parameters (Secondary Outcomes) Through Follow-Up by Study Arm in Overall Sample and Separately in Men and Women

eTable 3. Effect of the PREDIMED-Plus intervention on Body Composition Changes (z-Scores of Primary and Secondary Outcomes) Over Follow-Up Time (Completers-Only)

eTable 4. Effect of the PREDIMED-Plus Intervention on Body Composition Changes (Primary and Secondary Outcomes) Over Follow-Up Time (Multiple Imputations, n=100)

eFigure 4. Effect of the PREDIMED-Plus Intervention on Body Composition Changes Over Follow-Up Time by Subgroups (Completers-Only)

This supplementary material has been provided by the authors to give readers additional information about their work.

© 2023 Konieczna J et al. *JAMA Network Open.*

eTable 1. Number of study participants with and without data on body composition from centers having access to DXA device.

Recruting center	Total	Included[a]	Non-included[b]
	n	n (%)	n (%)
Total from all centers with available DXA	2519	1556 (61.8)	963 (38.2)
Center 3	331	271 (81.9)	60 (18.1)
Center 4	335	303 (90.4)	32 (9.6)
Center 5	302	152 (50.3)	150 (49.7)
Center 7	460	357 (77.6)	103 (22.4)
Center 10[c]	628	98 (15.6)	530 (84.4)
Center 20	205	135 (65.9)	70 (51.9)
Center 23	258	240 (93.0)	18 (7.0)

Data are shown as numbers (percentages).
(a) Participants with data on body composition measured with DXA and included in the analysis.
(b) Participants without DXA data excluded from the analysis.
(c) Center #10 was the vanguard center and started the recruitment and randomization many months earlier than the other centers, therefore many participants of center #10 had had their baseline evaluation before the DXA study started.

The sample of 1556 participants (n=1521 after exclusions of participants with missing data on visceral fat and covariables) was coming from seven out of the 23 recruitment centers as those seven centers were the only centers that had DXA scanner available. In each of these seven centers, either all participants or a sub-sample were invited to DXA scans.

© 2023 Konieczna J et al. *JAMA Network Open.*

eTable 2. Comparison of principal baseline characteristics of study participants with data on body composition measured with DXA and participants without DXA data from the total cohort.

Baseline characteristic	Total cohort	Included[(a)]	Non-included[(b)]	Relative percent difference
	No. (%)	No. (%)	No. (%)	% (95% CI)
n	6874	1556	5318	
Women	3335 (48.5)	746 (47.9)	2589 (48.7)	-0.74 (-3.56; 2.08)
Age, mean (SD), years	64.9 (4.9)	65.3 (5.0)	64.9 (4.9)	0.63 (0.20; 1.05)
BMI, mean (SD), kg/m^2	32.6 (3.5)	32.5 (3.4)	32.6 (3.5)	-0.09 (-0.69; 0.51)
Height, mean (SD), cm	163 (9.3)	163 (9.3)	163 (9.3)	-0.12 (-0.44; 0.21)
Weight, mean (SD), kg	86.6 (13.0)	86.4 (12.8)	86.7 (13.1)	-0.34 (-1.18; 0.51)
Waist circumference, mean (SD), cm	108 (9.7)	107 (9.3)	108 (9.8)	-0.41(-0.92;0.10)
Type 2 diabetes	2093 (30.5)	416 (26.7)	1677 (31.5)	-4.80 (-7.33; -2.27)
Current smokers	3840 (55.9)	896 (57.6)	2944 (55.4)	2.22 (-0.57; 5.02)
Higher education	1526 (22.2)	329 (21.1)	1197 (22.5)	-1.36 (-3.68; 0.95)
Physical activity, mean (SD), METs min/week	2463 (2301)	2686 (2316)	2397 (2293)	12.0 (6.62;17.5)
Chair-stand test, mean (SD), number of repeats[#]	13.8 (4.4)	14.7 (4.8)	13.5 (4.2)	8.65 (6.79;10.5)
Sedentary behaviour, mean (SD), h/day[§]	6.02 (2.0)	5.86 (1.9)	6.06 (2.0)	-3.26 (-5.09; -1.43)
Total energy intake, mean (SD), kcal/day*	2416 (633)	2428 (581)	2413 (647)	0.65 (-0.84; 2.13)
Alcohol intake, mean (SD), g/day*	11.2 (15.3)	11.7 (15.3)	11.1 (15.3)	5.85 (-2.0; 13.7)
Adherence to energy-reduced MedDiet, mean (SD), points	8.50 (2.7)	8.37 (2.6)	8.53 (2.7)	-1.91 (-3.68; -0.14)

Abbreviations: BMI – body mass index; MedDiet – Mediterranean Diet; METs – metabolic equivalent

(a) Participants with data on body composition measured with DXA and included in the analysis
(b) Participants without DXA data excluded from the analysis

Values shown are mean (SD) unless otherwise specified. Relative percent difference was calculated as the difference relative to non-included participants = ((a-b)/(a+b)/2))*100.
[#]Data are unavailable for 276 participants
*Data are unavailable for 36 participants

© 2023 Konieczna J et al. *JAMA Network Open.*

[§]Data are unavailable for 57 participants

For the current analysis educational level (higher education and technician vs secondary education, primary education, less or insufficient data) and smoking habits (current and ex-smoker vs never smoker or insufficient data) were recorded in two categories. Current type 2 diabetes was defined as previous diagnosis of diabetes, glycated hemoglobin ≥ 6.5%, use of antidiabetic medication, or having fasting glucose > 126 mg/dL in both the screening and baseline visit.

© 2023 Konieczna J et al. *JAMA Network Open.*

eFigure 1. Flow chart for the selection of participants for analysis. Abbreviations: DXA - dual-energy X-ray absorptiometry; FFQ – Food Frequency Questionnaire; MedDiet – Mediterranean Diet; PA – physical activity**.**

© 2023 Konieczna J et al. *JAMA Network Open.*

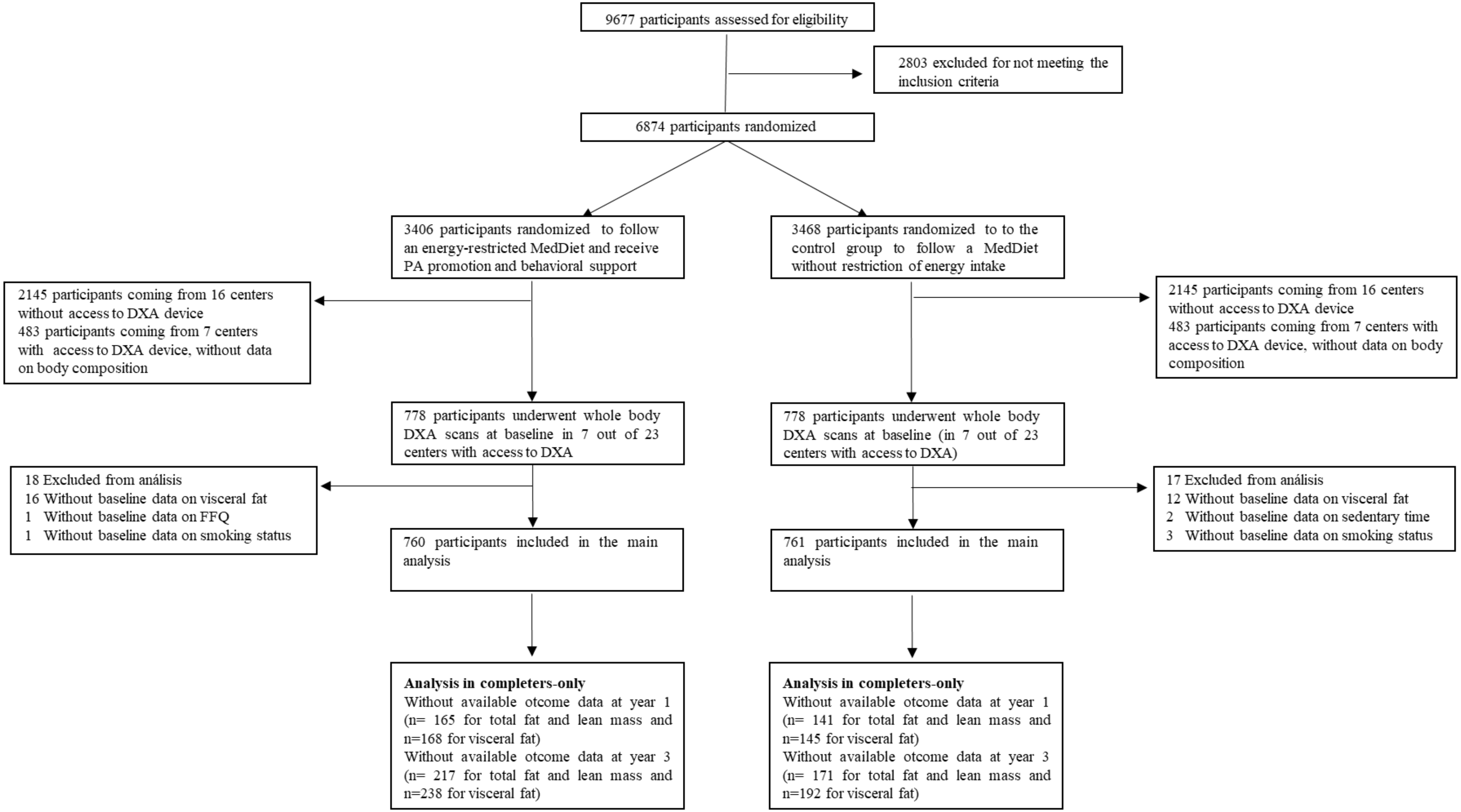

© 2023 Konieczna J et al. *JAMA Network Open.*

eMethods. Details on PREDIMED-Plus intervention and body composition measurement with DXA.

Details on PREDIMED-Plus intervention

The lifestyle intervention introduced to participants from the active arm of the trial was an intensive education program based on energy-reduced Mediterranean Diet (MedDiet) and physical activity (PA) counselling, coupled with behavioural support. Energy reduction in the active intervention arm was projected to be approximately 30% of estimated energy requirements (considering basal metabolic rate and level of PA of each participant), which is equivalent to a reduction of approximately 600 kcal/day. Notwithstanding, the real energy reduction was expected to be modest, as the study was conducted in free-living conditions and physical activity was promoted. Moreover, besides of the foods common for the traditional MedDiet, as implemented in the previous PREDIMED-1 trial[1,2], participants in the intervention group were specifically encouraged to limit consumption of red and processed meats, butter, margarine, cream, sweetened beverages, added sugar, biscuits, as well as white bread and other refined cereals while promoting wholegrains, according to a validated 17-point screener that measured adherence to energy-reduced Mediterranean Diet (MedDiet)[3]. Both groups of participants were supplied for free with extra-virgin olive oil (1 L/month) and all participants were encouraged to consume 500 g/month of mixed nuts.

Participants were also encouraged to progressively increase aerobic PA to ≥150 min/week of moderate-to-vigorous PA, with the final goal of walking ≥45 min/day or equivalent over six days/week, and doing specific exercises to improve strength (sessions of 30-40 min for ≥2 days/week). Participants were also encouraged to practice flexibility and balance exercises at the end of the PA sessions ≥3 days/week. Participants received a PA diary and pedometer a as self-monitoring and motivational tools. Compliance with PA recommendations was evaluated using questionnaires validated for the Spanish population, that were periodically administered to the participants: the REGICOR Short Physical Questionnaire that collect data on level of leisure-time PA[4], and the Nurses' Health Study questionnaire[5] that record data on sedentary behavior. Data collected were used to calculate total PA (metabolic equivalent of tasks (METs) min/week) and time spend on total sedentary behavior (h/day), as previously described[6]. Physical fitness was also assessed using previously validated chair-stand test[7], and a subsample of participants was provided with accelerometers.

One of the key elements that distinguish both groups of participants is the intensity of recommendations received. Participants in the intervention group received lifestyle recommendations by means of a tailored face-to-face educational program, delivered by trained dietitians. The frequency of contacts with this group of participants was three times a month (one group session, one phone call and one individual motivational interview) during the first year of the trial, and twice a month thereafter (one group session and alternate phone calls or personal interviews). In addition, participants receive behavioural and motivational support strategies including self-monitoring, goal setting and problem solving. In each individual motivational interview, participants provided feedback on progress toward personal goals associated with diet and PA recommendations, and could discuss barriers in accomplishing goals with the dietitians. Participants from control group were given advices to follow *ad libitum* traditional

© 2023 Konieczna J et al. *JAMA Network Open.*

MedDiet recommendations (as in the PREDIMED trial[1,2]), without PA promotion, during group sessions twice a year.

Details on body composition measurement with DXA

The regions of interest (ROI) for regional body composition measurement were defined using the software provided by the manufacturer. Among them, the abdominal android ROI, representing supra-umbilical abdomen, was defined by the pelvis line (lower boundary), the trunk lines (lateral boundaries) and a horizontal line identified measuring the 20% of the distance between the pelvis line and head line (upper boundary)[8]. The gynoid ROI, representing gluteo-femoral region, was defined by a horizontal line that is placed caudally 1.5 times the height of the android ROI (upper boundary), the hip lines (lateral boundaries) and another horizontal line identified measuring twice the height of the android ROI (lower boundary)[8]. For visceral fat measurements, scans were re-analyzed using validated CoreScan software application[9], which algorithms work through detection of the width of the subcutaneous fat layer on the lateral part of the abdomen and the anterior-posterior thickness of the abdomen, by x-ray attenuation of the abdominal cavity in the android region. This is automated procedure developed by GE Healthcare[9].

eReferences

1. Schröder H, Fitó M, Estruch R, et al. A Short Screener Is Valid for Assessing Mediterranean Diet Adherence among Older Spanish Men and Women. *J Nutr*. 2011;141(6):1140-1145. doi:10.3945/jn.110.135566

2. Martínez-González MA, García-Arellano A, Toledo E, et al. A 14-item mediterranean diet assessment tool and obesity indexes among high-risk subjects: The PREDIMED trial. *PLoS One*. 2012;7(8). doi:10.1371/journal.pone.0043134

3. Schröder H, Zomeño MD, Martínez-González MA, et al. Validity of the energy-restricted Mediterranean Diet Adherence Screener. *Clin Nutr*. 2021;40(8):4971-4979. doi:10.1016/j.clnu.2021.06.030

4. Molina L, Sarmiento M, Peñafiel J, et al. Validation of the Regicor Short Physical Activity Questionnaire for the Adult Population. *PLoS One*. 2017;12(1):e0168148. doi:10.1371/journal.pone.0168148

5. Martínez-González MA, López-Fontana C, Varo JJ, Sánchez-Villegas A, Martinez JA. Validation of the Spanish version of the physical activity questionnaire used in the Nurses' Health Study and the Health Professionals' Follow-up Study. *Public Health Nutr*. 2005;8(7):920-927. doi:10.1079/PHN2005745

6. Galmes-Panades AM, Konieczna J, Abete I, et al. Lifestyle factors and visceral adipose tissue: Results from the PREDIMED-PLUS study. *PLoS One*. 2019;14(1):e0210726. doi:10.1371/journal.pone.0210726

7. Jones CJ, Rikli RE, Beam WC. A 30-s Chair-Stand Test as a Measure of Lower Body Strength in Community-Residing Older Adults. *Res Q Exerc Sport*. 2013;70(2):113-119. doi:10.1080/02701367.1999.10608028

8. Bazzocchi A, Ponti F, Albisinni U, Battista G, Guglielmi G. DXA: Technical aspects and application. *Eur J Radiol*. 2016;85(8):1481-1492. doi:10.1016/j.ejrad.2016.04.004

9. Kaul S, Rothney MP, Peters DM, et al. Dual-Energy X-Ray Absorptiometry for Quantification of Visceral Fat. *Obesity*. 2012;20(6):1313-1318. doi:10.1038/oby.2011.393

© 2023 Konieczna J et al. *JAMA Network Open.*

eFigure 2. Adjusted Means (95% CIs) of Body Composition Parameters (Primary Outcomes) Through Follow-Up by Study Arm Separately in Men and Women (Completers-Only).

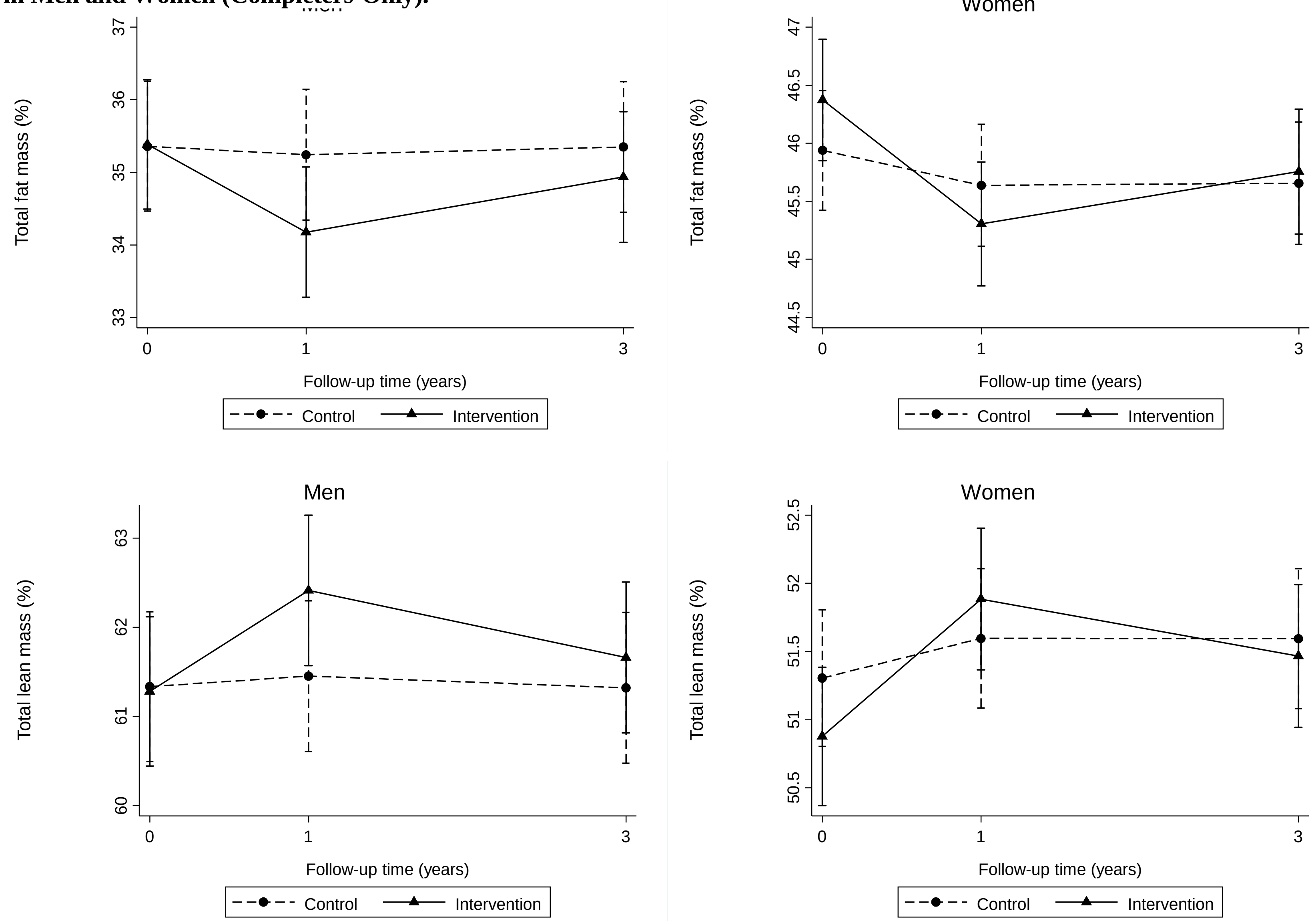

© 2023 Konieczna J et al. *JAMA Network Open.*

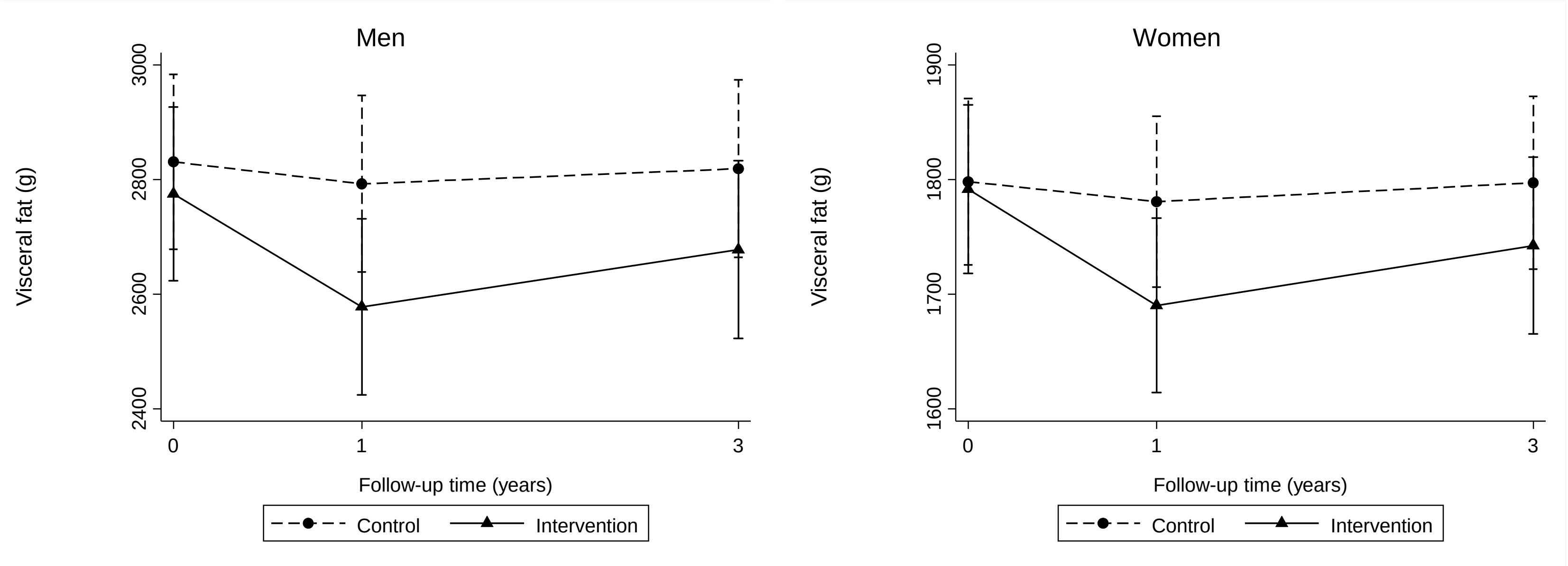

Adjusted means (95% CIs) of body composition parameters (primary outcomes) through follow-up by study arm separately in men and women (completers-only).

Percentages of fat mass and lean mass were calculated in relation to DXA-derived total body mass (sum of total bone, fat and muscle mass).

Means were minimally adjusted for centre, cluster family and individual subject.

© 2023 Konieczna J et al. *JAMA Network Open.*

eFigure 3. Adjusted Means (95% CIs) of Body Composition Parameters (Secondary Outcomes) Through Follow-Up by Study Arm in Overall Sample and Separately in Men and Women

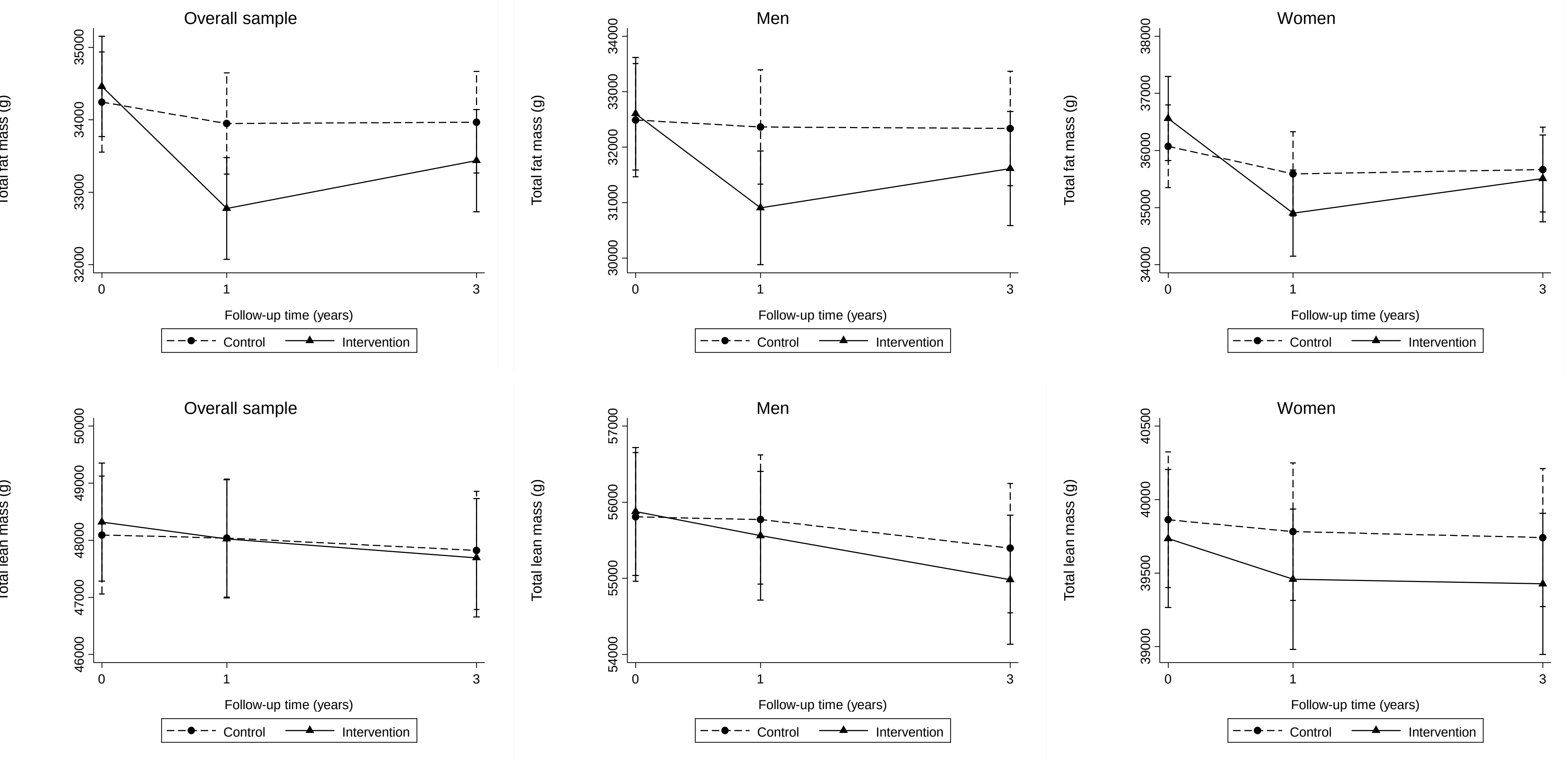

© 2023 Konieczna J et al. *JAMA Network Open.*

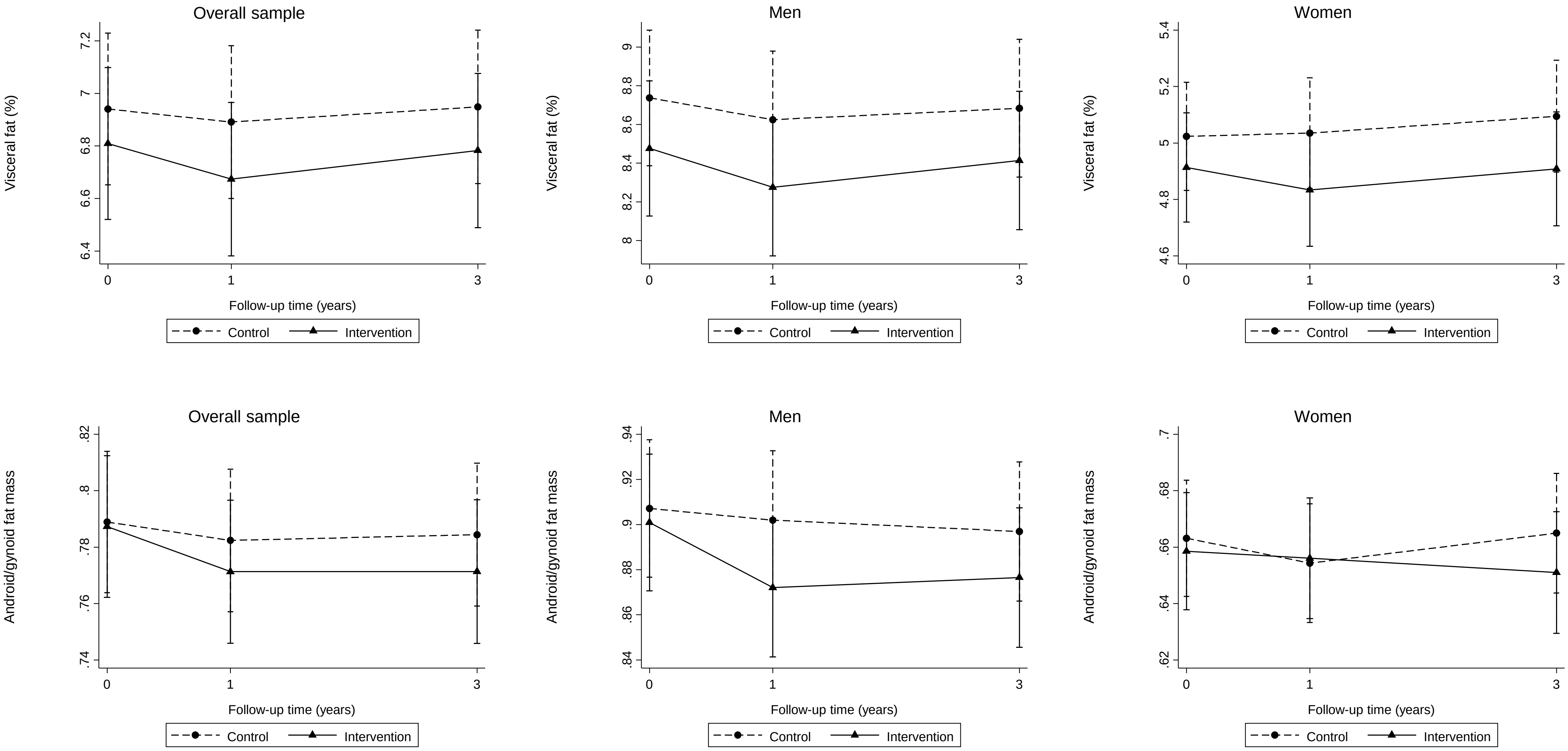

© 2023 Konieczna J et al. *JAMA Network Open.*

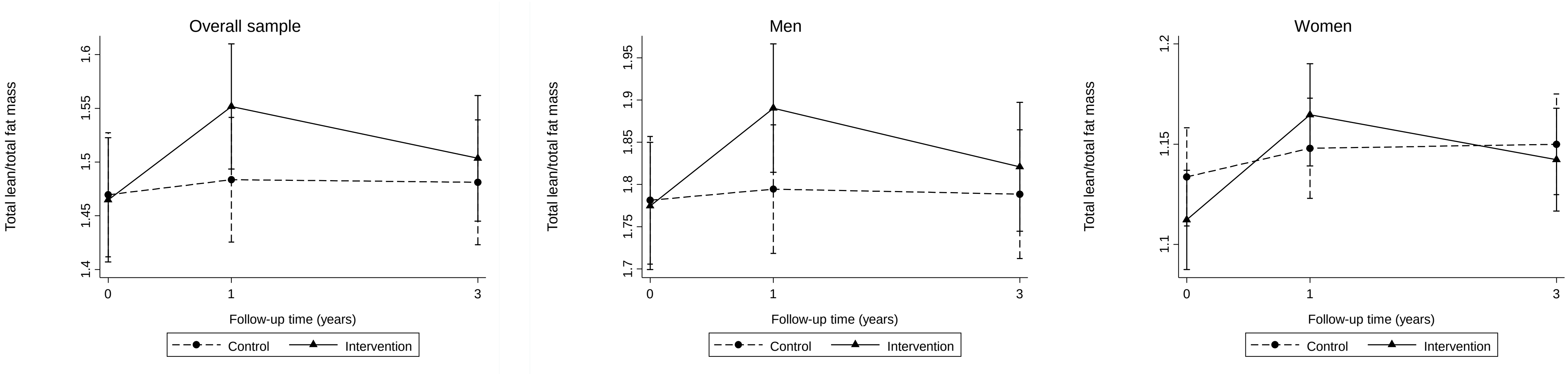

Adjusted means (95% CIs) of body composition parameters (secondary outcomes) through follow-up by study arm in overall sample and separately in men and women.

Percentage of visceral fat was calculated in relation to total fat mass.

Means were minimally adjusted for centre, cluster family and individual subject.

© 2023 Konieczna J et al. *JAMA Network Open.*

eTable 3. Effect of the PREDIMED-Plus intervention on body composition changes (z-scores of primary and secondary outcomes) over follow-up time (completers-only).

	Intervention	Control	Between-group difference	
	**Mean (95% CI)**	**Mean (95% CI)**	**Mean (95% CI)**	**p-value***
**Z-scores**				
**PRIMARY OUTCOMES**				
**Total fat mass (%)**				
Year 1 vs baseline	-0.05 (-0.07; -0.02)	0.09 (0.06; 0.11)	-0.13 (-0.17; -0.10)	<0.001
Year 3 vs baseline	-0.01 (-0.04;0.02)	0.04 (0.02; 0.07)	-0.05 (-0.09; -0.02)	
**Total lean mass (%)**				
Year 1 vs baseline	0.05 (0.02; 0.07)	-0.09 (-0.11; -0.06)	0.13 (0.09; 0.17)	<0.001
Year 3 vs baseline	0.01 (-0.02; 0.04)	-0.04 (-0.07; -0.02)	0.05 (0.01; 0.09)	
**Visceral fat (g)**				
Year 1 vs baseline	-0.08 (-0.12; -0.04)	0.07 (0.02; 0.11)	-0.14 (-0.20; -0.08)	<0.001
Year 3 vs baseline	-0.04 (-0.08; 0.01)	0.04 (-0.01; 0.08)	-0.08 (-0.14; -0.01)	
**SECONDARY OUTCOMES**				
**Total fat mass (g)**				
Year 1 vs baseline	-0.07 (-0.11; -0.04)	0.11 (0.08; 0.15)	-0.19 (-0.23; -0.14)	<0.001
Year 3 vs baseline	-0.04 (-0.08; -0.01)	0.06 (0.02; 0.09)	-0.10 (-0.15; -0.05)	
**Total lean mass (g)**				
Year 1 vs baseline	-0.02 (-0.03; -0.00)	0.01 (-0.01; 0.02)	-0.03 (-0.05; -0.01)	0.001
Year 3 vs baseline	-0.03 (-0.05; -0.02)	0.00 (-0.01; 0.02)	-0.04 (-0.06; -0.02)	
**Visceral fat (%)**				
Year 1 vs baseline	-0.04 (-0.08; -0.01)	0.00 (-0.04; 0.03)	-0.04 (-0.09; 0.01)	0.344
Year 3 vs baseline	-0.02 (-0.06; 0.02)	0.00 (-0.04; 0.04)	-0.02 (-0.07; 0.04)	
**Android/gynoid fat mass**				
Year 1 vs baseline	-0.02 (-0.06; 0.02)	0.03 (-0.01; 0.07)	-0.05 (-0.11; 0.01)	0.108
Year 3 vs baseline	-0.04 (-0.08; 0.00)	0.01 (-0.03; 0.06)	-0.06 (-0.12; 0.00)	
**Total lean mass/total fat mass**				
Year 1 vs baseline	0.06 (0.03; 0.09)	-0.09 (-0.12; -0.06)	0.15 (0.11; 0.19)	<0.001
Year 3 vs baseline	0.01 (-0.02; 0.05)	-0.04 (-0.08; -0.01)	0.06 (0.01; 0.10)	

Percentages of fat mass and lean mass were calculated in relation to DXA-derived total body mass (sum of total bone, fat and muscle mass), whereas percentage of visceral fat was calculated in relation to total fat mass. All outcomes were normalized into z-scores (mean = 0, SD = 1) for comparability between them.

Three-level linear mixed models with random intercepts at recruiting centre, cluster family and individual subject were usedto assess intervention group effects on changes in body composition parameters measured repeatedly over time (at each follow-up visit and for the overall follow-up period). Potential interactions of study arm with time were tested in these models after adjusting for sex (dichotomous), age (continuous), and baseline levels of smoking status (three categories), educational level (three categories), type 2 diabetes prevalence (dichotomous), height, total leisure-time physical activity, sedentary time, total energy intake and alcohol intake (continuous).

*P-value represents the intervention group effects assessed for the overall follow-up period.

The number of participants at baseline was 1521 (n=760 intervention group, n=761 control group); at 1 year n=1215 (n=595 for intervention group, n=620) for all body

© 2023 Konieczna J et al. *JAMA Network Open.*

components, except for visceral fat n=1208 (n=592 for intervention group, n=616); at 3 years n=1133 (n=543 for intervention group, n=590) for all body components, except for visceral fat n=1091 (n=522 for intervention group, n=569).

© 2023 Konieczna J et al. *JAMA Network Open.*

eTable 4. Effect of the PREDIMED-Plus intervention on body composition changes (primary and secondary outcomes) over follow-up time (multiple imputations, n=100).

	Model 1	Model 2
	Between-group difference	Between-group difference
	Mean (95% CI)	Mean (95% CI)
**PRIMARY OUTCOMES**		
**Total fat mass (%)**		
Year 1 vs baseline	-0.92 (-1.19; -0.65)	-0.92 (-1.19; -0.65)
Year 3 vs baseline	-0.39 (-0.67; -0.11)	-0.39 (-0.67; -0.11)
**Total lean mass (%)**		
Year 1 vs baseline	0.86 (0.60; 1.11)	0.86 (0.60; 1.11)
Year 3 vs baseline	0.34 (0.07; 0.61)	0.34 (0.07; 0.61)
**Visceral fat (g)**		
Year 1 vs baseline	-124 (-180; -68.1)	-124 (-180; -68.0)
Year 3 vs baseline	-77.0 (-138; -16.6)	-77.0 (-138; -16.6)
**SECONDARY OUTCOMES**		
**Total fat mass (g)**		
Year 1 vs baseline	-1368 (-1740; -996)	-1368 (-1740; -996)
Year 3 vs baseline	-803 (-1225; -382)	-803 (-1225; -382)
**Total lean mass (g)**		
Year 1 vs baseline	-242 (-445; -40.3)	-242 (-445; -40.3)
Year 3 vs baseline	-431 (-645; -217)	-431 (-645; -217)
**Visceral fat (%)**		
Year 1 vs baseline	-0.10 (-0.23; 0.04)	-0.10 (-0.23; 0.04)
Year 3 vs baseline	-0.08 (-0.22; 0.06)	-0.08 (-0.22; 0.06)
**Android/gynoid fat mass**		
Year 1 vs baseline	-0.01 (-0.02; 0.00)	-0.01 (-0.02; 0.00)
Year 3 vs baseline	-0.01 (-0.03; 0.00)	-0.01 (-0.03; 0.00)
**Total lean mass/total fat mass**		
Year 1 vs baseline	0.07 (0.05; 0.09)	0.07 (0.05; 0.09)
Year 3 vs baseline	0.03 (0.01; 0.05)	0.03 (0.01; 0.05)

Percentages of fat mass and lean mass were calculated in relation to DXA-derived total body mass (sum of total bone, fat and muscle mass), whereas percentage of visceral fat was calculated in relation to total fat mass.

Three-level linear mixed models with random intercepts at recruiting centre, cluster family and individual subject were used to assess intervention group effects on changes in body composition parameters measured repeatedly over time (at each follow-up visit and for the overall follow-up period). Potential interactions of study arm with time were tested in these models:

Model 1: Without additional adjustments
Model 2: Model 1 + sex (dichotomous), age (continuous), and baseline levels of smoking status (three categories), educational level (three categories), type 2 diabetes prevalence (dichotomous), height, total leisure-time physical activity, sedentary behaviour, total energy intake and alcohol intake (continuous).

© 2023 Konieczna J et al. *JAMA Network Open.*

eFigure 4. Effect of the PREDIMED-Plus intervention on body composition changes over follow-up time by subgroups (completers-only).

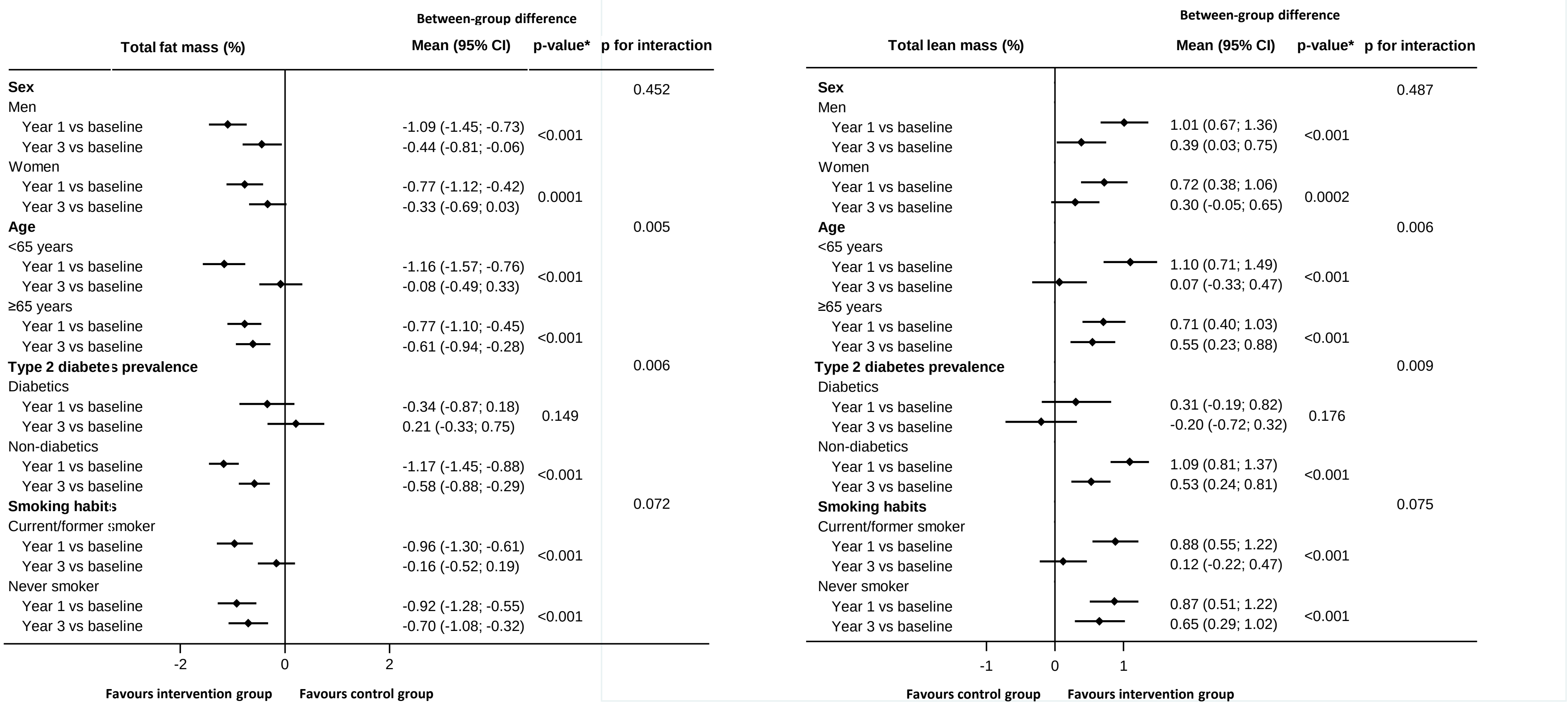

© 2023 Konieczna J et al. *JAMA Network Open.*

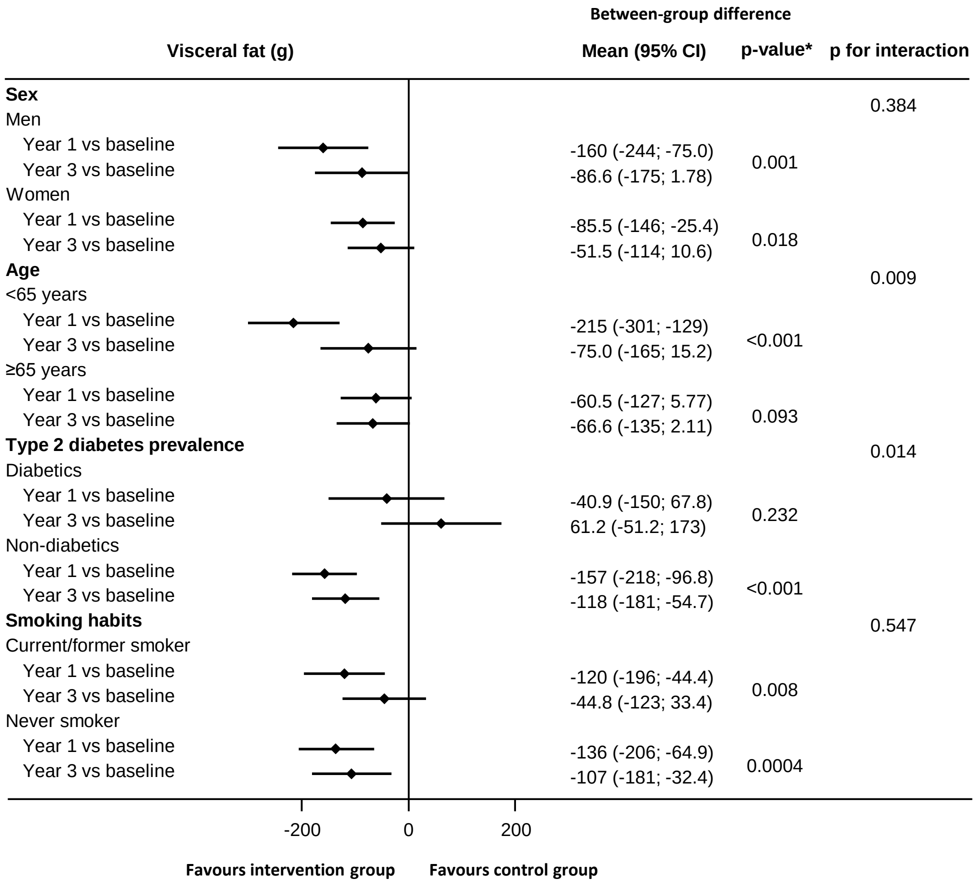

Percentages of fat mass and lean mass were calculated in relation to DXA-derived total body mass (sum of total bone, fat and muscle mass).

Three-level linear mixed models with random intercepts at recruiting centre, cluster family and individual subject were used to assess intervention group effects on changes in body composition parameters measured repeatedly over time (at each follow-up visit and for the overall follow-up

© 2023 Konieczna J et al. *JAMA Network Open.*

period) after adjusting for sex (dichotomous), age (continuous), and baseline levels of smoking status (three categories), educational level (three categories), type 2 diabetes prevalence (dichotomous), height, total leisure-time physical activity, sedentary behaviour, total energy intake and alcohol intake (continuous).

*P-value represents the intervention group effects on body composition parameters assessed for the overall follow-up period.
[§]P for interaction represents the intervention group effects on body composition parameters assessed for the overall follow-up period by corresponding baseline categories of sex, age, type 2 diabetes prevalence and smoking habits.

© 2023 Konieczna J et al. *JAMA Network Open.*
