# Supplementary material for: An Energy-Reduced Mediterranean Diet, Physical Activity, and Body Composition: An Interim Subgroup Analysis of the PREDIMED-Plus Randomized Clinical Trial
Source: JAMA Netw Open. 2023 Oct 18;6(10):e2337994. doi: 10.1001/jamanetworkopen.2023.37994 (PMC10585413; doi:10.1001/jamanetworkopen.2023.37994)
Supplement: Supplement 3. — Statistical Analysis Plan [file jamanetwopen-e2337994-s003.pdf]

SECTION 1: ADMINISTRATIVE INFORMATION

Title and trial registration

Effect of an energy-reduced Mediterranean diet, physical activity and behavioral intervention on the primary prevention of cardiovascular disease – Statistical analysis plan

Trial registered at the International Standard Randomized Controlled Trial (ISRCT; http://www.isrctn.com/ISRCTN89898870) with number 89898870 and a registration date of 24 July 2014.

SAP version: 2

SAP version 1- date November 2018

SAP version 2- date: July 2019

Protocol version

April 3rd, 2018

Available at: https://www.predimedplus.com/wp-content/uploads/2016/07/Protocolo-PREDIMED_PLUS_eng_Jan2014_12-03-2018-y-April_2018_03-04-2018.pdf

Accessed: July 2nd, 2019

SAP revisions

July 2019

Roles and responsibility

SAP contributors:

1. Person writing the SAP

   Estefanía Toledo, MD, MHP, PhD and Alfredo Gea, DPharm, PhD

   Department of Preventive Medicine and Public Health, University of Navarra

   CIBER Fisiopatología de la Obesidad y Nutrición

2. Senior statistician responsible

   Miguel A. Martínez-González, MD, PhD, MPH, Professor of Epidemiology

   Jesus López-Fidalgo, PhD, Professor of Statistics

3. Chief investigator/clinical lead: Jordi Salas-Salvadó, Miguel A. Martínez-González

Signatures

____

Estefanía Toledo

____

Alfredo Gea

____

Miguel A Martínez-González

____

Jesús López Fidalgo

____

Jordi Salas-Salvadó

SECTION 2: INTRODUCTION

Background and rationale

The completed PREDIMED (in Spanish: PREvención con DIeta MEDiterránea) cardiovascular primary prevention trial (Martinez-González et al, 2012) successfully demonstrated that long-term adherence to an unrestricted-energy Mediterranean diet (MedDiet) supplemented with extra-virgin olive oil (EVOO) or mixed nuts reduced the incidence of major clinical events of cardiovascular disease (CVD) in older individuals at high risk. Final results were republished in 2018 showing an approximately 30% relative reduction in the risk for the composite primary end-point of stroke, myocardial infarction and cardiovascular death (Estruch et al, 2018). However, the PREDIMED trial only tested changes in the composition of the overall diet, not in other lifestyle aspects such as total energy intake (as it was *ad libitum*), physical activity, or weight loss.

The rationale for the new PREDIMED-Plus randomized controlled trial (RCT) is to go beyond the intervention delivered in PREDIMED and to answer one of the most important questions for clinical practice in the context of the current unprecedented obesity pandemic (The GBD 2015 Obesity Collaborators, 2017; González-Muniesa et al, 2017): is intentional weight loss (using diet and physical activity) able to bring about a substantial reduction in clinical CVD events in the long-term? Our main hypothesis is that by addressing 3 new lifestyle factors (energy reduction with a high-quality dietary pattern, increased physical activity (PA) and weight loss) an even stronger reduction in the risk of hard CVD end-points will be attained (Martinez-González et al, 2018).

PREDIMED-Plus is expected to obtain synergy from the beneficial effects of a high-quality diet (a MedDiet) plus an intensive weight-loss intervention (using energy reduction and physical activity) on CVD incidence. This strategy should have positive effects on weight loss (focused on loss of fat mass) and long-term weight-loss maintenance, as shown in a 2-year randomized trial comparing an energy-reduced MedDiet versus low-fat versus low-carbohydrate diets (Shai et al, 2008; Schwarzfuchs et al, 2012).

Objectives

Our long-term objective is to provide effective treatment for reducing excessive CVD morbidity and mortality in overweight and obese adults, irrespective of whether the participants are diabetic at the beginning of the study. To achieve this goal, we will compare the effects on CVD rates of an intensive lifestyle and weight loss intervention program based on the traditional Mediterranean diet and including increased physical activity, energy reduction and behavioral support (intervention group) with those of a non-intensive intervention program that provides both education on the traditional Mediterranean diet for the prevention of CVD in accordance with the principles outlined in the PREDIMED trial and usual care by primary healthcare professionals (control group). The importance of attending visits to healthcare professionals will be stressed and general recommendations on management of the metabolic syndrome will be provided to the control group.

Main specific objectives

To evaluate the effect of an intensive weight-loss-oriented lifestyle intervention program based on a traditional Mediterranean diet with energy reduction, increased physical activity and behavioral therapy on 2 primary end-points:

1. The incidence of CVD (a composite of non-fatal myocardial infarction, non-fatal stroke and cardiovascular death)

2. Weight loss and long-term maintenance of weight-loss

Importantly, the 3 different components of the primary CVD end-point, namely stroke, myocardial infarction and cardiovascular death, will not be analyzed separately.

Specific secondary objectives

This intensive intervention program is likely to result in reductions of:

- waist circumference
- acute coronary syndromes
- coronary revascularization
- transient ischemic attack
- total mortality
- heart failure
- peripheral artery disease
- venous thrombosis
- atrial fibrillation
- type-2 diabetes
- complications of type-2 diabetes (diabetic nephropathy, diabetic retinopathy and diabetic polyneuropathy)
- total cancer
- cancer in main sites (breast, prostate, colorectal, lung and stomach)
- gallstone disease
- symptomatic gout
- neurodegenerative disorders (dementia and Parkinson's disease)
- unipolar depression
- osteoporotic fractures
- cataract surgery
- surgery for obesity
- eating behavior disorders

We will also address the effect of the intervention on the following intermediate outcomes: nutrient intake and adherence to an overall healthy dietary pattern, systolic and diastolic blood pressure, serum lipid concentrations, fasting glucose, glycated hemoglobin and uric acid, kidney function, liver function, C-reactive protein, anti-hypertensive, anti-diabetic and lipid-lowering medication needs, ECG traits, cognitive function, quality of life, and psychopathological scales.

We will also store plasma, serum, peripheral cells and urine samples to evaluate other hypotheses in the future, depending on availability of additional funding.

SECTION 3: STUDY METHODS

Trial design

The PREDIMED-Plus trial is a 6-year parallel-group, multicenter RCT involving 6,874 participants recruited in 23 Spanish recruiting centers. The main aim is to assess the effect of an intensive weight-loss intervention based on an energy-reduced Mediterranean diet (erMedDiet), PA promotion, and behavioral support on CVD events in comparison with a control group receiving usual care, including the recommendation to follow an unrestricted-energy MedDiet without advice to increase PA. The primary end point is a combination of CVD events (non-fatal myocardial infarction, non-fatal stroke and cardiovascular death).

A detailed description of the intervention can be found in the trial protocol (https://bit.ly/2OZsv3n).

Randomization

For the baseline visit, each recruiting center randomly assigned eligible candidates to one of two groups, intensive intervention group or usual care (control) group, using a centrally-controlled, computer-generated random-number system (available at: www.predimedplus.com). The University of Navarra, Department of Preventive Medicine and Public Health was responsible for the randomization procedure by which participants were randomly assigned with stratification by center, sex, and age group (<65, 65-70, >70 years) in blocks of 6. However, during the randomization stage, centers and staff were blinded to this block size in order to ensure absolutely blinded randomization. Members of the same household were randomized together. The recruiting centers entered the participants' identification criteria into the internet-based system in a blind manner, without any possible foretelling of the group that the participant will be allocated. Therefore, a completely blinded randomization procedure was used. The system automatically assigned each participant or members of the same household to their allocated groups according to a random and unpredictable algorithm, out of the control of any staff involved in the trial.

Once this occurred, the assigned group could not be changed. In the specific cases of other members of the same household who were recruited at a different time than the first recruited member of the same household, the last member of the same household entering the study was assigned (not randomized) to the same study arm as the first member of that household in order to ensure high compliance with the intervention, peer support, and also to avoid contamination and potential conflicts between members of the same household.

Sample size

We will determine the effect of the intensive weight-loss lifestyle intervention with an energy-reduced Mediterranean diet on the two primary outcomes below, assuming a two-tailed alpha error of 0.05.

1. Effect of the intervention on incident CVD (exclusively, a composite non-fatal myocardial infarction, non-fatal stroke, and cardiovascular death). The cumulative projected incidence after including as primary events the cardiovascular composite of stroke, cardiovascular deaths

and all non-fatal acute myocardial infarctions with positive high-sensitivity troponin tests after 6 years will be at least 10% in the control group, based on the observed results in the PREDIMED trial after 4.8 years (which did not include high-sensitivity troponin tests). The hazard ratio (HR) for the combined primary endpoint is anticipated to be 0.70[2] and will probably be even lower (greater protective effect) after considering that in the PREDIMED trial no energy reduction was implemented, physical activity was not encouraged, and weight loss was not a target of the intervention. Under these assumptions, therefore, even if the dropout rates were to reach 20%, the required sample size would be 2,400 per group (see Figure 1). To be conservative, however, we planned to recruit 6,000 participants and assign 3,000 participants to each group. The participants were recruited at 23 recruiting centers, each of which had the goal of recruiting, educating and following approximately 300 participants, 150 in the control group and 150 in the intensive intervention group.

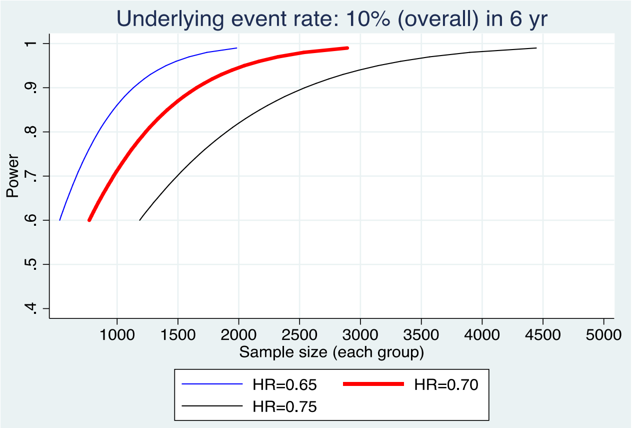

Figure 1. Estimation of the sample size required per intervention group in the PREDIMED-PLUS trial

2. Effect of the intervention on weight change. Based on previous studies, we can expect a minimum weight change for participants in the control group and a weight loss of 3-4.5 kg for those in the intensive lifestyle intervention group, with a standard deviation of 8 kg (Shai et al, 2008; Sacks et al, 2009; Look AHEAD Research Group, 2010). Assuming that our intervention will have only a small effect on weight change and calculating the sample size according to a weight change of 1 kg in the usual care group, a weight change of 3 kg in the intensive lifestyle intervention group (net difference = 2 kg), and a standard deviation of 8 kg, in order to achieve a statistical power of 0.80 we would need a sample size of only 337 in each group. Since the number of participants to be recruited is much higher than this figure, the statistical power needed to reach this objective is largely guaranteed.

Framework

Framework: superiority hypothesis testing.

Assessment of primary, secondary and intermediate outcomes will be based on this framework.

Statistical interim analyses and stopping guidance

Data from the PREDIMED-PLUS trial will be analyzed after 3 years of median follow-up, after 5 years of median follow-up, and at the end of the trial. For methodological reasons but especially for ethical motives, suitable follow-up for a trial must include at least one interim analysis (Schulz and Grimes, 2005). However, to preserve an overall alpha error of 0.05, interim analyses have to be penalized. We will use the O'Brien and Fleming boundaries (O'Brien and Fleming, 1979). With this method, the boundaries are stricter at the earlier stages of the study than at the later ones. Applying this rule leads to the following p values for stopping the trial (Schulz and Grimes, 2005):

- First interim analysis (median follow-up: 3 years); threshold p value: 0.0005.
- Second interim analysis (median follow-up: 5 years); p value: 0.014.
- Final analysis (median follow-up: 8 years); p value: 0.045

Timing of final analysis

Active intervention will be implemented during the first 6 years of trial duration (this 6-year period does not correspond to the median follow-up time, because all participants will homogeneously complete 6 years of intervention) and they will be followed-up for two further years to assess incident outcomes (Table 1). Thus, the total duration of the trial will be 8 years for each participant.

Information will be collected for 8 years after the baseline visit date for each participant.

Overall, hard clinical end-points will be assessed only after completion of the 8-year follow-up period. The only exceptions will be:

a) Type 2 diabetes–follow-up 7 years: according to the Diabetes Prevention Impact Toolkit (https://nccd.cdc.gov/Toolkit/DiabetesImpact/), the expected number of incident type 2 diabetes cases among participants initially free of diabetes during the first 7 years is high (620), so that we expect to have sufficient statistical power after 7 years of follow-up.
b) Parkinson's disease–follow-up 10 years: the incidence of Parkinson's disease is lower than for other reported outcomes so that participants will be followed for 10 years to ensure a high-enough statistical power
c) Cancer: we will consider not only overall cancer but also specific cancers as outcomes. In order to have a sufficient number of cases after breaking down by cancer location and to allow for a long-enough induction time, participants will be followed-up for 10 years.

Table 1. Timeline for reported outcomes

	Follow-up						
**Outcomes**	**M-6**	**Y-1**	**Y-6**	**Y-7**	**Y-8**	**Y-9**	**Y-10**
**Primary**							
1. Composite endpoint of cardiovascular death, non-fatal myocardial infarction or stroke					X		
2. Body weight change	X	X	X				
**Secondary**							
3. Death from any cause					X		
4. Waist circumference change	X	X	X				
5. Incidence of acute coronary syndrome (unstable angina)					X		
6. Incidence of coronary revascularization (percutaneous or surgical)					X		
7. Incidence of atrial fibrillation					X		
8. Incidence of peripheral artery disease					X		
9. Incidence of heart failure					X		
10. Incidence of type-2 diabetes				X			
11 Incidence of type-2 diabetes complications (diabetic nephropathy, diabetic retinopathy and diabetic polyneuropathy)					X		
12. Incidence of dementia/Alzheimer's disease					X		
14. Incidence of other dementias, diagnosed when there are cognitive or behavioral (neuropsychiatric) symptoms (see protocols)					X		
15. Incidence of Parkinson's disease							X
16. Incidence of major unipolar depression					X		
17. Incidence of osteoporotic fractures					X		
18. Incidence of gallstone disease or cholecystectomy					X		
19. Incidence of symptomatic gout					X		
20. Incidence of transient ischemic attack					X		
21. Incidence of cataract surgery					X		
22. Incidence of venous thromboembolism					X		
23. Incidence of total cancer and specific cancers in main cancer sites (breast, prostate, lung, colorectal, or stomach)							X

**Secondary (continued):**	**M-6**	**Y-1**	**Y-6**	**Y-7**	**Y-8**	**Y-9**	**Y-10**
24. Eating behavior disorders					X		
25. Surgery for obesity					X		
**Other intermediate outcomes are changes in:**							
27. Overall diet (17-item score of adherence to the energy-reduced Mediterranean diet and 14-item score of Mediterranean diet) and nutrient intake	X	X	X				
28. Blood pressure (Systolic[1] and Diastolic blood pressure)	X	X	X				
28. Fasting blood glucose and hemoglobin A1C levels	X	X			X		
29. Serum lipid concentrations (triglycerides[2], cholesterol, and HDL[3] and LDL[4] cholesterol)	X	X			X		
30. Renal function (changes in estimated glomerular filtration rate (eGFR) and urine microalbumin-to-creatinine ratio (UACR), incidence and reversion of chronic kidney disease (CKD, eGFR<60 ml/min/1.73m^2) and microalbuminuria (UACR≥30 mg/g))		X	X[#]		X		
31. Uric acid levels					X		
32. Liver function (liver fat content and non-invasive markers of liver status such as aspartate aminotransferase, alanine aminotransferase, gamma-glutamyltransferase)					X		
33. Inflammation markers (C-reactive protein and white blood cell counts and its subtypes (neutrophil, lymphocyte, monocyte, basophils, and eosinophil))					X		
34. Other intermediate markers of cardiovascular risk (circulating levels of fasting serum insulin, leptin, C-peptide, hs-CRP, interleukin-6 (IL-6), IL-8, IL-18, tumor necrosis factor-α, MCP-1, and regulated on activation, normal T-cell expressed and secreted cytokines		X					
35. Results of ECGs and alterations of the cardiac rhythm					X		
36. Quality of life (Short -Form 36 quality of life scale)				X			
37. Cognitive function (including 6 tests: Mini-Mental State Examination, clock drawing test, semantic and phonemic verbal fluency test, the reverse series of digits test (WAIS-III) and trail making test)			X				
38. Psychological and neuropsychological scores (including 3 tests: Beck Depression Inventory (BDI-II), multidimensional scale of weight locus control and screening for comorbid eating disorders with diagnostic criteria)			X				
39. Medication use (anti-hypertensive, anti-diabetic and lipid-lowering medication)			X				

**Other intermediate outcomes are changes in (continued):**	**M-6**	**Y-1**	**Y-6**	**Y-7**	**Y-8**	**Y-9**	**Y-10**
40. Total physical activity (PA), light-PA, and moderate-to-vigorous PA measured by periodically administered REGICOR Short Physical Questionnaire (self-reported data)	X	X	X				
41. Reversal of obesity, metabolic syndrome (MetS) and specific criteria for the MetS (abdominal obesity, hypertriglyceridemia, low HDL-cholesterol levels, high blood pressure, and high fasting plasma glucose concentrations).				X			
42. Total physical activity (PA), light-PA, and moderate-to-vigorous PA measured using objective methods, such as accelerometry			X				
43. Bone density and body composition measured with DXA*			X				
44. Ambulatory blood pressure, obtained by 24-hour ABPM			X				
**Other sub-studies**							
45. Analysis of the results in relation to genetic studies (DNA and mRNA)				X?			
46. Epigenetic studies and overexpression of microRNAs				X?			
47. Composition and function of intestinal microbiota by pyrosequencing		X*					
48. Metabolomics, transcriptomics and proteomics				X?			

*At 1 year and 3 years of follow-up. [#]At 5 years of follow-up.

1: Besides assessing systolic blood pressure as a continuous trait, we will also assess a 5 mm Hg systolic blood pressure reduction as a clinically meaningful change (Stamler et al, 1991).

2: Besides assessing triglycerides as a continuous trait, we will also assess a 10% triglyceride reduction as a clinically meaningful change. The reduction of serum triglycerides has been associated with reduced coronary heart disease rates in clinical trials of hypotriglyceridemic agents (Miller, 2011), but no algorithm relating percent triglyceride decrease to percent risk reduction has been developed. In the general population, there is a graded increase in risk with increasing fasting or nonfasting triglycerides (Nordestgaard, 2014), hence a 10% reduction can be considered clinically meaningful.

3: Besides assessing HDL-cholesterol as a continuous trait, we will also assess a 5% HDL-cholesterol increase as a clinically meaningful change (Gordon, 1989)

4: Besides assessing LDL-cholesterol as a continuous trait, we will also assess a 5% LDL-cholesterol reduction as a clinically meaningful change (Cholesterol Treatment Trialists' Collaboration, 2010)

Timing of outcome assessments

Weight and waist circumference are measured in duplicate by trained study personnel at the yearly follow-up visits.

Other primary and secondary outcomes will be ascertained yearly by systematic review of the participants' medical charts by medical doctors who are blinded to the intervention group. Codified copies of the medical reports in which the outcome is described will be sent to the Event Ascertainment Committee whose members will confirm the outcome.

Blood specimens are collected at odd-year visits and after 8 years of follow-up.

SECTION 4: STATISTICAL PRINCIPLES

Confidence intervals and p values

Primary outcomes:

Given that the list of primary outcomes has been defined *a priori* and that there is substantial evidence that suggests a potential beneficial effect of the intervention on the considered outcomes, we understand that no adjustment for multiplicity will be necessary. We will present point estimates together with 95% confidence intervals. P-values below 0.05 will be deemed as statistically significant.

Secondary outcomes:

We will present two forms of confidence intervals for the secondary outcomes: nominal confidence intervals and, only as ancillary analyses, multiple-testing-adjusted confidence intervals. Nominal 95% confidence intervals will describe results from a single outcome assessment. We will also estimate multiple-testing-adjusted confidence intervals based on the Bonferroni procedure ($1-\alpha/m$ confidence intervals, where m is the number of comparisons[1]) for secondary outcomes, where m is the total number of secondary outcomes, namely m=27. Our reports will primarily focus on nominal confidence intervals based on coherence and biological plausibility for interpretation of our findings, but we will also add multiple-testing-adjusted confidence intervals for secondary outcomes, only as ancillary analyses.

Adherence

Adherence in the control group will be defined as achieving a score of at least 10 points in the 14-item screener of adherence to the traditional Mediterranean diet (Schröder et al, 2011).

[1] The total number of comparisons will be 27: 1) death from any cause, 2) change in waist circumference, 3) acute coronary syndrome (unstable angina) or coronary revascularization (percutaneous or surgical), 4) atrial fibrillation, 5) peripheral artery disease, 6) heart failure, 7) type-2 diabetes, 8) diabetic nephropathy, 9) diabetic retinopathy, 10) diabetic polyneuropathy, 11) overall dementia and Alzheimer's disease 12) Parkinson's disease, 13) Major unipolar depression, 14) osteoporotic fractures, 15) gallstone disease or cholecystectomy, 16) symptomatic gout, 17) transient ischemic attack, 18) cataract surgery, 19) venous thromboembolism, 20) total cancer, 21) breast cancer, 22) prostate cancer, 23) lung cancer, 24) colorectal cancer, 25) stomach cancer, 26) eating behavior disorder, and 27) surgery for obesity.

Adherence in the intervention group will be defined as:

1) weight loss in comparison with baseline weight,
2) increased physical activity according to self-reported leisure-time physical activity (Minnesota questionnaire assessing METS-min/wk) or improvement in physical fitness according to the chair-test, and
3) achieving a score of at least 12 points in the 17-item screener of adherence to an energy-reduced Mediterranean diet.

Analysis populations

Main analyses will be conducted based on an intention-to-treat approach (each participant will remain in the randomly allocated group). Intention-to-treat analyses will be conducted based on a) participants with full data only and b) analysis with multiple imputation for missing data.

For the per-protocol analysis for weight change, participants in the intervention group will be censored if:

a) they show a persistent score of adherence to the energy-reduced Mediterranean diet below 12 points in two consecutive yearly assessments, or
b) they show a persistent decrease in physical activity (self-reported information) and physical fitness (chair test) compared with the baseline information in two consecutive yearly assessments.

and participants in the control group will be censored if they show a persistent score of adherence to the Mediterranean diet (14-item score, based on *ad libitum* energy intake) below 10 points in two consecutive yearly assessments.

In the per-protocol analysis for other outcomes, participants in the intervention group will be censored if:

c) they show a persistent score of adherence to the energy-reduced Mediterranean diet below 12 points in two consecutive yearly assessments, or
d) they show a persistent decrease in physical activity (self-reported information) and physical fitness (chair test) compared with the baseline information in two consecutive yearly assessments, or
e) they show a persistent weight gain compared with the baseline weight in two consecutive yearly assessments

and participants in the control group will be censored if they show a persistent score of adherence to the Mediterranean diet (14-item score, based on *ad libitum* energy intake) below 10 points in two consecutive yearly assessments.

We will also perform analysis on an as-treated basis, classifying participants according to their adherence to Mediterranean diet, weight loss and physical activity level, independently of their assigned intervention. This analysis will be complemented with formal analyses of mediation for the primary outcome (CVD). We will adapt the approaches proposed by Lange (Lange *et al*, 2011) and Lin (Lin *et al*, 2017), including time-varying mediators and confounders. These approaches model the mediation effect in a counterfactual framework and can estimate the direct effect and indirect effects of the lifestyle intervention on CVD risk. Thus, we can evaluate the mediation effects of both weight loss and through improvement in diet quality and physical fitness beyond the effects of weight loss.

SECTION 5: TRIAL POPULATION

Screening data

Table 2 shows the characteristics of the participants who attended the first screening visit but were not finally included in the trial and the participants who were finally randomized.

Table 2. Description of participants who attended the first screening visit but were not finally included in the trial and participants who were randomized.

Characteristics at baseline	Non-randomized	Randomized	p value
N	2803	6874	-
Age (mean years, SD)	65.9 (5.1)	64.9 (4.9)	<0.001
Female sex (%)	53.8	48.5	<0.001
Baseline weight (mean kg, SD)	84.4 (14.0)	87.0 (13.0)	<0.001
Baseline waist (mean cm, SD)	109.9 (9.8)	110.4 (8.6)	0.005
Waist-to-height ratio	66.2 (6.5)	66.4 (5.5)	0.090
Baseline BMI (kg/m^2; mean, SD)	32.4 (4.3)	32.7 (3.4)	<0.001
Obesity (%)	66.4	75.1	<0.001
Smoking			
Current smoker (%)	13.3	12.5	0.261
Former smoker (%)	35.6	43.4	<0.001
Self-reported diabetes (%)	29.0	27.2	0.074
Family history of premature CHD (%)	14.4	16.8	0.004
High blood cholesterol (%)	67.9	69.3	0.178
Total cholesterol (mean mg/dl, SD)	201.8 (43.6)	202.6 (40.0)	0.375
LDL cholesterol (mean mg/dl, SD)	122.7 (34.4)	123.9 (34.1)	0.163
HDL cholesterol (mean mg/dl, SD)	49.8 (13.3)	47.8 (11.8)	<0.001
Triglycerides (mean mg/dl, SD)	161.7 (84.8)	170.3 (91.3)	<0.001
Glucose (mean mg/dl, SD)	114.6 (32.7)	114.8 (30.7)	0.794
Hypertension (%)	77.8	83.1	<0.001
Non-European origin (%)	2.9	2.5	0.318
Willingness to change diet (mean, SD)	2.5 (0.7)	2.7 (0.5)	<0.001

Eligibility

Candidates for the PREDIMED-PLUS trial (Martínez-González et al, 2018) were adults aged 55-75 years for men and 60-75 years for women with a body mass index ≥27 and <40 kg/m^2 who met at least three criteria for the metabolic syndrome (Alberti et al, 2009). These criteria must be taken into consideration in view of evidence of the beneficial role of the Mediterranean diet on metabolic syndrome (Kastorini et al, 2011, Babio et al, 2014), insulin

resistance and diabetes (Salas-Salvadó et al, 2014), especially when accompanied by a program of physical activity for endurance (Fernández et al, 2012). Approximately, 50% of the study population is made of women and diabetic participants do not exceed 27% of the total cohort. Individuals who participated in the PREDIMED trial were not eligible to participate in PREDIMED-PLUS.

Recruitment

Figure 2 shows the flow-chart of participants in the PREDIMED-Plus trial.

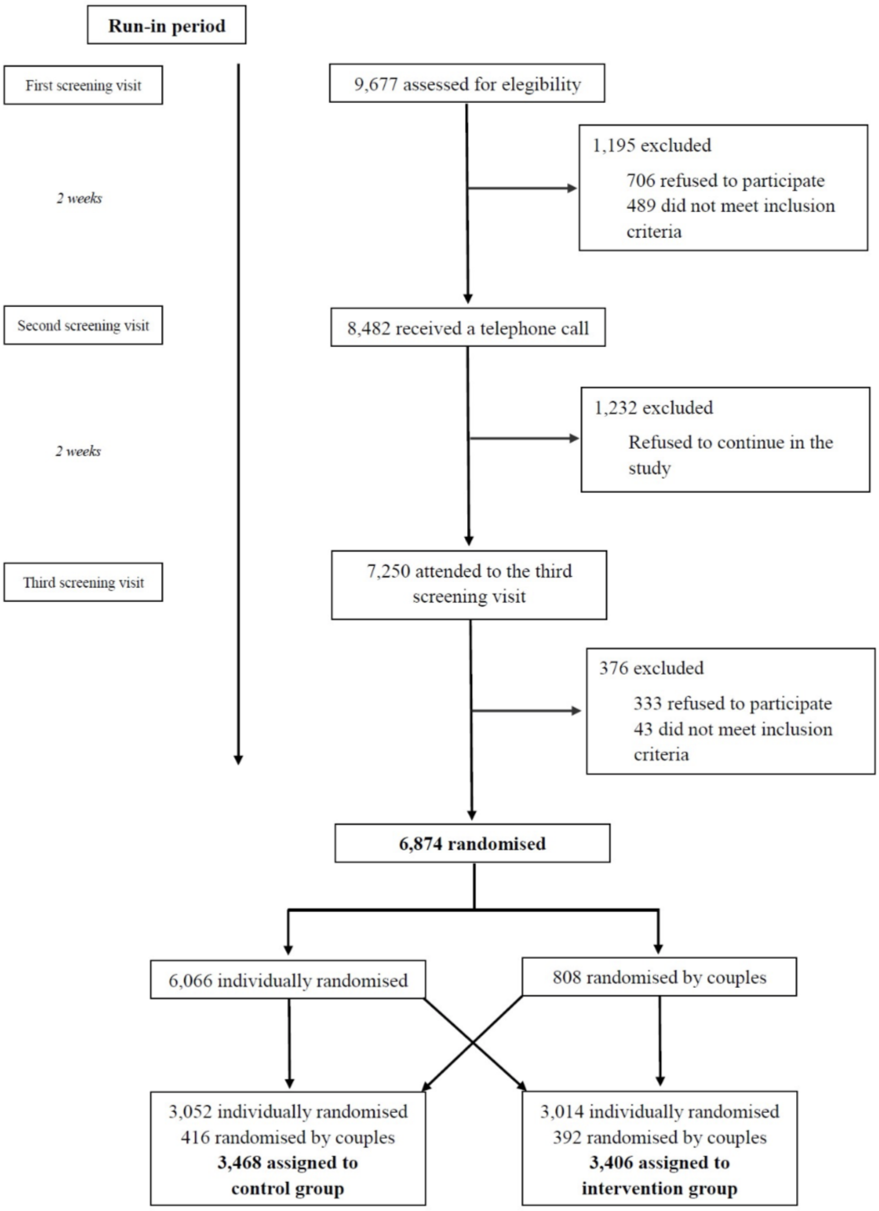

Figure 2. Flow-chart of participant recruitment and randomization in the PREDIMED-Plus trial.

Information on follow-up will be added to this flow-chart for final analyses.

Withdrawal/follow-up

Follow-up will be based on:

- yearly follow-up visits
- yearly systematic review of medical charts
- consultation of the National Death Index for participants with no information on follow-up for 1 year

Participants will be considered to be lost-to-follow-up if there is no available information in the above-mentioned sources of information for at least 2 years.

Participants who withdraw have several alternatives such as:
- no longer willing to be contacted but not reluctant to have their medical charts reviewed. In this case, participants will be follow-up based on the available information in their medical records
- explicitly asking to cancel their participation or withdraw their consent: they will be considered withdrawals and their future information will no longer be accessed.

The number of participants in each of these categories will be included in the study flow-chart. Information on losses-to-follow-up and withdrawals according to allocation group will be summarized as:
- number of participants who were lost-to-follow-up for at least 2 years
- participants who asked to cancel their participation or withdrew their consent.

Baseline participants' characteristics

Description of participants' baseline characteristics will include:

- Number of participants in each intervention group
- Number of participants individually randomized and number of participants randomized together with another person from the same household

A. Qualitative traits: summarized with number and percentages:
    - Female sex
    - Obesity
    - Smoking status: never, former, current
    - Self-reported baseline diabetes
    - Self-reported family history of coronary heart disease
    - Self-reported baseline high blood cholesterol
    - Self-reported baseline hypertension
    - Self-reported previous depression
    - Educational level
    - Origin: European vs. non-European
    - Marital status
    - Living alone
    - Being retired
    - Previous weight-loss dieting

B. Quantitative traits: summarized with means and standard deviations:

- Age
- Baseline weight
- Baseline waist circumference
- Baseline waist-to-height ratio
- Baseline body weight
- Total cholesterol
- LDL cholesterol
- HDL cholesterol
- Triglycerides
- Glucose
- Systolic blood pressure
- Diastolic blood pressure
- Leisure-time physical activity
- Chair test
- Adherence to the energy-restricted Mediterranean diet
- Adherence to the traditional Mediterranean diet
- Total energy intake
- Total fat intake
- Carbohydrate intake
- Protein intake
- Alcohol intake
- Dietary fiber intake
- Willingness to change diet

SECTION 6: ANALYSIS

Outcome definitions

A. Primary outcomes

1. Non-fatal acute coronary syndrome (acute myocardial infarction), non-fatal stroke or cardiovascular mortality.

1.a. Acute myocardial infarction (MI) are defined according to the third universal definition of MI on behalf of the Joint ESC/ACCF/AHA/WHF Task Force as evidence of myocardial necrosis in a clinical setting consistent with acute myocardial ischemia.

Any one of the following criteria meets the diagnosis for MI:

- Detection of a rise and/or fall of cardiac biomarker values [preferably cardiac troponin (cTn)] with at least one value above the 99th percentile upper reference limit (URL)

AND

- At least one of the following:

  (i) Symptoms of ischemia.

  (ii) New or presumed new significant ST-segment–T wave (ST–T) changes or new left bundle branch block.

(iii) Development of pathological Q waves in the ECG.

(iv) Imaging evidence of new loss of viable myocardium or new regional wall motion abnormality.

(v) Identification of an intracoronary thrombus by angiography.

Prior MI

Any one of the following criteria meets the diagnosis for prior MI:

• Pathological Q waves with or without symptoms in the absence of non-ischemic causes.

• Imaging evidence of a region of loss of viable myocardium that is thinned and fails to contract, in the absence of a non-ischemic cause.

• Pathological findings of a prior MI

1.b. Stroke is defined as an acute neurological deficit lasting more than 24 hours caused by an abrupt impairment of brain function due to blockage of blood flow in a particular artery supplying the brain (thrombosis or arterial embolism) or a cerebral haemorrhage.

Ischemic Stroke is defined following the updated definition of stroke for the 21st Century: A Statement for Healthcare Professionals from the American Heart Association/American Stroke Association as an episode of neurological dysfunction caused by focal cerebral, spinal, or retinal infarction. Central nervous system (CNS) infarction is brain, spinal cord, or retinal cell death attributable to ischemia, based on:

1. Pathological, imaging, or other objective evidence of cerebral, spinal cord, or retinal focal ischemic injury in a defined vascular distribution;

2. Clinical evidence of cerebral, spinal cord, or retinal focal ischemic injury based on symptoms persisting ≥24 hours or until death, and exclusion of other potential causes such as hypoglycaemia or seizures.

Silent CNS infarction are not considered as a primary end-point if defined as imaging or neuropathological evidence of CNS infarction without a history of acute neurological dysfunction attributable to the lesion.

Hemorrhagic Stroke. Stroke caused by intracerebral hemorrhage is defined as rapidly developing clinical signs of neurological dysfunction attributable to an intracerebral hemorrhage, defined as a focal collection of blood within the brain parenchyma or ventricular system that is not caused by trauma. Stroke caused by subarachnoid hemorrhage is defined as a rapidly developing signs of neurological dysfunction and/or headache because of bleeding into the subarachnoid space, which is not caused by trauma.

Silent cerebral hemorrhage is not considered as primary end-point. It is defined as a focal collection of chronic blood products within the brain parenchyma, subarachnoid space, or ventricular system detected at neuroimaging or neuropathological examination that is not caused by trauma and without a history of acute neurological dysfunction attributable to the lesion.

1.c. Cardiovascular mortality: Includes sudden death and non-sudden cardiovascular death.

Sudden (cardiac) death is due to cessation of cardiac activity with hemodynamic collapse, typically due to sustained ventricular tachycardia/ventricular fibrillation. It may be:

— Witnessed instantaneously in a previously stable patient. This may occur with or without preceding signs or symptoms, or may occur immediately following sudden dyspnea, light-headedness, or palpitations.

— Unwitnessed. Patient found dead who at the time of last witnessed contact was in his/her usual state of health without medical complaints or obvious difficulty. This applies to patients dying during sleep.

Non-sudden cardiac death: Includes deaths of patients from acute pulmonary edema with severe, progressive heart failure, cardiogenic shock, or after a recent cardiac surgical procedure.

Non-cardiac vascular death: Includes deaths due to thromboembolic events, stroke, dissecting aneurysm and peripheral artery disease.

2. Weight change. The study nurse records weight in duplicate at each follow-up visit. The measurements are made according to the study manual of operations and with participants dressed in light clothing and no shoes and accessories. The mean of the two measurements will be used.

B. Secondary outcomes

1. Total mortality. This endpoint comprises all causes of death, including those from CVD (see point 1c of primary end-point), as well as trauma, renal failure, neoplasia, sepsis, suicide and death of undetermined cause. All deaths will be confirmed by reviewing the National Death Index.

2. Changes in waist circumference. The study nurse measures waist circumference at each follow-up visit according to the manual of operations.

3. Non-ST-segment elevation acute coronary syndrome (unstable angina): The diagnosis of unstable angina is made following the definition of the ESC Guidelines for the management of acute coronary syndromes in patients presenting without persistent ST-segment elevation; It requires the presence of at least one of the following clinical characteristics:

a. Prolonged (>20 min) anginal pain at rest.

b. New onset (de novo) angina (Class II or III of the Classification of the Canadian Cardiovascular Society).

c. Recent destabilization of previously stable angina with at least Canadian Cardiovascular Society Class III angina characteristics (crescendo angina).

4. Coronary revascularization (percutaneous or surgical): The two main indications for percutaneous or surgical revascularization are:

1) Patients with unstable angina or non-ST-segment elevation acute coronary syndrome.

2) Patients considered likely to benefit from such surgery on the basis of the location and severity of chest pain, the number of vessels affected, and the presence of left ventricular dysfunction.

5. Heart failure. Acute and chronic heart failure (HF) is a syndrome in which patients have typical symptoms and signs resulting from an abnormality of cardiac structure or function. The cardinal manifestations of HF are dyspnea and fatigue, which may limit exercise tolerance, and fluid retention, which may lead to pulmonary and/or splanchnic congestion and/or peripheral edema.

5.a. The diagnosis of HF with Reduced Ejection Fraction requires three conditions to be satisfied: 1. Symptoms typical of HF; 2. Signs typical of HF and 3. Reduced ejection fraction (< 40%)

5.b. The diagnosis of HF with Preserved Ejection Fraction requires four conditions to be satisfied: 1. Symptoms typical of HF; 2. Signs typical of HF; 3. Normal or only mildly reduced left ventricular ejection fraction and non-dilated left ventricle; and 4. Relevant structural heart disease (left ventricular hypertrophy/left atrium enlargement) and/or diastolic dysfunction

5.c. A Heart Failure event may include hospitalization or an urgent outpatient visit. In this setting the event needs to meet ALL of the following criteria:

- The patient exhibits documented new or worsening symptoms of HF on presentation, including at least ONE of the following: Dyspnea, decreased exercise tolerance, fatigue or other symptoms of worsened end-organ perfusion or volume overload.

- The patient has objective evidence of new or worsening HF, consisting of at least TWO physical examination findings OR one physical examination finding and at least ONE laboratory criterion), including: Physical examination findings considered to be due to heart failure, including new or worsened peripheral edema, increasing abdominal distention or ascites (in the absence of primary hepatic disease), rales/crackles/crepitations at pulmonary auscultation, increased jugular venous pressure and/or hepatojugular reflux, S3 gallop, and clinically significant or rapid weight gain thought to be related to fluid retention

- Laboratory evidence of new or worsening HF, if obtained within 24 hours of presentation, including: Increased B-type natriuretic peptide (BNP)/ N-terminal pro-BNP (NT-proBNP) concentrations OR cardiological evidence of pulmonary congestion OR echocardiographic data of congestion or decreased cardiac output.

- The patient receives initiation or intensification of specific treatment for HF.

6. Peripheral artery disease. Ascertainment is made according to the Inter-Society Consensus for the Management of Peripheral Arterial Disease (TASC II) and ESC Guidelines for the diagnosis of peripheral artery disease. For participants with intermittent claudication, aged 60-69 with one cardiovascular risk factor, or aged ≥70 years and a resting ankle-brachial systolic pressure index ≤0.90, or an abnormal echo-Doppler examination, magnetic resonance imaging, or arteriography are considered as diagnostic (confirmed case).

7. Venous thromboembolism (VTE): all VTE need to satisfy the standard diagnosis criteria for venous thrombosis or Pulmonary (thromb-) Embolism (PE) in the general population (see below 1-3). The diagnosis should be confirmed by objective imaging techniques (including echography, phlebography, pulmonary computed tomography angiography (angioCTA), NMR, etc.) and not only be based on the clinical suspicion.

Standard diagnosis criteria for VTE in clinical studies:

1. Deep venous thrombosis, defined as the loss of venous compressibility or the inability of filling the deep vein intraluminal segment at the lower/upper limbs, as detected by echography with venous compression or phlebography, respectively.

- The presence of thrombus at the distal lower limb (distal from the popliteal vein) qualifies for primary VTE only if it is asymptomatic.

- All proximal thrombus qualify for final primary end-point if detected by imaging techniques (echography or radiology), regardless of whether it is or not asymptomatic.

2. Pulmonary Embolism (PE) is defined as:

Contrast pulmonary arteriography:

- Defects in intraluminal filling, as contrasted with two projections.

- Sudden stoppage of the contrast in one or several vessels with a diameter greater than 2.5 mm

- Pulmonary scintigraphy based on ventilation/perfusion (V/Q):

o A V/Q-pulmonary scintigraphy with high probability of PE in patients with no low clinical probability of PE.

- Pulmonary angiography using computed tomography:

o Defects in filling sub-segmental or more proximal vessels

3. Fatal PE is defined as:

- Death exclusively caused by PE and/or its confirmation at autopsy or using radiology techniques

Important considerations:

a) Superficial venous thrombophlebitis should not be described as VTE.

b) It is highly recommended to describe VTE according to the anatomic position:

- Lower limbs

- Upper limbs

- Pulmonary embolism

- Others: vessels at the splanchnic level, cerebral veins, etc.

c) The description of the VTE is highly convenient (for instance, distal to popliteal vein vs. proximal VTE; sub-segmental level vs. central PE)

d) VTE associated with a central catheter (for instance, deep venous thrombosis at the upper limbs) should be reported separately.

e) Incidental VTE should be differenced from any other symptomatic events.

8. Atrial fibrillation (AF): AF is defined following the Guidelines of the American College of Cardiology Foundation/American Heart Association Task Force on Practice Guidelines together with the European Society of Cardiology, the European Heart Rhythm Association, and the Heart Rhythm Society, as a cardiac arrhythmia with the following characteristics:

(1) The surface ECG shows 'absolutely' irregular RR intervals, i.e., RR intervals that do not follow a repetitive pattern.

(2) There are no distinct P waves on the surface ECG. Some apparently regular atrial electrical activity may be seen in some EKG leads, most often in lead V1.

(3) The atrial cycle length (when visible), i.e., the interval between two atrial activations, is usually variable and <200 ms (>300 bpm).

9. Type 2-diabetes. New-Onset Type 2 Diabetes cases are diagnosed following the recommendations of the American Diabetes Association:

1. HbA1C ≥6.5%. This test should be performed in a laboratory using a method that is National Glycohemoglobin Standardization Program (NGSP) certified and standardized to the DCCT assay. OR

2. Fasting plasma glucose (FPG) ≥126 mg/dL (7.0 mmol/L). Fasting is defined as no caloric intake for at least 8 hours OR

3. Two-hour plasma glucose (PG) ≥ 200 mg/dL (11.1 mmol/L) during an oral glucose tolerance test (OGTT). This test should be performed as described by the WHO, using a glucose load containing the equivalent of 75 g anhydrous glucose dissolved in water OR

4. In a patient with classic symptoms of hyperglycemia or hyperglycemic crisis, a random plasma glucose ≥200 mg/dL (11.1 mmol/L).

In the absence of unequivocal hyperglycemia, results should be confirmed by repeat testing according to the guidelines of the American Diabetes Association.

10. Type-2 diabetes complications. Participants are assessed yearly for microvascular complications of diabetes:

1. Diabetic nephropathy: Kidney disease in diabetes is defined based on the alteration of glomerular filtration rate (GFR) and /or the presence of persistent albuminuria at levels of 30 mg/24 h or more (normal albumin excretion is currently defined as < 30 mg/24 h). GFR is estimated through a quantitative formula, the CKD-Epi equation, that measures the progression of kidney involvement. Persistent albuminuria is determined by the urine albumin to creatinine ratio (normal <30 mg albumin/g creatinine) in a routine

morning urine sample. Because of variability in urinary albumin excretion, two of three morning specimens collected within a 3- to 6-month period should be abnormal before considering a patient to have developed increased urinary albumin excretion or a progression of albuminuria. The presence of one or two of the above criteria indicates renal disease in these patients and the requirement for appropriate follow-up for progression of renal disease according to the guidelines of the American Diabetes Association.

2. Diabetic retinopathy: Diagnosed by ophthalmologic examination and/or treatment with laser photocoagulation according to the guidelines of the American Diabetes Association.

3. Diabetic polyneuropathy: Diagnosed by clinical symptoms, neurological examination and results of electrophysiological studies of peripheral nerves according to the guidelines of the American Diabetes Association.

*Kidney damage defined as abnormalities in urine, blood, or imaging tests.

11. Cancer. All cancers except non-melanoma skin cancer are considered. Cancer cases are coded according to the International Classification of Diseases (ICD 10) of the World Health Organization.

12. Dementia/Alzheimer’s disease. Cases are ascertained according to the Recommendations from the National Institute on Aging and the Alzheimer´s Association workgroup (McKhann et al, , 2011) or if a diagnosis of dementia is reported by a neurologist.

13. Other dementias: Cases are ascertained according to McKhann et al, 2011 criteria (see below) or if a diagnosis of dementia is reported by a neurologist.

Dementia is diagnosed when there are cognitive or behavioral (neuropsychiatric) symptoms that: 1. Interfere with the ability to function at work or at usual activities; and 2. Represent a decline from previous levels of cognitive functioning; and 3. Are not explained by delirium or major psychiatric disorder; 4. Cognitive impairment is detected and diagnosed through a combination of (1) history-taking from the patient and a knowledgeable informant and (2) an objective cognitive assessment; 5. The cognitive or behavioral impairment involves a minimum of two cognitive domains.

14. Parkinson’s disease. Cases are ascertained according to the diagnostic criteria described by Hughes et al (1992) or if reported by a neurologist.

15. Unipolar depression. The diagnosis must be made according to the DSM-V criteria. In this, definition major depression, persistent depression, and other depressions included in Depressive Disorders (DSM V) are accepted. Diagnosis of depression made by primary care physicians or psychiatrist in participants treated with antidepressant drugs for more than 6 moths is accepted. If this is the case, ICD 10 (International Statistical Classification of Diseases and Related Health Problems, 10th version) diagnosis of depressive episodes are also accepted. For physicians and psychiatrists not using ICD 10 or DSM V, a positive response to the two questions included in the NICE clinical guidelines is recommended (https://www.nice.org.uk).

16. Osteoporotic fractures. Low-energy fracture is defined as the fracture produced by a same-level fall. Fractures are identified from X-rays reports obtained from at least two

radiological reports. High trauma fractures, potentially pathological fractures (e.g., cancer or Paget's disease), or fractures of the head, fingers and toes are not considered.

17. Gallstone disease or cholecystectomy: Gallstone disease is diagnosed according to the findings obtained by imaging techniques including abdominal ultrasonography, computed tomography or magnetic resonance imaging. Diagnosis of cholecystectomy require the corresponding surgical report.

18. Symptomatic gout: Defined following the criteria of the *American College of Rheumatology*. Typically, the disease first presents as arthritis that is acute and episodic, but can be recurrent. Gout can also present as chronic arthritis of one or more joints. This clinical picture is built on a foundation of an excess body burden of uric acid, manifested in part by hyperuricemia, which is defined as serum uric acid levels greater than 7.0 mg/dL.

19. Transient Ischemic Attack: The diagnosis must be made according to the Scientific Statement of the American Heart Association/American Stroke Association Stroke Council: a transient episode of neurological dysfunction caused by focal brain, spinal cord, or retinal ischemia, without acute infarction demonstrated by neuroimaging, preferably magnetic resonance imaging techniques.

20. Cataract surgery: Defined by a medical report of cataract surgery.

21. Surgery for obesity: Defined by a medical report of bariatric surgery.

C. Intermediate markers

Changes in nutrient intake and dietary patterns will be determined by changes in the 17-item score of adherence to the energy-restricted Mediterranean diet (intensive intervention group) or the 14-item score (control group) and by changes in food and nutrient intake determined by the 143-item food frequency questionnaire administered during follow-up.

Changes in systolic and diastolic blood pressure, serum lipid concentrations, fasting glucose levels, renal function, uric acid, hemoglobin A1C, C-reactive protein, and liver function are evaluated yearly for the duration of the intervention.

Yearly, are also evaluated the percentage of participants in each group requiring anti-hypertensive, anti-diabetic or lipid-lowering medication, results of ECGs, cognitive function, quality of life, and psychological and neuropsychological questionnaire scores.

Analysis methods

Analysis methods

For primary and secondary outcomes –except for changes in weight and waist circumference– Cox regression models will be used for assessing the association between the intervention and the outcome. Time-at-risk will be given by the time between the baseline visit (in which the participants learn about their allocation group) and the date the outcome happened. For those participants who are right censored, follow-up will finish at the last available date of follow-up (last visit or last date in medical records, whichever occur later). For

participants with follow-up longer that the closing date of the database, the closing date of the database will be considered as censoring date. Also, for fatal outcomes, date of death will be considered as end of follow-up.

For changes in weight, we will used multilevel, mixed-effects linear regression models with repeated measurements with a random intercept and taking into consideration the intra-cluster correlation of members of the same household. We will assess within group changes as well as between group changes during follow-up. The center will be included as a random factor.

Also, for weight changes, we will define a weight loss of 5% as clinically meaningful (Williamson et al, 2015). Baseline body weight in the PREDIMED-Plus trial was 86.5 kg, thus a 5% loss is 4.3 kg. Two landmark trials of lifestyle interventions in high-risk population groups (with high BMI and impaired glucose tolerance, like many participants in the PREDIMED-Plus trial) achieved average weight losses of 3–6 kg, translating into highly significant reductions in diabetes risk (Tuomilehto et al, 2001, Knowler et al, 2002). Similar 58% reduction in diabetes risk were observed in both RCTs.

As an ancillary analysis to changes in weight, we will assess changes in BMI with BMI as a continuous trait with multilevel, mixed-effects linear regression models with repeated measurements with a random intercept and taking into consideration the intra-cluster correlation of members of the same household. We will assess within group changes as well as between group changes during follow-up. Also, we will consider a 5% loss in BMI as a clinically meaningful change. Baseline BMI in the PREDIMED-Plus cohort was 32.5 kg/m^2 hence a 5% loss is 1.6 kg/m^2. In a previous large prospective study (Feldman et al, 2017), among participants with baseline BMI 30-34.9 kg/m2, moderate loss (−3.0 to −7.0%) resulted in an OR for diabetes of 0.38 (95% CI, 0.19- 0.79). Therefore, 5% loss in BMI is definitively a change of a sufficient size as to be clinically meaningful.

Finally, for waist circumference changes, we will use multilevel, mixed-effects linear regression models with repeated measurements with a random intercept and taking into consideration the intra-cluster correlation of members of the same household and the center as another random factor. We will assess within group changes as well as between group changes during follow-up. As for weight and BMI change, a 5% reduction in waist circumference will be considered as clinically meaningful. Baseline waist circumference was 108 cm in the PREDIMED-Plus cohort and a 5% reduction is 5.4 cm. In the cited Finnish study (Tuomilehto et al, 2001) the lifestyle intervention that reduced diabetes risk by 58% resulted in a mean 4.4 cm reduction in waist circumference.

<u>Adjustment for covariates</u>

Main analyses will be crude analyses based on intention-to-treat approaches. Robust variance estimators will consider the intra-cluster correlation of members of the same household and Cox regression models will be stratified by center.

For the combined outcome, additional analyses will be stratified according to recruitment center and adjusted for sex, age, educational level, smoking status, baseline hypertension, baseline dyslipidemia, baseline type 2 diabetes, family history of coronary heart disease, body-mass index, waist-to-height ratio and baseline physical activity (METS-min/d, as derived from the self-reported physical activity questionnaire).

When assessing changes in weight as an outcome, models will be also adjusted for recruitment center, sex, age, educational level, smoking status, baseline hypertension,

baseline dyslipidemia, baseline diabetes, family history of coronary heart disease, and baseline physical activity.

Secondary outcomes may include some further specific confounders.

Methods used for assumptions to be checked for statistical methods

Proportionality of the hazards will be assessed with Schoenfeld's residual test and testing time-varying-covariates.

Alternative methods to be used if distributional assumptions do not hold

If hazards are not proportional, we will reassess the proportionality assumption by including some potential confounders as strata. If hazards remain non-proportional, we will describe the effect of the intervention separately for different follow-up periods.

Planned sensitivity analyses for the outcomes

Main analyses will be based on an intention-to-treat approach with completers only. As sensitivity analysis, the intention-to-treat approach will be repeated with multiple imputation for missing outcomes (see below).

Also, per-protocol analyses will be done for the primary outcome.

Planned subgroup analyses

Subgroup analyses will be done by sex, age (median age as cut-off point), educational level (2 categories), baseline diabetes, number of criteria for the metabolic syndrome (3 vs. >=4), smoking, body-mass index (obese and non-obese), and baseline score of adherence to the energy-reduced Mediterranean diet.

Missing data

Multivariate imputation will be done with chained equations (STATA "mi" command), generating 20 imputations for each missing measurement from regression equations to predict missing outcomes among participants lost for 2 years or longer. The imputation models included as predictors will be sex, age, smoking, leisure-time physical activity, baseline BMI, baseline weight, prevalent diabetes, prevalent hypertension, prevalent hypercholesterolemia, family history of coronary heart disease, intervention group, being 2nd member of the same household and educational level.

Additional analyses

For the per-protocol analysis, causal inference methods will be used (inverse probability weighting, G-formula) (Estruch et al, 2018).

Let A_j be 1 if the participant adheres to the intervention, and 0 otherwise (from the moment on when he/she do no longer adhere to it). The per-protocol effect is the effect of *Aj*, and can be estimated using the same approach as the intention-to-treat effect with one

important difference: individuals are artificially censored at the end of the interval when they deviate from the study protocol because they stopped adhering to the intervention, i.e., when Aj=0.

Of course, to determine whether participants adhere during given year, they must provide information on adherence at the subsequent follow-up visit, and for that, they must attend the follow-up visit in the first place. Therefore, there are 3 different censoring mechanisms in this per-protocol analysis of interval studies:

1) Incomplete follow-up. This type of censoring arises when individuals do not attend a visit (with a pre-specified period of 12 months). Let C_j be an indicator of censoring by incomplete follow-up at month j.

2) Insufficient information to determine adherence among those who attend a visit. Let N_j be an indicator for attending a visit at time j (1: yes, 0: no). Among those with N_j=1, we define the censoring indicator R_j (1: yes, 0: no) for missing information on A_j.

3) No adherence. Among those with N_j=1 and R_j=0, participants are censored if A_j=0

Censoring by any of the above mechanisms may introduce bias. We will estimate inverse probability weights to adjust for the potential selection bias (Hernán, 2013; Hernán, in press) under the assumption that loss to follow-up, data collection, and adherence were effectively randomized at each time point given the measured pre- and post-randomization prognostic factors. Information on the specific methods that should be used to estimate the probabilities, to compute the stabilized weights, and to fit the models, together with an example, can be found in Estruch et al, 2018, Supplemental appendix, pages 36-38.

Harms

Information on adverse effects (headache, fatigue, constipation and increased bowel rhythm) is collected at 6 months and yearly thereafter. This information will be described as percentage across intervention groups in the report of the primary outcome.

References

Alberti KG, Eckel RH, Grundy SM, Zimmet PZ, Cleeman JI, Donato KA, et al, Harmonizing the metabolic syndrome: a joint interim statement of the International Diabetes Federation Task Force on Epidemiology and Prevention; National Heart, Lung, and Blood Institute; American Heart Association; World Heart Federation; International Atherosclerosis Society; and International Association for the Study of Obesity. Circulation. 2009;120(16):1640-5.

Babio N, Toledo E, Estruch R, Ros E, Martínez-González MA, Castañer O, et al, Mediterranean diets and metabolic syndrome status in the PREDIMED randomized trial. CMAJ. 2014;186(17):E649-57.

Cholesterol Treatment Trialists' (CTT) Collaboration. Efficacy and safety of more intensive lowering of LDL cholesterol: a meta-analysis of data from 170,000 participants in 26 randomised trials. Lancet. 2010;376:1670-81

Estruch R, Ros E, Salas-Salvadó J, Covas MI, Corella D, Arós F, et al, Primary prevention of cardiovascular disease with a Mediterranean diet. N Engl J Med. 2018 Jun 21;378(25):e34.

Fernández JM, Rosado-Álvarez D, Da Silva Grigoletto ME, Rangel-Zúñiga OA, Landaeta-Díaz LL, Caballero-Villarraso J, López-Miranda J, Pérez-Jiménez F, Fuentes-Jiménez F. Moderate-to-high-intensity training and a hypocaloric Mediterranean diet enhance endothelial progenitor cells and fitness in subjects with the metabolic syndrome. Clin Sci (Lond). 2012;123(6):361-73.

Feldman AL, Griffin SJ, Ahern AL, Long GH, Weinehall L, Fhärm E, Norberg M, Wennberg P. Impact of weight maintenance and loss on diabetes risk and burden: a population-based study in 33,184 participants. BMC Public Health. 2017;17(1):170.

González-Muniesa P, Martínez-González MA, Hu FB, Després JP, Matsuzawa Y, Loos RJF, et al, Obesity. Nat Rev Dis 2017; 3:17034.

Gordon DJ, Probstfield JL, Garrison RJ, et al, High-density lipoprotein cholesterol and cardiovascular disease. Four prospective American studies. Circulation. 1989;79:8–15.

Hernán MA, Hernández-Díaz S, Robins JM. Randomized trials analyzed as observational studies. Ann Intern Med 2013;159:560-2.

Hernán MA, Robins JM. Causal inference. Boca Raton, FL: Chapman & Hall/CRC (in press).

Hughes AJ, Ben-Shlomo Y, Daniel SE, Lees AJ. What features improve the accuracy of clinical diagnosis in Parkinson's disease: a clinicopathologic study. Neurology 1992;42:1142-6. Erratum in: Neurology 1992;42(7):1436.

Kastorini CM, Milionis HJ, Esposito K, Giugliano D, Goudevenos JA, Panagiotakos DB. The effect of Mediterranean diet on metabolic syndrome and its components: a meta-analysis of 50 studies and 534,906 individuals. J Am Coll Cardiol. 2011;57(11):1299-313.

Knowler WC, Barrett-Connor E, Fowler SE, Hamman RF, Lachin JM, Walker EA, Nathan DM, Diabetes Prevention Program Research G. Reduction in the incidence of type 2 diabetes with lifestyle intervention or metformin. N Engl J Med. 2002;346(6):393–403.

Lange T, Hansen J V. Direct and Indirect Effects in a Survival Context. Epidemiology. 2011;22(4):575-581.

Lin S-H, Young JG, Logan R, VanderWeele TJ. Mediation analysis for a survival outcome with time-varying exposures, mediators, and confounders. Stat Med. 2017;36(26):4153-4166.

Look AHEAD Research Group, Wing RR. Long-term effects of a lifestyle intervention on weight and cardiovascular risk factors in individuals with type 2 diabetes mellitus: four-year results of the Look AHEAD trial. Arch Intern Med. 2010;170(17):1566-75.

Martínez-González MA, Corella D, Salas-Salvadó J, Ros E, Covas MI, Fiol M, et al, Cohort profile: design and methods of the PREDIMED study. Int J Epidemiol 2012; 41:377-85.

Martínez-González MA, Buil-Cosiales P, Corella D, Bulló M, Fitó M, Vioque J, et al, Cohort Profile: Design and methods of the PREDIMED-Plus randomized trial. Int J Epidemiol. 2018 Nov 22. doi: 10.1093/ije/dyy225. [Epub ahead of print]

McKhann GM, Knopman DS, Chertkow H, Hyman BT, Jack CR Jr, Kawas CH, et al. The diagnosis of dementia due to Alzheimer's disease: recommendations from the National Institute on Aging-Alzheimer's Association workgroups on diagnostic guidelines for Alzheimer's disease. Alzheimers Dement. 2011;7(3):263-9.

Miller M, Stone NJ, Ballantyne C, Bittner V, Criqui MH, Ginsberg HN, et al. Triglycerides and cardiovascular disease: a scientific statement from the American Heart Association. Circulation. 2011;123(20):2292-333.

Nordestgaard BG, Varbo A. Triglycerides and cardiovascular disease. Lancet. 2014;384:626–35.

O'Brien PC, Fleming TR. A multiple testing procedure for clinical trials. Biometrics. 1979;35(3):549-56.

Sacks FM, Bray GA, Carey VJ, Smith SR, Ryan DH, Anton SD, McManus K, Champagne CM, Bishop LM, Laranjo N, Leboff MS, Rood JC, de Jonge L, Greenway FL, Loria CM, Obarzanek E, Williamson DA. Comparison of weight-loss diets with different compositions of fat, protein, and carbohydrates. N Engl J Med. 2009;360(9):859-73.

Salas-Salvadó J, Bulló M, Estruch R, Ros E, Covas MI, Ibarrola-Jurado N, Corella D, Arós F, Gómez-Gracia E, Ruiz-Gutiérrez V, Romaguera D, Lapetra J, Lamuela-Raventós RM, Serra-Majem L, Pintó X, Basora J, Muñoz MA, Sorlí JV, Martínez-González MA. Prevention of diabetes with Mediterranean diets: a subgroup analysis of a randomized trial. Ann Intern Med. 2014;160(1):1-10.

Schröder H, Fitó M, Estruch R, Martínez-González MA, Corella D, Salas-Salvadó J, et al, A short screener is valid for assessing Mediterranean diet adherence among older Spanish men and women. J Nutr 2011;141(6):1140-5.

Schulz KF, Grimes DA. Multiplicity in randomised trials II: subgroup and interim analyses. Lancet 2005;365(9471):1657-61.

Schwarzfuchs D, Golan R, Shai I. Four-year follow-up after two-year dietary interventions. N Engl J Med 2012; 367:1373-4.

Shai I, Schwarzfuchs D, Henkin Y, Shahar DR, Witkow S, Greenberg I, et al, Weight loss with a low-carbohydrate, Mediterranean, or low-fat diet. N Engl J Med 2008; 359:229-41.

Stamler J. Blood pressure and high blood pressure. Aspects of risk. Hypertension 1991;18(3 Suppl):I95-107.

Stamler R. Implications of the INTERSALT study. Hypertension 1991;17(1Suppl):I16–20.

The GBD 2015 Obesity Collaborators. Health effects of overweight and obesity in 195 countries over 25 years. N Engl J Med 2017; 377:13-27.

Thompson WA Jr. On the treatment of grouped observations in life studies. Biometrics 1977;33:463-70.

Tuomilehto J, Lindström J, Eriksson JG, Valle TT, Hämäläinen H, Ilanne-Parikka P, Keinänen-Kiukaanniemi S, Laakso M, Louheranta A, Rastas M, Salminen V, Uusitupa M; Finnish Diabetes

Prevention Study Group. Prevention of type 2 diabetes mellitus by changes in lifestyle among subjects with impaired glucose tolerance. N Engl J Med. 2001;344(18):1343-50.

Williamson DA, Bray GA, Ryan DH. Is 5% weight loss a satisfactory criterion to define clinically significant weight loss? Obesity (Silver Spring). 2015;23(12):2319-20.
