# Supplementary material for: An Energy-Reduced Mediterranean Diet, Physical Activity, and Body Composition: An Interim Subgroup Analysis of the PREDIMED-Plus Randomized Clinical Trial
Source: JAMA Netw Open. 2023 Oct 18;6(10):e2337994. doi: 10.1001/jamanetworkopen.2023.37994 (PMC10585413; doi:10.1001/jamanetworkopen.2023.37994)
Supplement: Supplement 4. — Data Sharing Statement [file jamanetwopen-e2337994-s004.pdf]

Data Sharing Statement

Konieczna. An Energy-Reduced Mediterranean Diet, Physical Activity, and Body Composition. *JAMA Netw Open*. Published October 18, 2023. doi:10.1001/jamanetworkopen.2023.37994

Data
Data available: Yes
Data types: Deidentified participant data
How to access data: There are restrictions on the availability of data for the PREDIMED-Plus study, due to the signed consent agreements around data sharing, which only allow access to external researchers for research following the project purposes. Requestors wishing to access the PREDIMED-Plus trial data used in this study can request it to the PREDIMED-Plus trial Steering Committee: predimed_plus_scommittee@googlegroups.com
When available: With publication

Supporting Documents
Document types: None

Additional Information
Who can access the data: To external researchers for research following the project purposes. Requestors wishing to access the PREDIMED-Plus trial data used in this study can request it to the PREDIMED-Plus trial Steering Committee: predimed_plus_scommittee@googlegroups.com.
Types of analyses: Specified purpose
Mechanisms of data availability: After approval of a proposal
